# The Preparatory (Anti)Bonding Character of Molecular Orbitals

**DOI:** 10.1002/advs.76530

**Published:** 2026-07-13

**Authors:** Jonas O. Wenzel, Johannes Werner, Pascal Weisenburger, Joachim Podlech, Ralf Köppe, Ingo Krossing, Dieter Fenske, Andreas Reiß, Claus Feldmann, Israel Fernández, Frank Breher

**Affiliations:** ^1^ Karlsruhe Institute of Technology (KIT) Institute For Inorganic Chemistry (AOC) Karlsruhe Germany; ^2^ Departamento De Química Orgánica Facultad De Ciencias Químicas Universidad Complutense De Madrid Madrid Spain; ^3^ Karlsruhe Institute of Technology (KIT) Institute of Physical Chemistry (IPC) Karlsruhe Germany; ^4^ Karlsruhe Institute of Technology (KIT) Institute of Organic Chemistry (IOC) Karlsruhe Germany; ^5^ University of Freiburg Institute For Inorganic and Analytical Chemistry (IAAC) Freiburg Germany; ^6^ Karlsruhe Institute of Technology (KIT) Institute of Nanotechnology (INT) Karlsruhe Germany

**Keywords:** atomic orbital, bond order, chemical bond, chemistry, homolysis, molecular orbital, molecular orbital diagram, molecular orbital theory, natural bond orbital, valence bond theory

## Abstract

The strengths of chemical bonds define our world by influencing chemical reactivity and molecular structure. This work presents unpredicted changes in bonding energies of covalent aluminum‐carbon bonds in isolated molecular aluminum complexes while populating remote ligand‐centered orbitals. Their chemical bonding situation was investigated computationally by energy decomposition analysis, which revealed that populating frontier molecular orbitals enables the relaxation (or preparation) of homolysis products, consequently influencing bonding strength. It was demonstrated in the case of several organic, inorganic, and organometallic standard molecules that generally bond dissociation energies can change upon the population of frontier preparatory (anti)bonding molecular orbitals, which do not necessarily have to show orbital coefficients at the chemical bond. The concept of preparatory character of orbitals allows qualitative predictions of trends in bonding energies upon oxidation or reduction of molecular compounds, being a valuable addition to molecular orbital theory across all fields of chemistry.

## Graphical abstract

Herein, a concept to qualitatively predict changes in bond dissociation energies upon reduction or oxidation of molecular compounds is introduced. Changing the population of so‐called preparatory molecular orbitals influences how molecular fragments are relaxing after bond cleavage, which is shown for isolated organometallic aluminum compounds as well as a variety of standard molecules like acetylene or benzene.

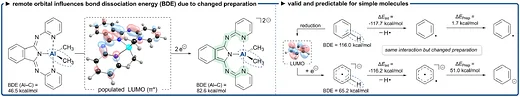

## 1 Main Text

The nature and strength of a chemical bond between two atoms is a fundamental question in chemistry [1, 2] influencing molecular structure [3, 4], chemical reactivity [5, 6], and consequently all sciences that are based on these measures like biochemistry [7, 8], materials science [9], or energy conversion technology [10]. The energy required to homolytically cleave a chemical bond is called the bond dissociation energy (BDE) and is the most common metric for bonding strength [11, 12, 13]. By definition, the BDE is connected to the thermodynamic stability of the radical homolysis products. In this regard, it is important to distinguish between the BDE of one isolated chemical bond, which is considered in this work, and the average BDE (BDE_mean_) of all bonds within the molecule, which can be quite different (Figure 1A). The formation of radicals is important for organic synthesis [14], catalysis [15, 16], or radical polymerization [17]. Especially in active research fields like electrosynthesis [18, 19], proton‐coupled electron transfer (PCET) [20, 21], and photoredox [22, 23, 24] or electron catalysis [25, 26], the alteration of BDEs upon reduction/oxidation of chemical compounds is relevant.

**FIGURE 1:**
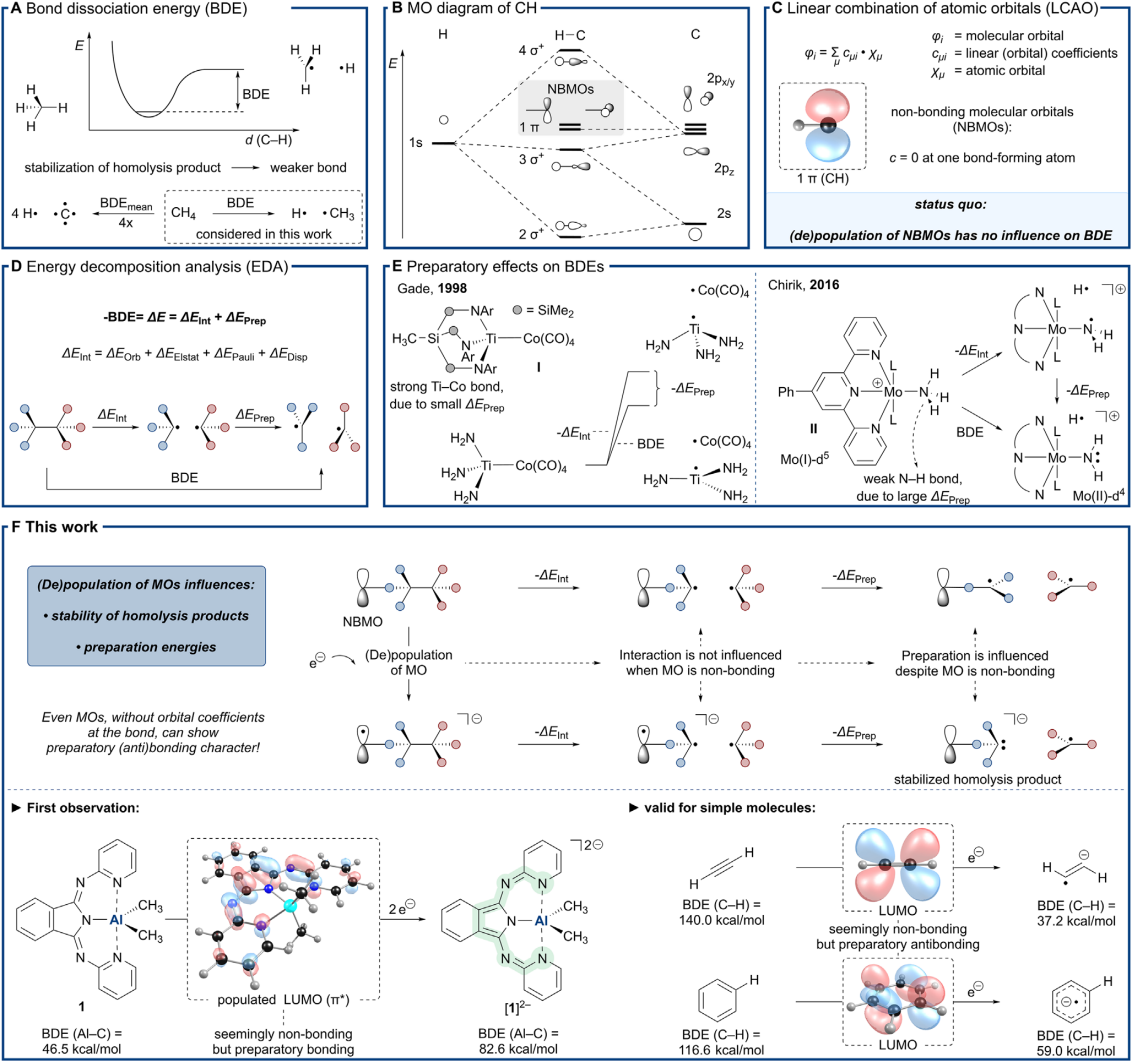
(A) Schematic depiction of a potential energy surface showing the homolysis of the C─H bond in methane to visualize the bond dissociation energy (BDE) and reaction equations to show the different definitions of BDE and average BDEs (BDE_mean_). (B) Schematic molecular orbital diagram of methyne (CH) to clarify the origin of non‐bonding molecular orbitals (NBMOs). (C) General LCAO formulation of molecular orbital theory for the construction of molecular orbitals (MOs) from atomic orbitals (AOs) and depiction of the NBMO in methyne. *i* orbital index, *µ* atom index. (D) Equation defined within energy decomposition analysis (EDA) to express bond dissociation energies (BDEs) as a function of the intrinsic interaction energy (Δ*E*_Int_) and the preparation energy (Δ*E*_Prep_), and schematic representation of the equation upon the homolysis of a C─C bond. Within EDA, Δ*E*_Int_ is further separated into the orbital interaction term (Δ*E*_Orb_), the electrostatic interaction (Δ*E*_Elstat_), the Pauli repulsion (Δ*E*_Pauli_), and the dispersion interaction (Δ*E*_Disp_). (E) Reported examples in which BDEs were severely influenced by tuning preparation energies (L = PPh_2_Me). (F) Schematic representation of this work: MOs can show preparatory bonding character when their (de)population changes how the molecule is geometrically and electronically relaxing after bond fragmentation. By this means, also the (de)population of NBMOs can influence BDEs. The first observation of this phenomenon is described based on Al─C bonds within bis(pyridylimino) isoindolide (BPI) aluminum complexes, but the concept is also shown to be applicable to a variety of small standard molecules such as acetylene or benzene. Given BDEs obtained at the PBE0‐D3BJ/def2‐TZVPD/CPCM(THF) level.

The concepts that scientists intuitively think about chemical bonding are mainly based on molecular orbital (MO) theory [27, 28, 29, 30, 31], which was shaped by pioneers like Friedrich Hund [32], Robert Mulliken [33], John Lennard‐Jones [34], or Erich Hückel [35] (Figure 1B). For the prediction of how BDEs are changing upon reduction or oxidation, most chemists would look at the molecular orbital, which is getting populated or depopulated, respectively, and rely on concepts of MO theory. The basic approach in MO theory is the linear combination of atomic orbitals (LCAO), which gives a set of MOs with either bonding, antibonding, or non‐bonding character.^1^ The contribution of one former AO to a MO is represented by the LCAO coefficients (orbital coefficients). Orbitals without orbital coefficients at both bond‐forming atoms are non‐bonding MOs (NBMOs, e.g., *p*_x,y_ orbitals of the carbon atom in CH, Figure 1C). The International Union of Pure and Applied Chemistry (IUPAC) defines NBMOs as “*molecular orbitals whose occupation by electrons does not contribute (significantly) to the binding energy of the molecule*” [36]. With that in mind, we ask the readership a central question of this work: Does the C─H bond energy in acetylene (H─C≡C─H) change upon reduction, hence, the population of the π* orbitals, which are *perpendicular* to the C─H bond? One might argue that electrostatic interactions and s‐ and/or p‐character of orbitals are changed in the radical anion [H─C≡C─H]^•–^, but based on the above‐described assumptions, the answer should be ‘*no*’ or at most ‘*little, but insignificantly*’. But, in the following, we will explain why the answer must instead be ‘*yes*’.

In this work, we describe bond homolysis by using the formalism of Morokuma's energy decomposition analysis (EDA) [37, 38, 39]. The bond dissociation energy (BDE or ‐Δ*E*) is defined as sum of the intrinsic interaction energy (‐Δ*E*_Int_) between both bond‐forming fragments and the preparation energy (‐Δ*E*_Prep_), that accounts for electronic and structural reorganization of the fragments from their initial geometries to those adopted in the final homolysis products (or thought reversely to ‘prepare the homolysis products for bonding’, Figure 1D) [38]. BDEs can be strongly influenced by electronic and structural relaxation (preparation) of the homolysis products, without changing the intrinsic interaction between the atoms [40, 41]. This was, for example, described by Gade for geometrically fixed metal complexes (Figure 1E,I) [42] or is considered an important mechanism in the coordination‐induced bond‐weakening (CIBW) [43], e.g., in Chirik's molybdenum complex **II** (detailed explanation in Supporting Information) [44].

Our group is interested in the BDEs of Al─C bonds in aluminum complexes bearing the redox‐active bis(pyridylimino) isoindolide (BPI) ligand (e.g., [BPI–AlMe_2_] **1**) [45, 46]. Herein, we report the isolation of singly and doubly reduced derivatives of **1**. Only for the latter, we observed a dramatic increase in Al–C BDE, although remote ligand‐centered orbitals without orbital coefficients at the organometallic fragment were populated. The reason for bond strengthening is that the population of frontier molecular orbitals (FMOs) changes the stability of homolysis products, which is equivalent to a change in preparation and unequivocally a change in BDE. While we elaborated this phenomenon for the first time when looking at our particular organometallic system, we show this concept to be indeed valid for non‐bonding, bonding, and antibonding MOs in general within standard molecules that occur in every day's life of a chemist (Figure 1F).

## 2 Synthesis and Characterization of the Key Molecules

Indications that [BPI–AlMe_2_] (**1**) represents a suitable platform for isolating isostructural reduced compounds were obtained by cyclic voltammetry (CV) in tetrahydrofuran (THF), revealing two quasi‐reversible reductions at *E* ^0^_1/2_ = −1.86 and −2.26 V (vs. Fc^+/0^). We isolated [K(thf)_x_][BPI–AlMe_2_] (**2**) by treating **1** with one equivalent of potassium in THF (Figure 2A), which leads to a color change from bright yellow to dark red, severe air sensitivity, absence of signals in nuclear magnetic resonance (NMR) spectra, and one single line in continuous‐wave electron paramagnetic resonance (cw‐EPR) spectroscopic measurements (Figure 2B). Single crystals were obtained after **2** was treated with one equivalent of 18‐crown‐6 or 2.2.2‐cryptand. The molecular structures of [K(18‐crown‐6)][BPI–AlMe_2_] (**3**) and [K(2.2.2‐cryptand)][BPI–AlMe_2_] (**4**) were elucidated by single‐crystal X‐ray diffraction (SC‐XRD) (Figure 2C). Compound **3** represents the radical anion [**1**]^•–^, coordinating via the bridging nitrogen atom (N2) to the potassium ion, whose coordination sphere is saturated by the crown ether. **4** displays a non‐coordinating ion pair of the radical anion [**1**]^•–^ and the cation [K(2.2.2‐cryptand)]^+^. The molecular structure of the radical anion in **3** and **4** is identical, thus independent of the coordination of the potassium ion to the nitrogen atom N2. Compared to the molecular structure of **1** [47], the former C = N imine bond (C2–N2) in **3/4** is 4.5/4.2 pm longer, while the C2–C3 atom distance is 3.7/3.6 pm shorter. Such bond length alterations are the consequence of populated π* orbitals of the BPI ligand [48, 49]. Together with the still trigonal‐bipyramidal coordination environment around the aluminum atom, it follows that the reduction of **1** occurs fully ligand‐centered, leaving the aluminum atom still in its highest (formal) valency +III, as it was already reported for several aluminum complexes of redox non‐innocent ligands (NILs) [50]. The Al─C bond lengths within **3** and **4** are 198.9 and 200.2 pm, respectively, which is within the range of all other reported Al─C bonds for compounds of the type [BPI–AlR_2_] with sp^3^‐hybridized carbon moieties (198.4–201.6 pm) [45], and indicates neither bond weakening nor strengthening of the Al─C bond upon ligand reduction. Electronation of **1** with decamethyl cobaltocene (CoCp*_2_, Cp = cyclopentamethylid) yielded [CoCp*_2_][BPI–AlMe_2_] (**5**), which likewise does not show any contact between the metallocenium and aluminum ion and confirms the structural parameters for the free radical anion [**1**]^•–^ (see Supporting Information).

**FIGURE 2:**
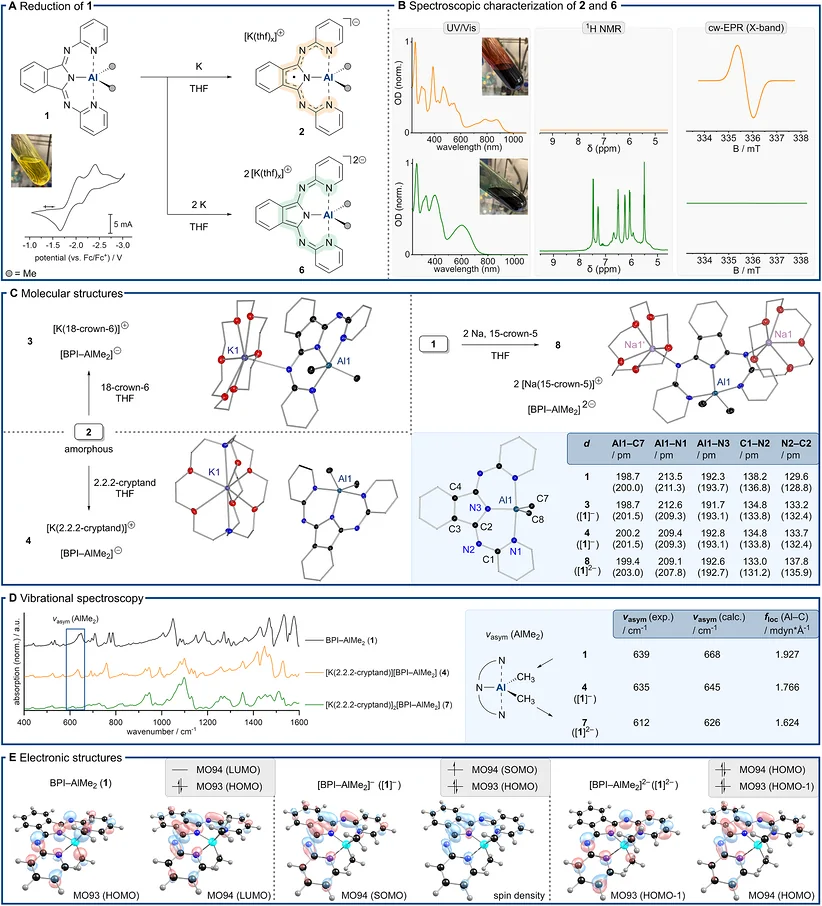
(A) Reaction scheme for the synthesis of **2** by reduction of **1** (conditions: 1.00 equiv. K, THF, r.t., overnight, quant.) and synthesis of **6** by reduction of **1** (conditions: 2.00 equiv. or excess K, THF, r.t., overnight, quant.), as well as cyclic voltammogram of **1** (THF, Pt/[NBu_4_][Al(OtBu^F^)_4_]/Ag, *v* = 100 mV/s, potential given vs. Fc/Fc^+^). (B) Spectroscopic characterization of **2** and **6** by UV/Vis (1 mmol/L in THF, d = 1 mm), ^1^H NMR (THF‐*d_8_*) and cw‐EPR spectroscopy (isotropic at r.t., ∼0.1 mmol/L in THF, 9.41 MHz). (C) SC‐XRD structures and reaction schemes for the synthesis of **3**, **4**, and **8** and a table with selected experimental bond lengths of **1**, **3**, **4**, and **8** in direct comparison to theoretical bond lengths of **1**, [1]^•–^ and [1]^2–^ in brackets, obtained by geometry optimization at the PBE0‐D3BJ/def2‐TZVP/CPCM(THF) level. (D) Vibrational analysis by infrared (IR) spectroscopy on **1**, **4**, and **7**. Theoretical vibrational frequencies were obtained at the PBE0‐D3BJ/def2‐TZVP level, and local force constants were obtained using LModeA‐nano. (E) Schematic representation of the orbital occupation as well as selected MO and spin density plots of **1**, [1]^•–^, and [1]^2–^, obtained at the PBE0‐D3BJ/def2‐TZVPD/CPCM(THF) level. MOs are depicted with an isovalue of 0.05 and spin densities with 0.005 a.u.

When **1** was treated with two equivalents or an excess of potassium in THF, a color change from bright yellow to dark red and to dark green was observed. A compound with the general constitution [K(thf)_x_]_2_[BPI–AlMe_2_] (**6**) was isolated (Figure 2A). **6** shows EPR inactivity and broadened NMR signals in the range between 5 and 8 ppm (Figure 2B), which are strongly shifted to high field compared to **1**. The addition of 2.2.2‐cryptand led to the isolation of [K(2.2.2‐cryptand)]_2_[BPI–AlMe_2_] (**7**), supporting the successful synthesis of a dianionic [**1**]^2–^ compound (see Supporting Information). Moreover, the use of sodium as a reductant and 15‐crown‐5 as a chelating agent enabled the synthesis and isolation of single crystals of [Na(15‐crown‐5)]_2_[BPI–AlMe_2_] (**8**) (Figure 2C). The BPI bond lengths observed in **8** are consistent with a doubly reduced, i.e., trianionic BPI ligand, while the complex remains trigonal‐bipyramidal. The Al─C bond length of 199.4 pm again does not differ significantly from the previously discussed molecular structures of **1**, **3**, or **4**. Infrared (IR) spectroscopy on **1**, **4**_,_ and **7** revealed only very slightly weakened antisymmetric Al–C stretching vibrations of 639, 635, and 612 cm^−1^, respectively (Figure 2D).

The free anion [**1**]^•–^ and the free dianion [**1**]^2–^ were investigated by density functional theory (DFT) computations. Geometry optimization and vibrational frequency calculations reproduced experimental molecular structures and IR spectra well. Upon reduction of **1**, no qualitative change of the frontier molecular orbitals is observed, implying that MO93, the highest occupied molecular orbital (HOMO) of **1**, is still the HOMO in [**1**]^•–^ and the HOMO‐1 in [**1**]^2–^. Equally, the LUMO of **1** (MO94) becomes, as expected, the singly occupied molecular orbital (SOMO) of [**1**]^•–^ and the HOMO of [**1**]^2–^ (Figure 2E). MO94 is fully ligand‐centered and does not show any orbital coefficient at the aluminum atom or the carbon atoms of the methyl moieties, which even lie within the nodal planes of this putative non‐bonding molecular orbital.

## 3 BDEs and EDA on Aluminum Organometallics

The Al–C BDEs within **1**, [**1**]^•–^ and [**1**]^2–^ were computed to 46.5 kcal/mol, 47.6 kcal/mol, and 82.6 kcal/mol, respectively, implying a slight change during the first reduction but a tremendous increase during the second one (Figure 3). This trend was observed for varied basis sets, DFT functionals, or by conducting highly accurate domain‐based local pair natural orbital coupled‐cluster (DLPNO‐CCSD(T)) calculations, which is considered a benchmark level of theory (see Supporting Information). As postulated previously [43], the BDE(Al–C) in **1** is unusually low for aluminum organometallics, which is mainly due to stabilization of the homolysis product [BPI–AlMe]^•^ ([**9**]^•^) by delocalization of the unpaired electron. As a comparison, the *vertical* fragmentation of the trimethyl aluminum dimer (Al_2_Me_6_) to the structurally constrained fragments [CH_3_]^•^ and [Al_2_Me_5_]^•^ requires overcoming an energy of 97.1 kcal/mol. Structural relaxation (geometry optimization) of both fragments to the *adiabatic* state releases 10.1 kcal/mol in energy, 9.9 kcal/mol of which originates from [CH_3_]^•^ going from the *C*_3v_ structure to the trigonal planar *D*_3h_ structure and only 0.2 kcal/mol originates from structurally barely relaxed [Al_2_Me_5_]^•^ (Figure 3A). The same energy scheme for **1** reveals a very similar Δ*E*_Int_ (−96.3 kcal/mol), but the relaxation from the constrained fragment [BPI–AlMe]^•^ ([**9**_Frag_]^•^) with sawhorse‐like‐coordination sphere around the aluminum atom to the distorted tetrahedrally coordinated actual homolysis product [9]^•^ is connected to a significantly higher preparation energy of 34.1 kcal/mol (Figure 3B). In other words, the homolysis product [**9**]^•^ is thermodynamically strongly stabilized by spin density delocalization, because a comparatively stable ligand‐centered radical is formed, rather than a highly energetic alanyl radical. The spin density distribution in [**9**]^•^ mimics that of the radical anion [**1**]^•–^. Furthermore, the SOMO of [**9**]^•^ qualitatively corresponds to the SOMO of [**1**]^•–^ and therefore to the MO94 of the [BPI–AlMe_2_] system in general. We conclude that MO94 is correlated with an MO of the homolysis product [**9**]^•^, which is mainly responsible for the delocalization of the unpaired electron and consequently also for the unusually low BDE of **1**.

**FIGURE 3:**
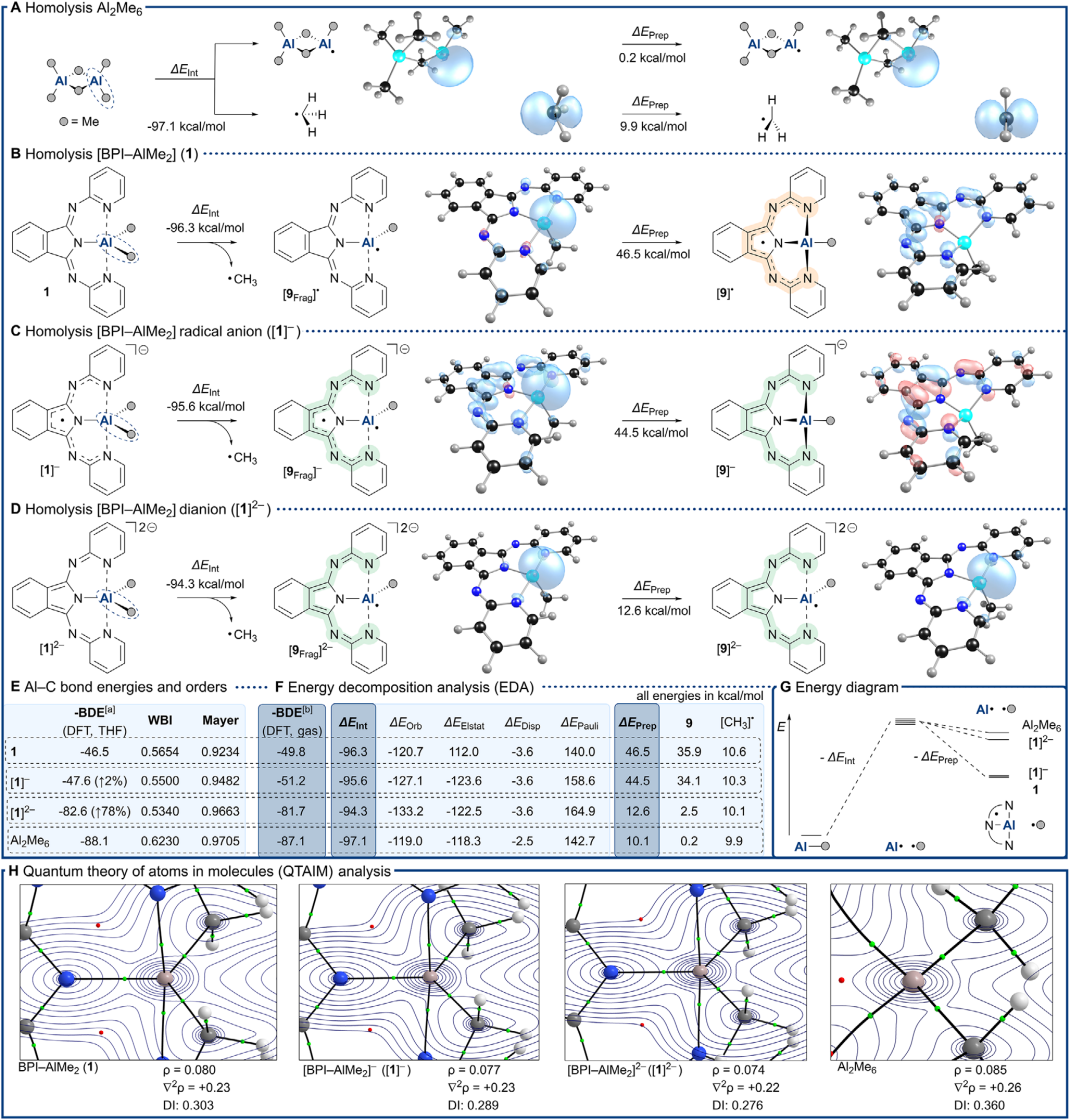
Fragmentation of the Al–C bond in Al_2_Me_6_ (**A**), **1** (**B**), [1]^–^ (**C**) and [1]^2–^ (**D**) as well as the reorganization to the actual homolysis products. Spin density plots are depicted for open‐shell fragments / homolysis products and HOMO plots are depicted for closed‐shell homolysis products, both were obtained at the PBE0‐D3BJ/def2‐TZVPD/CPCM(THF) level. (E) Table showing the bond dissociation energy (BDE) as well as the Wiberg bond index and the Mayer bond order of the Al–C bonds in Al_2_Me_6_, **1**, [1]^–^, and [1]^2–^. All values were obtained at the PBE0‐D3BJ/def2‐TZVPD/CPCM(THF) level. (F) Table showing the energy decomposition of ‐BDE into Δ*E*_Prep_ and Δ*E*_Int_ and further into Δ*E*_Orb_, Δ*E*_Elstat_, Δ*E*_Disp_, and Δ*E*_Pauli_ at the PBE0‐D3BJ/TZ2P level of theory. (G) Energy diagram visualizing the magnitudes of ‐BDE, Δ*E*_Int_, and Δ*E*_Prep_. (H) Contour plot of the electron density distribution for **1**, [1]^–^, and [1]^2–^, obtained during quantum theory of atoms in molecules (QTAIM) analysis (ρ: electron density, ∇^2^r: Laplacian of the electron density, DI: delocalization index).

The values for BDE, Δ*E*_Int_ and Δ*E*_Prep_ in [**1**]^•–^ are quite similar to those of the neutral derivative. In this case, the fragmentation needs 95.6 kcal/mol to yield both *vertical* particles [CH_3_]^•^ (*C*_3v_) and the structurally constrained [BPI–AlMe]^–^ ([**9**_Frag_]^–^), the latter in its triplet state with one unpaired electron residing on the BPI ligand and one unpaired electron at the aluminum atom. This state is called ‘prepared‐for‐bonding’ during EDA procedures. Preparation of [**9**_Frag_]^–^ to the relaxed homolysis product [**9**]^–^ implies a change in electron configuration and structural relaxation into the distorted tetrahedral coordination mode corresponding to a Δ*E*_Prep_ of 34.1 kcal/mol. The homolysis product [**9**]^–^ is a closed‐shell singlet species with a doubly reduced, thus trianionic BPI ligand and the aluminum atom in formal oxidation state +III. In other words, the delocalization of the unpaired electron formed by Al–C bond homolysis into the BPI scaffold, which is thermodynamically stabilizing the homolysis product, is still feasible for the anionic system [**1**]^•–^ leading to a similar bond energy as for **1** (Figure 3C). The situation changes for the dianionic system (Figure 3D). While Δ*E*_Int_ of [**1**]^2–^ still amounts to ‐94.3 kcal/mol, Δ*E*_Prep_ is smaller with only 12.7 kcal/mol, most of it originating from the relaxing methyl radical. The initial fragment [**9**_Frag_]^2–^ does only slightly relax electronically and structurally to the radical [**9**]^2–^. This homolysis product is now indeed a (rather unfavorable) alanyl radical with formal oxidation state +II at the aluminum atom and a doubly reduced BPI ligand, as indicated by an aluminum‐centered spin density and BPI bond lengths indicative of the trianionic redox state [48]. As a consequence, the BDE for [**1**]^2–^ is most similar to that of Al_2_Me_6_, because the unpaired electron formed during bond homolysis cannot be delocalized to the already doubly reduced BPI ligand, leading to a substantially less stabilized homolysis product (Figure 3E–G).

The intrinsic *vertical* interaction between the [BPI–AlMe] and CH_3_ radical fragments remains almost unchanged but is becoming slightly weaker upon reduction of the BPI ligand. This almost unaltered bonding situation is supported by only slightly varying values for Δ*E*_Int_ (2% deviation) and minor deviations in the decomposed individual EDA terms as Δ*E*_Int_, Δ*E*_Orb_, Δ*E*_Elstat_, Δ*E*_Disp_, or Δ*E*_Pauli_. The Wiberg bond index within natural bond orbitals (NBO) analysis (WBI, 6% deviation), Mayer bond order (5% deviation), and the Al–C atom distance (1% deviation) are only minorly deviating as well. This is consistent with almost identical experimental Al–C bond lengths (1% deviation) and stretching vibrations (4% deviation, Figure 2D). Furthermore, quantum theory of atoms in molecules (QTAIM) analyses on all four compounds revealed only slight changes in the electron density delocalization index (DI) and nearly identical bond critical points and electron density distributions at the Al─C bonds (Figure 3H). Still, the BDE increases by 78% going from **1** to [**1**]^2–^ due to a change in preparation energy. We conclude that the population of the non‐bonding MO94 does not significantly change the intrinsic interaction between the [BPI–AlMe] and CH_3_ fragments. The populated MO94 is correlated with an MO of the fragment, which enables spin density delocalization, thus stabilization of the homolysis product and bond weakening. If the MO94 is populated a priori by double reduction, this precludes stabilization of the radical homolysis product and, hence, also the bond weakening effect through stabilization by spin density delocalization is disabled. This implies that MO94 and its occupation upon bond homolysis have a preparatory bonding effect on the strengths of the Al–C bond.

## 4 The Preparatory Character of Molecular Orbitals

The above‐described findings state that changing the occupation of frontier molecular orbitals can change how homolysis products are relaxing and, therefore, influence the bonding energy. We asked ourselves if this phenomenon of preparatory effects associated with the population of MOs is an exclusive feature of our particular organometallic system or if it is found in general when looking at covalent bonds surrounded by π systems. In the following, we describe theoretical investigations on changes of C–H bonds in simple organic molecules upon the (de)population of their perpendicular π systems in whose nodal plane the bond of interest is located. As a reference system, we computed diatomic methyne [CH]^•^, which homolyzes to the atoms C and H and thus is incapable of preparation (*no delocalization or structural relaxation possible*, Figure 4A). Population or depopulation of the singly occupied SOMO of [CH]^•^ changes the BDE(C─H) only slightly by up to 13%. Hence, we assume a deviation in the magnitude of up to 15% in BDE and Δ*E*_Int_ as being the upper limit induced by secondary effects related to changed electrostatics or contracted / expanded MOs.

**FIGURE 4:**
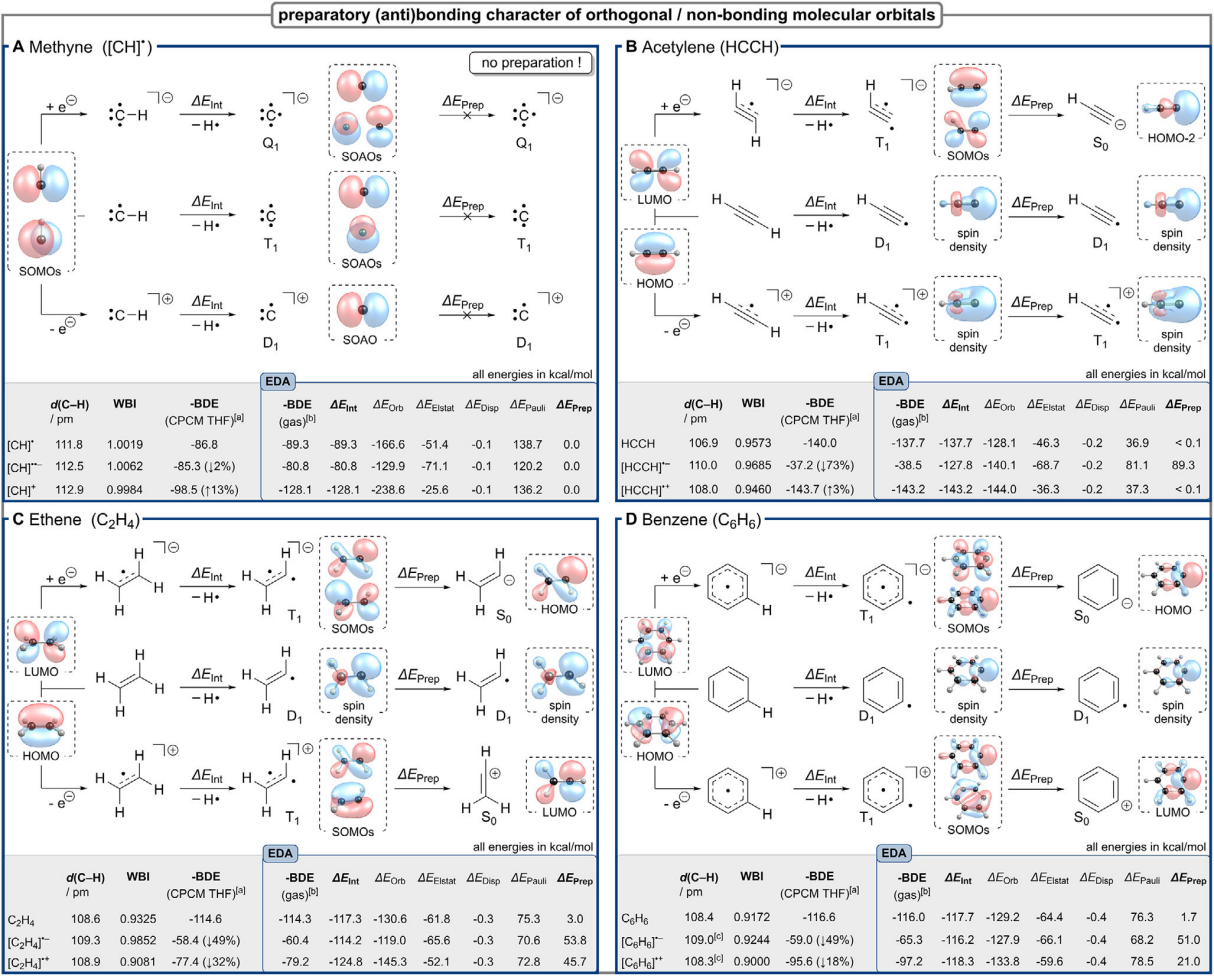
Homolysis path with separation into the intrinsic interaction and preparation of methyne ([CH]^•^, (A), acetylene (HCCH, B), ethene (C_2_H_4_, C) and benzene (C_6_H_6_, D) in the corresponding neutral, oxidized, and reduced states. Computed ‐BDE values of C─H bonds in kcal/mol, Wiberg bond indices (WBIs), atom distances in pm, and values obtained during EDA in kcal/mol are given. All calculations were conducted using geometries obtained at the PBE0‐D3BJ/def2‐TZVP/CPCM(THF) level. Shown molecular orbitals were displayed with isovalues of 0.05 and spin densities with values of 0.005 a.u. [a] ‐BDE obtained at the PBE0‐D3BJ/def2‐TZVPD/CPCM(THF) level of theory. [b] EDA conducted at the PBE0‐D3BJ/TZ2P level. [c] Benzene is of *D*_2h_ symmetry after oxidation or reduction. Values are depicted for the C─H bonds lying within the symmetry axis. Further values are given in the Supporting Information.

For acetylene (HCCH, Figure 4B) no change in C─H BDE upon reduction or oxidation was expected, since HOMO and LUMO are both perpendicular to the C─H bond and do not exhibit any coefficient along this bond. The C─H BDE in acetylene increases by 3% upon oxidation but decreases drastically by 73% upon reduction. The minor influence of oxidation can be easily predicted by using Lewis structures. The fragmentation of the C─H bond in acetylene yields the hydrogen atom and a fragment, which is best described as [HCC_frag_]^•^ radical, the Δ*E*_Prep_ of which is negligible. This means that the unpaired electron, which forms the C─H bond, is not delocalized within the {HCC} framework after homolysis. Within the oxidized system, this situation does not change. When we break the C─H bond in the doublet species [HCCH]^•+^, the fragment has one unpaired electron in the π system and one at the former position of the C─H bond, which is electronically expressed as a triplet state. The relaxed [HCC]^+^ cation has a triplet ground state, which is why the [HCC_frag_]^•+^ is barely stabilized and again shows almost no preparation wherefore the BDE remains almost unchanged. Within the anionic system [HCCH]^•–^, which is of *C*_2h_ symmetry, C─H bond is fragmented into a hydrogen atom and a *vertical* species [HCC_frag_]^••–^ with one electron in the π system and one electron in the σ plane. This fragment relaxes geometrically to a linear adiabatic molecular ion and electronically to a singlet configuration (S_0_), which is the electronic ground state of the acetylide anion. This homolysis product is well known to be very stable, because the negative charge is located in a *sp*‐hybrid orbital with large *s*‐character. The values for Δ*E*_Int_ are only slightly deviating for the neutral, cationic, and anionic system, but a strong Δ*E*_Prep_ of 89.9 kcal/mol for the acetylene anion is leading to a significantly weaker C─H bond after population of the LUMO of acetylene, which does not show an orbital coefficient at the perpendicular C─H bond, but is of preparatory antibonding character.

The same argumentation holds true for a weakening of the C─H bond in ethene (H_2_C = CH_2_) by 49% (C_2_H_4_, Figure 4C) upon reduction. As *sp* ^2^‐hybridized carbanions are qualitatively spoken less stabilized than *sp* carbanions, it seems intuitive that Δ*E*_Prep_ is with 54.1 kcal/mol also less pronounced compared to the acetylene system. For ethene, the oxidation likewise enables preparation of the fragment after C─H cleavage as alkenyl cations have singlet ground states leading to a Δ*E*_Prep_ of 46.0 kcal/mol and a drop in BDE of 32% compared to ethene. The same phenomenon is observed in aromatic systems. The fragmentation of the C─H bond in benzene is showing almost no preparation (C_6_H_6_, Figure 4D). Yet, the oxidation of benzene to the radical cation breaks down aromaticity. If the C─H bond is cleaved to a fragment with one unpaired electron in the π and one in the σ system, the driving force for rearomatization gives rise to a considerable preparation energy of 21.0 kcal/mol to form the phenyl cation, resulting in a drop of BDE by 18%. The corresponding mechanism for the anion is even more pronounced, as the diradicaloid fragment [C_6_H_5frag_]^••–^ rearomatizes to the rather stable phenyl anion as homolysis product. The anionic charge is more stable within the *sp* ^2^‐hybrid orbital, making the phenyl anion a more stabilized species than the phenyl cation. This agrees with the synthetic accessibility of a manifold of organometallic phenyl anion equivalents, like Ph–Li etc., but the absence of true phenyl cation equivalents. Hence, the homolysis of the C─H bond in the benzene radical anion is connected to 47.6 kcal/mol of preparation energy and a BDE, which is 49% weaker than in benzene. This means that the π orbitals of benzene have preparatory antibonding character on the C─H bonds laying in their nodal planes, because the stability of the homolysis products relatively to the starting compounds is increased.

In all mentioned examples, the bond lengths, the WBIs, and individual EDA terms as Δ*E*_Int_, Δ*E*_Orb_, Δ*E*_Elstat_, Δ*E*_Disp_, or Δ*E*_Pauli_ of the C–H bonds did only change insignificantly upon the (de)population of the π orbitals. The Δ*E*_Prep_, that is the energy difference between *vertical* and *adiabatic* states, is the dominating term, correlated with the change in BDE. These examples illustrate how the potential presence of preparatory bonding (NB)MOs can qualitatively be predicted by Lewis structures. The molecule of interest must be drawn in its neutral, cationic, and reduced form, following conventional molecular chemistry intuition. Then the bond of interest is replaced by unpaired electrons, one at each fragment without changing the remaining molecule. If these fragments already look ‘satisfied’ in a molecular chemistry manner (e.g., [C_6_H_5frag_]^•^) the preparation to the actual homolysis product might be insignificant. But, if the fragment looks ‘wrong’ or ‘unstable’ (e.g., the bent [HCC_frag_]^••–^ as π,σ diradicaloid), there might be a better geometric and electronic valence bond description of this species, which is equivalent to a pronounced preparation in the EDA picture and gives the first clear hint for the preparatory character of the (de)populated frontier MOs.

Preparatory character is not an exclusive property of non‐bonding MOs, but we rather postulate that in many cases preparatory and the ‘real’ (anti)bonding character coexist. The BDE of the C─H bond in methane is, for instance, reduced by 55 and 54% upon reduction or oxidation, respectively. Most chemists would see this as obvious because the FMOs are of clear bonding (HOMO) and antibonding (LUMO) character (Figure 5A). But the intrinsic interaction between C and H atom in methane and its reduced/oxidized derivative is not really changing. Instead, a large preparation energy due to electronic relaxation is observed, because the diradicaloid fragments [CH_3frag_]^••–^ and [CH_3frag_]^••+^ relax to the well‐known closed‐shell methanide anion and methylium cation. The α‐CH bond in diethyl ether becomes weaker upon oxidation, which implies depopulation of the bonding HOMO, a lone pair orbital of the oxygen atom hyperconjugated with the α‐CH bonds (Figure 5B). Although the intrinsic interaction between C and H atoms even slightly increases due to contracted orbitals and better orbital overlap, this increase is outweighed by a pronounced preparation energy. This preparation can be expressed as relaxation of a diradicaloid fragment with spin density on the oxygen atom and at the bond‐forming carbon atom to the closed‐shell oxonium ion as very stable homolysis product. For many further molecules the change in BDE upon oxidation/reduction can likewise be explained by large preparation energies or stabilization of the homolysis products rather than by changes in the intrinsic interaction energy. Impressive examples are the oxidation of formaldehyde (Figure 5C), which even doubles the interaction energy and still decreases the BDE of the C─H bond, the weakening of the C–C bond in toluene (Figure 5D), or the C─F bond in fluorobenzene (Figure 5F) by the same mechanism as previously described for benzene. Moreover, the BDE(N–H) in pyrrole is reduced by 94% upon reduction, whereas Δ*E*_Int_ remains mostly unaltered (Figure 5E). The same mechanism as in methane is observed for the reduction of water (Figure 5G), ammonia (Figure 5H), silane (Figure 5I), or sulfur hexafluoride (Figure 5J) as inorganic examples. For the well‐known chromium carbonyl arene complex Cr(C_6_H_6_)(CO)_3_ (Figure 5K), electronic preparation, thus BDE alteration, is enabled by metal‐organic cooperativity. The LUMO of Cr(C_6_H_6_)(CO)_3_ is delocalized over the metal and the arene ligand. Reduction leads to a 19 valence electron (VE) complex, which could be described as Cr(‐I)‐d^7^ complex with neutral C_6_H_6_ ligand or as Cr(0)‐d^6^ complex with anionic [C_6_H_6_]^•–^ ligand. Anyhow, the fragmentation of the C─H bond in the C_6_H_6_ scaffold yields a diradicaloid fragment with spin density in the σ plane of the arene ligand and at the chromium atom. This fragment relaxes to a stable closed‐shell 18 VE electron complex with a phenyl anion as π ligand. Here the high stability of 18 VE complexes is connected to a Δ*E*_Prep_ of 52.0 kcal/mol and, consequently, to a considerable drop in ‐BDE from 116.7 to 64.1 kcal/mol.

**FIGURE 5:**
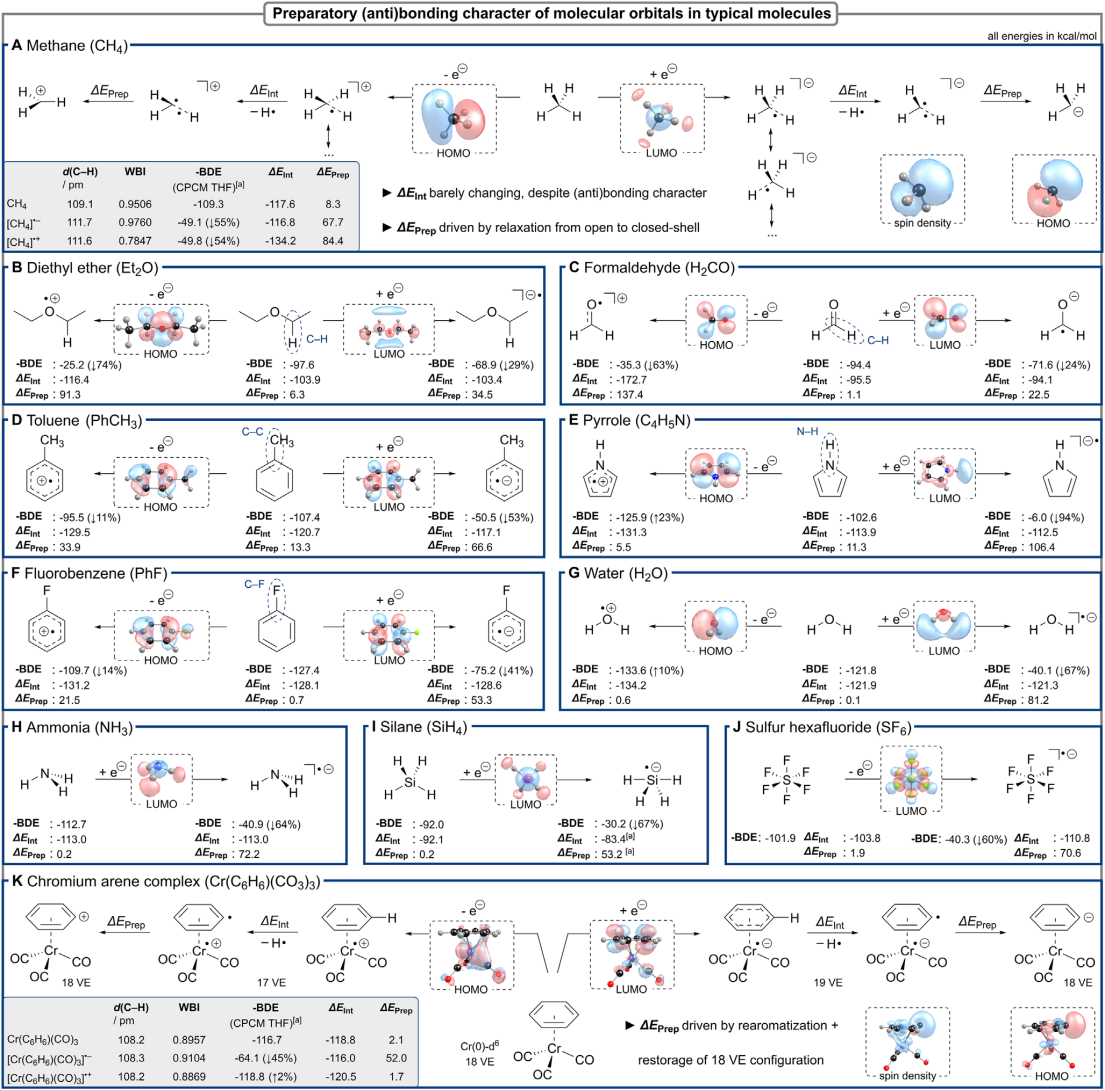
(A) Homolysis path with separation in the intrinsic interaction and preparation of reduced and oxidized methane. Computed ‐BDEs, Δ*E*_Int_, and Δ*E*_Prep_ of bonds in kcal/mol, Wiberg bond indices (WBIs), and atom distances in pm are given. (B–J) ‐BDEs, Δ*E*_Int_, and Δ*E*_Prep_ of neutral, reduced, and oxidized diethyl ether, formaldehyde, toluene, pyrrole, fluorobenzene, and water as well as for neutral and reduced ammonia, silane, and sulfur hexafluoride to show the concept of preparatory bonding orbitals for varied organic and inorganic examples. (K) Homolysis path with separation in the intrinsic interaction and preparation of reduced and oxidized Cr(PhH)(CO)_3_. Computed ‐BDEs, Δ*E*_Int_, and Δ*E*_Prep_ of C─H bonds in kcal/mol, Wiberg bond indices (WBIs), and atom distances in pm are given. All values were obtained at the PBE0‐D3BJ/def2‐TZVPD/CPCM(THF)//PBE0‐D3BJ/def2‐TZVP/CPCM(THF) level.

## 5 Conclusion

This work presents the preparatory character of molecular orbitals as a valuable concept for predicting trends in BDEs upon oxidation/reduction of molecular compounds. The (de)population of MOs with preparatory character changes how the molecular fragments relax to the actual homolysis products or, thought reversely how both bond‐forming radical fragments must be prepared to form the bond. In other words, homolysis products can get thermodynamically stabilized or destabilized by (de)populating FMOs, and this intrinsically influences the strength of chemical bonds. Aluminum organometallics are shown as first isolated compounds highlighting that orbital coefficients of populated MOs at the bond of interest are no prerequisite for preparatory effects on chemical bonds. Even the (de)population of MOs, that are non‐bonding according to the qualitative MO theory picture, influences BDEs of bonds that even lay within the nodal plane of the given MO, whereas the intrinsic interaction between the bond‐forming atom indeed stays mostly unchanged. For MOs, that actually are bonding or antibonding, preparation can be seen as coexistent or an alternative explanation why chemical bonds are getting weaker or stronger upon the population or depopulation of these orbitals.

It is noted that the basics of EDA theory are hotly debated and there is no physical justification for the complete decoupling of intrinsic interaction and preparation. Consequently, we do not want to point out the exact numbers for Δ*E*_Int_ and Δ*E*_Prep_, which in some cases might be quite different if the electronic states of the fragments are defined differently. But rather an understanding of the general concept of preparatory character of molecular orbitals is presented, which is observed for a variety of molecules in organic and inorganic chemistry. The presented concept is simple and predictable by simple Lewis structures using pen and paper. It is expected that the preparatory character of orbitals has been overlooked until now, because it is unintuitive when only looking at orbital coefficients and often only the interaction energy is associated with bonding strength, thus opposing our definition of BDEs. We are confident that preparatory (anti)bonding MOs will get increased attention within research fields like photoredox catalysis, electro‐catalysis, PCET, or synthetic electrochemistry as well as within teaching of theoretical organic and organometallic chemistry.

## Author Contributions

JOW: Investigation (lead, synthesis, spectroscopy, quantum chemistry), Data Curation (lead), Visualization (lead), Validation (lead), Conceptualization (lead), Project Administration (equal), Writing – Original Draft Preparation (lead). JW: Investigation (equal, UV/Vis and vibrational spectroscopy, quantum chemistry), Writing – Review & Editing (equal). PW: Investigation (equal, synthesis, SC‐XRD), Writing – Review & Editing (equal). JP: Writing – Review & Editing (equal). IK: Writing – Review & Editing (equal). DF: Investigation (equal, SC‐XRD). RK: Investigation (equal, vibrational spectroscopy), Writing – Review & Editing (equal). AR: Investigation (equal, pXRD). CF: Investigation (equal, pXRD). IF: Investigation (equal, quantum chemistry), Conceptualization (equal), Project Administration (equal), Resources (equal), Supervision (lead), Writing – Review & Editing (lead). FB: Conceptualization (equal), Project Administration (lead), Resources (lead), Supervision (lead), Writing – Review & Editing (lead)

## Funding

J.W. and P.W. thank the Verband der Chemischen Industrie (VCI) for financial support of their PhD by Kekulé scholarships. This work was partly carried out with the support of the Karlsruhe Nano Micro Facility (KNMF), a Helmholtz Research Infrastructure at Karlsruhe Institute of Technology (KIT). Support by the state of Baden‐Württemberg through bwHPC and the German Research Foundation (DFG) through grants No. INST40/575‐1 FUGG (JUSTUS 2 Cluster) is acknowledged. Support from the Spanish MICIU/AEI/10.13039/501100011033 (PID2022‐139318NB‐I00 and RED2022‐134287‐T) is acknowledged.

## Conflicts of Interest

The authors declare no conflict of interest.

## Supporting information




**Supporting File**: advs76530‐sup‐0001‐SuppMat1.pdf.

## Data Availability

All data supporting this study are given in the Supporting Information. All raw data were deposited at Zenodo: https://doi.org/10.5281/zenodo.17827259. Crystallographic data were deposited at the Cambridge Crystallographic Data Centre (CCDC). Deposition numbers are **3** (2513348), **4** (2513358), **5** (2513359), **7** (2513360), **8** (2513361), **10** (2513362).

## ENDNOTE

1 Within this work, canonical molecular orbitals are discussed.
