# Supplementary material for: The Preparatory (Anti)Bonding Character of Molecular Orbitals
Source: Adv Sci (Weinh). 2026 Jul 13:e76530. Online ahead of print. doi: 10.1002/advs.76530 (PMC13359395; doi:10.1002/advs.76530)
Supplement: Supplementary file 1 — Supporting File: advs76530‐sup‐0001‐SuppMat1.pdf. [file ADVS-9999-e76530-s001.pdf]

The preparatory (anti)bonding character of molecular orbitals

Authors: Jonas O. Wenzel[1,2]*, Johannes Werner[1,3], Pascal Weisenburger[1], Joachim Podlech[4], Ralf Köppe[1], Ingo Krossing[5], Dieter Fenske[6], Andreas Reiß[1], Claus Feldmann[1], Israel Fernández[2]* and Frank Breher[1]*

Affiliations:

[1] Karlsruhe Institute of Technology (KIT), Institute for Inorganic Chemistry (AOC); Engesserstraße 15, 76131 Karlsruhe, Germany

[2] Departamento de Química Orgánica, Facultad de Ciencias Químicas Universidad Complutense de Madrid; 28040 Madrid, Spain

[3] Karlsruhe Institute of Technology (KIT), Institute of Physical Chemistry (IPC); Fritz-Haber-Weg 2, 76131 Karlsruhe, Germany

[4] Karlsruhe Institute of Technology (KIT), Institute of Organic Chemistry (IOC); Fritz-Haber-Weg 6, 76131 Karlsruhe, Germany

[5] University of Freiburg, Institute for Inorganic and Analytical Chemistry (IAAC); Alberstraße 21, 79104 Freiburg, Germany

[6] Karlsruhe Institute of Technology (KIT), Institute of Nanotechnology (INT); Kaiserstraße 12, 76131 Karlsruhe, Germany

Supporting Information

1. Materials and methods 4
1.1. Techniques 4
1.2. Chemicals and solvents 4
1.3. NMR spectroscopy 4
1.4. EPR spectroscopy 5
1.5. FT-IR spectroscopy 5
1.6. UV/Vis spectroscopy 5
1.7. Single crystal X-ray diffractometry 5
1.8 Powder X-ray diffraction (pXRD) 6
1.9. Cyclovoltammetry 6
1.10. Elemental analysis (CHN) 6
1.11. Quantum chemical calculations 7
2. Synthetic procedures 8
2.1. Synthesis and characterization of reduced derivatives of BPI–$AlMe_2$ (1) 8
2.2. The observation of disproportionation products 17
3. NMR spectra 19
3.1 NMR characterization of 6 19
4. EPR spectroscopy 24
4.1 EPR spectroscopy on $[1]^{\bullet-}$ 24
4.2 EPR spectroscopy on $[1]^{2-}$ 28
5. IR spectra 31
6. UV/Vis spectra 34
7. SC-XRD 35
8. pXRD 48
9. Computational data 52
9.1 Geometry optimization and vibrational frequencies 52
9.2 Molecular orbitals, spin densities, population analysis and natural bond orbital analysis 288
9.3 Bond dissociation energies (BDEs) and bond orders 293

9.4 Energy decomposition analysis (EDA).. 300

9.5 EPR parameters of $[1]^{•-}$ 308

9.6 EDA on molybdenum ammonia complex.. 309

10. References 310

1. Materials and methods

1.1. Techniques

If not stated otherwise all manipulations were carried out under argon atmosphere within MBraun gloveboxes (MB200 G and Unilab) or using conventional Schlenk-techniques. Used argon was purchased from Air liquid with a purity grade of 99.999 % and used without further drying. Solid chemicals were transferred with spatulas and weighed in with an accuracy of ±1%. If the purity of compounds was below 98 wt% the used amount was corrected by the impurity factor. Liquid chemicals were transferred by single-use Injekt® syringes by B. Braun. Filtrations were conducted by using syringe filters within the glovebox.

1.2. Chemicals and solvents

If not stated otherwise all used chemicals were purchased from commercials used without further purification. Solvents were dried over Na/benzophenone or K/benzophenone, degassed by refluxing and purified by distillation. THF-d_8 was dried over Na/K alloy with benzophenone. C_6D_6 was dried over K with benzophenone. Deuterated solvents were degassed by iterative freeze-pump-thaw cycles and purified by trap-to-trap condensation prior to use. BPI–H was synthesized according to the literature procedure from *o*-phthalonitrile and 2-amino pyridine.[1] BPI–$AlMe_2$ was synthesized according to the literature procedure from BPI–H and $AlMe_3$.[2]

1.3. NMR spectroscopy

NMR spectra were measured on a Bruker Avance Neo 400 or an Avance 300 spectrometer. ^1H and ^{13}C chemical shifts are referenced to the used solvent referred to TMS.[3] Coupling constants *J* are given in Hertz as positive values regardless of their real individual signs. The multiplicity of the signals is indicated as s, d, t, q, dec or m for singlets, doublets, triplets, quartets, decets or multiplets, respectively. The abbreviation br. is given for broad signals. If not stated otherwise, NMR samples were prepared in oven-dried 5-mm NMR tubes and sealed under argon. Unless otherwise stated, standard Bruker software routines (TOPSPIN and XWINNMR) were used for the 1D and 2D NMR measurements and the MestreNova software package was used for analyzing the spectra. All given yields and amounts of impurities determined by NMR spectroscopy are considered with an estimated error of ±5%.

1.4. EPR spectroscopy

Continuous wave EPR spectroscopy (CW-EPR) was performed at X-band on a Bruker EMX or Bruker EMXplus spectrometer. The field was calibrated by using 2,2-diphenyl-1-picrylhydrazine (DDPH) with a *g* value of 2.0036.[4] CW EPR simulations were carried out by using the EasySpin toolbox for MatLab.

1.5. FT-IR spectroscopy

IR-spectra were measured using the ATR technique (attenuated total reflection) on a Bruker Vertex 70 spectrometer in the range from 4000 cm^{-1} to 400 cm^{-1}. The intensity of the absorption band is indicated as vw (very weak), w (weak), m (medium), s (strong), vs (very strong), and br (broad). For interactive baseline correction, straight lines were adjusted to the experimental IR spectrum, which were afterwards subjected to a rubber band correction.

1.6. UV/Vis spectroscopy

Stationary spectra were recorded with a CaryWin5000 (Varian) or a Mettler Toledo BenchTop spectrophotometer in a cuvette (Starna, Suprasil300) under argon atmosphere and sealed with J-Young cock with a path length of 1 cm or 1 mm, respectively. The spectra shown are corrected for cuvette and solvent effects.

1.7. Single crystal X-ray diffractometry

Diffraction data were measured using a Stoe IPDS II diffractometer and graphite-monochromated MoK_{α} (0.71073 Å) radiation or Stoe STADIVARI diffractometer and GaK_{α} (1.34134 Å) radiation. Absorption corrections were carried out using the STOE LANA software package.[5] Structure solutions were carried out using OLEX2 1.5[6] by dual-space direct methods with SHELXT,[7] by full-matrix least-squares refinement using SHELXL-2014/7.[8] All non-hydrogen atoms were refined anisotropically. The contribution of the hydrogen atoms, in their calculated positions, was included in the refinement using a riding model. A full listing of atomic coordinates, bond lengths, angles and displacement parameters for all the structures have been deposited at the Cambridge Crystallographic Data Centre. For the individual numbers, please refer to the XRD tables.

1.8 Powder X-ray diffraction (pXRD)

Diffraction measurements were performed on a Stoe Stadi-MP diffractometer (Stoe, Germany) operating with Ge-monochromatized Cu-Kα-radiation (λ = 1.54178 Å). For sample preparation, the compounds **2, 3, 4, 5, 6** and **8** were filled into glass capillaries (0.5 mm in diameter, Hilgenberg, Germany) with argon. Rietveld refinements were performed with the program TOPAS-Academic (version 5), using the *cif*-data from single-crystal structure analysis to potentially confirm the composition, symmetry, and purity of the respective title compound. The refinements were carried out using an axial model.

1.9. Cyclovoltammetry

Cyclic voltammetry measurements were performed with a Autolab potentiostat by Metrohm (AUT40259) and an electrochemical cell within an argon filled glovebox. A freshly polished Pt disk working electrode by Metrohm, a Pt wire as counter electrode, and an Ag wire as (pseudo) reference electrode was used. As electrolyte {[nBu_4N][$Al\{OC(CF_3)_3\}_4$]} (0.01 M in THF) was used. Potentials were calibrated against the Fc/Fc^+ couple by internal standardization with ferrocene or decamethylferrocene. For decamethylferrocene in THF, a redox potential of -225 mV vs. the Fc/Fc^+ couple was considered.[9]

1.10. Elemental analysis (CHN)

Elemental analyses of C, H and N were obtained with a Vario Micro Cube (Elementar Analysensysteme GmbH) in the institutional laboratories of the Karlsruhe Institute of Technology (KIT). Elemental analysis data were gathered from isolated crystalline material of the corresponding compounds and reported as obtained even if the established deviation of ±0.4% was not reached as these requirements were shown to be misleading in some cases.[10]

1.11. Quantum chemical calculations

All computational studies in this work were carried out on the Justus2 bwhpc cluster of the State Baden-Württemberg, Germany or the MINERVA cluster. Quantum chemical computations were conducted using the Orca 6.0.1 software package.[11,12] Natural Bonding Orbital (NBO) analysis was conducted using NBO 6.18.[13] Quantum Theory in Atoms in Moleculs (QTAIM) analysis was done using AIMALL 19.10.12.[14] Local force constants were obtained by using LModeA-Nano[15] based on hessian files, obtained by Orca calculations. We are aware of the difference of force constant values obtained from Wilson's method[16] and the local mode[17] approach,[18] but with regard to diagonal force constants these differences are usually within the uncertainty of experimentally determined values. Methodologial details about certain computations are given in the section 9. Computational data.

2. Synthetic procedures

2.1. Synthesis and characterization of reduced derivatives of BPI–AlMe$_2$ (1) [K(thf)$_x$][BPI–AlMe$_2$] (2)

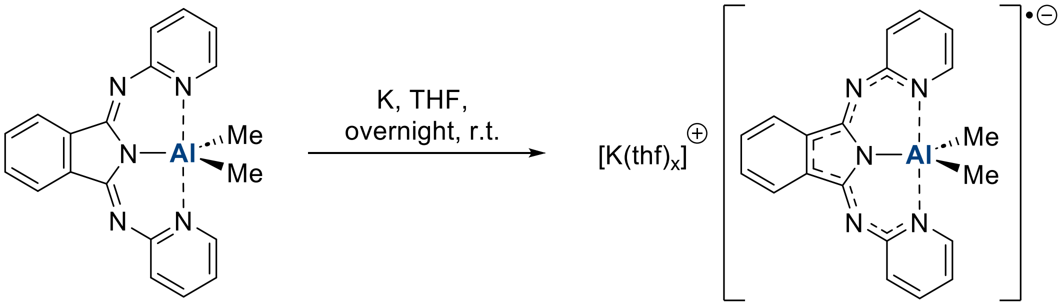

1	2	
$C_{20}H_{18}AlN_5$	$C_{20}H_{18}AlKN_5$	+ x C_4H_8O
355.38 g/mol	394,48 g/mol	+ x 72.11 g/mol

200 mg of **1** (563 µmol, 1.00 equiv.) and 22.0 mg potassium (563 µmol, 1.00 equiv.) were suspended in 10.0 mL THF and stirred at ambient temperature overnight. The solvent was removed under reduced pressure and the residue was dried under high vacuum (~10^{-3} mbar) for several hours. The product was obtained as black solid (312 mg, x = 2: 579 µmol, 103%, x =3: 511 µmol, 91%).

***2** is NMR silent, but the amount of incorporated THF was approximately determined by ^{1}H NMR spectroscopy with internal standardization to a value of x = 2–3 for several batches, depending on the time of drying. It is not recommended to dry the compound under warming/heating as in our hands this was often accompanied with undefined signals in ^{1}H NMR measurements, which we interpreted as decomposition.*

EPR (X-band): g = 2.0035.

FT-IR: 2868 (w), 1581 (s), 1533 (s), 1453 (vs), 1420 (vs), 1293 (m), 1273 (s), 1189 (s), 1147 (s), 1084 (m), 1057 (vs), 994 (s), 855 (m), 758 (s), 713 (s), 694 (s), 638 (vs), 570 (s), 520 (s), 440 (m), 396 (m), 383 (m).

Elemental analysis:

$C_{20}H_{18}N_5AlK$: calc. C 60.90, H 4.599, N 17.75; exp. C 58.01, H 6.738, N 11.67;

$C_{20}H_{18}N_5AlK$ • 3 THF: calc. C 62.93, H 6.931, N 11.47.; exp. C 58.01, H 6.738, N 11.67.

*The deviation between experimental and calculated CHN values are interpreted as consequence of the severe air-sensitivity of **2**, insufficient combustion behavior of*

organometallic compounds, unknown amount of remaining THF and the intrinsic inaccuracies of elemental analysis measurements.

[K(18-crown-6)][BPI–AlMe$_2$] (3)

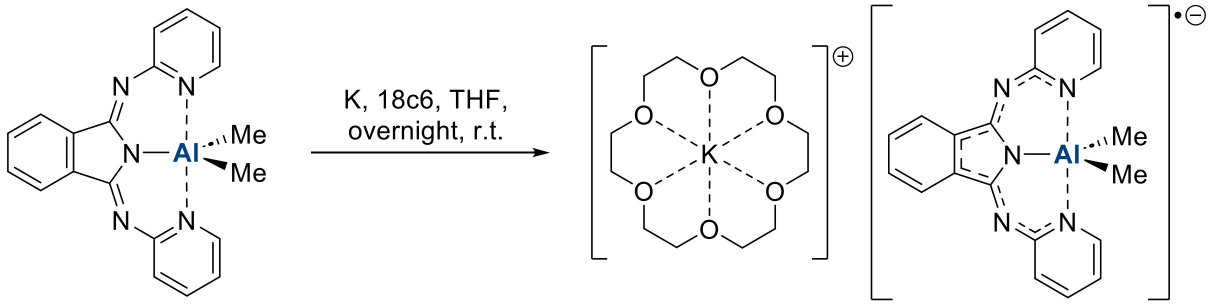

1
$C_{20}H_{18}AlN_5$
355.38 g/mol

3
$C_{32}H_{42}AlKN_5O_6$
658.80 g/mol

237 mg of **1** (667 µmol, 1,00 equiv.), 26.1 mg potassium (667 µmol, 1,00 equiv.) and 176 mg 18-crown-6 (667 µmol, 1,00 equiv.) were suspended in 10.0 mL THF and stirred at ambient temperature overnight. The solvent was removed under reduced pressure and the residue was dried under high vacuum (~10^{-3} mbar) for several hours. The product was obtained as black solid (360 mg, 547 µmol, 82%).

***3** is NMR silent.*

EPR: 2.0035

FT-IR: 2883 (w), 1641 (w), 1604 (w), 1581 (s), 1536 (m), 1450 (s), 1419 (s), 1350 (m), 1315 (w), 1294 (m), 1273 (m), 1248 (m), 1188 (m), 1104 (vs), 1058 (vs), 993 (m), 959 (s), 836 (m), 780 (m), 757 (m), 718 (m), 695 (m), 638 (s), 522 (m), 441 (m), 390 (w).

Elemental analysis:

$C_{32}H_{42}N_5AlKO_6$: calc. C 58.34, H 6.426, N 10.63; exp. C 57.95, H 6.085, N 10.58.

[K(2.2.2-cryptand)][BPI–AlMe$_2$] (4)

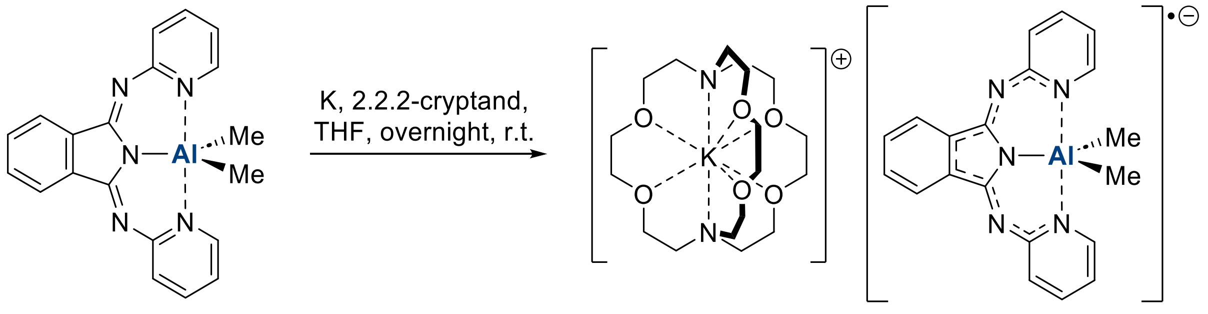

1
$C_{20}H_{18}AlN_5$
355.38 g/mol

4
$C_{38}H_{54}AlKN_7O_6$
770.96 g/mol

191 mg of **1** (537 µmol, 1.00 equiv.) and 21.0 mg potassium (537 µmol, 1,00 equiv.) were suspended in 10.0 mL THF and stirred at ambient temperature overnight. 203 mg 2.2.2-cryptand (537 µmol, 1.00 equiv.) were added and the mixture was stirred for additional two hours, which led to the precipitation of the product. The mother liquor was decanted and the residue was dried under high vacuum (~10^{-3} mbar) for several hours. The product was obtained as black solid (259 mg, 336 µmol, 63%).

***4** is NMR silent.*

EPR: 2.0035

FT-IR: 2962 (w), 2881 (w), 2813 (w), 1582 (w), 1531 (s), 1504 (w), 1450 (vs), 1418 (vs), 1354 (m), 1297 (m), 1260 (m), 1181 (w), 1145 (m), 1131 (m), 1099 (vs), 1082 (vs), 993 (s), 946 (s), 931 (s), 851 (w), 831 (m), 760 (vs), 734 (m), 709 (m), 693 (s), 669 (w), 635 (s), 570 (m), 526 (m), 439 (w).

Elemental analysis:

$C_{38}H_{54}N_7O_6AlK$: calc. C 59.20, H 7.060, N 12.72; exp. C 59,57, H 6,317, N 12,32.

[CoCp*₂][BPI–AlMe₂] (5)

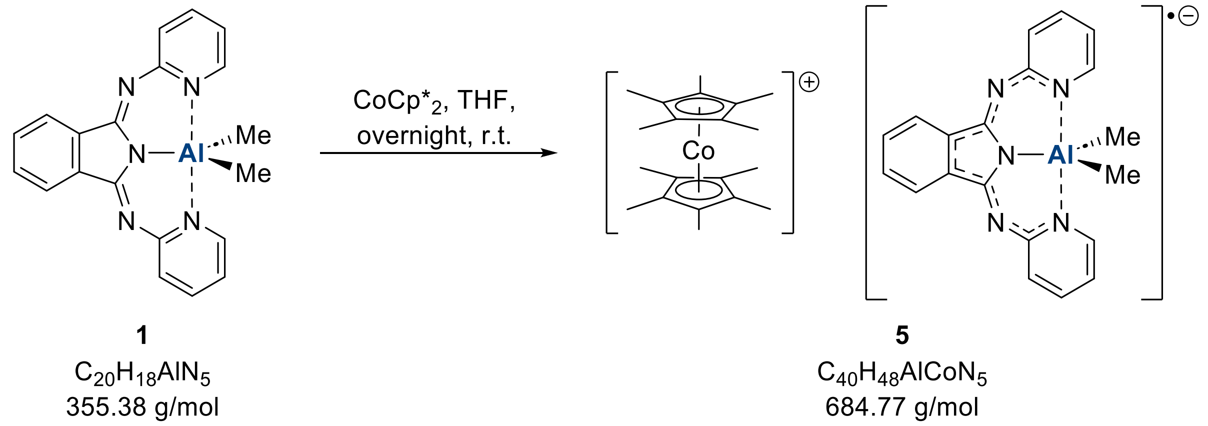

200 mg of **1** (563 µmol, 1.00 equiv.) and 185 mg of bis(pentamethylcyclopentadienide) cobalt(II) (563 µmol, 1.00 equiv.) were dissolved in 10.0 mL THF and stirred at ambient temperature overnight. The solvent was removed under reduced pressure and the residue was dried under high vacuum (~10^{-3} mbar) for several hours. The product was isolated as black solid (300 mg, 438 µmol, 78%).

***5** is NMR silent.*

EPR: 2.0034

FT-IR: 2910 (w), 1581 (m), 1531 (s), 1503 (m), 1452 (vs), 1418 (vs), 1389 (s), 1294 (m), 1271 (m), 1186 (s), 1144 (s), 1110 (m), 1087 (m), 1059 (s), 1019 (m), 992 (s), 907 (m), 850 (m), 753 (s), 709 (m), 695 (s), 669 (s), 638 (vs), 570 (m), 528 (s), 441 (s).

Elemental analysis:

$C_{40}H_{48}N_5AlCo$: calc. C 70.16, H 7.065, N 10.23; exp. C 70,75, H 6,913, N 9,76.

$[K(thf)_x]_2[BPI–AlMe_2]$ (6)

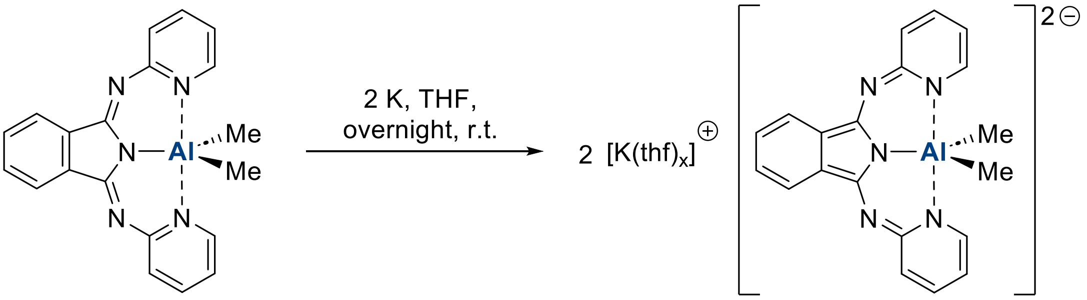

**1**	**6**
$C_{20}H_{18}AlN_5$	$C_{20}H_{18}AlK_2N_5$ + x C_4H_8O
355.38 g/mol	433.58 g/mol + x 72.11

468 mg of **1** (1.32 mmol, 1.00 equiv.) and 103 mg potassium (2.63 mmol, 2.00 equiv.) were suspended in 12.0 mL THF and stirred overnight. The solvent was removed under reduced pressure and the residue was dried under high vacuum (~10^{-3} mbar) for several hours. The product was isolated as black solid (622 mg, x = 2: 1.08 mmol, 82%; x = 3: 957 µmol, 73%).

^{1}H NMR (400 MHz, THF-d_8, ppm): δ = 7.56–7.44 (m, 2H, CH^1), 7.39–7.26 (m, 2H, CH^2), 6.55 (t, J = 6.7, 6.3, 3.4 Hz, 2H, CH^3), 6.36–6.26 (m, 2H, CH^2), 6.11 (d, J = 9.0 Hz, 2H, CH^4), 5.55 (t, J = 6.3 Hz, 2H, CH^5), -0.93 (s, 6H, $CH_3{}^6$).

^{13}C{^{1}H} NMR (101 MHz, THF-d_8, ppm): δ = 153.6 ($C_{quart.}$), 146.6 (CH^1), 131.8 (CH^3), 120.4 (CH^5), 120.3 (CH^2), 116.4 (CH^2), 115.2 ($C_{quart.}$), 101.8 (CH^5), -0.6 ($CH_3{}^6$).

The signal of one quaternary carbon atom was not detected, most probably due to too low signal intensity, because of line broadening effects. For details see section 3.

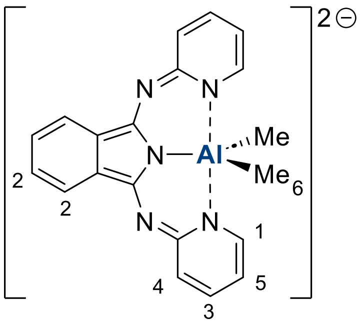

FT-IR: 1606 (w), 1582 (w), 1533 (m), 1470 (m), 1450 (s), 1421 (s), 1371 (m), 1266 (m), 1189 (m), 1145 (m), 1123 (m), 1058 (m), 1022 (m), 985 (m), 865 (m), 846 (m), 745 (vs), 709 (s), 671 (vs), 626 (vs), 568 (s), 526 (vs), 440 (vs), 416 (s), 383 (s).

Elemental analysis:

$C_{20}H_{18}N_5AlK_2$: calc. C 55.40, H 4.184, N 16.15; exp.C 51.27, H 4.878, N 11.92.

$C_{20}H_{18}N_5AlK$ • 3 THF: calc. C 59.14, H 6.514, N 11.47.; exp. C 51.27, H 4.878, N 11.92.

The deviation between experimental and calculated CHN values are interpreted as consequence of the severe air-sensitivity of ***6****, insufficient combustion behavior of organometallic compounds, unknown amount of remaining THF and the intrinsic inaccuracies of elemental analysis measurements.*

[K(2.2.2-cryptand)]$_2$[BPI–AlMe$_2$] (7)

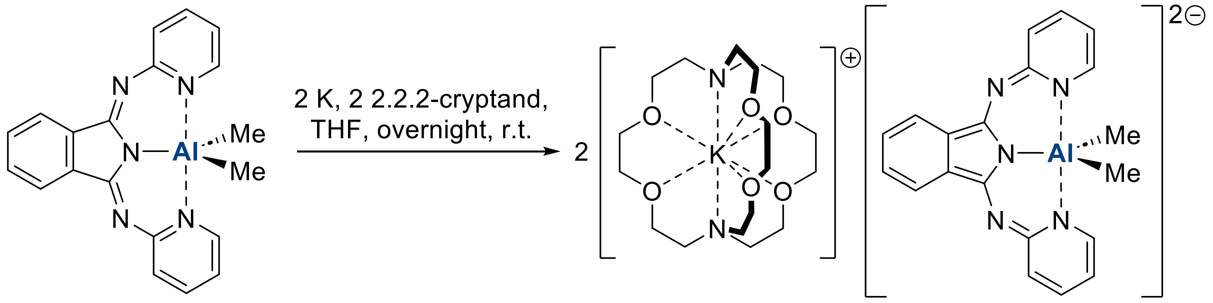

7

$C_{56}H_{90}AlK_2N_9O_{12}$

1186.57 g/mol

105 mg of **1** (296 µmol, 1.00 equiv.) and 44.0 mg potassium (1.125 mmol, 3.81 equiv.) were suspended in 10.0 mL THF and stirred at ambient temperature overnight. To the mixture 232 mg 2.2.2-cryptand (296 µmol, 2.00 equiv.), dissolved in 5.00 mL THF, were added, which afforded precipitation of the product. The mother liquor was decanted and the solid residue dried under high vacuum (~10^{-3} mbar) for several hours. The product was isolated as black solid (264 mg, 222 µmol, 75%).

***7** does not show sufficient solubility in any solvent without decomposition, wherefor we are unable to report on chemical shifts.*

FT-IR: 2872 (m), 2806 (w), 1598 (w), 1575 (w), 1531 (m), 1516 (m), 1452 (s), 1419 (s), 1377 (w), 1353 (s), 1272 (m), 1259 (m), 1169 (w), 1130 (s), 1098 (vs), 1077 (vs), 1022 (m), 993 (m), 972 (m), 945 (vs), 861 (m), 830 (m), 729 (s), 698 (m), 677 (m), 633 (m), 612 (m), 566 (m), 522 (m), 440 (m).

Elemental analysis:

$C_{38}H_{54}N_7O_6AlK$: calc. C 56.96, H 7.645, N 10.62; exp. C 56.52, H 7.503, N 10.52.

[Na(15-crown-5)]$_2$[BPI–AlMe$_2$] (8)

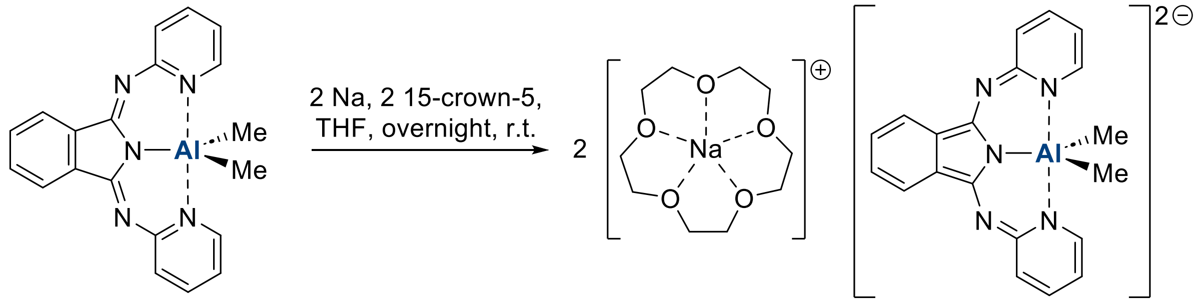

8
$C_{40}H_{58}AlN_5Na_2O_{10}$
841,89 g/mol

200 mg of **1** (563 µmol, 1.00 equiv.) and 35.0 mg sodium (1.52 mmol, 2,71 equiv.) were suspended in 10.0 mL THF and stirred at ambient temperature overnight. 248 mg of 15-crown-5 (1.13 mmol, 2,00 equiv.) were added and the mixture was stirred for additional two hours at ambient temperature. The solvent was removed under reduced pressure and the residue was dried under high vacuum for several hours. The product was obtained as black solid (401 mg, 476 µmol, 85%).

***8** does not show sufficient solubility in any solvent without decomposition, wherefor we are unable to report on chemical shifts.*

FT-IR: 3030 (vw), 2907 (vw), 2869 (vw), 1606 (w), 1571 (vw), 1534 (s), 1522 (m), 1503 (vs), 1484 (m), 1455 (s), 1427 (m), 1378 (w), 1350 (m), 1293 (w), 1274 (s), 1244 (w), 1199 (vw), 1172 (vw), 1145 (m), 1114 (vs), 1088 (vs), 1038 (m), 1026 (m), 994 (w), 981 (w), 944 (s), 862 (m), 852 (w), 822 (w), 737 (vs), 713 (w), 690 (vw), 675 (m), 636 (w), 620 (s), 570 (vw), 552 (vw).

Elemental analysis:

$C_{40}H_{58}N_5AlNa_2O_{10}$: calc. C 58.67, H 7.139, N 8.55; exp. C 55.36, H 6.970, N 8.23.

2.2. The observation of disproportionation products $[K_4(thf)_6][BPI–AlMe_2]_3$ (10)

The reduction of BPI–$AlMe_2$ (**1**) with alkali metals is accompanied by a color change from BPI-typical bright yellow to very dark red upon single reduction and to very dark green upon double reduction. We investigated if precipitation by addition of *n*-hexane to THF solution is a suitable method for purifying compounds like **2**. Interestingly, we noticed that the intense dark red solutions of **2** became slightly yellowish after the addition of *n*-hexane, which already indicated the formation of non-reduced **1**. This was observed even when the *n*-hexane was thoroughly dried and freshly distilled from potassium/benzophenone. We interpret the color change after *n*-hexane addition as partial disproportionation of **2** into **1** and **6** (Figure S1).

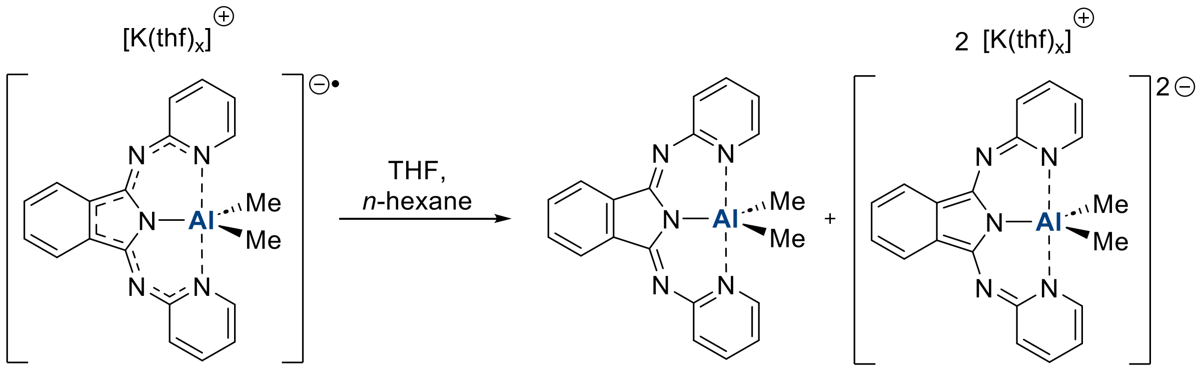

2 **1** **6**

Figure S1. Schematic disproportionation of **2** into **1** and **6**, upon addition of n-hexane into THF solutions of **2**.

Our proposal of disproportionation was supported by isolation of single-crystals from THF/n-hexane solutions of **2**, which revealed the molecular solid-state structure of $[K_4(thf)_6][BPI–AlMe_2]$ (**10**) (Figure S2). Compound **10** represents a coordination polymer, which consists out of one doubly reduced $[BPI–AlMe_2]^{2-}$ and two singly reduced $[BPI–AlMe_2]^{-}$ fragments, which are connected by coordinative bonds to potassium ions, whose coordination sphere is furthermore saturated by THF molecules. The fact, that $[BPI–AlMe_2]^{2-}$ was isolated as cocrystal with the used $[BPI–AlMe_2]^{-}$, hints on the occurrence of disproportionation reaction in solutions. For crown ether and cryptand compounds this phenomenon was not observed, wherefore we follow, that the formation of oligomeric coordination compounds in solution favors electron transfer processes between different BPI–$AlMe_2$ fragments.

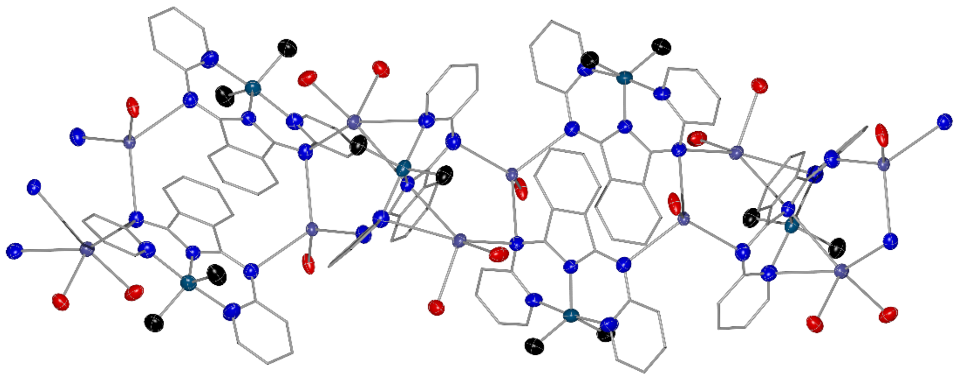

Figure S2. SC-XRD structure of **10**. Hydrogen atoms are omitted for clarity. THF molecules were only depicted by their oxygen atom for clarity. For details concerning bond lengths and angles see section 7.

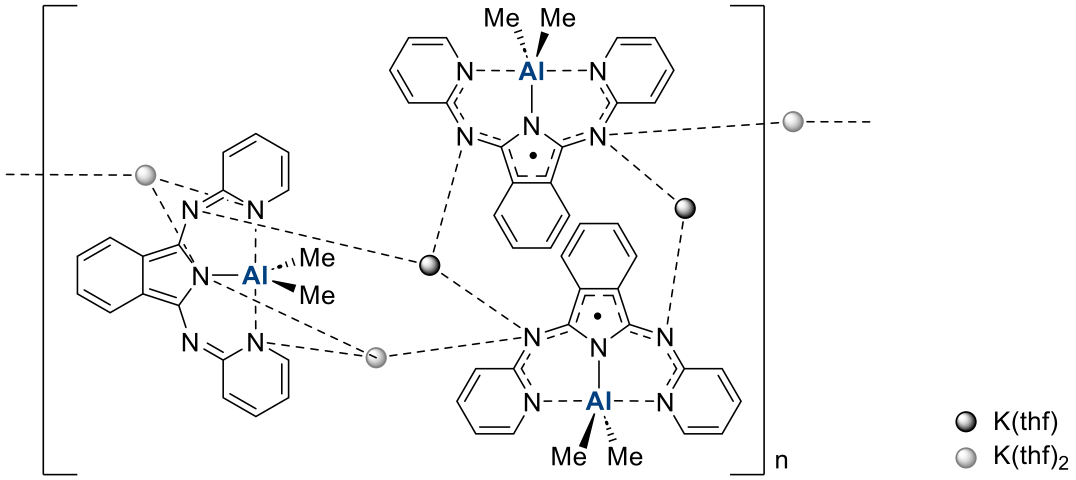

Figure S3. Schematic representation of the solid-state structure of **10** by *Lewis* structure.

3. NMR spectra

3.1 NMR characterization of 6

When ^{1}H NMR measurements were conducted by preparing a sample of isolated compound **6** in THF-d_8 solution, in most cases no signals or only very broad signals in the aromatic region were observed. We interpreted this as potential line broadening due to the presence of paramagnetic [**1**]$^{•-}$ as decomposition product as consequence of the severe air sensitivity of **6**. Therefore, we reduced **1** with an excess of potassium within an NMR tube and flame sealed the tube under potassium atmosphere (Figure S4). The obtained spectra showed sharper NMR signals and are depicted in the following.

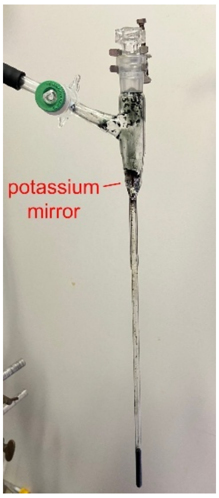

Figure S4. Photograph of the NMR sample preparation of **6** within an NMR tube in the presence of an excess of potassium, which was flame-sealed after quantitative in-situ reduction of **1** to **6**.

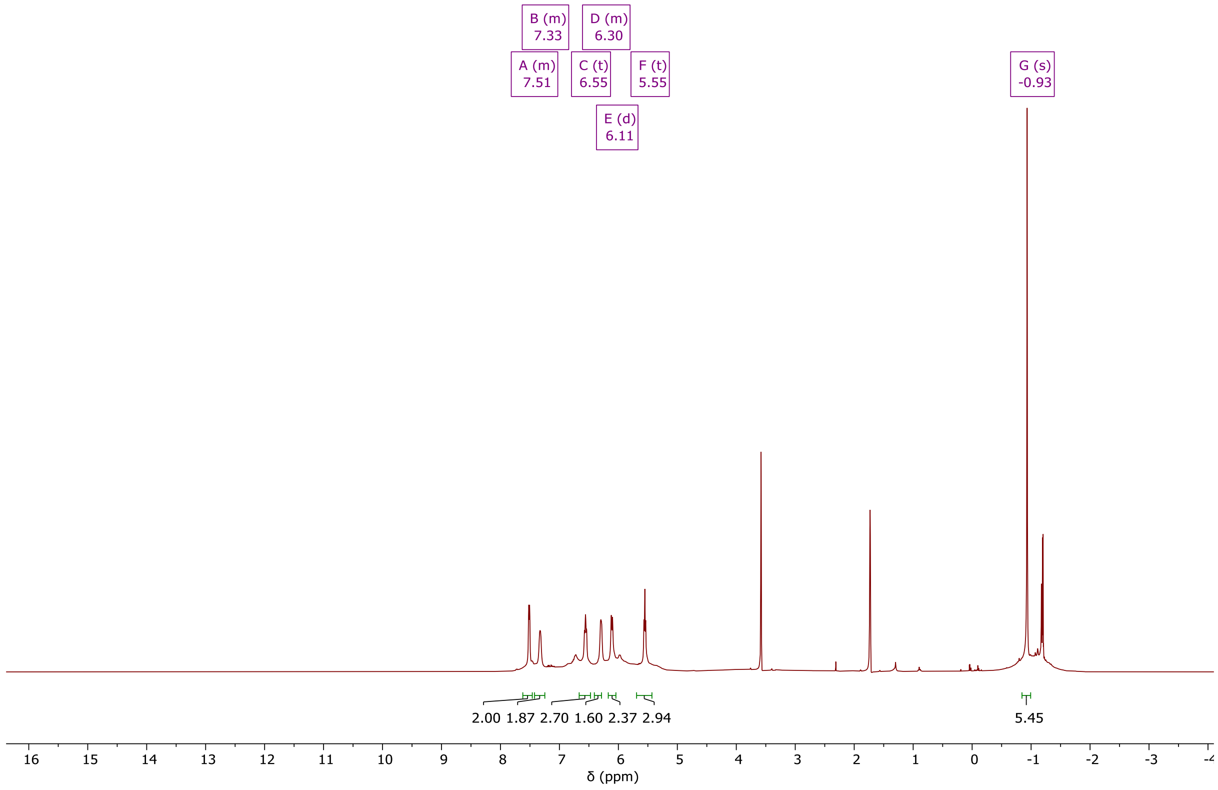

Figure S5. ^{1}H NMR spectrum of **6** in THF-d_8.

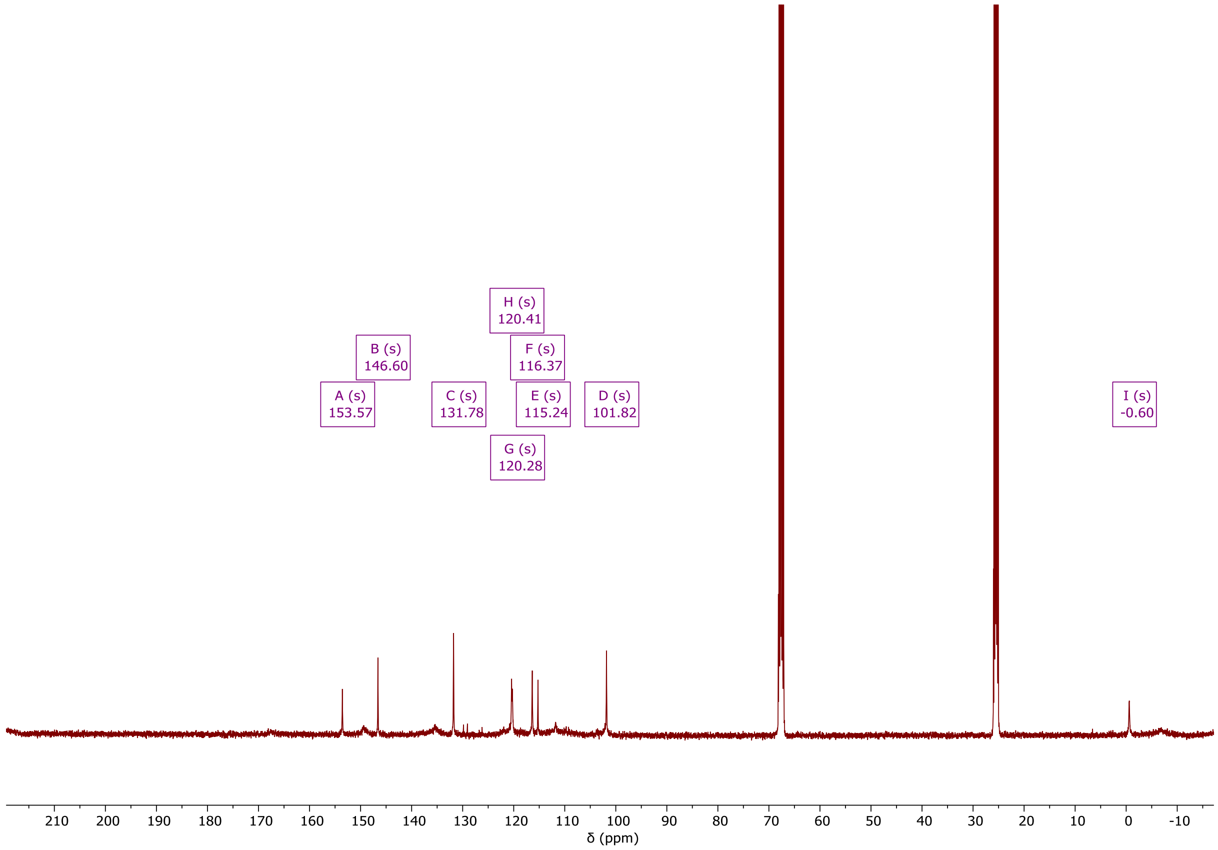

Figure S6. $^{13}C\{^{1}H\}$ NMR spectrum of **6** in THF-d_8.

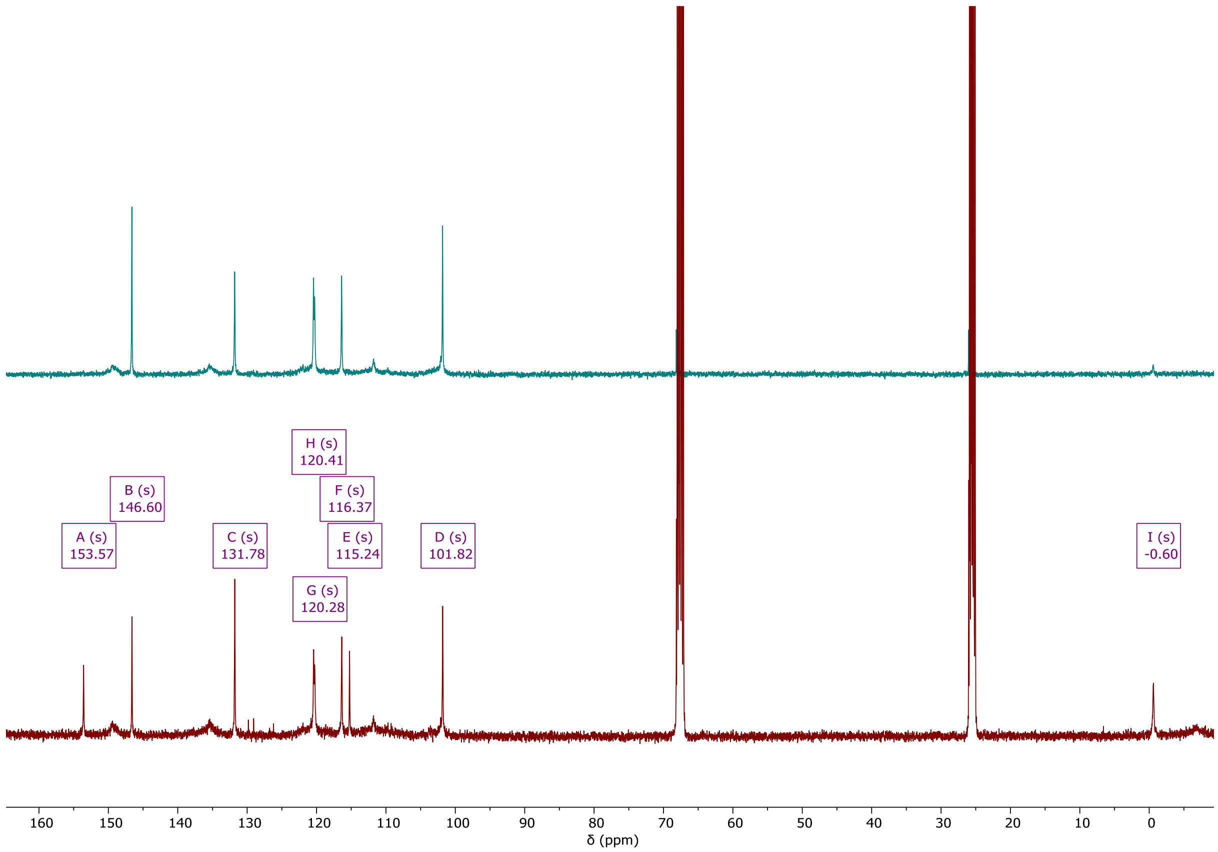

Figure S7. dept135 ^{13}C NMR spectrum (top) stacked with the $^{13}C\{^1H\}$ NMR spectrum (bottom) of **6** in THF-d_8.

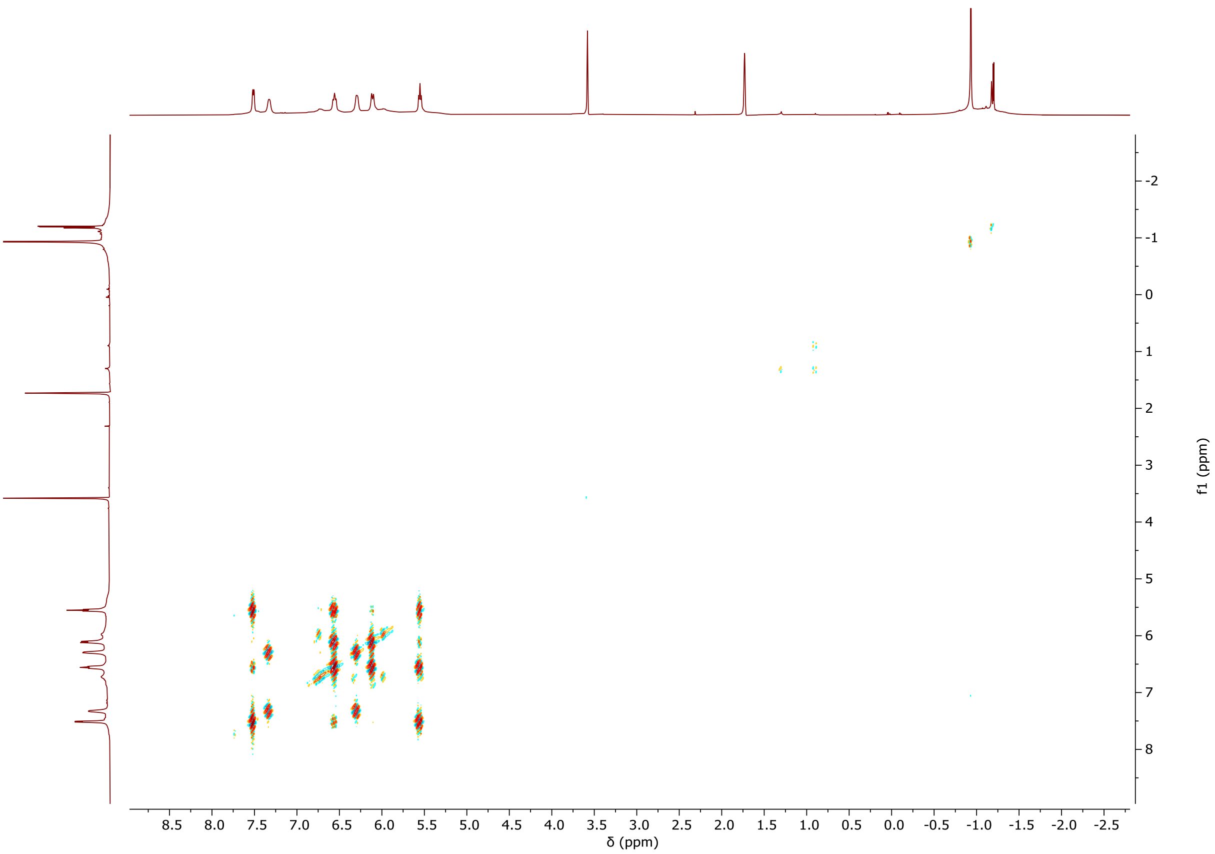

Figure S8. $^{1}H^{1}H$ COSY NMR spectrum of **6** in THF-d_8.

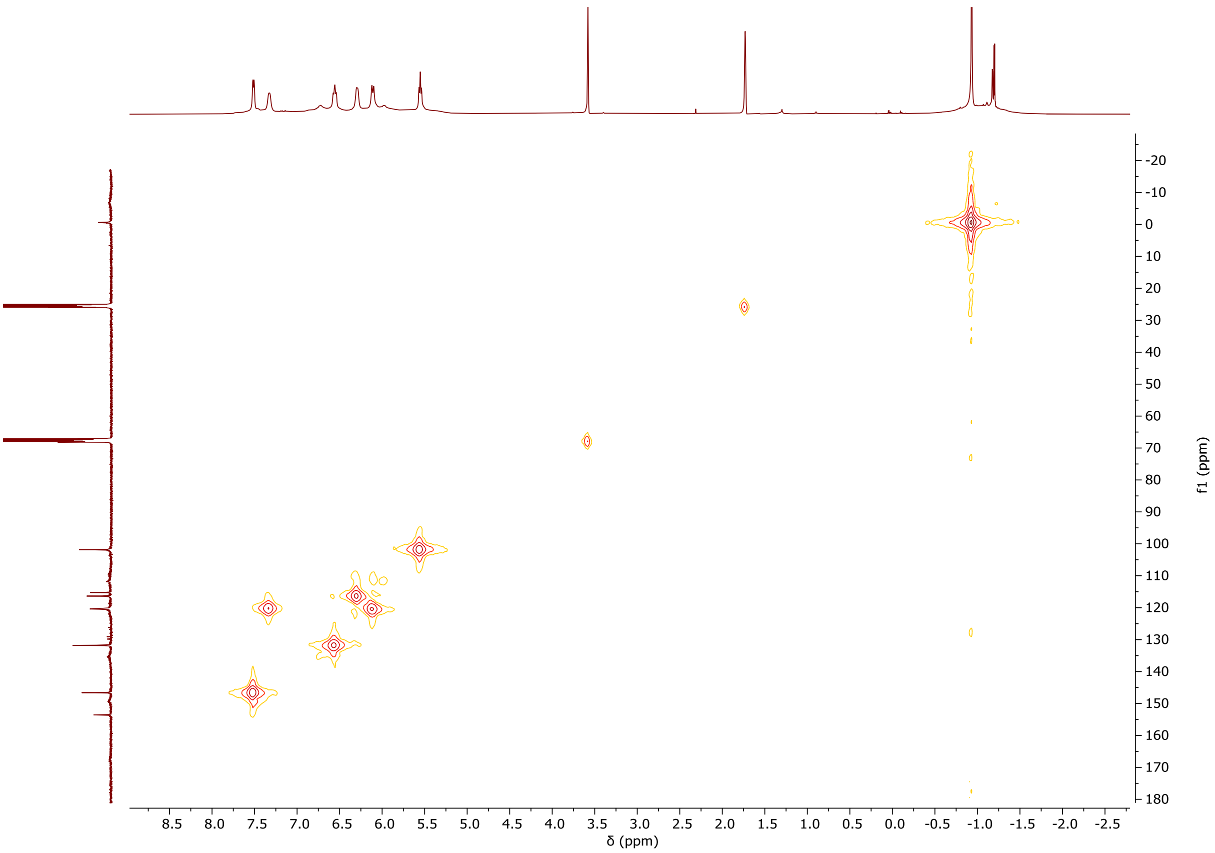

Figure S9. $^{1}H^{13}C$ HSQC NMR spectrum of **6** in THF-d_8.

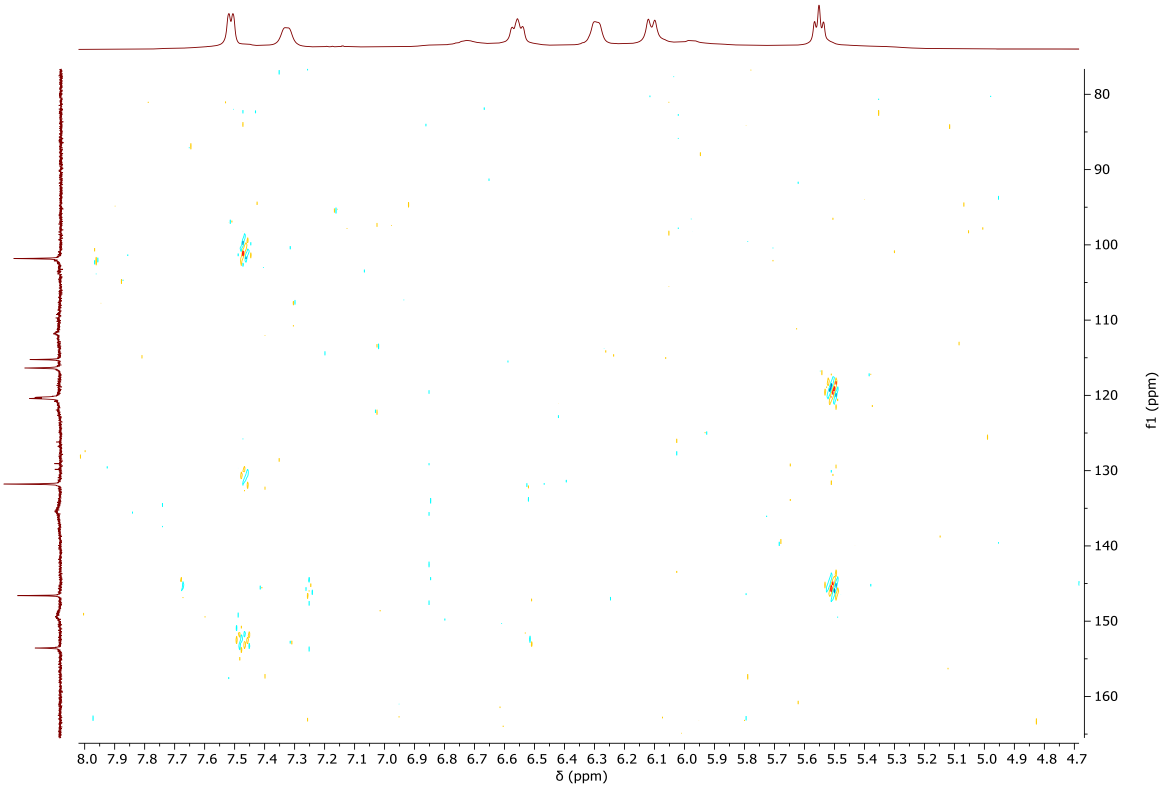

Figure S10. $^{1}H^{13}C$ HMBC NMR spectrum of **6** in THF-d_8.

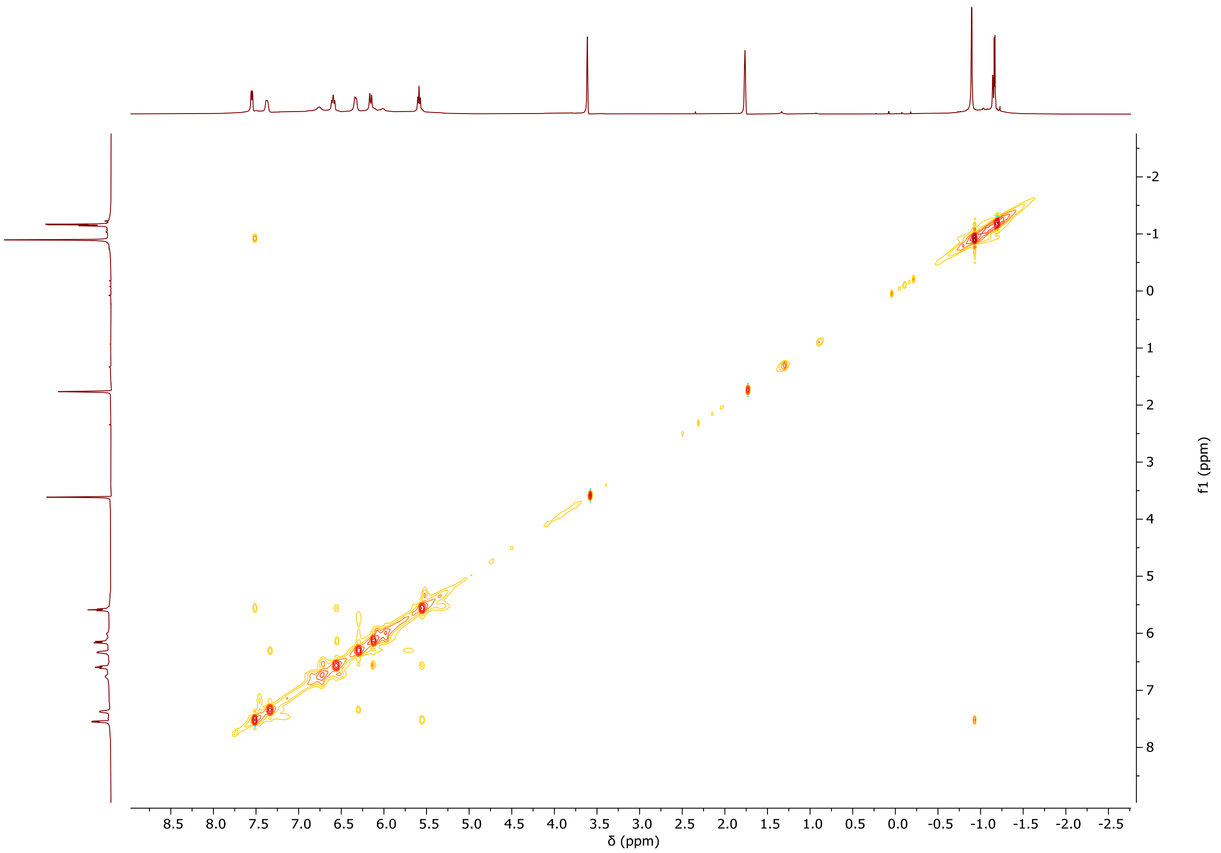

Figure S11. $^{1}H^{1}H$ NOESY NMR spectrum of **6** in THF-d_8.

4. EPR spectroscopy

4.1 EPR spectroscopy on $[1]^{\bullet-}$

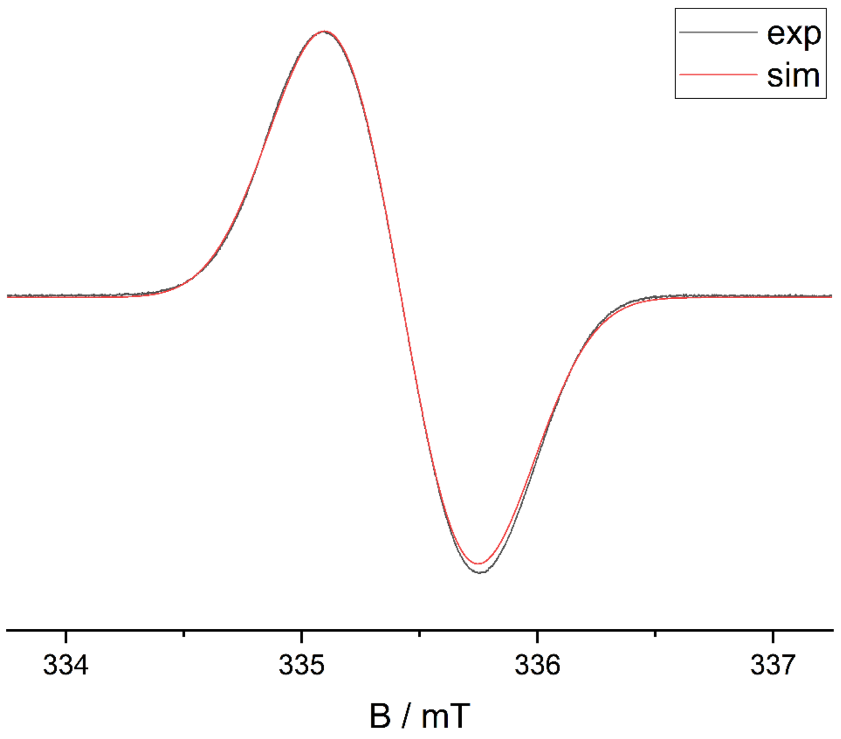

Figure S12. Simulated and experimental cw-EPR spectrum of **2** in THF. Measuring conditions: 10^{-4} mol/L in THF, r.t. isotropic, X-band (9.405578 GHz), 1 scan, 2048 pt). Simulation parameters: g = 2.0035, S = 0.5, Nucs: 1xN, 2xN, 2xN, 2xH, 2xH, 2xH, 2xH, A = -5.1746 1.6186 3.2963 4.8496 1.2293 1.5294 3.3900 (based on computed hyperfine couplings constants and under consideration of A values >1 MHz, details in section 9), lwpp = 0.29 mT.

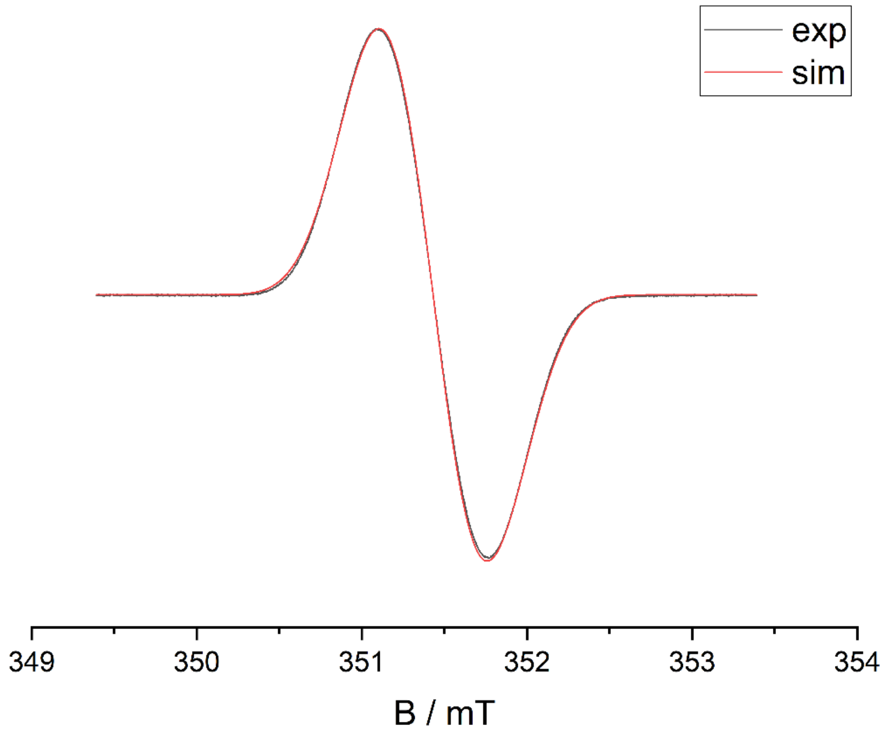

Figure S13 Simulated and experimental cw-EPR spectrum of **3** in THF. Measuring conditions: 10^{-4} mol/L in THF, r.t. isotropic, X-band (9.854700 GHz), 1 scan, 2048 pt). Simulation parameters: g = 2.0035, S = 0.5, Nucs: 1xN, 2xN, 2xN, 2xH, 2xH, 2xH, 2xH, A = -5.1746 1.6186 3.2963 4.8496 1.2293 1.5294 3.3900 (based on computed hyperfine couplings constants and under consideration of A values >1 MHz, details in section 9), lwpp = 0.30 mT.

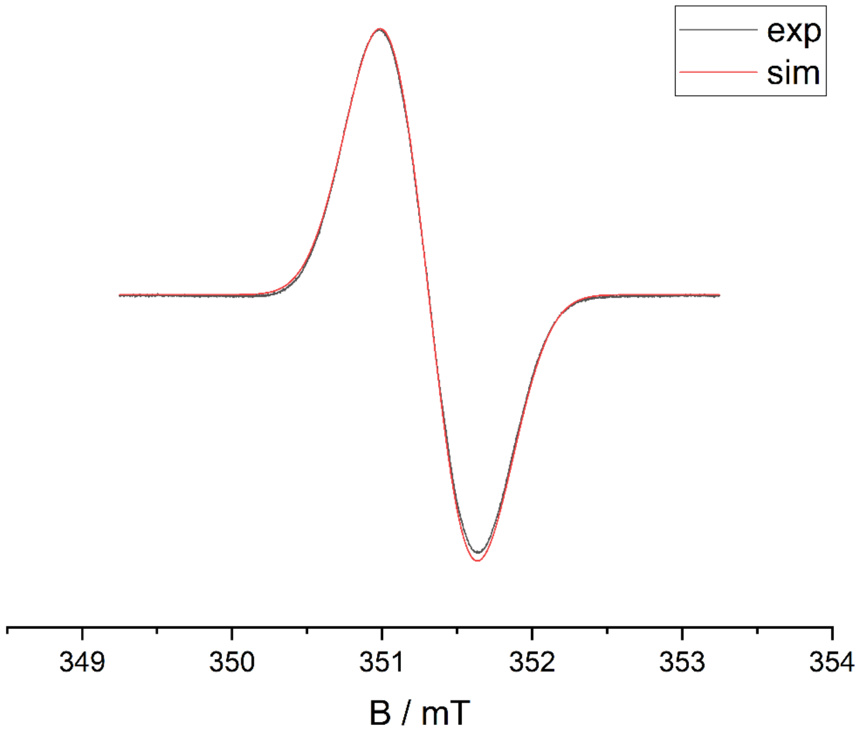

Figure S14 Simulated and experimental cw-EPR spectrum of **4** in THF. Measuring conditions: 10^{-4} mol/L in THF, r.t. isotropic, X-band (9.851139 GHz), 1 scan, 2048 pt). Simulation parameters: g = 2.0035, S = 0.5, Nucs: 1xN, 2xN, 2xN, 2xH, 2xH, 2xH, 2xH, A = -5.1746 1.6186 3.2963 4.8496 1.2293 1.5294 3.3900 (based on computed hyperfine couplings constants and under consideration of A values >1 MHz, details in section 9), lwpp = 0.28 mT.

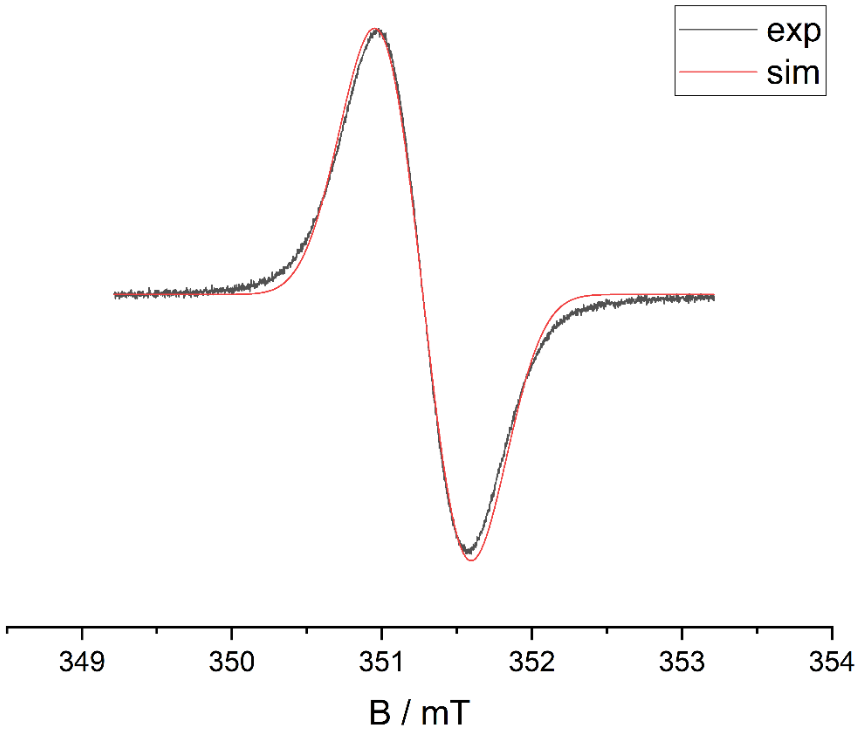

Figure S15 Simulated and experimental cw-EPR spectrum of **5** in THF. Measuring conditions: 10^{-4} mol/L (see comment below) in THF, r.t. isotropic, X-band (9.849789 GHz), 100 scans, 2048 pt). Simulation parameters: g = 2.0034, S = 0.5, Nucs: 1xN, 2xN, 2xN, 2xH, 2xH, 2xH, 2xH, A = -5.1746 1.6186 3.2963 4.8496 1.2293 1.5294 3.3900 (based on computed hyperfine couplings constants and under consideration of A values >1 MHz, details in section 9), lwpp = 0.27 mT.

Comment: The compound showed decomposition during dilution in the glovebox, which was indicated by a color change from pale red to yellow. Therefor the actual concentration is much lower than 10^{-4} mol/L and 100 Scans were necessary for a good signal to noise ratio.

4.2 EPR spectroscopy on [**1**]$^{2-}$

The dianion [**1**]$^{2-}$ is diamagnetic. Still, if samples were prepared by dissolving **6** in THF, single-line spectra with low intensity were observed in cw-EPR spectroscopic measurements. This is due to the extreme sensitivity towards oxidation of compounds including the dianion [**1**]$^{2-}$ to the paramagnetic radical anion [**1**]$^{\bullet-}$. The signal intensity was very low, when **6** was freshly synthesized by reducing **1** with an excess of potassium within the upper part of the EPR tube. For that, a solution of **1** (10^{-4} mol/L) was filled into an EPR tube, to whose upper glass wall a potassium mirror was condensed. The EPR tube was placed in such an orientation that the solution of **1** was stored overnight on top of the potassium mirror, which led to the expected color change from bright yellow to green. Afterwards the EPR tube was brought to an upright position and the EPR measurement was conducted as usual. We compared the relative intensity of the signal of the EPR spectrum of freshly prepared **6** with an authentic sample of **2**. Both measurements were conducted at the same concentration of 10^{-4} mol/L with equal settings for the receiver gain. Both obtained spectra are shown with the same intensity scale in Figure S16. A further plot of the EPR spectrum of **6** with scaled intensity is shown in Figure S17, in which the very low intense remaining single line spectrum, originating from traces of [**1**]$^{\bullet-}$, can be seen.

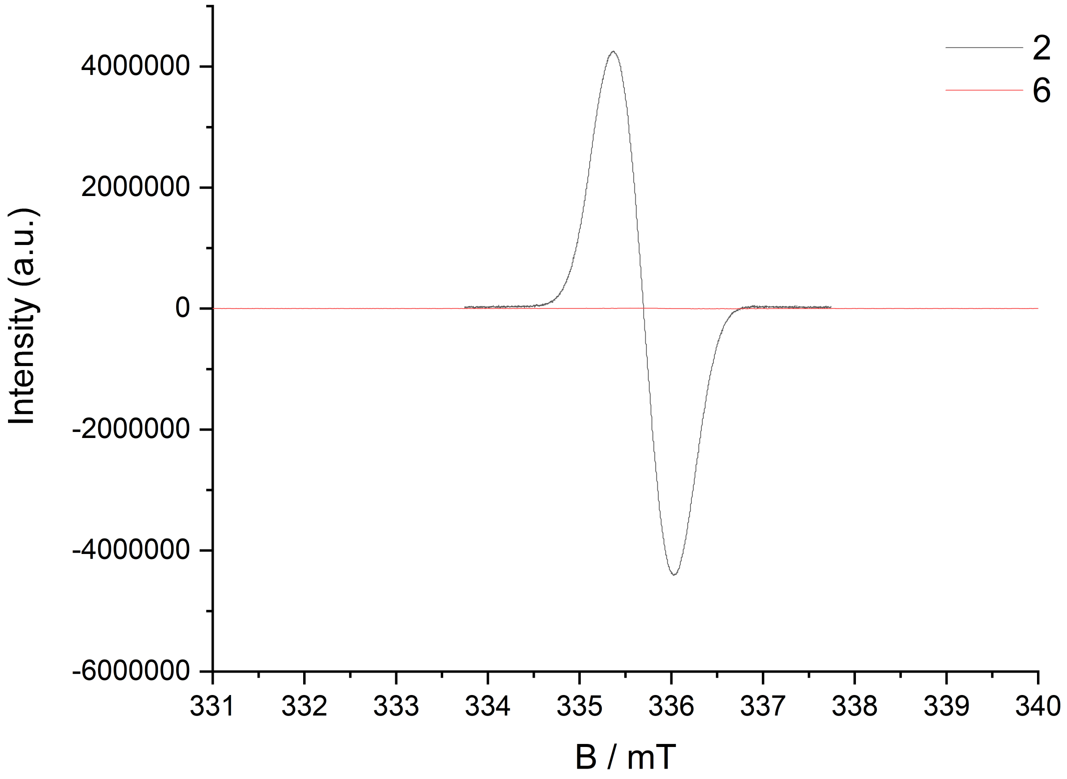

Figure S16. Comparison of the cw-EPR spectrum of **2** (black line) and **6** (red line) in THF with the same concentration of 10^{-4} mol/L. The latter was freshly prepared by reduction of **1** with an excess of potassium within the EPR tube.

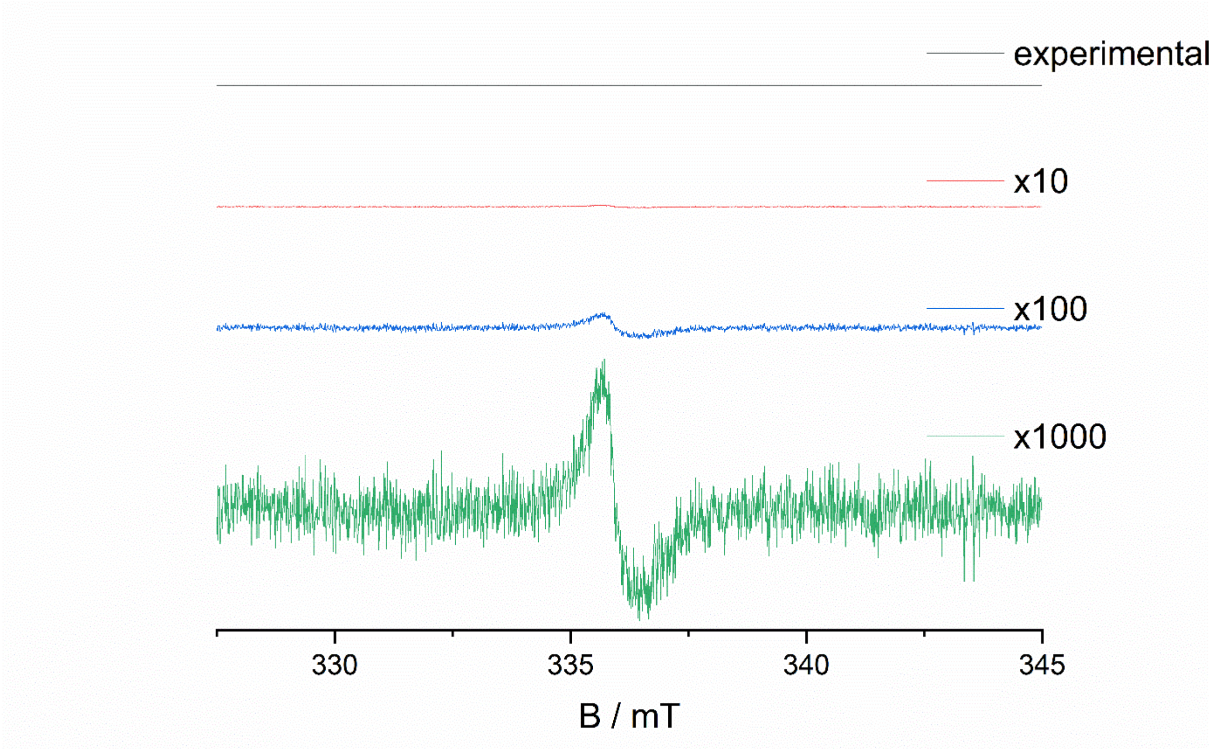

Figure S17. cw-EPR spectrum of **6**, which was freshly prepared by reduction of **1** with an excess of potassium within the EPR tube. The same spectrum is plotted with different intensity scaling.

5. IR spectra

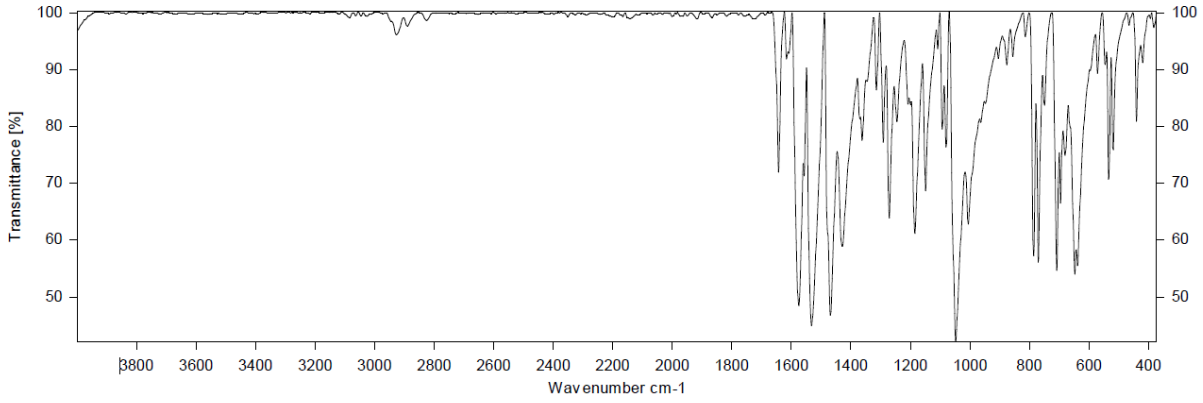

Figure S18. FT-IR spectrum of **1**.

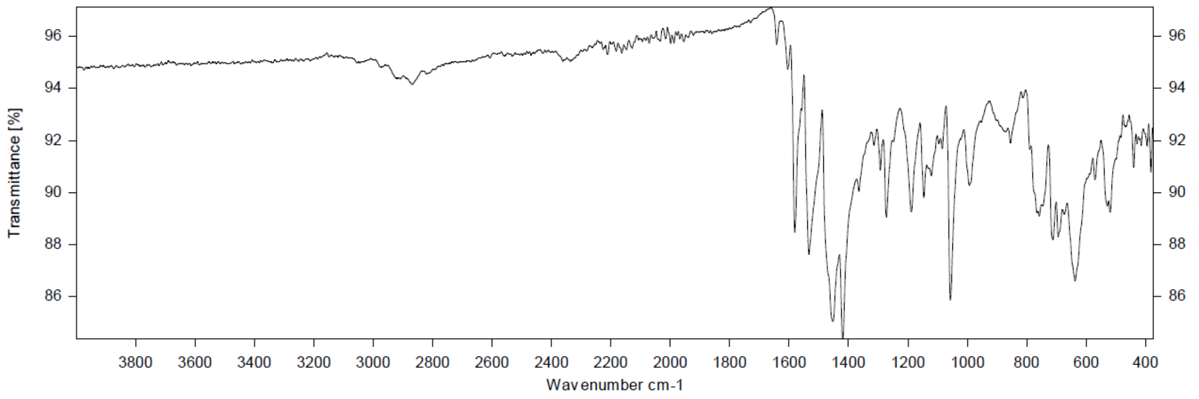

Figure S19. FT-IR spectrum of **2**.

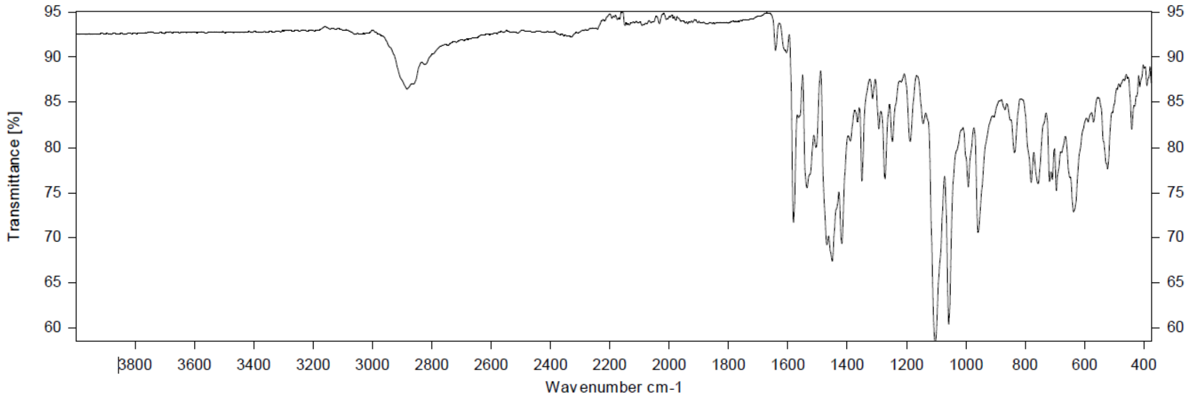

Figure S20. FT-IR spectrum of **3**.

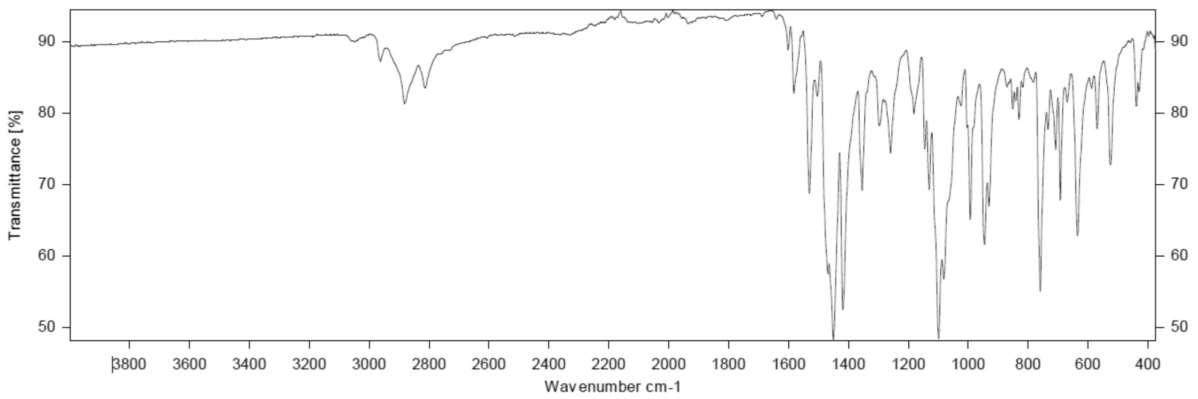

Figure S21. FT-IR spectrum of **4**.

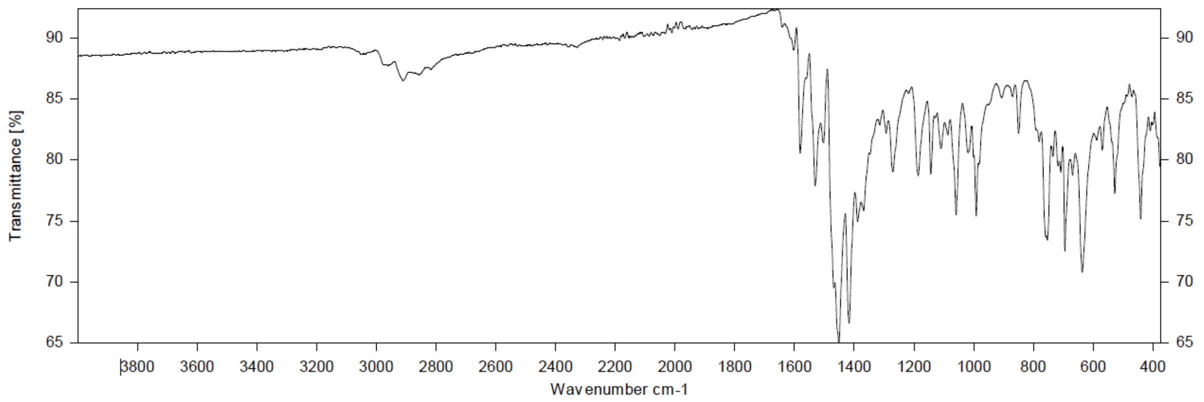

Figure S22. FT-IR spectrum of **5**.

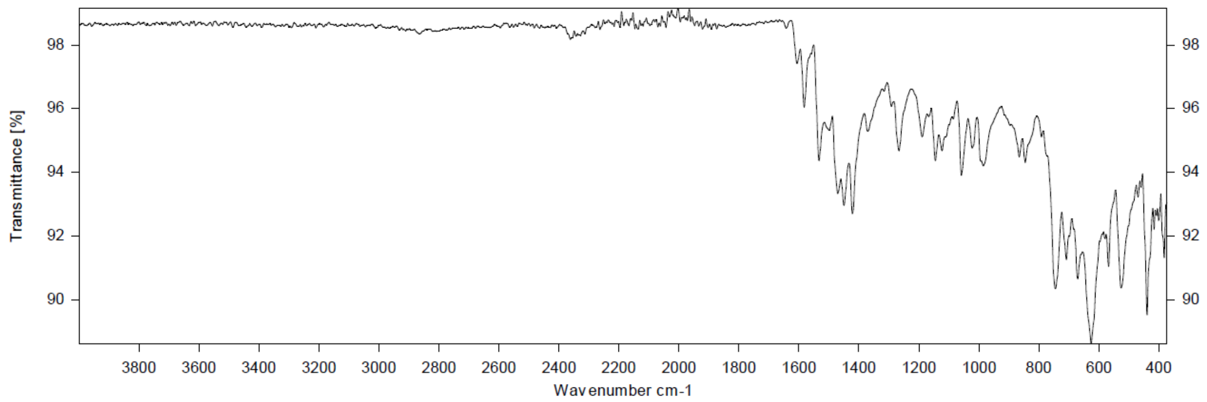

Figure S23. FT-IR spectrum of **6**.

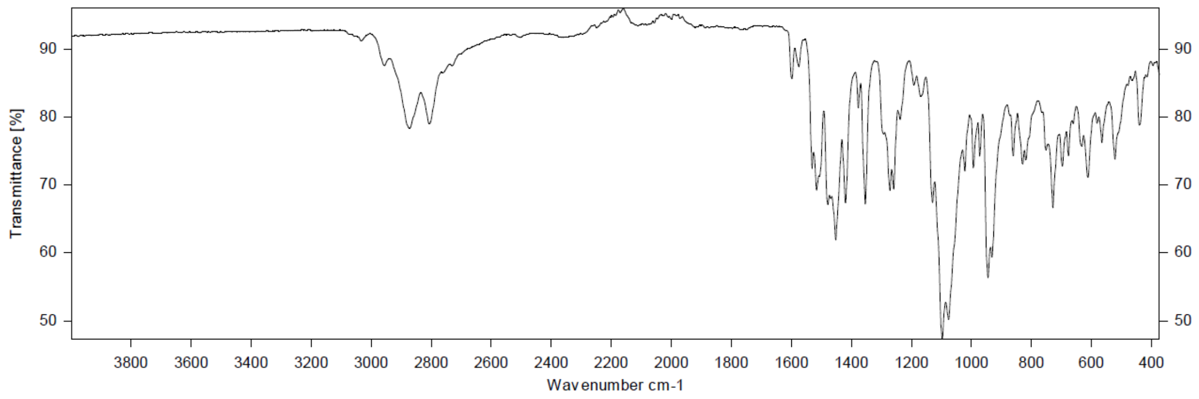

Figure S24. FT-IR spectrum of **7**.

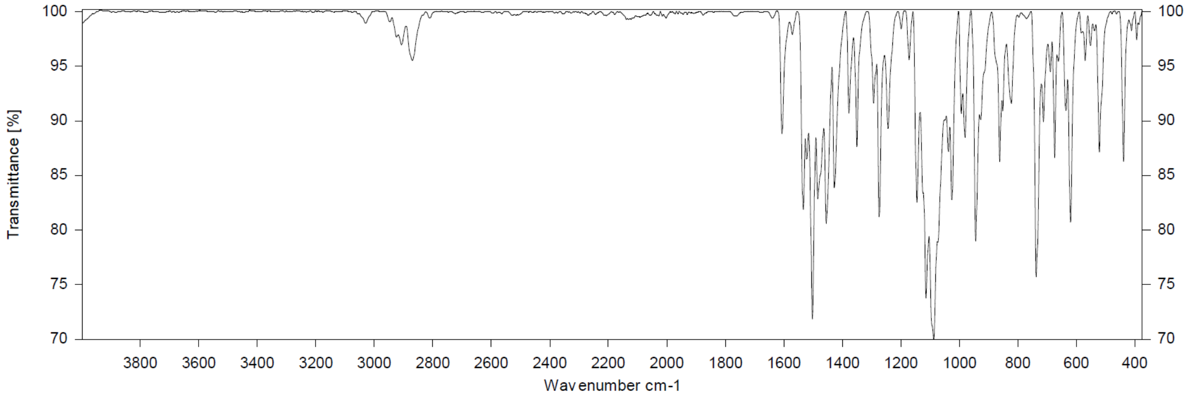

Figure S25. FT-IR spectrum of **8**.

6. UV/Vis spectra

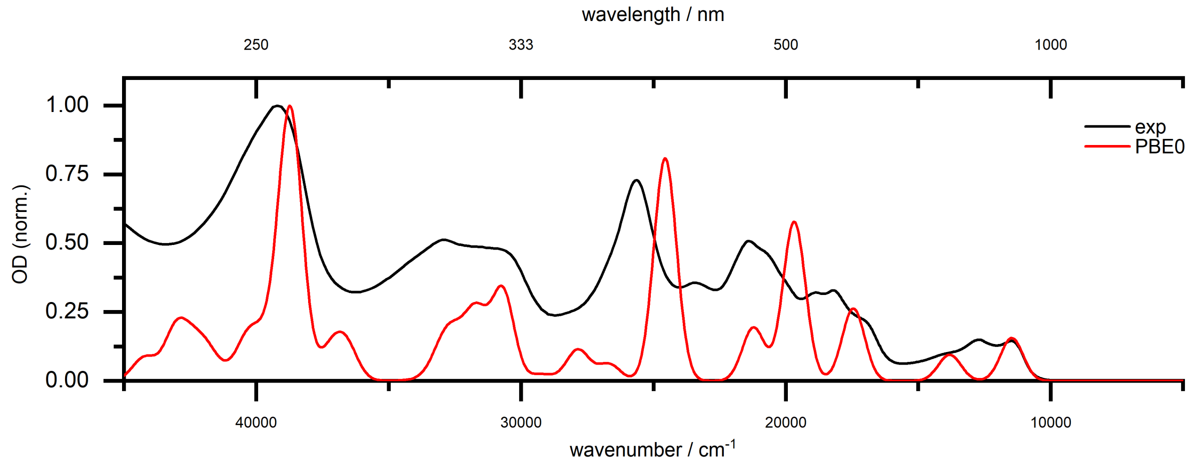

Figure S26. Experimental UV/Vis spectrum of **2** (10^{-3} mol/L in THF, black line) in comparison with the computed UV/Vis spectrum of **[1]**⁻ (red line), obtained on the PBE0-D3BJ/def2-TZVPD/CPCM(THF)//PBE0-D3BJ/def2-TZVP/CPCM(THF) level of theory and simulated by applying a gaussian line width of 1000 cm^{-1}.

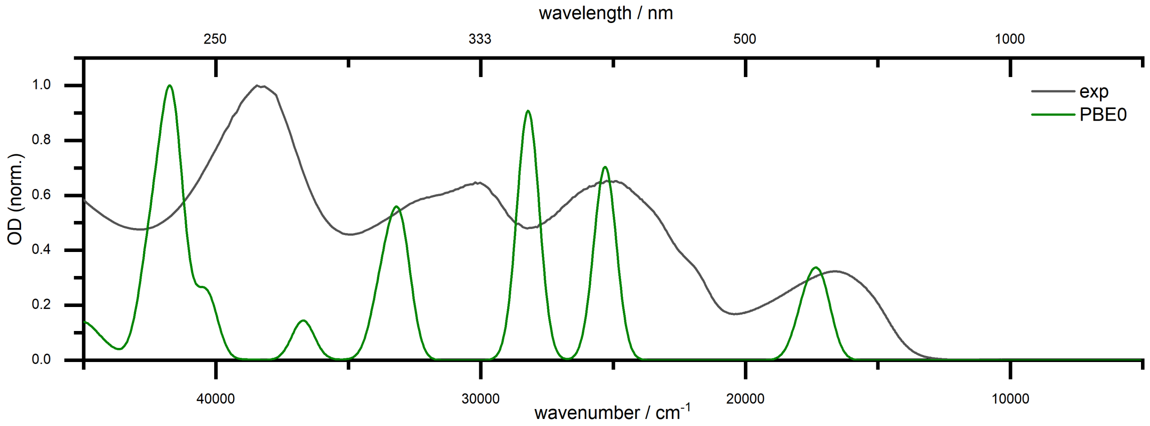

Figure S27. Experimental UV/Vis spectrum of **6** (10^{-3} mol/L in THF, black line) in comparison with the computed UV/Vis spectrum of $[\mathbf{1}]^{2-}$ (red line), obtained on the PBE0-D3BJ/def2-TZVPD/CPCM(THF)//PBE0-D3BJ/def2-TZVP/CPCM(THF) level of theory and simulated by applying a gaussian line width of 1000 cm^{-1}.

7. SC-XRD

[K(18-crown-6)][BPI–AlMe$_2$] (3)

Single crystals of **3** were obtained by storing a supersaturated THF solution at ambient temperature. **3** crystallizes in the hexagonal space group P6$_5$ with one formula unit in the asymmetric unit.

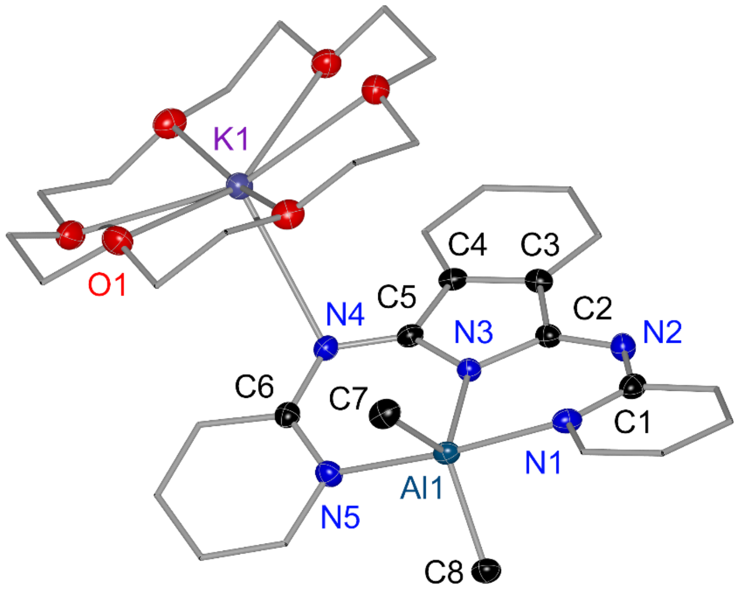

Figure S28. SC-XRD structure of **2**. Hydrogen atoms are omitted for clarity. Thermal ellipsoids are given at the 30% probability level. Selected bond lengths and angles in pm and °: N1–Al1 212.6(2), N3–Al1 191.65(17), N5–Al1 212.6(2), C7–Al1 198.7(3), C8–Al1 199.0(3), N1–C1 136.0(3), N5–C6 135.8(3), C1–N2 134.8(3), C6–N4 134.9(3), N2–C2 133.2(3), N4–C5 134.3(3), C2–C3 143.3(3), C5–C4 143.7(3), C3–C4 139.7(3), K1–N4 292.13(19), C7–Al1–C8 125.61(11), C7–Al1–N3 116.81(10), N1–Al1–N3 86.62(7).

Table S1. Crystallographic details of **3**.

CCDC #	2513348
Empirical formula	$C_{32}H_{42}AlKN_5O_6$
Formula weight	658.78
Temperature/K	180(2)
Crystal system	hexagonal
Space group	$P6_5$
a / Å	10.8690(2)
b / Å	10.8690(2)
c / Å	48.8527(11)
α / °	90
β / °	90
γ / °	120
Volume/Å^3	4998.0(2)
Z	6
ρ_{calc}g/cm^3	1.313
μ / mm^{-1}	1.348
F(000)	2094.0
Radiation	GaKα (λ = 1.34143)
$\Theta_{min} – \Theta_{max}$ / °	8.322 to 124.968
Reflections collected	24950
Independent reflections	7389
R_{int}	0.0330
R_{sigma}	0.0329
Data/restraints/parameters	7389/1/574
GooF	0.968
R_1	0.0293
wR_2	0.0645
R_1(all)	0.0386
wR_2(all)	0.0669
ρ_{e-} max/ρ_{e-} min/ e Å^{-3}	0.15/-0.15
Flack parameter	0.015(9)

[K(2.2.2-cryptand)][BPI–AlMe$_2$] (4)

Single crystals of **4** were obtained by storage of a THF solution at ambient temperature overnight after addition of approximately half the volume of *n*-hexane. **4** crystallizes in the monoclinic space group P2$_1$/c with one formula unit in the asymmetric unit.

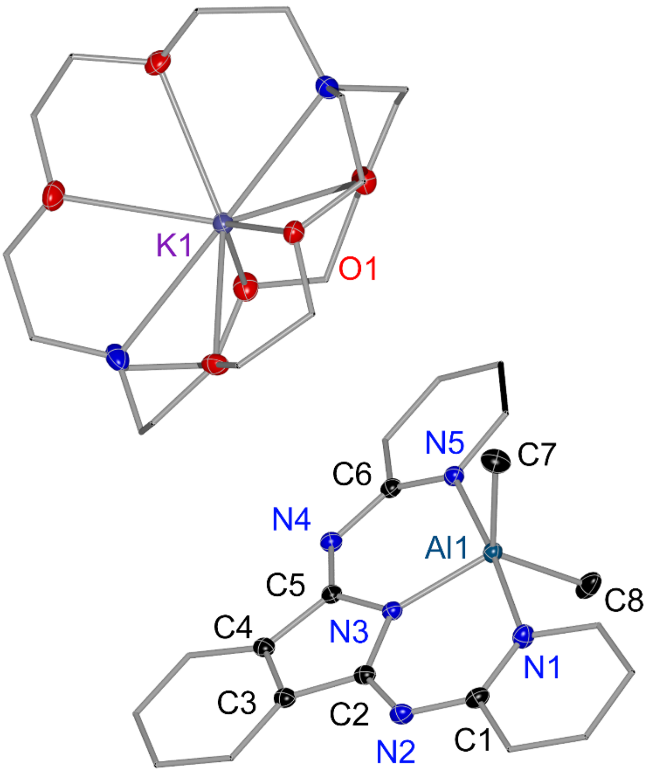

Figure S29. SC-XRD structure of **4**. Hydrogen atoms are omitted for clarity. Thermal ellipsoids are given at the 30% probability level. Selected bond lengths and angles in pm and °: N1–Al1 209.37(9), N3–Al1 192.77(10), N5–Al1 209.81(10), C7–Al1 200.17(13), C8–Al1 200.11(13), N1–C1 136.19(14), N5–C6 135.98(15), C1–N2 134.80(14), C6–N4 134.09(15), N2–C2 133.71(13), N4–C5 133.38(13), C2–C3 143.60(15), C5–C4 143.45(16), C3–C4 140.75(14), C7–Al1–C8 123.16(6), C7–Al1–N3 118.77(6), N1–Al1–N3 87.67(4).

Table S2. Crystallographic details of **4**.

CCDC #	2513358
Empirical formula	$C_{38}H_{55}AlKN_7O_6$
Formula weight	771.97
Temperature/K	150(2)
Crystal system	monoclinic
Space group	$P2_1/c$
a / Å	12.9990(2)
b / Å	15.6228(2)
c / Å	19.8799(4)
α / °	90
β / °	97.0660(10)
γ / °	90
Volume/Å^3	4006.56(11)
Z	4
ρ_{calc}g/cm^3	1.280
μ / mm^{-1}	1.191
F(000)	1648.0
Radiation	GaKα (λ = 1.34143)
$\Theta_{min} – \Theta_{max}$ / °	5.96 to 124.992
Reflections collected	33061
Independent reflections	9467
R_{int}	0.0144
R_{sigma}	0.0134
Data/restraints/parameters	9467/0/481
GooF	1.042
R_1	0.0317
wR_2	0.0872
R_1(all)	0.0365
wR_2(all)	0.0891
ρ_{e-} max/ρ_{e-} min/ e Å^{-3}	0.31/-0.69

$[CoCp^*_2][BPI–AlMe_2]$ (5)

Single crystals of **5** were obtained by slow solvent evaporation at ambient pressure of a THF solution. **5** crystallizes in the monoclinic space group $P2_1/c$ with two formula units and two non-coordinated THF molecules in the asymmetric unit.

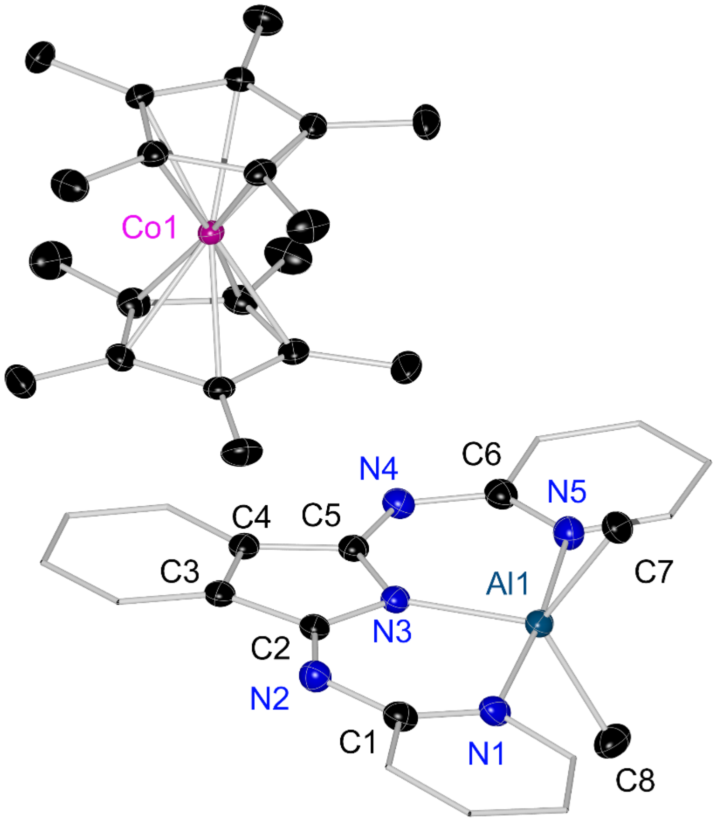

Figure S30. SC-XRD structure of **5**. Hydrogen atoms and one non-coordinated THF molecule are omitted for clarity. Thermal ellipsoids are given at the 30% probability level. Selected bond lengths and angles in pm and °: N1–Al1 209.36(19), N3–Al1 193.50(17), N5–Al1 209.62(18), C7–Al1 199.9(2), C8–Al1 199.7(2), N1–C1 136.1(3), N5–C6 136.5(3), C1–N2 134.8(3), C6–N4 134.8(3), N2–C2 133.0(3), N4–C5 133.3(3), C2–C3 143.6(3), C5–C4 143.8(3), C3–C4 139.9(3), C7–Al1–C8 119.25(10), C7–Al1–N3 121.15(9), N1–Al1–N3 87.15(7).

Table S3. Crystallographic details of **5**.

CCDC #	2513359
Empirical formula	$C_{44}H_{56}AlCoN_5O$
Formula weight	756.84
Temperature/K	150
Crystal system	monoclinic
Space group	$P2_1/c$
a / Å	17.2710(4)
b / Å	21.7722(5)
c / Å	21.3118(6)
α / °	90
β / °	101.372(2)
γ / °	90
Volume/Å^3	7856.5(3)
Z	8
ρ_{calc}g/cm^3	1.280
μ / mm^{-1}	2.734
F(000)	3224.0
Crystal size/mm^3	0.3×0.28×0.26
Radiation	Ga Kα (λ = 1.34143)
Θ_{min} – Θ_{max} / °	4.54 to 128.808
Reflections collected	52744
Independent reflections	18665
R_{int}	0.0199
R_{sigma}	0.0272
Data/restraints/parameters	18665/210/1058
GooF	1.014
R_1	0.0414
wR_2	0.0963
R_1(all)	0.0625
wR_2(all)	0.1072
ρ_{e-} max/ρ_{e-} min/ e Å^{-3}	0.80/–0.49

[K(2.2.2-cryptand)]$_2$[BPI–AlMe$_2$] (7)

Single crystals of **7** were obtained by layering a solution of **6** in THF with a solution of two equivalents of 2.2.2-cryptand in THF. **7** crystallizes in the triclinic space group $P\bar{1}$ with two formula units and two non-coordinated THF molecules in the asymmetric unit.

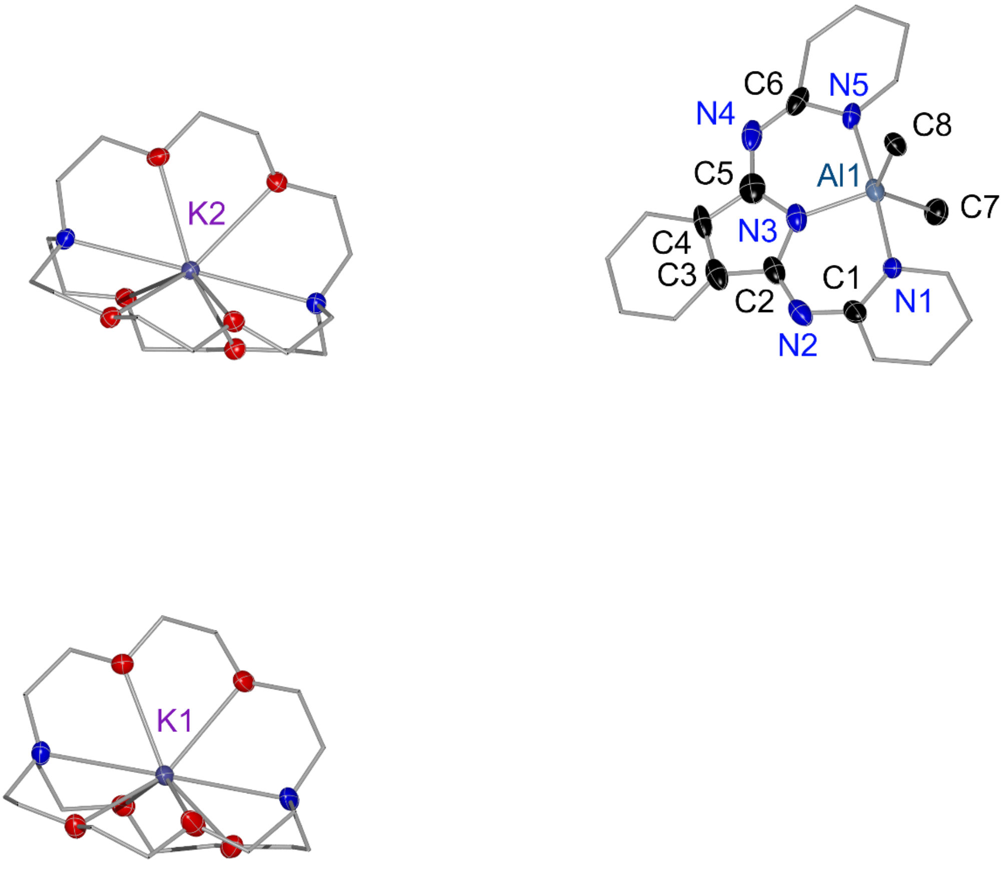

Figure S31. SC-XRD structure of **7**. Hydrogen atoms and one non-coordinated THF molecule are omitted for clarity. Thermal ellipsoids are given at the 30% probability level. Selected bond lengths and angles in pm and °: N1–Al1 210.1(6), N3–Al1 192.7(7), N5–Al1 208.0(7), C7–Al1 202.2(2), C8–Al1 201.1(2), N1–C1 138.7(10), N5–C6 138.7(9), C1–N2 130.5(10), C6–N4 132.0(10), N2–C2 137.0(10), N4–C5 137.0(10), C2–C3 139.6(10), C5–C4 138.7(10), C3–C4 144.5(10), C7–Al1–C8 120.84(9), C7–Al1–N3 122.9(5), N1–Al1–N3 85.6(5).

Table S4. Crystallographic details of **7**.

CCDC #	2513360
Empirical formula	$C_{56}H_{90.21}AlK_2N_9O_{12}$
Formula weight	1186.75
Temperature/K	180(2)
Crystal system	triclinic
Space group	P-1
a / Å	13.5822(4)
b / Å	22.4360(6)
c / Å	24.7943(7)
α / °	109.610(2)
β / °	97.154(2)
γ / °	106.471(2)
Volume/Å^3	6620.3(3)
Z	4
ρ_{calc}g/cm^3	1.191
μ / mm^{-1}	1.274
F(000)	2545.0
Crystal size/mm^3	0.150×0.093×0.030
Radiation	Ga Kα (λ = 1.34143)
Θ_{min} – Θ_{max} / °	5.972–109.998
Reflections collected	71861
Independent reflections	24211
R_{int}	0.0293
R_{sigma}	0.0354
Data/restraints/parameters	24211/1008/2324
GooF	1.042
R_1	0.0439
wR_2	0.1142
R_1(all)	0.0690
wR_2(all)	0.1229
ρ_{e-} max/ρ_{e-} min/ e Å^{-3}	0.30/-0.24

[Na(15-crown-6)]$_2$[BPI–AlMe$_2$] (8)

Single crystals of **8** were obtained by slow solvent evaporation at ambient pressure of of a solution of **8** in THF. **8** crystallizes in the orthorhombic space group Pbcn with half a formula unit in the asymmetric unit.

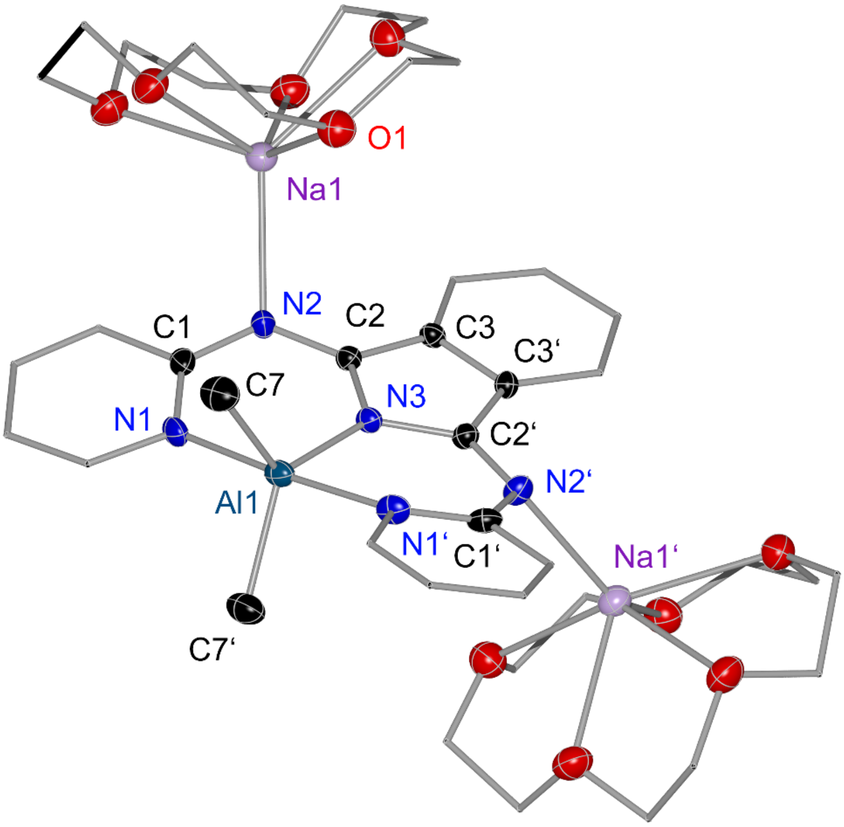

Figure S32. SC-XRD structure of **8**. Hydrogen atoms are omitted for clarity. Thermal ellipsoids are given at the 30% probability level. Selected bond lengths and angles in pm and °: N1–Al1 209.11(19), N3–Al1 192.6(2), C7–Al1 199.4(3), N1–C1 137.3(3), C1–N2 133.0(3), N2–C2 137.8(3), C2–C3 140.5(3), C3–C4 141.1(3), C7–Al1–C7' 119.6(2), C7–Al1–N3 120.19(11), N1–Al1–N3 87.12(5).

Table S5. Crystallographic details of **8**.

CCDC #	2513361
Empirical formula	$C_{40}H_{58}AlN_5Na_2O_{10}$
Formula weight	841.87
Temperature/K	180
Crystal system	orthorhombic
Space group	Pbcn
a / Å	16.8354(9)
b / Å	18.1280(7)
c / Å	13.9358(5)
α / °	90
β / °	90
γ / °	90
Volume/Å^3	4253.1(3)
Z	4
ρ_{calc}g/cm^3	1.315
μ / mm^{-1}	0.727
F(000)	1792.0
Crystal size/mm^3	0.05 × 0.043 × 0.04
Radiation	Ga Kα (λ = 1.34143)
Θ_{min} – Θ_{max} / °	8.328 to 114.988
Reflections collected	13160
Independent reflections	4316
R_{int}	0.0597
R_{sigma}	0.1141
Data/restraints/parameters	4316/0/379
GooF	0.826
R_1	0.0431
wR_2	0.0765
R_1(all)	0.1290
wR_2(all)	0.0927
ρ_{e-} max/ρ_{e-} min/ e Å^{-3}	0.25/-0.26

$[K_4(thf)_6][BPI–AlMe_2]_3$ (10)

Single crystals of **10** were obtained by treating a THF solution of **10** with approximately double the volume of *n*-hexane. **10** crystallizes in the monoclinic space group C2/c with half a formula unit in the asymmetric unit.

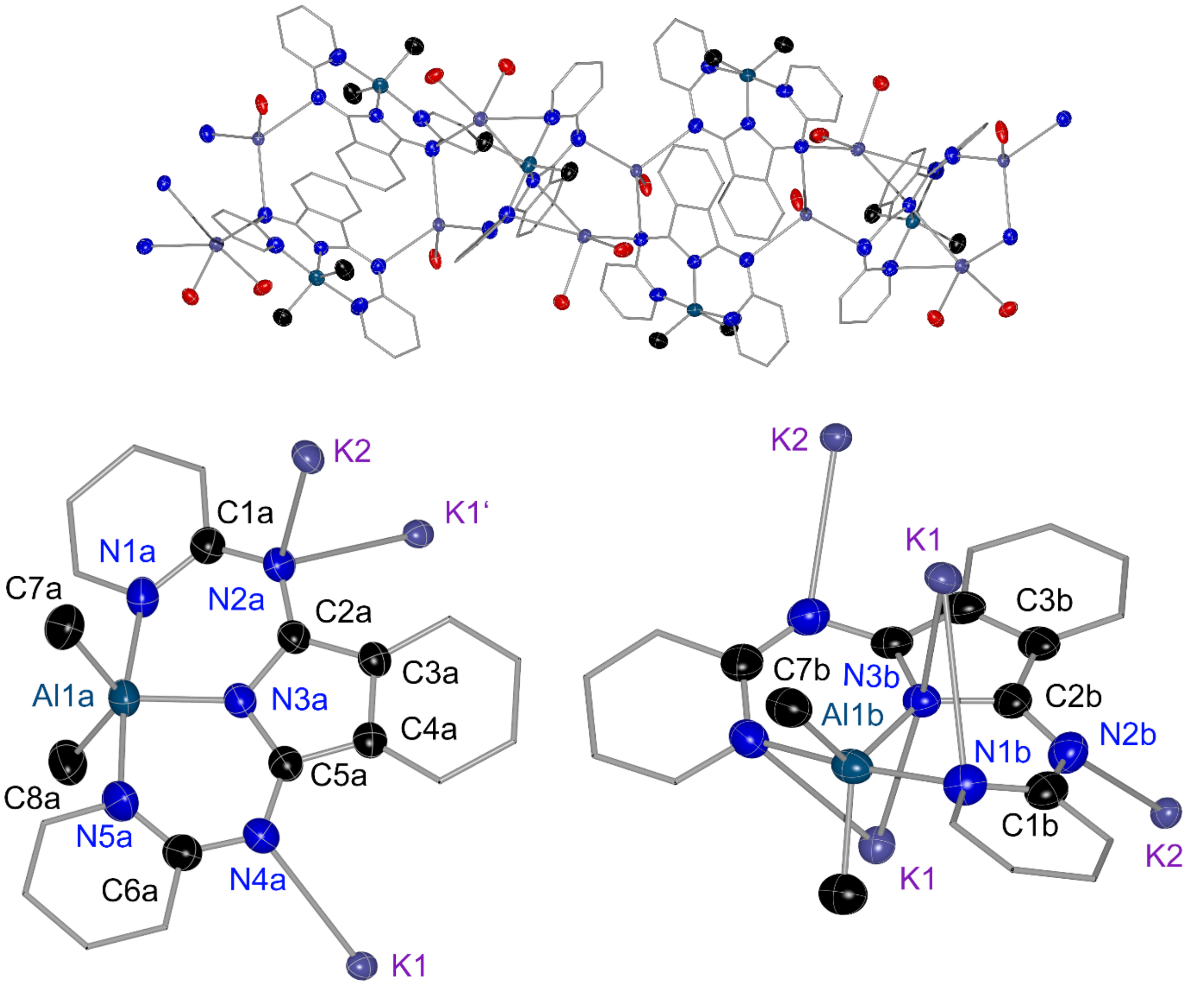

Figure S33. SC-XRD structure of **10**. Hydrogen atoms are omitted for clarity. Thermal ellipsoids are given at the 30% probability level. Selected bond lengths and angles in pm and °: N1a–Al1a 209.1(3), N3a–Al1a 192.5(3), N5a–Al1a 209.5(3), C7a–Al1a 198.7(3), C8a–Al1a 200.0(4), N1a–C1a 136.1(4), N5a–C6a 135.9(4), C1a–N2a 135.9(4), C6a–N4a 135.1(4), N2a–C2a 134.2(4), N4a–C5a 134.2(4), C2a–C3a 143.0(4), C5a–C4a 143.6(4), C3a–C4a 140.6(4), C7a–Al1a–C8a 122.76(16), C7a–Al1a–N3a 114.65(14), N1a–Al1a–N3a 86.92(11).

N1b–Al1b 210.9(3), N3b–Al1b 190.6(4), C7b–Al1b 200.3(3), N1b–C1b 138.2(4), C1b–N2b 131.4(4), N2b–C2b 137.6(4), C2b–C3b 139.4(4), C3b–C3‘b 144.7(6), C7b–Al1b–C7‘b 120.3(2), C7b–Al1b–N3b 119.86(11), N1b–Al1b–N3b 86.36(9).

Table S6. Crystallographic details of **10**.

CCDC #	2513362
Empirical formula	$C_{84}H_{102}Al_3K_4N_{15}O_6$
Formula weight	1655.14
Temperature/K	200
Crystal system	monoclinic
Space group	C2/c
a / Å	27.7577(11)
b / Å	11.6972(7)
c / Å	28.4277(11)
α / °	90
β / °	107.969(3)
γ / °	90
Volume/Å^3	8779.9(7)
Z	4
ρ_{calc}g/cm^3	1.252
μ / mm^{-1}	0.292
F(000)	3496.0
Crystal size/mm^3	0.4 × 0.2 × 0.1
Radiation	Mo Kα (λ = 0.71073)
Θ_{min} – Θ_{max} / °	3.012 to 51
Reflections collected	28151
Independent reflections	8167
R_{int}	0.0648
R_{sigma}	0.0740
Data/restraints/parameters	8167/62/601
GooF	1.001
R_1	0.0521
wR_2	0.1002
R_1(all)	0.1256
wR_2(all)	0.1265
ρ_{e-} max/ρ_{e-} min/ e Å^{-3}	0.19/-0.21

Table S7. Overview of selected bond lengths within the neutral, anionic and dianionic BPI–$AlMe_2$ framework, obtained experimentally for **1**,[2] **3**, **4**, **5**, **7**, **8** and **10**. For comparison of BPI bond lengths typically observed in the mono-, di- and trianionic states see our previous work.[19,20]

	d (Al–C7) / pm	*d* (N1–C1) / pm	*d* (C1–N2) / pm	*d* (N2–C2) / pm	*d* (C2–C3) / pm	*d* (C3–C4) / pm
**1**	198.7	134.9	138.2	129.6	147.4	138.1
**3**	198.7	136.0	134.8	133.2	143.3	139.7
**4**	200.2	136.2	134.8	133.7	143.6	140.8
**5**	199.9	136.1	134.8	133.0	143.6	139.9
**7**[a]	202.2	138.7	130.5	137.0	139.6	144.5
**8**	199.4	137.3	133.0	137.8	140.5	141.1
**10**	198.7 [**1**]$^{\bullet-}$	136.1	135.9	134.2	143.0	140.6[b]
	200.3 [**1**]$^{2-}$	138.2	131.4	137.6	139.4	144.7[b]

[a] Values should not be interpreted due to insufficient quality of the diffractometric data set. [b] Values are given for *d* (C3–C3').

8. pXRD

The singly reduced derivative **2**, which did not yield single crystals suitable for SC-XRD, was investigated by pXRD. The measurement shows reflexes with weak intensity. The compound is expected to be mainly obtained as amorphous solid with low crystallinity. The presence of **1** as an impurity in **2** can be excluded by comparison of the pXRD data of **2** with the pXRD data predicted for **1** based on SC-XRD data (Figure S34). **6** shows only non-specific broad reflexes with very low intensity, suggesting that this compound is predominately amorphous as solid as well (Figure S35).

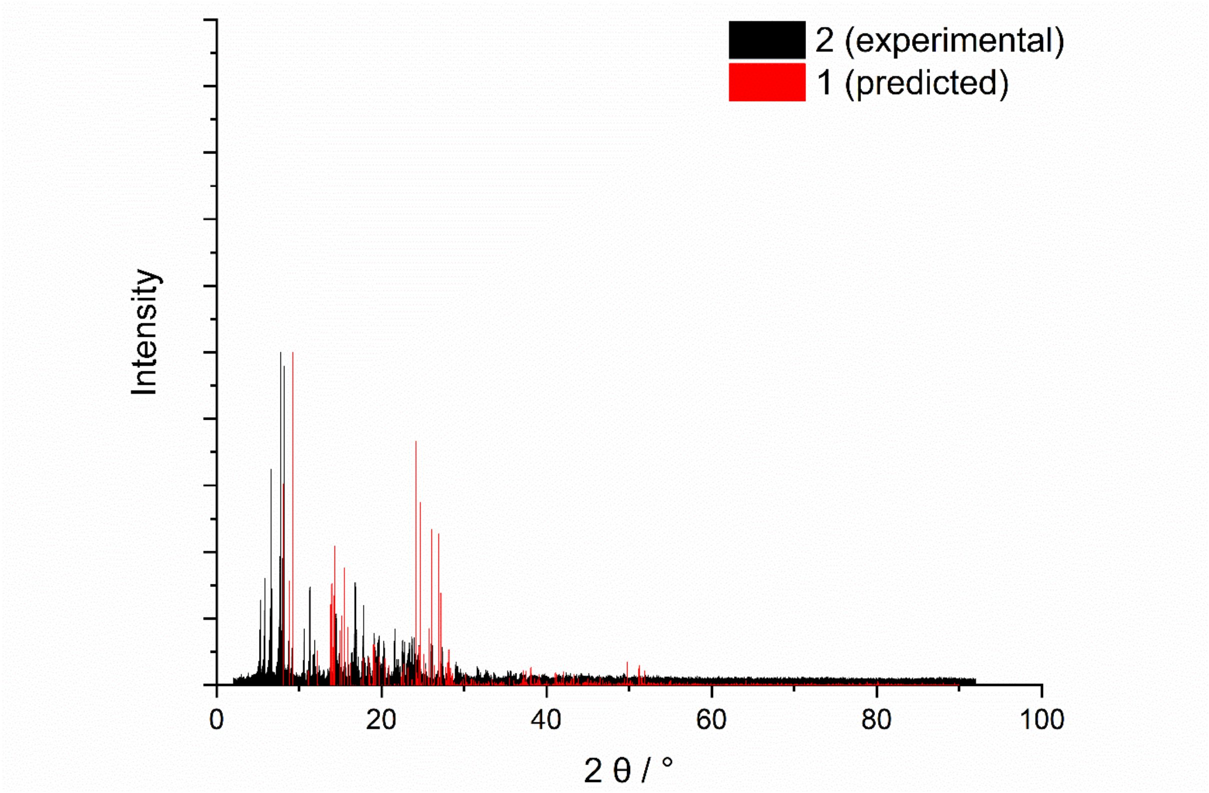

Figure S34. pXRD data of **2** (black line) in comparison with the predicted pXRD of **1** (red bars), calculated pXRD generated based on SC-XRD data of **1**.

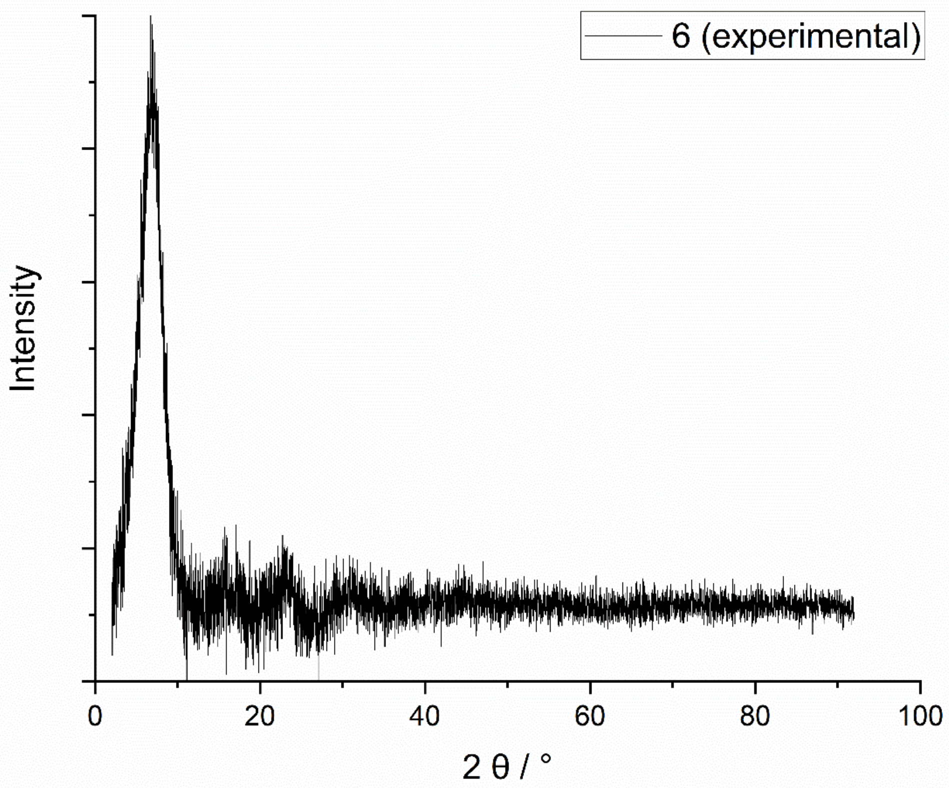

Figure S35. Experimental pXRD spectrum of **6**.

Furthermore, we investigated **3**, **4**, **5** and **8** by pXRD. The absence of any reflexes related to reported values for potential decomposition products like KOH, K_2O, KO_2, K_2O_2, 18-crown-6 or 2.2.2-cryptand further support the purity of the obtained compounds. For **3** and **4**, the pXRD data do not well reproduce those predicted based on the SC-XRD dataset. We interpret this as consequence of multiple potential phases, in which the compounds can crystallize, and it is not given that, upon sole removal of the solvent after synthesis, the same phase is obtained as for the picked single crystal. For **5** and **8**, the experimental and calculated pXRD match surprisingly well, indicating that less different phases exist for these compounds and that, after removal of the solvent during the synthesis, the same crystalline phase that we observe for SC-XRD measurements can be obtained. In general, the intensity of the reflexes of the powder diffractograms was low, indicating a predominately amorphous character of the compounds, which is usually obtained via the above communicated synthetic procedures.

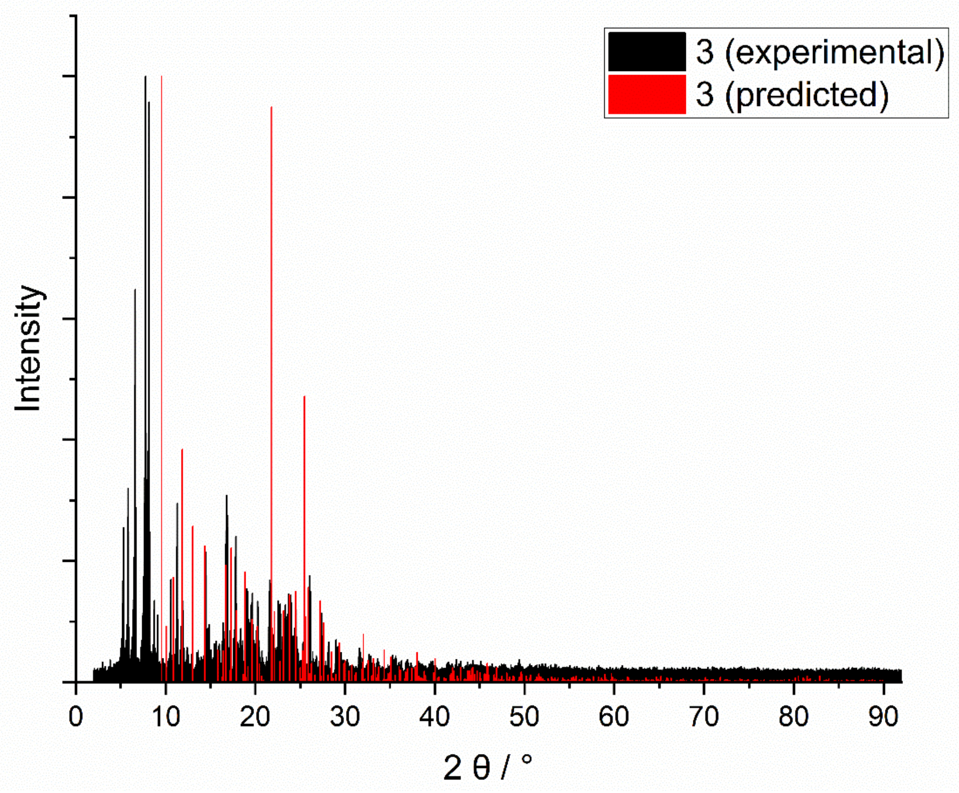

Figure S36. Experimental and calculated pXRD of **3**.

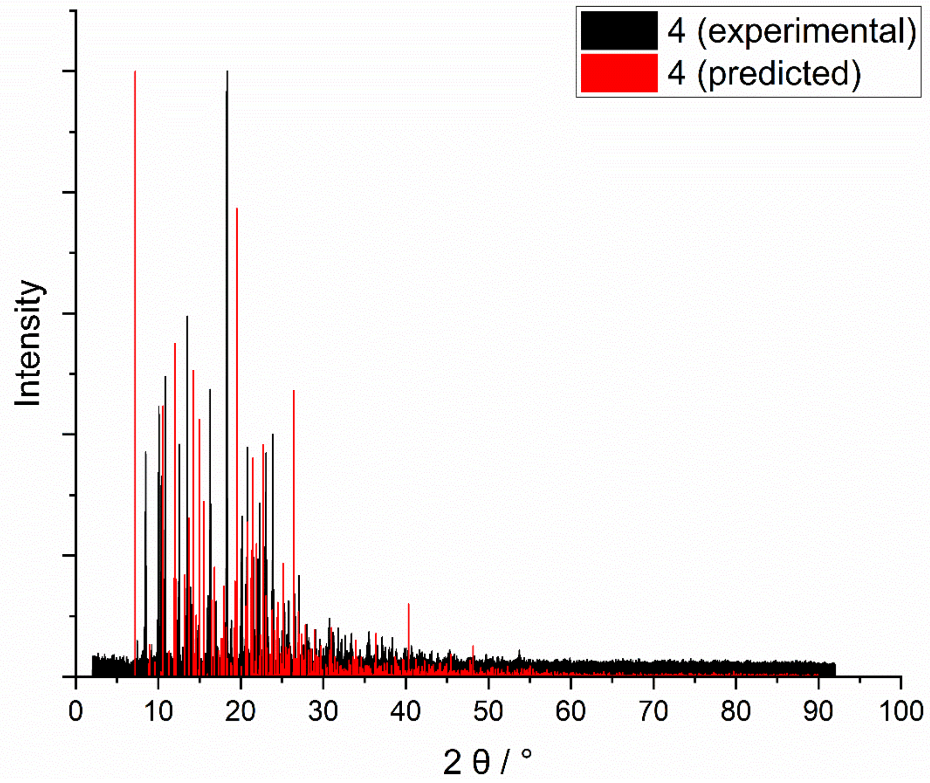

Figure S37. Experimental and calculated pXRD of **4**.

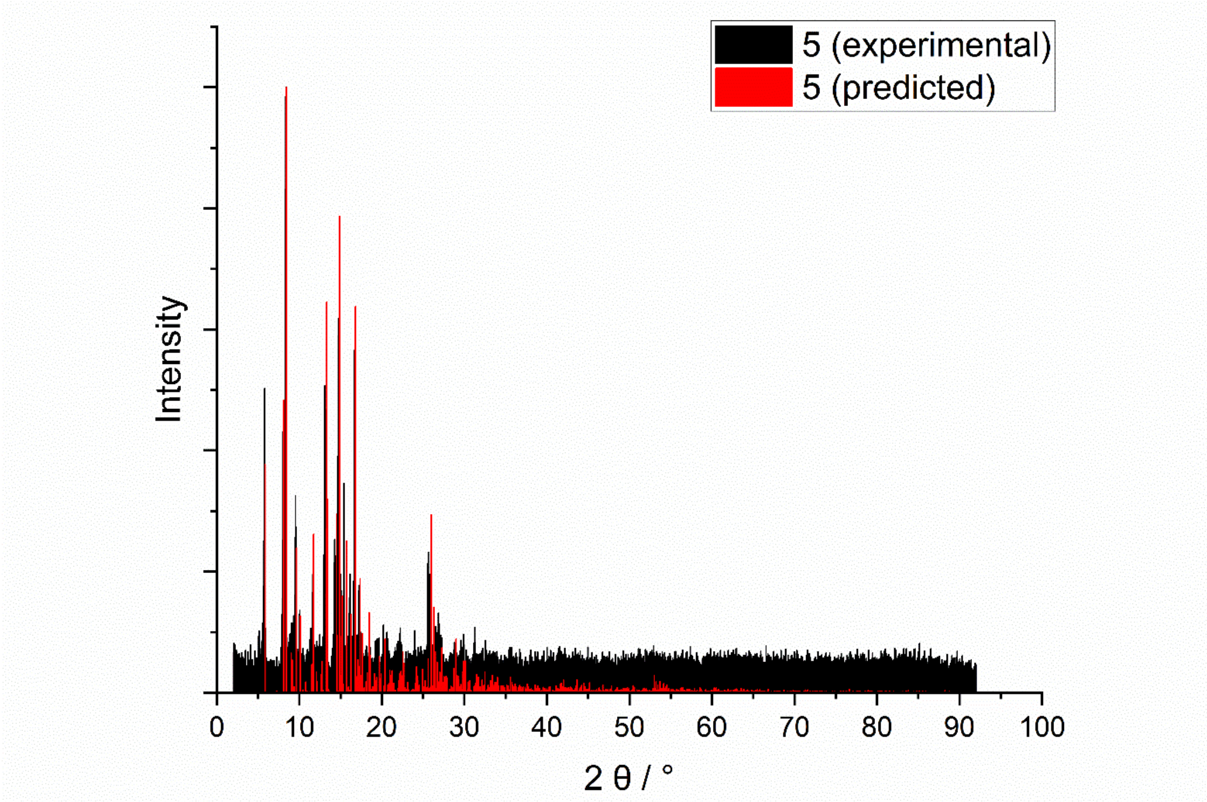

Figure S38. Experimental and calculated pXRD of **5**.

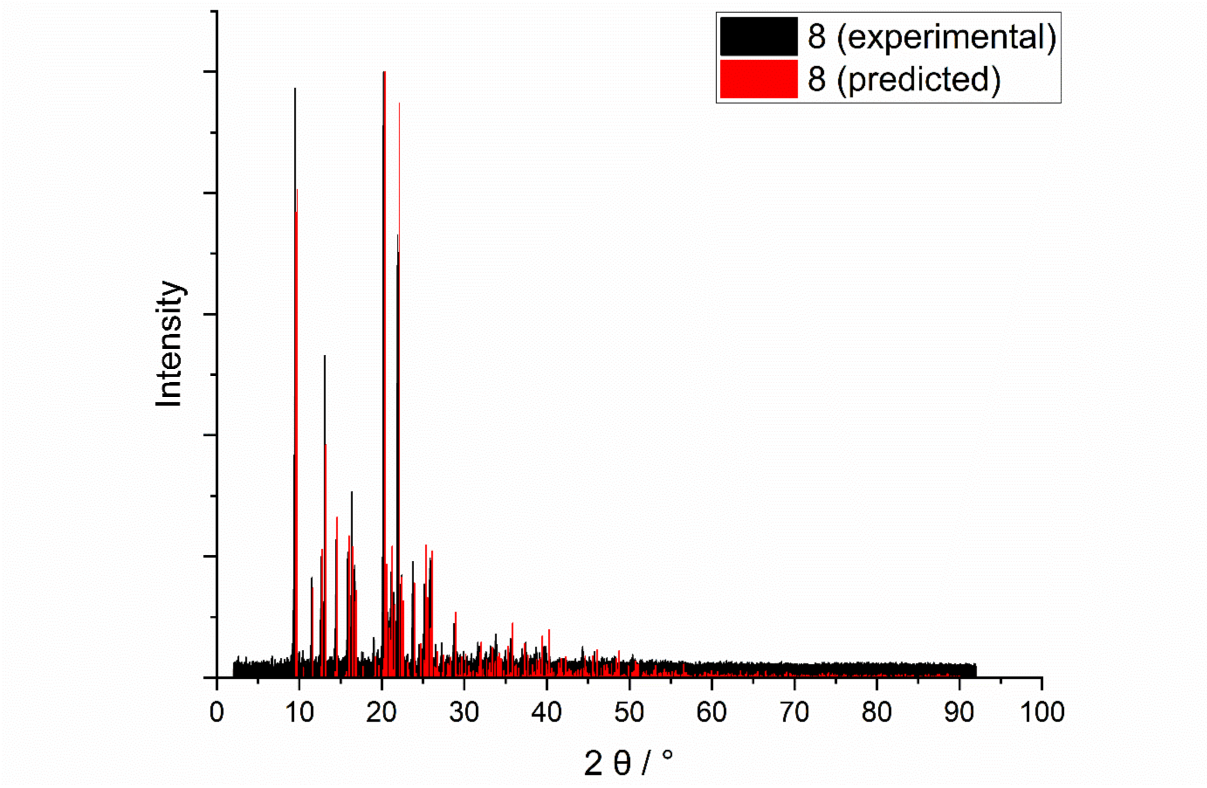

Figure S39. Experimental and calculated pXRD of **8**.

9. Computational data

9.1 Geometry optimization and vibrational frequencies

If not stated otherwise, all molecular structures investigated by computational methods were optimized by density functional theory (DFT) methods on the PBE0[21]-D3BJ[22]/def2-TZVP[23] level of theory. Typically, we are using the PBE0 functional, which was already discussed by Grimme to be near to benchmark level for geometry optimizations and single point energies of small organic molecules without heavy atoms.[24] For comparison, also the BP86,[25,26] the TPSS[27] or the B3LYP[28] density functionals were used within this work. If following investigations were conducted using the conducter-like polarizable continuum model (CPCM)[29] for THF, also the structures were optimized with solvent correction. On all obtained optimized structures numerical frequency calculations were conducted to show the absence of negative frequencies, thus the real nature of a local minimum structure. Negative frequencies below 50 cm^{-1}, that could not be removed by very tight convergence criteria, were tolerated, because the energy difference of the obtained structure to the real local minimum was expected to be negligible and imaginary force constants in this magnitude can also be just artifacts of numerical noise. An exemplary Orca input file for geometry optimizations is given below:

```
! UKS RIJCOSX pbe0 def2-TZVP D3BJ defgrid3 VeryTightSCF VeryTightOpt NumFreq Normalprint
Printbasis PrintMOs
%CPCM SMD TRUE
    SMDSOLVENT "THF"
end
%geom
maxiter 1000
end
%pal nprocs 12
end
%maxcore 3000
%scf
MaxIter 1000
end
* xyz 0 1
xyz coordinates
*
```

The xyz files of all obtained geometries and the corresponding vibrational frequencies are given in the following, labelled as “name (abbreviation) – level of theory (electronic state)”.

BPI–$AlMe_2$ (**1**) – PBE0-D3BJ/def2-TZVP (S_0)

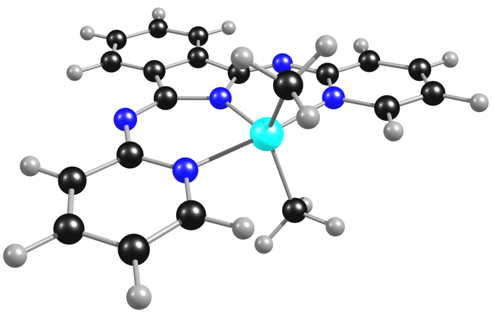

Al	2.751565000	9.709088000	5.466870000
N	3.238628000	8.282191000	4.247054000
N	0.845541000	9.696123000	4.546620000
N	5.480203000	7.699020000	4.986340000
N	1.261845000	8.090825000	2.844638000
N	4.713500000	9.560232000	6.249287000
C	6.851622000	8.599589000	6.675261000
C	5.340925000	5.658006000	2.763765000
H	6.292797000	5.572122000	3.272750000
C	2.451202000	7.780323000	3.228097000
C	7.097462000	9.528602000	7.657325000
H	8.030898000	9.510446000	8.207871000
C	5.005458000	4.849585000	1.686175000
H	5.710078000	4.104957000	1.334397000
C	4.434295000	7.589020000	4.243416000
C	2.840566000	5.917546000	1.461897000
H	1.879702000	6.029789000	0.975429000
C	5.640330000	8.633152000	5.968589000
C	3.185266000	6.716031000	2.535400000
C	3.773274000	4.978009000	1.044019000
H	3.544124000	4.331068000	0.205169000
C	4.405121000	6.589491000	3.170390000
C	0.466558000	9.017649000	3.454220000

C	3.042055000	11.582659000	4.840295000
H	4.082162000	11.916848000	4.898766000
H	2.443006000	12.320376000	5.388045000
H	2.741222000	11.675298000	3.790300000
C	-0.009208000	10.555427000	5.105689000
H	0.353585000	11.061952000	5.993105000
C	-1.270448000	10.802075000	4.613473000
H	-1.918414000	11.511315000	5.110492000
C	-0.802972000	9.221625000	2.893477000
C	2.004207000	9.177843000	7.240739000
H	0.954034000	8.872671000	7.209727000
H	2.079956000	9.969029000	7.996681000
H	2.566525000	8.327132000	7.642732000
C	4.966612000	10.463970000	7.198334000
H	4.182251000	11.193173000	7.367751000
C	6.133243000	10.494320000	7.927681000
H	6.280579000	11.251975000	8.685418000
C	-1.671135000	10.116240000	3.471320000
H	-2.654183000	10.279541000	3.045079000
H	7.565442000	7.831125000	6.412143000
H	-1.059966000	8.651241000	2.011474000

0:	0.00 cm^{-1}	14:	140.10 cm^{-1}	28:	355.64 cm^{-1}
1:	0.00 cm^{-1}	15:	141.53 cm^{-1}	29:	384.11 cm^{-1}
2:	0.00 cm^{-1}	16:	144.84 cm^{-1}	30:	433.92 cm^{-1}
3:	0.00 cm^{-1}	17:	162.80 cm^{-1}	31:	445.87 cm^{-1}
4:	0.00 cm^{-1}	18:	206.85 cm^{-1}	32:	457.13 cm^{-1}
5:	0.00 cm^{-1}	19:	220.24 cm^{-1}	33:	482.93 cm^{-1}
6:	31.59 cm^{-1}	20:	227.30 cm^{-1}	34:	530.96 cm^{-1}
7:	33.31 cm^{-1}	21:	227.85 cm^{-1}	35:	540.81 cm^{-1}
8:	53.23 cm^{-1}	22:	274.53 cm^{-1}	36:	551.04 cm^{-1}
9:	61.50 cm^{-1}	23:	280.99 cm^{-1}	37:	561.15 cm^{-1}
10:	72.69 cm^{-1}	24:	284.40 cm^{-1}	38:	582.98 cm^{-1}
11:	76.14 cm^{-1}	25:	291.67 cm^{-1}	39:	590.93 cm^{-1}
12:	107.62 cm^{-1}	26:	292.66 cm^{-1}	40:	606.48 cm^{-1}
13:	110.82 cm^{-1}	27:	318.50 cm^{-1}	41:	643.98 cm^{-1}

42:	651.22 cm^{-1}	72:	1039.80 cm^{-1}	102:	1513.57 cm^{-1}
43:	666.27 cm^{-1}	73:	1042.97 cm^{-1}	103:	1516.34 cm^{-1}
44:	667.98 cm^{-1}	74:	1083.45 cm^{-1}	104:	1523.84 cm^{-1}
45:	676.93 cm^{-1}	75:	1085.70 cm^{-1}	105:	1534.75 cm^{-1}
46:	691.45 cm^{-1}	76:	1106.60 cm^{-1}	106:	1607.07 cm^{-1}
47:	693.81 cm^{-1}	77:	1123.90 cm^{-1}	107:	1624.95 cm^{-1}
48:	703.44 cm^{-1}	78:	1132.69 cm^{-1}	108:	1653.08 cm^{-1}
49:	712.03 cm^{-1}	79:	1137.11 cm^{-1}	109:	1674.96 cm^{-1}
50:	732.37 cm^{-1}	80:	1164.71 cm^{-1}	110:	1680.79 cm^{-1}
51:	743.33 cm^{-1}	81:	1168.23 cm^{-1}	111:	1681.38 cm^{-1}
52:	769.29 cm^{-1}	82:	1172.28 cm^{-1}	112:	1687.15 cm^{-1}
53:	770.63 cm^{-1}	83:	1202.21 cm^{-1}	113:	1722.18 cm^{-1}
54:	802.39 cm^{-1}	84:	1221.52 cm^{-1}	114:	3020.56 cm^{-1}
55:	812.79 cm^{-1}	85:	1222.74 cm^{-1}	115:	3022.82 cm^{-1}
56:	820.43 cm^{-1}	86:	1224.56 cm^{-1}	116:	3084.33 cm^{-1}
57:	826.05 cm^{-1}	87:	1312.98 cm^{-1}	117:	3086.04 cm^{-1}
58:	841.63 cm^{-1}	88:	1314.08 cm^{-1}	118:	3103.61 cm^{-1}
59:	887.75 cm^{-1}	89:	1325.34 cm^{-1}	119:	3107.43 cm^{-1}
60:	902.66 cm^{-1}	90:	1329.22 cm^{-1}	120:	3190.74 cm^{-1}
61:	904.10 cm^{-1}	91:	1332.95 cm^{-1}	121:	3192.28 cm^{-1}
62:	906.02 cm^{-1}	92:	1353.53 cm^{-1}	122:	3197.64 cm^{-1}
63:	912.77 cm^{-1}	93:	1358.19 cm^{-1}	123:	3201.46 cm^{-1}
64:	926.34 cm^{-1}	94:	1378.13 cm^{-1}	124:	3205.16 cm^{-1}
65:	977.39 cm^{-1}	95:	1425.86 cm^{-1}	125:	3207.49 cm^{-1}
66:	989.91 cm^{-1}	96:	1450.65 cm^{-1}	126:	3220.35 cm^{-1}
67:	991.82 cm^{-1}	97:	1451.70 cm^{-1}	127:	3223.67 cm^{-1}
68:	993.66 cm^{-1}	98:	1455.81 cm^{-1}	128:	3231.53 cm^{-1}
69:	1007.69 cm^{-1}	99:	1460.87 cm^{-1}	129:	3232.93 cm^{-1}
70:	1008.17 cm^{-1}	100:	1481.11 cm^{-1}	130:	3236.55 cm^{-1}
71:	1039.07 cm^{-1}	101:	1482.68 cm^{-1}	131:	3239.37 cm^{-1}

This molecular structure was also optimized and the vibrational frequencies calculated using the BP86, the TPSS or the B3LYP density functional. The deviation from the results given above are negligible though. The xyz coordinates and vibrational frequencies can be found in the corresponding Orca output file within the deposited raw data.

BPI–$AlMe_2$ (**1**) – PBE0-D3BJ/def2-TZVP/CPCM(THF) (S_0)

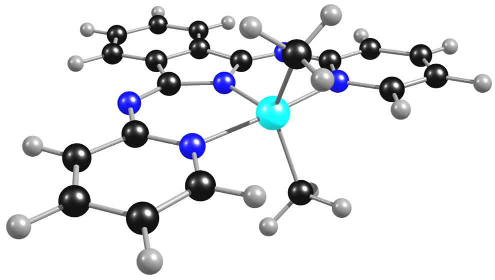

Al	2.752033000	9.704350000	5.464850000
N	3.239519000	8.281309000	4.244825000
N	0.851334000	9.714856000	4.542412000
N	5.487035000	7.721905000	4.973084000
N	1.252303000	8.079023000	2.865063000
N	4.700941000	9.553815000	6.267752000
C	6.861640000	8.628537000	6.659646000
C	5.348966000	5.667977000	2.750760000
H	6.309862000	5.583996000	3.244244000
C	2.446508000	7.771930000	3.235772000
C	7.098466000	9.546792000	7.655969000
H	8.037256000	9.538305000	8.197616000
C	5.009439000	4.851433000	1.679501000
H	5.717301000	4.111254000	1.324305000
C	4.439469000	7.598194000	4.234750000
C	2.833105000	5.901082000	1.473234000
H	1.868634000	5.995564000	0.988662000
C	5.643388000	8.650001000	5.966506000
C	3.181234000	6.708141000	2.540371000
C	3.769641000	4.966748000	1.049475000
H	3.536418000	4.314371000	0.215736000
C	4.409719000	6.594451000	3.163940000
C	0.459014000	9.017308000	3.467502000
C	3.072512000	11.573997000	4.830691000
H	4.128481000	11.865515000	4.842078000
H	2.530547000	12.326609000	5.417230000
H	2.723654000	11.685535000	3.796189000
C	0.004518000	10.591437000	5.090686000
H	0.372462000	11.120202000	5.963240000

C	-1.260226000	10.831846000	4.602949000
H	-1.900649000	11.555383000	5.089826000
C	-0.814347000	9.211333000	2.914389000
C	1.983847000	9.147837000	7.225842000
H	0.947001000	8.799667000	7.163718000
H	2.003653000	9.949608000	7.974880000
H	2.571170000	8.322600000	7.648224000
C	4.943858000	10.446814000	7.231803000
H	4.149953000	11.159661000	7.425391000
C	6.116793000	10.486584000	7.951487000
H	6.255320000	11.233941000	8.721567000
C	-1.675152000	10.122282000	3.481066000
H	-2.661745000	10.279290000	3.060545000
H	7.593411000	7.880683000	6.383690000
H	-1.088775000	8.626534000	2.046235000

0:	0.00 cm^{-1}	21:	229.61 cm^{-1}	42:	646.79 cm^{-1}
1:	0.00 cm^{-1}	22:	275.03 cm^{-1}	43:	653.89 cm^{-1}
2:	0.00 cm^{-1}	23:	279.86 cm^{-1}	44:	655.24 cm^{-1}
3:	0.00 cm^{-1}	24:	284.92 cm^{-1}	45:	673.56 cm^{-1}
4:	0.00 cm^{-1}	25:	291.72 cm^{-1}	46:	679.74 cm^{-1}
5:	0.00 cm^{-1}	26:	292.49 cm^{-1}	47:	693.51 cm^{-1}
6:	32.30 cm^{-1}	27:	320.03 cm^{-1}	48:	695.87 cm^{-1}
7:	37.32 cm^{-1}	28:	356.08 cm^{-1}	49:	711.39 cm^{-1}
8:	60.54 cm^{-1}	29:	384.85 cm^{-1}	50:	730.53 cm^{-1}
9:	72.19 cm^{-1}	30:	433.37 cm^{-1}	51:	742.83 cm^{-1}
10:	84.65 cm^{-1}	31:	448.39 cm^{-1}	52:	772.25 cm^{-1}
11:	99.39 cm^{-1}	32:	457.91 cm^{-1}	53:	772.98 cm^{-1}
12:	107.32 cm^{-1}	33:	483.08 cm^{-1}	54:	805.03 cm^{-1}
13:	112.96 cm^{-1}	34:	531.18 cm^{-1}	55:	816.68 cm^{-1}
14:	133.60 cm^{-1}	35:	532.52 cm^{-1}	56:	822.16 cm^{-1}
15:	138.25 cm^{-1}	36:	551.81 cm^{-1}	57:	827.05 cm^{-1}
16:	141.35 cm^{-1}	37:	562.02 cm^{-1}	58:	840.68 cm^{-1}
17:	162.83 cm^{-1}	38:	580.84 cm^{-1}	59:	888.35 cm^{-1}
18:	205.18 cm^{-1}	39:	587.16 cm^{-1}	60:	902.55 cm^{-1}
19:	222.43 cm^{-1}	40:	606.79 cm^{-1}	61:	909.32 cm^{-1}
20:	228.06 cm^{-1}	41:	645.90 cm^{-1}	62:	910.37 cm^{-1}

63:	915.86 cm^{-1}	86:	1215.60 cm^{-1}	109:	1669.45 cm^{-1}
64:	925.68 cm^{-1}	87:	1312.11 cm^{-1}	110:	1677.13 cm^{-1}
65:	987.15 cm^{-1}	88:	1314.62 cm^{-1}	111:	1677.64 cm^{-1}
66:	1000.07 cm^{-1}	89:	1324.77 cm^{-1}	112:	1680.36 cm^{-1}
67:	1000.74 cm^{-1}	90:	1327.31 cm^{-1}	113:	1718.69 cm^{-1}
68:	1016.71 cm^{-1}	91:	1329.89 cm^{-1}	114:	3009.88 cm^{-1}
69:	1026.26 cm^{-1}	92:	1347.59 cm^{-1}	115:	3011.11 cm^{-1}
70:	1026.88 cm^{-1}	93:	1350.96 cm^{-1}	116:	3078.31 cm^{-1}
71:	1039.96 cm^{-1}	94:	1373.30 cm^{-1}	117:	3079.52 cm^{-1}
72:	1041.29 cm^{-1}	95:	1420.05 cm^{-1}	118:	3092.81 cm^{-1}
73:	1043.68 cm^{-1}	96:	1431.82 cm^{-1}	119:	3093.20 cm^{-1}
74:	1085.61 cm^{-1}	97:	1432.77 cm^{-1}	120:	3197.62 cm^{-1}
75:	1087.44 cm^{-1}	98:	1433.96 cm^{-1}	121:	3201.04 cm^{-1}
76:	1106.14 cm^{-1}	99:	1436.46 cm^{-1}	122:	3204.75 cm^{-1}
77:	1122.53 cm^{-1}	100:	1475.18 cm^{-1}	123:	3208.44 cm^{-1}
78:	1133.17 cm^{-1}	101:	1477.24 cm^{-1}	124:	3208.57 cm^{-1}
79:	1135.87 cm^{-1}	102:	1510.77 cm^{-1}	125:	3209.20 cm^{-1}
80:	1158.28 cm^{-1}	103:	1511.71 cm^{-1}	126:	3217.28 cm^{-1}
81:	1164.13 cm^{-1}	104:	1521.79 cm^{-1}	127:	3222.00 cm^{-1}
82:	1165.29 cm^{-1}	105:	1529.82 cm^{-1}	128:	3230.59 cm^{-1}
83:	1192.49 cm^{-1}	106:	1598.37 cm^{-1}	129:	3231.05 cm^{-1}
84:	1194.46 cm^{-1}	107:	1621.99 cm^{-1}	130:	3236.27 cm^{-1}
85:	1198.68 cm^{-1}	108:	1643.59 cm^{-1}	131:	3236.94 cm^{-1}

This molecular structure was also optimized and the vibrational frequencies calculated using the BP86, the TPSS or the B3LYP density functional. The deviation from the results given above are negligible though. The xyz coordinates and vibrational frequencies can be found in the corresponding Orca output file within the deposited raw data.

BPI–AlMe ([**9**]$^{\bullet}$) – PBE0-D3BJ/def2-TZVP (D_1)

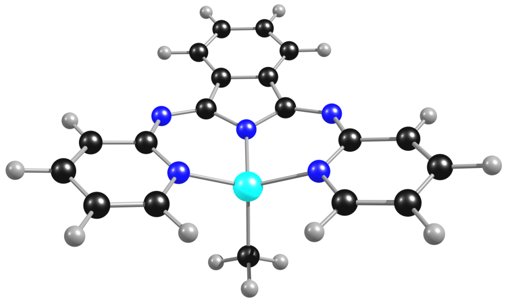

Al	3.152045000	10.041408000	4.632380000
N	3.444769000	8.443580000	3.808070000
N	1.250250000	9.807367000	4.573440000
N	5.545625000	7.905599000	4.812903000
N	1.299446000	8.170505000	2.790638000
N	4.363095000	9.613847000	6.055148000
C	6.437241000	8.690435000	6.834098000
C	5.424955000	5.654112000	2.587235000
H	6.394551000	5.591198000	3.066150000
C	2.573112000	7.849973000	2.935666000
C	6.374404000	9.446515000	7.963867000
H	7.161667000	9.384521000	8.706020000
C	5.033992000	4.746241000	1.623813000
H	5.710873000	3.953180000	1.327695000
C	4.604694000	7.723290000	3.903259000
C	2.869675000	5.813278000	1.369965000
H	1.886665000	5.871949000	0.918657000
C	5.414738000	8.733286000	5.848787000
C	3.262079000	6.745848000	2.325270000
C	3.767918000	4.825117000	1.020696000
H	3.493861000	4.091288000	0.271570000
C	4.534606000	6.666541000	2.931413000
C	0.663406000	9.029450000	3.586293000
C	3.533690000	11.866979000	4.067943000
H	4.603382000	12.021679000	3.900438000
H	3.223582000	12.602854000	4.818416000
H	3.007369000	12.121033000	3.143455000
C	0.470827000	10.601256000	5.347688000

H	0.997755000	11.195144000	6.089039000
C	-0.880517000	10.694239000	5.228448000
H	-1.442806000	11.344404000	5.883774000
C	-0.742653000	9.138060000	3.414940000
C	4.331935000	10.361826000	7.185113000
H	3.489305000	11.041621000	7.273642000
C	5.278393000	10.311652000	8.160129000
H	5.188950000	10.932945000	9.040090000
C	-1.501266000	9.936877000	4.213929000
H	-2.574041000	9.990842000	4.070208000
H	7.255051000	8.009240000	6.641947000
H	-1.174048000	8.535215000	2.627383000

0:	0.00 cm^{-1}	24:	352.49 cm^{-1}	48:	776.25 cm^{-1}
1:	0.00 cm^{-1}	25:	398.53 cm^{-1}	49:	776.94 cm^{-1}
2:	0.00 cm^{-1}	26:	417.99 cm^{-1}	50:	786.28 cm^{-1}
3:	0.00 cm^{-1}	27:	436.94 cm^{-1}	51:	799.98 cm^{-1}
4:	0.00 cm^{-1}	28:	444.92 cm^{-1}	52:	853.42 cm^{-1}
5:	0.00 cm^{-1}	29:	448.32 cm^{-1}	53:	866.58 cm^{-1}
6:	42.07 cm^{-1}	30:	465.54 cm^{-1}	54:	873.12 cm^{-1}
7:	45.09 cm^{-1}	31:	530.37 cm^{-1}	55:	874.22 cm^{-1}
8:	51.04 cm^{-1}	32:	539.35 cm^{-1}	56:	888.49 cm^{-1}
9:	80.71 cm^{-1}	33:	546.58 cm^{-1}	57:	915.56 cm^{-1}
10:	82.49 cm^{-1}	34:	580.60 cm^{-1}	58:	916.64 cm^{-1}
11:	104.98 cm^{-1}	35:	605.74 cm^{-1}	59:	944.79 cm^{-1}
12:	120.85 cm^{-1}	36:	638.50 cm^{-1}	60:	950.05 cm^{-1}
13:	127.34 cm^{-1}	37:	643.57 cm^{-1}	61:	963.50 cm^{-1}
14:	143.96 cm^{-1}	38:	654.74 cm^{-1}	62:	978.05 cm^{-1}
15:	164.80 cm^{-1}	39:	667.63 cm^{-1}	63:	1002.35 cm^{-1}
16:	175.05 cm^{-1}	40:	670.43 cm^{-1}	64:	1002.74 cm^{-1}
17:	222.87 cm^{-1}	41:	689.96 cm^{-1}	65:	1034.22 cm^{-1}
18:	236.14 cm^{-1}	42:	692.53 cm^{-1}	66:	1039.96 cm^{-1}
19:	268.05 cm^{-1}	43:	704.13 cm^{-1}	67:	1046.62 cm^{-1}
20:	269.73 cm^{-1}	44:	721.01 cm^{-1}	68:	1057.69 cm^{-1}
21:	289.77 cm^{-1}	45:	745.42 cm^{-1}	69:	1060.59 cm^{-1}
22:	319.23 cm^{-1}	46:	752.01 cm^{-1}	70:	1106.61 cm^{-1}
23:	348.26 cm^{-1}	47:	755.13 cm^{-1}	71:	1144.27 cm^{-1}

72: 1145.56 cm^{-1}	88: 1415.41 cm^{-1}	104: 1683.54 cm^{-1}
73: 1150.19 cm^{-1}	89: 1441.40 cm^{-1}	105: 3034.12 cm^{-1}
74: 1164.20 cm^{-1}	90: 1445.30 cm^{-1}	106: 3104.54 cm^{-1}
75: 1170.56 cm^{-1}	91: 1472.56 cm^{-1}	107: 3125.61 cm^{-1}
76: 1172.28 cm^{-1}	92: 1477.69 cm^{-1}	108: 3167.97 cm^{-1}
77: 1216.97 cm^{-1}	93: 1498.19 cm^{-1}	109: 3171.82 cm^{-1}
78: 1226.05 cm^{-1}	94: 1507.92 cm^{-1}	110: 3190.70 cm^{-1}
79: 1233.74 cm^{-1}	95: 1524.06 cm^{-1}	111: 3201.90 cm^{-1}
80: 1286.52 cm^{-1}	96: 1533.29 cm^{-1}	112: 3202.79 cm^{-1}
81: 1289.01 cm^{-1}	97: 1546.37 cm^{-1}	113: 3203.92 cm^{-1}
82: 1313.04 cm^{-1}	98: 1577.81 cm^{-1}	114: 3215.03 cm^{-1}
83: 1317.52 cm^{-1}	99: 1585.74 cm^{-1}	115: 3220.61 cm^{-1}
84: 1330.46 cm^{-1}	100: 1597.47 cm^{-1}	116: 3234.36 cm^{-1}
85: 1385.21 cm^{-1}	101: 1631.78 cm^{-1}	117: 3236.45 cm^{-1}
86: 1390.10 cm^{-1}	102: 1663.18 cm^{-1}	118: 3238.14 cm^{-1}
87: 1402.43 cm^{-1}	103: 1681.65 cm^{-1}	119: 3239.98 cm^{-1}

This molecular structure was also optimized and the vibrational frequencies calculated using the BP86, the TPSS or the B3LYP density functional. The deviation from the results given above are negligible though. The xyz coordinates and vibrational frequencies can be found in the corresponding Orca output file within the deposited raw data.

BPI–AlMe ([**9**]$^{\bullet}$) – PBE0-D3BJ/def2-TZVP/CPCM(THF) (D_1)

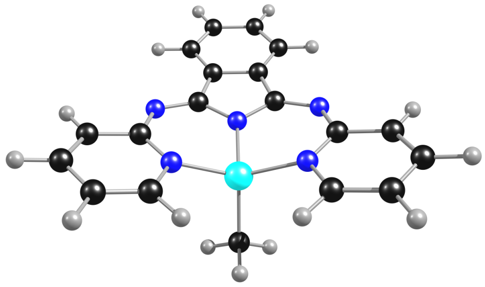

Al	2.551921000	8.853059000	5.818130000
N	3.153837000	7.862006000	4.398935000
N	1.174014000	9.663523000	4.741850000
N	5.444888000	7.659746000	5.057287000
N	1.180214000	7.939047000	3.049655000
N	4.332250000	9.457008000	6.225257000
C	6.678289000	9.055023000	6.491500000
C	5.380599000	5.559595000	2.680530000
H	6.363756000	5.501546000	3.133936000
C	2.368179000	7.446522000	3.360667000
C	6.790041000	10.138758000	7.311962000
H	7.750649000	10.399247000	7.741672000
C	5.065634000	4.810350000	1.564126000
H	5.808712000	4.147547000	1.135068000
C	4.403318000	7.313499000	4.318069000
C	2.820161000	5.728657000	1.475377000
H	1.844888000	5.800518000	1.007137000
C	5.438027000	8.698683000	5.902474000
C	3.120746000	6.475918000	2.612652000
C	3.795554000	4.894267000	0.966199000
H	3.584388000	4.294658000	0.087878000
C	4.398289000	6.392098000	3.213808000
C	0.645651000	9.012328000	3.646551000
C	0.571227000	10.782839000	5.210409000
H	1.042139000	11.238442000	6.075530000
C	-0.556112000	11.321563000	4.666383000
H	-0.986101000	12.221335000	5.084716000
C	-0.549906000	9.527657000	3.082944000

C	1.732479000	8.245673000	7.477085000
H	0.810818000	7.686219000	7.284514000
H	1.469676000	9.083716000	8.132550000
H	2.408826000	7.594859000	8.041389000
C	4.466521000	10.527119000	7.044418000
H	3.549921000	11.069052000	7.254599000
C	5.651512000	10.915053000	7.594580000
H	5.695387000	11.782821000	8.238450000
C	-1.137118000	10.657472000	3.571123000
H	-2.043557000	11.040355000	3.116185000
H	7.532905000	8.438523000	6.244096000
H	-0.961264000	8.993499000	2.236151000

0:	0.00 cm^{-1}	24:	353.84 cm^{-1}	48:	780.79 cm^{-1}
1:	0.00 cm^{-1}	25:	396.18 cm^{-1}	49:	786.05 cm^{-1}
2:	0.00 cm^{-1}	26:	424.29 cm^{-1}	50:	790.02 cm^{-1}
3:	0.00 cm^{-1}	27:	437.33 cm^{-1}	51:	802.11 cm^{-1}
4:	0.00 cm^{-1}	28:	446.26 cm^{-1}	52:	849.81 cm^{-1}
5:	0.00 cm^{-1}	29:	447.54 cm^{-1}	53:	870.37 cm^{-1}
6:	47.68 cm^{-1}	30:	464.55 cm^{-1}	54:	881.67 cm^{-1}
7:	51.00 cm^{-1}	31:	532.08 cm^{-1}	55:	882.10 cm^{-1}
8:	51.52 cm^{-1}	32:	541.65 cm^{-1}	56:	890.25 cm^{-1}
9:	84.11 cm^{-1}	33:	549.80 cm^{-1}	57:	912.05 cm^{-1}
10:	87.49 cm^{-1}	34:	580.88 cm^{-1}	58:	917.73 cm^{-1}
11:	117.79 cm^{-1}	35:	606.89 cm^{-1}	59:	973.31 cm^{-1}
12:	122.62 cm^{-1}	36:	621.61 cm^{-1}	60:	977.34 cm^{-1}
13:	130.84 cm^{-1}	37:	644.77 cm^{-1}	61:	979.16 cm^{-1}
14:	147.67 cm^{-1}	38:	648.58 cm^{-1}	62:	997.87 cm^{-1}
15:	163.15 cm^{-1}	39:	663.51 cm^{-1}	63:	1014.20 cm^{-1}
16:	179.27 cm^{-1}	40:	668.64 cm^{-1}	64:	1014.66 cm^{-1}
17:	227.12 cm^{-1}	41:	682.46 cm^{-1}	65:	1033.25 cm^{-1}
18:	236.09 cm^{-1}	42:	689.24 cm^{-1}	66:	1034.36 cm^{-1}
19:	267.10 cm^{-1}	43:	703.06 cm^{-1}	67:	1044.96 cm^{-1}
20:	268.64 cm^{-1}	44:	720.77 cm^{-1}	68:	1065.75 cm^{-1}
21:	290.46 cm^{-1}	45:	745.74 cm^{-1}	69:	1066.47 cm^{-1}
22:	317.58 cm^{-1}	46:	756.95 cm^{-1}	70:	1104.62 cm^{-1}
23:	349.89 cm^{-1}	47:	759.14 cm^{-1}	71:	1139.60 cm^{-1}

72:	1140.37 cm^{-1}	88:	1412.27 cm^{-1}	104:	1680.02 cm^{-1}
73:	1145.10 cm^{-1}	89:	1420.28 cm^{-1}	105:	3028.14 cm^{-1}
74:	1152.12 cm^{-1}	90:	1423.03 cm^{-1}	106:	3104.14 cm^{-1}
75:	1160.70 cm^{-1}	91:	1465.78 cm^{-1}	107:	3112.40 cm^{-1}
76:	1162.34 cm^{-1}	92:	1467.57 cm^{-1}	108:	3188.60 cm^{-1}
77:	1199.29 cm^{-1}	93:	1491.30 cm^{-1}	109:	3189.47 cm^{-1}
78:	1210.10 cm^{-1}	94:	1495.94 cm^{-1}	110:	3189.68 cm^{-1}
79:	1231.11 cm^{-1}	95:	1517.23 cm^{-1}	111:	3198.75 cm^{-1}
80:	1293.53 cm^{-1}	96:	1521.48 cm^{-1}	112:	3203.77 cm^{-1}
81:	1297.03 cm^{-1}	97:	1540.08 cm^{-1}	113:	3205.16 cm^{-1}
82:	1311.87 cm^{-1}	98:	1579.51 cm^{-1}	114:	3207.84 cm^{-1}
83:	1317.33 cm^{-1}	99:	1588.87 cm^{-1}	115:	3214.47 cm^{-1}
84:	1327.64 cm^{-1}	100:	1599.06 cm^{-1}	116:	3226.93 cm^{-1}
85:	1377.60 cm^{-1}	101:	1623.90 cm^{-1}	117:	3227.96 cm^{-1}
86:	1381.32 cm^{-1}	102:	1655.51 cm^{-1}	118:	3238.18 cm^{-1}
87:	1400.04 cm^{-1}	103:	1679.09 cm^{-1}	119:	3238.46 cm^{-1}

This molecular structure was also optimized and the vibrational frequencies calculated using the BP86, the TPSS or the B3LYP density functional. The deviation from the results given above are negligible though. The xyz coordinates and vibrational frequencies can be found in the corresponding Orca output file within the deposited raw data.

[BPI–AlMe]$^+$ (**9**)$^+$ – PBE0-D3BJ/def2-TZVP (S_0)

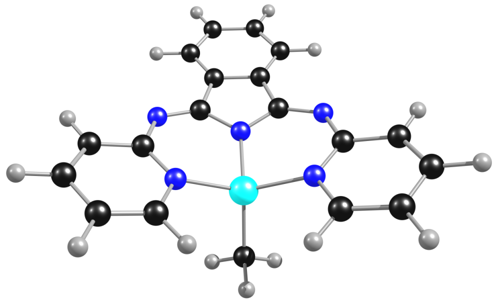

Al	2.515953000	8.770485000	5.877052000
N	3.135740000	7.842371000	4.432503000
N	1.148580000	9.617468000	4.787849000
N	5.429359000	7.678466000	5.002898000
N	1.231976000	7.951365000	3.026486000
N	4.305848000	9.412287000	6.274545000
C	6.673374000	9.141603000	6.348882000
C	5.373249000	5.543339000	2.697940000
H	6.354356000	5.496682000	3.153351000
C	2.368775000	7.449117000	3.363684000
C	6.788392000	10.217710000	7.193791000
H	7.762565000	10.526959000	7.552919000
C	5.049528000	4.778627000	1.583041000
H	5.789776000	4.109465000	1.161692000
C	4.399313000	7.317102000	4.319764000
C	2.811565000	5.709770000	1.491776000
H	1.837292000	5.790151000	1.026498000
C	5.420617000	8.735423000	5.869900000
C	3.127899000	6.447830000	2.614399000
C	3.791349000	4.860364000	0.990641000
H	3.576656000	4.253237000	0.119674000
C	4.389368000	6.365856000	3.208350000
C	0.683631000	9.043417000	3.639440000
C	0.530548000	10.710422000	5.279773000
H	0.925540000	11.101900000	6.211534000
C	-0.545578000	11.303562000	4.676987000
H	-0.997409000	12.182153000	5.116141000
C	-0.432296000	9.603684000	3.003326000

C	1.695500000	8.246113000	7.547060000
H	0.760382000	7.704203000	7.381066000
H	1.451353000	9.102224000	8.185006000
H	2.355790000	7.599793000	8.131843000
C	4.434334000	10.456816000	7.117736000
H	3.510791000	10.934190000	7.428614000
C	5.639883000	10.901540000	7.589027000
H	5.681177000	11.748185000	8.260159000
C	-1.039782000	10.726684000	3.508286000
H	-1.898036000	11.155080000	3.004796000
H	7.532742000	8.576788000	6.015342000
H	-0.778738000	9.117366000	2.102072000

0:	0.00 cm^{-1}	24:	353.03 cm^{-1}	48:	806.33 cm^{-1}
1:	0.00 cm^{-1}	25:	394.90 cm^{-1}	49:	810.03 cm^{-1}
2:	0.00 cm^{-1}	26:	424.99 cm^{-1}	50:	819.71 cm^{-1}
3:	0.00 cm^{-1}	27:	430.46 cm^{-1}	51:	821.05 cm^{-1}
4:	0.00 cm^{-1}	28:	446.19 cm^{-1}	52:	864.06 cm^{-1}
5:	0.00 cm^{-1}	29:	450.00 cm^{-1}	53:	875.91 cm^{-1}
6:	41.92 cm^{-1}	30:	477.26 cm^{-1}	54:	913.68 cm^{-1}
7:	48.69 cm^{-1}	31:	527.39 cm^{-1}	55:	914.77 cm^{-1}
8:	53.25 cm^{-1}	32:	549.92 cm^{-1}	56:	917.79 cm^{-1}
9:	80.00 cm^{-1}	33:	556.10 cm^{-1}	57:	921.21 cm^{-1}
10:	83.74 cm^{-1}	34:	580.94 cm^{-1}	58:	927.18 cm^{-1}
11:	112.42 cm^{-1}	35:	605.44 cm^{-1}	59:	981.96 cm^{-1}
12:	120.08 cm^{-1}	36:	646.32 cm^{-1}	60:	985.23 cm^{-1}
13:	128.17 cm^{-1}	37:	662.03 cm^{-1}	61:	990.47 cm^{-1}
14:	145.65 cm^{-1}	38:	671.61 cm^{-1}	62:	1023.41 cm^{-1}
15:	165.78 cm^{-1}	39:	672.85 cm^{-1}	63:	1038.82 cm^{-1}
16:	172.70 cm^{-1}	40:	688.27 cm^{-1}	64:	1039.01 cm^{-1}
17:	213.48 cm^{-1}	41:	695.34 cm^{-1}	65:	1045.33 cm^{-1}
18:	226.06 cm^{-1}	42:	699.55 cm^{-1}	66:	1051.80 cm^{-1}
19:	278.06 cm^{-1}	43:	709.66 cm^{-1}	67:	1056.62 cm^{-1}
20:	280.77 cm^{-1}	44:	738.90 cm^{-1}	68:	1083.06 cm^{-1}
21:	293.27 cm^{-1}	45:	757.33 cm^{-1}	69:	1085.75 cm^{-1}
22:	303.00 cm^{-1}	46:	773.00 cm^{-1}	70:	1110.29 cm^{-1}
23:	346.83 cm^{-1}	47:	774.15 cm^{-1}	71:	1141.10 cm^{-1}

72: 1141.33 cm^{-1}
73: 1157.75 cm^{-1}
74: 1178.09 cm^{-1}
75: 1183.66 cm^{-1}
76: 1184.56 cm^{-1}
77: 1205.42 cm^{-1}
78: 1239.35 cm^{-1}
79: 1241.14 cm^{-1}
80: 1307.83 cm^{-1}
81: 1311.14 cm^{-1}
82: 1313.52 cm^{-1}
83: 1319.07 cm^{-1}
84: 1331.48 cm^{-1}
85: 1347.95 cm^{-1}
86: 1351.35 cm^{-1}
87: 1395.09 cm^{-1}
88: 1436.78 cm^{-1}
89: 1438.33 cm^{-1}
90: 1440.81 cm^{-1}
91: 1483.07 cm^{-1}
92: 1484.29 cm^{-1}
93: 1503.24 cm^{-1}
94: 1507.13 cm^{-1}
95: 1519.97 cm^{-1}
96: 1524.08 cm^{-1}
97: 1590.64 cm^{-1}
98: 1605.33 cm^{-1}
99: 1653.10 cm^{-1}
100: 1664.28 cm^{-1}
101: 1675.45 cm^{-1}
102: 1683.93 cm^{-1}
103: 1691.17 cm^{-1}
104: 1716.96 cm^{-1}
105: 3040.20 cm^{-1}
106: 3112.26 cm^{-1}
107: 3130.50 cm^{-1}
108: 3186.97 cm^{-1}
109: 3189.36 cm^{-1}
110: 3207.73 cm^{-1}
111: 3214.90 cm^{-1}
112: 3217.11 cm^{-1}
113: 3220.99 cm^{-1}
114: 3228.98 cm^{-1}
115: 3232.04 cm^{-1}
116: 3241.87 cm^{-1}
117: 3246.21 cm^{-1}
118: 3247.49 cm^{-1}
119: 3250.66 cm^{-1}

This molecular structure was also optimized and the vibrational frequencies calculated using the BP86, the TPSS or the B3LYP density functional. The deviation from the results given above are negligible though. The xyz coordinates and vibrational frequencies can be found in the corresponding Orca output file within the deposited raw data.

[BPI–AlMe]+ (**9**)+ – PBE0-D3BJ/def2-TZVP/CPCM(THF) (S_0)

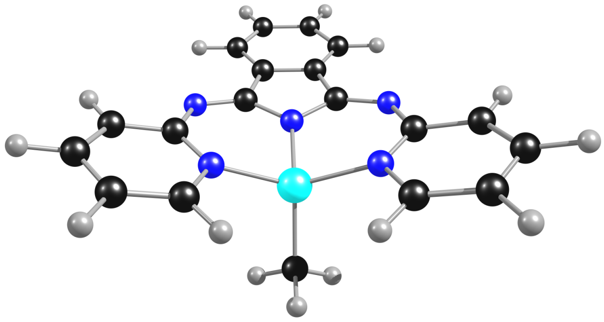

Al	2.526676000	8.818257000	5.868672000
N	3.150107000	7.890891000	4.410905000
N	1.133391000	9.635047000	4.777833000
N	5.433033000	7.683928000	5.010330000
N	1.226086000	7.959365000	3.030477000
N	4.327643000	9.428898000	6.273904000
C	6.685867000	9.122535000	6.383199000
C	5.366633000	5.547821000	2.707333000
H	6.348828000	5.494627000	3.161310000
C	2.374059000	7.479951000	3.361907000
C	6.802821000	10.201347000	7.227763000
H	7.775931000	10.495514000	7.602737000
C	5.038864000	4.773407000	1.600044000
H	5.775559000	4.095165000	1.185722000
C	4.402085000	7.347104000	4.316234000
C	2.806928000	5.717266000	1.502297000
H	1.832444000	5.794127000	1.035241000
C	5.435002000	8.739064000	5.887734000
C	3.129256000	6.467785000	2.615589000
C	3.780492000	4.856786000	1.007510000
H	3.560205000	4.242052000	0.142646000
C	4.389498000	6.384824000	3.208755000
C	0.662132000	9.050556000	3.642595000
C	0.521210000	10.732301000	5.262493000
H	0.936474000	11.142063000	6.176453000
C	-0.568589000	11.309693000	4.663280000
H	-1.016515000	12.193941000	5.095742000
C	-0.464057000	9.590131000	3.011605000

C	1.715957000	8.200883000	7.514728000
H	0.798406000	7.638402000	7.314378000
H	1.450035000	9.031918000	8.176680000
H	2.398346000	7.548656000	8.069060000
C	4.453292000	10.479814000	7.105506000
H	3.529406000	10.975558000	7.382290000
C	5.661058000	10.906643000	7.594940000
H	5.704199000	11.760171000	8.257712000
C	-1.075161000	10.715515000	3.511958000
H	-1.942331000	11.131497000	3.012954000
H	7.546995000	8.548871000	6.068016000
H	-0.820899000	9.097423000	2.117310000

0:	0.00 cm^{-1}	23:	349.51 cm^{-1}	46:	773.33 cm^{-1}
1:	0.00 cm^{-1}	24:	354.51 cm^{-1}	47:	774.21 cm^{-1}
2:	0.00 cm^{-1}	25:	393.42 cm^{-1}	48:	805.33 cm^{-1}
3:	0.00 cm^{-1}	26:	429.15 cm^{-1}	49:	811.86 cm^{-1}
4:	0.00 cm^{-1}	27:	431.53 cm^{-1}	50:	818.41 cm^{-1}
5:	0.00 cm^{-1}	28:	449.23 cm^{-1}	51:	822.96 cm^{-1}
6:	44.45 cm^{-1}	29:	450.06 cm^{-1}	52:	861.35 cm^{-1}
7:	53.93 cm^{-1}	30:	480.54 cm^{-1}	53:	877.13 cm^{-1}
8:	55.03 cm^{-1}	31:	528.10 cm^{-1}	54:	908.41 cm^{-1}
9:	82.05 cm^{-1}	32:	549.71 cm^{-1}	55:	915.31 cm^{-1}
10:	87.07 cm^{-1}	33:	559.17 cm^{-1}	56:	917.75 cm^{-1}
11:	112.83 cm^{-1}	34:	581.55 cm^{-1}	57:	918.78 cm^{-1}
12:	118.18 cm^{-1}	35:	608.80 cm^{-1}	58:	929.48 cm^{-1}
13:	128.13 cm^{-1}	36:	626.94 cm^{-1}	59:	991.06 cm^{-1}
14:	147.17 cm^{-1}	37:	657.59 cm^{-1}	60:	996.16 cm^{-1}
15:	164.34 cm^{-1}	38:	668.78 cm^{-1}	61:	1000.25 cm^{-1}
16:	172.69 cm^{-1}	39:	669.93 cm^{-1}	62:	1026.19 cm^{-1}
17:	214.97 cm^{-1}	40:	687.11 cm^{-1}	63:	1038.99 cm^{-1}
18:	231.68 cm^{-1}	41:	690.73 cm^{-1}	64:	1039.67 cm^{-1}
19:	271.23 cm^{-1}	42:	692.40 cm^{-1}	65:	1043.43 cm^{-1}
20:	283.48 cm^{-1}	43:	710.79 cm^{-1}	66:	1057.28 cm^{-1}
21:	296.79 cm^{-1}	44:	736.96 cm^{-1}	67:	1059.08 cm^{-1}
22:	305.88 cm^{-1}	45:	754.00 cm^{-1}	68:	1089.37 cm^{-1}

69: 1091.03 cm^{-1}	86: 1348.22 cm^{-1}	103: 1686.75 cm^{-1}
70: 1108.10 cm^{-1}	87: 1391.10 cm^{-1}	104: 1714.50 cm^{-1}
71: 1137.08 cm^{-1}	88: 1418.19 cm^{-1}	105: 3034.01 cm^{-1}
72: 1137.52 cm^{-1}	89: 1419.37 cm^{-1}	106: 3112.19 cm^{-1}
73: 1157.51 cm^{-1}	90: 1431.64 cm^{-1}	107: 3119.90 cm^{-1}
74: 1164.55 cm^{-1}	91: 1477.06 cm^{-1}	108: 3201.93 cm^{-1}
75: 1170.04 cm^{-1}	92: 1477.59 cm^{-1}	109: 3203.04 cm^{-1}
76: 1170.64 cm^{-1}	93: 1502.86 cm^{-1}	110: 3207.07 cm^{-1}
77: 1201.36 cm^{-1}	94: 1507.08 cm^{-1}	111: 3212.53 cm^{-1}
78: 1205.19 cm^{-1}	95: 1513.51 cm^{-1}	112: 3215.85 cm^{-1}
79: 1234.41 cm^{-1}	96: 1521.03 cm^{-1}	113: 3218.28 cm^{-1}
80: 1308.24 cm^{-1}	97: 1592.00 cm^{-1}	114: 3221.29 cm^{-1}
81: 1312.58 cm^{-1}	98: 1608.48 cm^{-1}	115: 3225.53 cm^{-1}
82: 1319.08 cm^{-1}	99: 1645.27 cm^{-1}	116: 3237.54 cm^{-1}
83: 1328.06 cm^{-1}	100: 1663.50 cm^{-1}	117: 3238.69 cm^{-1}
84: 1336.89 cm^{-1}	101: 1676.65 cm^{-1}	118: 3243.80 cm^{-1}
85: 1338.36 cm^{-1}	102: 1678.04 cm^{-1}	119: 3244.65 cm^{-1}

This molecular structure was also optimized and the vibrational frequencies calculated using the BP86, the TPSS or the B3LYP density functional. The deviation from the results given above are negligible though. The xyz coordinates and vibrational frequencies can be found in the corresponding Orca output file within the deposited raw data.

[BPI–AlMe$_2$]$^-$ ([**1**]$^{\bullet-}$) – PBE0-D3BJ/def2-TZVP (D_1)

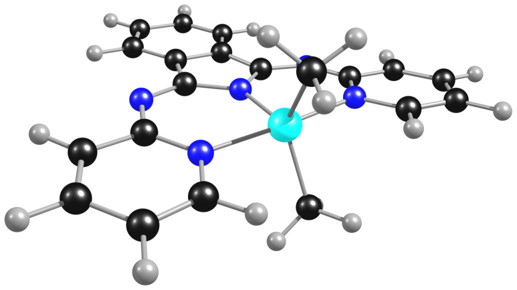

Al	2.760933000	9.682187000	5.443935000
N	3.246277000	8.260398000	4.228227000
N	0.862269000	9.679007000	4.556071000
N	5.489992000	7.666407000	5.033467000
N	1.250787000	8.128896000	2.801314000
N	4.702459000	9.561123000	6.226305000
C	6.842463000	8.598327000	6.717162000
C	5.333769000	5.630670000	2.770412000
H	6.279149000	5.530962000	3.291226000
C	2.462717000	7.776841000	3.196291000
C	7.080275000	9.547789000	7.667353000
H	8.007650000	9.537892000	8.231194000
C	5.008239000	4.833813000	1.691365000
H	5.709670000	4.081873000	1.344752000
C	4.435825000	7.555207000	4.242872000
C	2.854412000	5.924186000	1.437556000
H	1.900549000	6.048765000	0.937994000
C	5.633198000	8.602850000	5.968812000
C	3.180182000	6.729678000	2.526068000
C	3.774352000	4.980297000	1.027591000
H	3.548786000	4.338615000	0.182058000
C	4.407430000	6.583884000	3.186356000
C	0.484298000	9.018699000	3.428227000
C	3.026449000	11.582272000	4.829583000
H	4.041154000	11.970062000	4.964584000
H	2.341607000	12.294236000	5.307923000
H	2.804745000	11.632587000	3.756152000
C	-0.000289000	10.519497000	5.137323000

H	0.366428000	10.998675000	6.039649000
C	-1.260851000	10.788390000	4.667715000
H	-1.904506000	11.482377000	5.191752000
C	-0.807132000	9.264880000	2.886048000
C	2.021244000	9.174159000	7.247758000
H	0.950627000	8.946146000	7.255507000
H	2.195255000	9.935205000	8.019358000
H	2.542992000	8.273855000	7.595631000
C	4.961914000	10.486059000	7.156561000
H	4.179041000	11.224696000	7.297216000
C	6.112692000	10.538999000	7.901405000
H	6.253015000	11.318257000	8.638693000
C	-1.666025000	10.134010000	3.492480000
H	-2.650887000	10.312894000	3.072859000
H	7.555264000	7.816528000	6.488619000
H	-1.068908000	8.726550000	1.984272000

0:	0.00 cm^{-1}	20:	224.84 cm^{-1}	40:	600.55 cm^{-1}
1:	0.00 cm^{-1}	21:	240.96 cm^{-1}	41:	638.43 cm^{-1}
2:	0.00 cm^{-1}	22:	272.10 cm^{-1}	42:	639.45 cm^{-1}
3:	0.00 cm^{-1}	23:	275.16 cm^{-1}	43:	644.87 cm^{-1}
4:	0.00 cm^{-1}	24:	283.10 cm^{-1}	44:	650.33 cm^{-1}
5:	0.00 cm^{-1}	25:	285.98 cm^{-1}	45:	660.18 cm^{-1}
6:	35.19 cm^{-1}	26:	300.44 cm^{-1}	46:	681.63 cm^{-1}
7:	36.98 cm^{-1}	27:	329.44 cm^{-1}	47:	686.02 cm^{-1}
8:	56.43 cm^{-1}	28:	361.75 cm^{-1}	48:	701.47 cm^{-1}
9:	59.90 cm^{-1}	29:	385.02 cm^{-1}	49:	706.49 cm^{-1}
10:	71.52 cm^{-1}	30:	437.02 cm^{-1}	50:	718.11 cm^{-1}
11:	84.04 cm^{-1}	31:	439.17 cm^{-1}	51:	728.34 cm^{-1}
12:	108.99 cm^{-1}	32:	456.39 cm^{-1}	52:	746.87 cm^{-1}
13:	121.61 cm^{-1}	33:	457.11 cm^{-1}	53:	747.25 cm^{-1}
14:	138.54 cm^{-1}	34:	526.03 cm^{-1}	54:	761.77 cm^{-1}
15:	144.53 cm^{-1}	35:	535.80 cm^{-1}	55:	765.73 cm^{-1}
16:	154.15 cm^{-1}	36:	536.45 cm^{-1}	56:	776.61 cm^{-1}
17:	157.51 cm^{-1}	37:	544.42 cm^{-1}	57:	789.71 cm^{-1}
18:	211.59 cm^{-1}	38:	578.53 cm^{-1}	58:	838.21 cm^{-1}
19:	223.63 cm^{-1}	39:	581.49 cm^{-1}	59:	853.35 cm^{-1}

60:	854.33 cm^{-1}	84:	1201.21 cm^{-1}	108:	1593.32 cm^{-1}
61:	865.23 cm^{-1}	85:	1205.37 cm^{-1}	109:	1613.48 cm^{-1}
62:	884.56 cm^{-1}	86:	1212.72 cm^{-1}	110:	1633.81 cm^{-1}
63:	902.48 cm^{-1}	87:	1289.25 cm^{-1}	111:	1646.91 cm^{-1}
64:	915.33 cm^{-1}	88:	1295.89 cm^{-1}	112:	1669.32 cm^{-1}
65:	939.23 cm^{-1}	89:	1315.24 cm^{-1}	113:	1670.46 cm^{-1}
66:	940.32 cm^{-1}	90:	1336.24 cm^{-1}	114:	3007.96 cm^{-1}
67:	961.99 cm^{-1}	91:	1338.36 cm^{-1}	115:	3010.03 cm^{-1}
68:	963.02 cm^{-1}	92:	1375.64 cm^{-1}	116:	3071.54 cm^{-1}
69:	971.84 cm^{-1}	93:	1386.38 cm^{-1}	117:	3072.80 cm^{-1}
70:	972.70 cm^{-1}	94:	1390.46 cm^{-1}	118:	3093.68 cm^{-1}
71:	1020.60 cm^{-1}	95:	1422.21 cm^{-1}	119:	3094.96 cm^{-1}
72:	1025.64 cm^{-1}	96:	1448.50 cm^{-1}	120:	3167.75 cm^{-1}
73:	1029.17 cm^{-1}	97:	1449.38 cm^{-1}	121:	3174.46 cm^{-1}
74:	1061.04 cm^{-1}	98:	1454.37 cm^{-1}	122:	3175.94 cm^{-1}
75:	1062.96 cm^{-1}	99:	1459.74 cm^{-1}	123:	3181.23 cm^{-1}
76:	1077.12 cm^{-1}	100:	1469.97 cm^{-1}	124:	3182.37 cm^{-1}
77:	1115.22 cm^{-1}	101:	1475.70 cm^{-1}	125:	3185.04 cm^{-1}
78:	1132.55 cm^{-1}	102:	1500.22 cm^{-1}	126:	3204.21 cm^{-1}
79:	1133.70 cm^{-1}	103:	1512.28 cm^{-1}	127:	3208.35 cm^{-1}
80:	1149.94 cm^{-1}	104:	1528.83 cm^{-1}	128:	3217.67 cm^{-1}
81:	1157.75 cm^{-1}	105:	1540.43 cm^{-1}	129:	3220.69 cm^{-1}
82:	1159.92 cm^{-1}	106:	1558.24 cm^{-1}	130:	3222.02 cm^{-1}
83:	1199.63 cm^{-1}	107:	1588.90 cm^{-1}	131:	3228.08 cm^{-1}

This molecular structure was also optimized and the vibrational frequencies calculated using the BP86, the TPSS or the B3LYP density functional. The deviation from the results given above are negligible though. The xyz coordinates and vibrational frequencies can be found in the corresponding Orca output file within the deposited raw data.

[BPI–AlMe$_2$]$^-$ ([**1**]$^{\bullet-}$) – PBE0-D3BJ/def2-TZVP/CPCM(THF) (D$_1$)

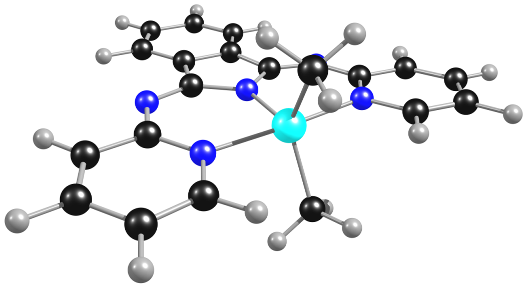

Al	2.762164000	9.676112000	5.440935000
N	3.247781000	8.255919000	4.225269000
N	0.870628000	9.697583000	4.545176000
N	5.506617000	7.695971000	5.008659000
N	1.233973000	8.102222000	2.828602000
N	4.690378000	9.547776000	6.245140000
C	6.858237000	8.633728000	6.696516000
C	5.347976000	5.643368000	2.747415000
H	6.306625000	5.547312000	3.245363000
C	2.455062000	7.759130000	3.206611000
C	7.077608000	9.567674000	7.669081000
H	8.009107000	9.571149000	8.224860000
C	5.015830000	4.837194000	1.677161000
H	5.721246000	4.093003000	1.323569000
C	4.446022000	7.565131000	4.227811000
C	2.843431000	5.898090000	1.451080000
H	1.883511000	5.997664000	0.956230000
C	5.642266000	8.623483000	5.962938000
C	3.174387000	6.715420000	2.532251000
C	3.769571000	4.964266000	1.031716000
H	3.539449000	4.315711000	0.193326000
C	4.415578000	6.589043000	3.175095000
C	0.471094000	9.011563000	3.445234000
C	3.066935000	11.569421000	4.823181000
H	4.111528000	11.896308000	4.877178000
H	2.476239000	12.305969000	5.383757000
H	2.756381000	11.670792000	3.774799000
C	0.022961000	10.566116000	5.115396000

H	0.401425000	11.072134000	5.997972000
C	-1.240596000	10.829782000	4.652344000
H	-1.872314000	11.545347000	5.161877000
C	-0.825175000	9.245602000	2.914481000
C	1.992275000	9.148190000	7.226233000
H	0.943113000	8.833881000	7.187518000
H	2.053972000	9.949443000	7.974584000
H	2.561605000	8.305781000	7.640923000
C	4.929739000	10.460132000	7.198142000
H	4.131800000	11.176612000	7.365475000
C	6.084620000	10.523258000	7.934830000
H	6.211030000	11.287240000	8.690550000
C	-1.669226000	10.141941000	3.506471000
H	-2.658658000	10.315248000	3.097062000
H	7.597484000	7.881162000	6.451382000
H	-1.114170000	8.685832000	2.033505000

0:	0.00 cm^{-1}	20:	234.38 cm^{-1}	40:	601.13 cm^{-1}
1:	0.00 cm^{-1}	21:	251.83 cm^{-1}	41:	632.96 cm^{-1}
2:	0.00 cm^{-1}	22:	274.30 cm^{-1}	42:	641.49 cm^{-1}
3:	0.00 cm^{-1}	23:	276.97 cm^{-1}	43:	645.26 cm^{-1}
4:	0.00 cm^{-1}	24:	283.63 cm^{-1}	44:	650.41 cm^{-1}
5:	0.00 cm^{-1}	25:	286.52 cm^{-1}	45:	651.57 cm^{-1}
6:	40.83 cm^{-1}	26:	303.51 cm^{-1}	46:	672.06 cm^{-1}
7:	51.88 cm^{-1}	27:	327.63 cm^{-1}	47:	683.18 cm^{-1}
8:	62.30 cm^{-1}	28:	359.03 cm^{-1}	48:	696.19 cm^{-1}
9:	86.31 cm^{-1}	29:	387.41 cm^{-1}	49:	705.61 cm^{-1}
10:	88.33 cm^{-1}	30:	438.04 cm^{-1}	50:	720.41 cm^{-1}
11:	107.50 cm^{-1}	31:	441.91 cm^{-1}	51:	727.14 cm^{-1}
12:	111.84 cm^{-1}	32:	455.97 cm^{-1}	52:	759.57 cm^{-1}
13:	120.71 cm^{-1}	33:	456.75 cm^{-1}	53:	759.77 cm^{-1}
14:	141.20 cm^{-1}	34:	525.79 cm^{-1}	54:	776.69 cm^{-1}
15:	142.49 cm^{-1}	35:	536.99 cm^{-1}	55:	791.91 cm^{-1}
16:	151.67 cm^{-1}	36:	541.51 cm^{-1}	56:	794.26 cm^{-1}
17:	160.48 cm^{-1}	37:	550.46 cm^{-1}	57:	803.07 cm^{-1}
18:	208.97 cm^{-1}	38:	576.90 cm^{-1}	58:	835.36 cm^{-1}
19:	231.36 cm^{-1}	39:	581.09 cm^{-1}	59:	874.37 cm^{-1}

60:	874.80 cm^{-1}	84:	1191.17 cm^{-1}	108:	1592.14 cm^{-1}
61:	883.52 cm^{-1}	85:	1200.20 cm^{-1}	109:	1609.06 cm^{-1}
62:	885.21 cm^{-1}	86:	1208.35 cm^{-1}	110:	1627.77 cm^{-1}
63:	903.49 cm^{-1}	87:	1294.14 cm^{-1}	111:	1645.39 cm^{-1}
64:	913.50 cm^{-1}	88:	1300.65 cm^{-1}	112:	1667.39 cm^{-1}
65:	966.02 cm^{-1}	89:	1317.91 cm^{-1}	113:	1668.64 cm^{-1}
66:	983.96 cm^{-1}	90:	1329.29 cm^{-1}	114:	3004.96 cm^{-1}
67:	984.70 cm^{-1}	91:	1331.48 cm^{-1}	115:	3006.81 cm^{-1}
68:	985.60 cm^{-1}	92:	1368.65 cm^{-1}	116:	3071.54 cm^{-1}
69:	1000.58 cm^{-1}	93:	1380.67 cm^{-1}	117:	3071.84 cm^{-1}
70:	1001.13 cm^{-1}	94:	1387.10 cm^{-1}	118:	3085.87 cm^{-1}
71:	1021.19 cm^{-1}	95:	1418.66 cm^{-1}	119:	3086.30 cm^{-1}
72:	1027.69 cm^{-1}	96:	1431.51 cm^{-1}	120:	3182.62 cm^{-1}
73:	1030.16 cm^{-1}	97:	1434.00 cm^{-1}	121:	3187.20 cm^{-1}
74:	1066.83 cm^{-1}	98:	1436.69 cm^{-1}	122:	3189.12 cm^{-1}
75:	1068.07 cm^{-1}	99:	1439.82 cm^{-1}	123:	3192.83 cm^{-1}
76:	1078.85 cm^{-1}	100:	1465.90 cm^{-1}	124:	3193.31 cm^{-1}
77:	1117.90 cm^{-1}	101:	1467.42 cm^{-1}	125:	3194.07 cm^{-1}
78:	1134.21 cm^{-1}	102:	1496.15 cm^{-1}	126:	3201.89 cm^{-1}
79:	1135.95 cm^{-1}	103:	1506.70 cm^{-1}	127:	3209.27 cm^{-1}
80:	1146.90 cm^{-1}	104:	1519.83 cm^{-1}	128:	3216.65 cm^{-1}
81:	1156.11 cm^{-1}	105:	1527.15 cm^{-1}	129:	3218.60 cm^{-1}
82:	1157.81 cm^{-1}	106:	1536.51 cm^{-1}	130:	3229.38 cm^{-1}
83:	1185.11 cm^{-1}	107:	1591.20 cm^{-1}	131:	3229.71 cm^{-1}

This molecular structure was also optimized and the vibrational frequencies calculated using the BP86, the TPSS or the B3LYP density functional. The deviation from the results given above are negligible though. The xyz coordinates and vibrational frequencies can be found in the corresponding Orca output file within the deposited raw data.

[BPI–AlMe]⁻ ([**9**]⁻) – PBE0-D3BJ/def2-TZVP (S_0)

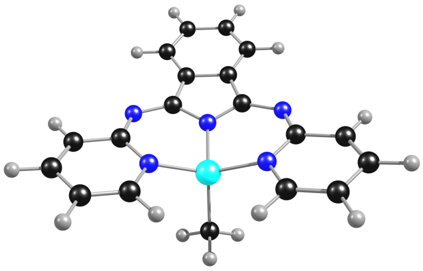

Al	3.136700000	9.985560000	4.658017000
N	3.443233000	8.410472000	3.807411000
N	1.247141000	9.780128000	4.605454000
N	5.578123000	7.929070000	4.817135000
N	1.279426000	8.199227000	2.767391000
N	4.336687000	9.586840000	6.077668000
C	6.492221000	8.766875000	6.810600000
C	5.421372000	5.629555000	2.593556000
H	6.394026000	5.564727000	3.069413000
C	2.575354000	7.835228000	2.920077000
C	6.416715000	9.509428000	7.939891000
H	7.226772000	9.489651000	8.661526000
C	5.031299000	4.719976000	1.643512000
H	5.705239000	3.920257000	1.352800000
C	4.613641000	7.707253000	3.892010000
C	2.865232000	5.789881000	1.374468000
H	1.882943000	5.847633000	0.917887000
C	5.439240000	8.736154000	5.833397000
C	3.245120000	6.744314000	2.332167000
C	3.752003000	4.800233000	1.033385000
H	3.475074000	4.060156000	0.289169000
C	4.539611000	6.663075000	2.949485000
C	0.661326000	9.036050000	3.555565000
C	3.529207000	11.821882000	4.074189000
H	4.600343000	11.973121000	3.908406000
H	3.217917000	12.557572000	4.825824000
H	3.001621000	12.072743000	3.148758000
C	0.458651000	10.559675000	5.387028000
H	0.979825000	11.105967000	6.170588000

C	-0.882834000	10.702473000	5.242585000
H	-1.441880000	11.341095000	5.912742000
C	-0.749725000	9.221584000	3.357760000
C	4.303612000	10.319976000	7.218597000
H	3.427537000	10.954766000	7.335112000
C	5.267557000	10.318166000	8.173378000
H	5.163103000	10.929071000	9.059585000
C	-1.501680000	10.005776000	4.165207000
H	-2.568307000	10.103862000	3.991931000
H	7.344335000	8.134545000	6.595247000
H	-1.179205000	8.670373000	2.530696000

0:	0.00 cm^{-1}	25:	399.83 cm^{-1}	50:	755.48 cm^{-1}
1:	0.00 cm^{-1}	26:	403.61 cm^{-1}	51:	764.59 cm^{-1}
2:	0.00 cm^{-1}	27:	427.42 cm^{-1}	52:	818.04 cm^{-1}
3:	0.00 cm^{-1}	28:	440.73 cm^{-1}	53:	819.01 cm^{-1}
4:	0.00 cm^{-1}	29:	442.82 cm^{-1}	54:	836.40 cm^{-1}
5:	0.00 cm^{-1}	30:	460.63 cm^{-1}	55:	839.01 cm^{-1}
6:	37.63 cm^{-1}	31:	529.57 cm^{-1}	56:	849.84 cm^{-1}
7:	47.93 cm^{-1}	32:	530.68 cm^{-1}	57:	902.42 cm^{-1}
8:	52.12 cm^{-1}	33:	538.08 cm^{-1}	58:	912.64 cm^{-1}
9:	80.50 cm^{-1}	34:	576.42 cm^{-1}	59:	913.75 cm^{-1}
10:	84.10 cm^{-1}	35:	602.60 cm^{-1}	60:	919.43 cm^{-1}
11:	96.52 cm^{-1}	36:	613.09 cm^{-1}	61:	921.94 cm^{-1}
12:	122.61 cm^{-1}	37:	630.16 cm^{-1}	62:	925.55 cm^{-1}
13:	125.59 cm^{-1}	38:	641.69 cm^{-1}	63:	968.41 cm^{-1}
14:	145.26 cm^{-1}	39:	661.19 cm^{-1}	64:	969.11 cm^{-1}
15:	157.76 cm^{-1}	40:	663.20 cm^{-1}	65:	1019.28 cm^{-1}
16:	179.40 cm^{-1}	41:	677.31 cm^{-1}	66:	1027.30 cm^{-1}
17:	218.84 cm^{-1}	42:	688.91 cm^{-1}	67:	1030.51 cm^{-1}
18:	249.88 cm^{-1}	43:	696.24 cm^{-1}	68:	1032.58 cm^{-1}
19:	252.49 cm^{-1}	44:	702.36 cm^{-1}	69:	1040.59 cm^{-1}
20:	258.03 cm^{-1}	45:	723.92 cm^{-1}	70:	1089.13 cm^{-1}
21:	285.85 cm^{-1}	46:	728.11 cm^{-1}	71:	1140.68 cm^{-1}
22:	330.38 cm^{-1}	47:	729.51 cm^{-1}	72:	1142.27 cm^{-1}
23:	350.03 cm^{-1}	48:	730.30 cm^{-1}	73:	1145.74 cm^{-1}
24:	361.92 cm^{-1}	49:	741.33 cm^{-1}	74:	1149.59 cm^{-1}

75:	1154.72 cm^{-1}	90:	1447.66 cm^{-1}	105:	3022.76 cm^{-1}
76:	1156.36 cm^{-1}	91:	1467.02 cm^{-1}	106:	3092.00 cm^{-1}
77:	1205.59 cm^{-1}	92:	1481.53 cm^{-1}	107:	3113.70 cm^{-1}
78:	1220.85 cm^{-1}	93:	1490.46 cm^{-1}	108:	3139.71 cm^{-1}
79:	1251.38 cm^{-1}	94:	1500.81 cm^{-1}	109:	3144.33 cm^{-1}
80:	1259.65 cm^{-1}	95:	1522.10 cm^{-1}	110:	3160.38 cm^{-1}
81:	1265.32 cm^{-1}	96:	1548.93 cm^{-1}	111:	3175.55 cm^{-1}
82:	1315.71 cm^{-1}	97:	1571.72 cm^{-1}	112:	3176.45 cm^{-1}
83:	1321.66 cm^{-1}	98:	1581.13 cm^{-1}	113:	3177.27 cm^{-1}
84:	1348.87 cm^{-1}	99:	1589.82 cm^{-1}	114:	3190.25 cm^{-1}
85:	1382.81 cm^{-1}	100:	1591.12 cm^{-1}	115:	3196.88 cm^{-1}
86:	1392.86 cm^{-1}	101:	1598.05 cm^{-1}	116:	3214.24 cm^{-1}
87:	1395.37 cm^{-1}	102:	1651.29 cm^{-1}	117:	3218.23 cm^{-1}
88:	1417.07 cm^{-1}	103:	1674.37 cm^{-1}	118:	3224.82 cm^{-1}
89:	1442.71 cm^{-1}	104:	1675.24 cm^{-1}	119:	3227.51 cm^{-1}

This molecular structure was also optimized and the vibrational frequencies calculated using the BP86, the TPSS or the B3LYP density functional. The deviation from the results given above are negligible though. The xyz coordinates and vibrational frequencies can be found in the corresponding Orca output file within the deposited raw data.

[BPI–AlMe]⁻ ([**9**]⁻) – PBE0-D3BJ/def2-TZVP/CPCM(THF) (S_0)

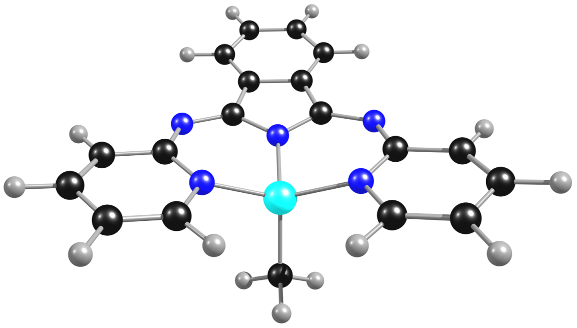

Al	2.574293000	8.871256000	5.774443000
N	3.161215000	7.839886000	4.383600000
N	1.209812000	9.688977000	4.716621000
N	5.458466000	7.641632000	5.099894000
N	1.137424000	7.923207000	3.069545000
N	4.333577000	9.479149000	6.191868000
C	6.668769000	9.012850000	6.581252000
C	5.394811000	5.562433000	2.656844000
H	6.380419000	5.497936000	3.107492000
C	2.366094000	7.418561000	3.353624000
C	6.774302000	10.103623000	7.379859000
H	7.720898000	10.342492000	7.852872000
C	5.088586000	4.830836000	1.537107000
H	5.835448000	4.177648000	1.097932000
C	4.409755000	7.285469000	4.313542000
C	2.829977000	5.731436000	1.451031000
H	1.854252000	5.796638000	0.979439000
C	5.443484000	8.669185000	5.916597000
C	3.109405000	6.483212000	2.608045000
C	3.804069000	4.915493000	0.933132000
H	3.598771000	4.325369000	0.046038000
C	4.411529000	6.398175000	3.219769000
C	0.631521000	8.985681000	3.651142000
C	0.619827000	10.827643000	5.167083000
H	1.133204000	11.316965000	5.990230000
C	-0.531333000	11.347409000	4.667839000
H	-0.940950000	12.262728000	5.073126000
C	-0.615652000	9.490409000	3.149803000

C	1.744355000	8.256200000	7.439859000
H	0.820532000	7.699111000	7.247400000
H	1.481021000	9.094940000	8.095495000
H	2.415416000	7.603527000	8.009417000
C	4.475504000	10.563092000	6.999098000
H	3.567552000	11.138116000	7.159490000
C	5.639695000	10.930774000	7.594187000
H	5.681413000	11.810673000	8.221792000
C	-1.175535000	10.630255000	3.624902000
H	-2.110146000	10.992246000	3.209838000
H	7.516276000	8.364758000	6.392505000
H	-1.076865000	8.923249000	2.350120000

0:	0.00 cm^{-1}	24:	356.44 cm^{-1}	48:	753.88 cm^{-1}
1:	0.00 cm^{-1}	25:	400.57 cm^{-1}	49:	755.15 cm^{-1}
2:	0.00 cm^{-1}	26:	403.00 cm^{-1}	50:	763.27 cm^{-1}
3:	0.00 cm^{-1}	27:	429.20 cm^{-1}	51:	784.24 cm^{-1}
4:	0.00 cm^{-1}	28:	440.33 cm^{-1}	52:	832.95 cm^{-1}
5:	0.00 cm^{-1}	29:	442.61 cm^{-1}	53:	842.35 cm^{-1}
6:	42.08 cm^{-1}	30:	462.06 cm^{-1}	54:	844.01 cm^{-1}
7:	47.51 cm^{-1}	31:	534.42 cm^{-1}	55:	853.03 cm^{-1}
8:	48.09 cm^{-1}	32:	535.46 cm^{-1}	56:	858.37 cm^{-1}
9:	84.02 cm^{-1}	33:	542.53 cm^{-1}	57:	904.09 cm^{-1}
10:	87.25 cm^{-1}	34:	578.12 cm^{-1}	58:	907.70 cm^{-1}
11:	119.37 cm^{-1}	35:	601.15 cm^{-1}	59:	950.76 cm^{-1}
12:	122.91 cm^{-1}	36:	615.80 cm^{-1}	60:	958.66 cm^{-1}
13:	142.20 cm^{-1}	37:	620.01 cm^{-1}	61:	959.95 cm^{-1}
14:	147.91 cm^{-1}	38:	638.73 cm^{-1}	62:	967.17 cm^{-1}
15:	156.66 cm^{-1}	39:	658.35 cm^{-1}	63:	994.16 cm^{-1}
16:	193.05 cm^{-1}	40:	673.15 cm^{-1}	64:	994.37 cm^{-1}
17:	225.47 cm^{-1}	41:	673.46 cm^{-1}	65:	1015.73 cm^{-1}
18:	249.36 cm^{-1}	42:	692.75 cm^{-1}	66:	1026.52 cm^{-1}
19:	254.84 cm^{-1}	43:	710.00 cm^{-1}	67:	1034.83 cm^{-1}
20:	257.17 cm^{-1}	44:	713.00 cm^{-1}	68:	1041.42 cm^{-1}
21:	288.58 cm^{-1}	45:	734.43 cm^{-1}	69:	1042.66 cm^{-1}
22:	329.32 cm^{-1}	46:	740.31 cm^{-1}	70:	1085.29 cm^{-1}
23:	352.70 cm^{-1}	47:	745.67 cm^{-1}	71:	1138.19 cm^{-1}

72:	1139.50 cm^{-1}	88:	1414.16 cm^{-1}	104:	1677.08 cm^{-1}
73:	1142.80 cm^{-1}	89:	1422.79 cm^{-1}	105:	3021.64 cm^{-1}
74:	1146.33 cm^{-1}	90:	1434.24 cm^{-1}	106:	3094.93 cm^{-1}
75:	1150.43 cm^{-1}	91:	1461.89 cm^{-1}	107:	3103.52 cm^{-1}
76:	1152.27 cm^{-1}	92:	1477.32 cm^{-1}	108:	3170.81 cm^{-1}
77:	1202.40 cm^{-1}	93:	1494.88 cm^{-1}	109:	3171.93 cm^{-1}
78:	1210.30 cm^{-1}	94:	1501.89 cm^{-1}	110:	3172.80 cm^{-1}
79:	1253.51 cm^{-1}	95:	1523.74 cm^{-1}	111:	3178.58 cm^{-1}
80:	1268.01 cm^{-1}	96:	1541.82 cm^{-1}	112:	3189.38 cm^{-1}
81:	1273.62 cm^{-1}	97:	1556.80 cm^{-1}	113:	3189.69 cm^{-1}
82:	1311.87 cm^{-1}	98:	1570.99 cm^{-1}	114:	3191.30 cm^{-1}
83:	1315.11 cm^{-1}	99:	1580.06 cm^{-1}	115:	3200.33 cm^{-1}
84:	1347.76 cm^{-1}	100:	1582.44 cm^{-1}	116:	3212.98 cm^{-1}
85:	1373.69 cm^{-1}	101:	1591.08 cm^{-1}	117:	3213.53 cm^{-1}
86:	1394.84 cm^{-1}	102:	1644.54 cm^{-1}	118:	3230.73 cm^{-1}
87:	1397.00 cm^{-1}	103:	1676.98 cm^{-1}	119:	3231.51 cm^{-1}

This molecular structure was also optimized and the vibrational frequencies calculated using the BP86, the TPSS or the B3LYP density functional. The deviation from the results given above are negligible though. The xyz coordinates and vibrational frequencies can be found in the corresponding Orca output file within the deposited raw data.

[BPI–AlMe]$^-$ ([**9**]$^-$) – PBE0-D3BJ/def2-TZVP (T_1)

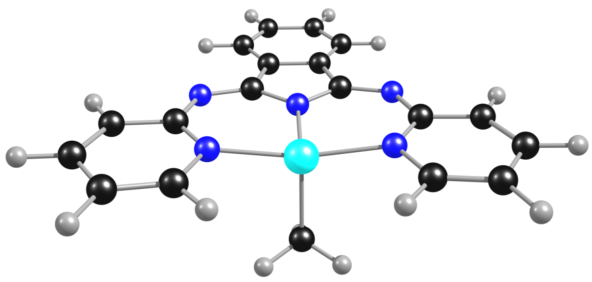

Al	2.826961000	9.824872000	5.301245000
N	3.247418000	8.299665000	4.200718000
N	0.908876000	9.764339000	4.563312000
N	5.522668000	7.796757000	4.944236000
N	1.180889000	8.065377000	2.913407000
N	4.603887000	9.533270000	6.294490000
C	6.795684000	8.658722000	6.721860000
C	5.373015000	5.752817000	2.664789000
H	6.348394000	5.702245000	3.134935000
C	2.431368000	7.770422000	3.223451000
C	6.962414000	9.515345000	7.771387000
H	7.880688000	9.500958000	8.349730000
C	5.028283000	4.928801000	1.611822000
H	5.745541000	4.207400000	1.234160000
C	4.460483000	7.644947000	4.172547000
C	2.813273000	5.911038000	1.467556000
H	1.828196000	5.981724000	1.020792000
C	5.598688000	8.660247000	5.956746000
C	3.158942000	6.746569000	2.526371000
C	3.754803000	5.007524000	1.016194000
H	3.514692000	4.345320000	0.190757000
C	4.427609000	6.668164000	3.119732000
C	0.445770000	8.980245000	3.545205000
C	3.341959000	11.558013000	4.434063000
H	4.089680000	12.118925000	5.004228000
H	2.489161000	12.221902000	4.258568000
H	3.785781000	11.343172000	3.451669000
C	0.081004000	10.654846000	5.127453000
H	0.526902000	11.218208000	5.945470000

C	-1.219913000	10.853927000	4.748134000
H	-1.827998000	11.595124000	5.250148000
C	-0.900945000	9.136184000	3.120613000
C	4.787853000	10.359353000	7.333860000
H	3.934818000	11.001936000	7.545541000
C	5.925313000	10.407211000	8.095356000
H	6.002167000	11.104553000	8.919347000
C	-1.722562000	10.055622000	3.705986000
H	-2.750428000	10.161732000	3.374119000
H	7.559935000	7.951451000	6.426007000
H	-1.232693000	8.495461000	2.313610000

0:	0.00 cm^{-1}	25:	366.07 cm^{-1}	50:	774.11 cm^{-1}
1:	0.00 cm^{-1}	26:	389.34 cm^{-1}	51:	789.18 cm^{-1}
2:	0.00 cm^{-1}	27:	435.97 cm^{-1}	52:	838.30 cm^{-1}
3:	0.00 cm^{-1}	28:	436.95 cm^{-1}	53:	851.61 cm^{-1}
4:	0.00 cm^{-1}	29:	446.97 cm^{-1}	54:	851.80 cm^{-1}
5:	0.00 cm^{-1}	30:	456.74 cm^{-1}	55:	864.84 cm^{-1}
6:	29.58 cm^{-1}	31:	535.59 cm^{-1}	56:	875.79 cm^{-1}
7:	40.05 cm^{-1}	32:	536.82 cm^{-1}	57:	890.47 cm^{-1}
8:	56.43 cm^{-1}	33:	544.25 cm^{-1}	58:	915.39 cm^{-1}
9:	58.69 cm^{-1}	34:	574.40 cm^{-1}	59:	938.12 cm^{-1}
10:	76.90 cm^{-1}	35:	586.34 cm^{-1}	60:	939.28 cm^{-1}
11:	89.57 cm^{-1}	36:	600.94 cm^{-1}	61:	957.80 cm^{-1}
12:	114.00 cm^{-1}	37:	620.76 cm^{-1}	62:	959.10 cm^{-1}
13:	141.53 cm^{-1}	38:	638.97 cm^{-1}	63:	972.58 cm^{-1}
14:	143.89 cm^{-1}	39:	643.95 cm^{-1}	64:	973.57 cm^{-1}
15:	160.68 cm^{-1}	40:	650.43 cm^{-1}	65:	1016.36 cm^{-1}
16:	196.13 cm^{-1}	41:	670.25 cm^{-1}	66:	1022.53 cm^{-1}
17:	214.32 cm^{-1}	42:	683.00 cm^{-1}	67:	1028.27 cm^{-1}
18:	220.98 cm^{-1}	43:	705.20 cm^{-1}	68:	1056.73 cm^{-1}
19:	262.99 cm^{-1}	44:	717.06 cm^{-1}	69:	1057.62 cm^{-1}
20:	273.24 cm^{-1}	45:	728.04 cm^{-1}	70:	1088.54 cm^{-1}
21:	280.15 cm^{-1}	46:	745.62 cm^{-1}	71:	1117.11 cm^{-1}
22:	292.78 cm^{-1}	47:	745.97 cm^{-1}	72:	1131.08 cm^{-1}
23:	321.86 cm^{-1}	48:	760.02 cm^{-1}	73:	1132.60 cm^{-1}
24:	326.66 cm^{-1}	49:	761.05 cm^{-1}	74:	1150.25 cm^{-1}

75:	1156.62 cm^{-1}	90:	1448.38 cm^{-1}	105:	3001.51 cm^{-1}
76:	1158.87 cm^{-1}	91:	1466.15 cm^{-1}	106:	3075.31 cm^{-1}
77:	1190.34 cm^{-1}	92:	1468.60 cm^{-1}	107:	3100.81 cm^{-1}
78:	1206.03 cm^{-1}	93:	1494.83 cm^{-1}	108:	3131.73 cm^{-1}
79:	1215.51 cm^{-1}	94:	1508.57 cm^{-1}	109:	3132.18 cm^{-1}
80:	1275.87 cm^{-1}	95:	1523.07 cm^{-1}	110:	3166.65 cm^{-1}
81:	1285.71 cm^{-1}	96:	1534.00 cm^{-1}	111:	3174.17 cm^{-1}
82:	1315.32 cm^{-1}	97:	1547.59 cm^{-1}	112:	3175.89 cm^{-1}
83:	1329.03 cm^{-1}	98:	1577.08 cm^{-1}	113:	3184.85 cm^{-1}
84:	1332.25 cm^{-1}	99:	1584.55 cm^{-1}	114:	3204.25 cm^{-1}
85:	1377.20 cm^{-1}	100:	1601.52 cm^{-1}	115:	3207.85 cm^{-1}
86:	1381.68 cm^{-1}	101:	1632.54 cm^{-1}	116:	3214.82 cm^{-1}
87:	1387.05 cm^{-1}	102:	1647.23 cm^{-1}	117:	3215.02 cm^{-1}
88:	1420.76 cm^{-1}	103:	1660.80 cm^{-1}	118:	3219.81 cm^{-1}
89:	1447.81 cm^{-1}	104:	1662.99 cm^{-1}	119:	3220.49 cm^{-1}

This molecular structure was also optimized and the vibrational frequencies calculated using the BP86, the TPSS or the B3LYP density functional. The deviation from the results given above are negligible though. The xyz coordinates and vibrational frequencies can be found in the corresponding Orca output file within the deposited raw data.

[BPI–AlMe]$^{-}$ ([**9**]$^{-}$) – PBE0-D3BJ/def2-TZVP/CPCM(THF) (T_1)

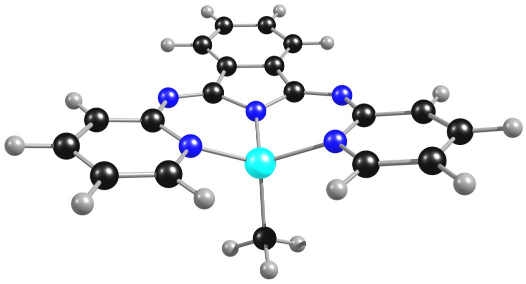

Al	2.532174000	8.869771000	5.786661000
N	3.146294000	7.824545000	4.414198000
N	1.214297000	9.687091000	4.735561000
N	5.439853000	7.631741000	5.080735000
N	1.214157000	7.962573000	3.010869000
N	4.319381000	9.466787000	6.197993000
C	6.660075000	9.029636000	6.525489000
C	5.386531000	5.536338000	2.679996000
H	6.364546000	5.460037000	3.142698000
C	2.371726000	7.458388000	3.346327000
C	6.775721000	10.124274000	7.328146000
H	7.731657000	10.375569000	7.773967000
C	5.079588000	4.807552000	1.548239000
H	5.824065000	4.145123000	1.119599000
C	4.400786000	7.274171000	4.333195000
C	2.846403000	5.750516000	1.448905000
H	1.877702000	5.842691000	0.969223000
C	5.426848000	8.678042000	5.909743000
C	3.135795000	6.476348000	2.597730000
C	3.818478000	4.909900000	0.933613000
H	3.611448000	4.326512000	0.043377000
C	4.405639000	6.372415000	3.220830000
C	0.642156000	9.054921000	3.623593000
C	0.555164000	10.813451000	5.239605000
H	1.028007000	11.283453000	6.094925000
C	-0.611366000	11.301493000	4.712873000
H	-1.057564000	12.180244000	5.167272000
C	-0.533540000	9.534041000	3.083296000
C	1.778914000	8.232520000	7.480830000

H	0.860775000	7.656900000	7.317489000
H	1.519319000	9.063101000	8.148035000
H	2.482229000	7.592016000	8.024932000
C	4.462541000	10.549258000	7.000895000
H	3.552122000	11.113589000	7.180422000
C	5.639193000	10.928750000	7.571328000
H	5.682760000	11.808042000	8.199741000
C	-1.204354000	10.673705000	3.614791000
H	-2.124633000	11.037995000	3.176641000
H	7.509935000	8.394230000	6.308452000
H	-0.923637000	9.005511000	2.221656000

0:	0.00 cm^{-1}	25:	359.66 cm^{-1}	50:	765.42 cm^{-1}
1:	0.00 cm^{-1}	26:	395.75 cm^{-1}	51:	771.87 cm^{-1}
2:	0.00 cm^{-1}	27:	428.27 cm^{-1}	52:	795.86 cm^{-1}
3:	0.00 cm^{-1}	28:	435.68 cm^{-1}	53:	808.00 cm^{-1}
4:	0.00 cm^{-1}	29:	450.20 cm^{-1}	54:	842.75 cm^{-1}
5:	0.00 cm^{-1}	30:	463.56 cm^{-1}	55:	854.55 cm^{-1}
6:	43.61 cm^{-1}	31:	523.51 cm^{-1}	56:	861.19 cm^{-1}
7:	50.23 cm^{-1}	32:	538.35 cm^{-1}	57:	878.36 cm^{-1}
8:	53.14 cm^{-1}	33:	557.86 cm^{-1}	58:	890.93 cm^{-1}
9:	74.50 cm^{-1}	34:	574.77 cm^{-1}	59:	906.61 cm^{-1}
10:	83.06 cm^{-1}	35:	579.58 cm^{-1}	60:	914.13 cm^{-1}
11:	112.32 cm^{-1}	36:	603.96 cm^{-1}	61:	962.64 cm^{-1}
12:	120.36 cm^{-1}	37:	610.65 cm^{-1}	62:	967.73 cm^{-1}
13:	126.09 cm^{-1}	38:	629.83 cm^{-1}	63:	981.78 cm^{-1}
14:	145.86 cm^{-1}	39:	637.43 cm^{-1}	64:	993.54 cm^{-1}
15:	162.92 cm^{-1}	40:	645.02 cm^{-1}	65:	996.57 cm^{-1}
16:	171.24 cm^{-1}	41:	659.37 cm^{-1}	66:	1026.54 cm^{-1}
17:	219.19 cm^{-1}	42:	665.47 cm^{-1}	67:	1030.77 cm^{-1}
18:	228.85 cm^{-1}	43:	675.29 cm^{-1}	68:	1035.31 cm^{-1}
19:	259.23 cm^{-1}	44:	680.24 cm^{-1}	69:	1050.68 cm^{-1}
20:	266.96 cm^{-1}	45:	689.60 cm^{-1}	70:	1054.16 cm^{-1}
21:	287.55 cm^{-1}	46:	701.17 cm^{-1}	71:	1090.82 cm^{-1}
22:	310.96 cm^{-1}	47:	719.73 cm^{-1}	72:	1101.49 cm^{-1}
23:	322.09 cm^{-1}	48:	737.92 cm^{-1}	73:	1135.80 cm^{-1}
24:	346.02 cm^{-1}	49:	746.52 cm^{-1}	74:	1138.00 cm^{-1}

75:	1146.48 cm^{-1}	90:	1422.02 cm^{-1}	105:	3020.72 cm^{-1}
76:	1155.31 cm^{-1}	91:	1423.83 cm^{-1}	106:	3095.69 cm^{-1}
77:	1188.53 cm^{-1}	92:	1439.64 cm^{-1}	107:	3102.71 cm^{-1}
78:	1194.42 cm^{-1}	93:	1459.99 cm^{-1}	108:	3176.11 cm^{-1}
79:	1237.29 cm^{-1}	94:	1479.86 cm^{-1}	109:	3180.16 cm^{-1}
80:	1254.70 cm^{-1}	95:	1491.35 cm^{-1}	110:	3183.12 cm^{-1}
81:	1269.81 cm^{-1}	96:	1511.70 cm^{-1}	111:	3190.92 cm^{-1}
82:	1283.18 cm^{-1}	97:	1517.98 cm^{-1}	112:	3191.85 cm^{-1}
83:	1304.71 cm^{-1}	98:	1530.56 cm^{-1}	113:	3196.30 cm^{-1}
84:	1313.70 cm^{-1}	99:	1562.29 cm^{-1}	114:	3199.29 cm^{-1}
85:	1324.84 cm^{-1}	100:	1591.71 cm^{-1}	115:	3200.82 cm^{-1}
86:	1352.96 cm^{-1}	101:	1612.50 cm^{-1}	116:	3209.21 cm^{-1}
87:	1379.68 cm^{-1}	102:	1633.49 cm^{-1}	117:	3217.85 cm^{-1}
88:	1386.77 cm^{-1}	103:	1641.28 cm^{-1}	118:	3218.65 cm^{-1}
89:	1411.02 cm^{-1}	104:	1658.89 cm^{-1}	119:	3233.17 cm^{-1}

This molecular structure was also optimized and the vibrational frequencies calculated using the BP86, the TPSS or the B3LYP density functional. The deviation from the results given above are negligible though. The xyz coordinates and vibrational frequencies can be found in the corresponding Orca output file within the deposited raw data.

[BPI–AlMe$_2$]$^{2-}$ ([**1**]$^{2-}$) – PBE0-D3BJ/def2-TZVP (S_0)

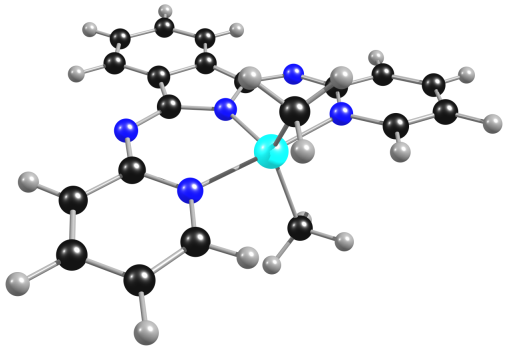

Al	2.768900000	9.658441000	5.423617000
N	3.253240000	8.239811000	4.210587000
N	0.870270000	9.670695000	4.567433000
N	5.502430000	7.644335000	5.073327000
N	1.235247000	8.159021000	2.768753000
N	4.694805000	9.568155000	6.213656000
C	6.841775000	8.599806000	6.756792000
C	5.332490000	5.607154000	2.769246000
H	6.275854000	5.495865000	3.295448000
C	2.470791000	7.767600000	3.168579000
C	7.075068000	9.565853000	7.682220000
H	7.998563000	9.561167000	8.257079000
C	5.015754000	4.816390000	1.690113000
H	5.716841000	4.059381000	1.346921000
C	4.440756000	7.525762000	4.237638000
C	2.864655000	5.921650000	1.416344000
H	1.915158000	6.050968000	0.905507000
C	5.634755000	8.579332000	5.972445000
C	3.170836000	6.738287000	2.516234000
C	3.774823000	4.975103000	1.009180000
H	3.550468000	4.336427000	0.158221000
C	4.415439000	6.578902000	3.199393000
C	0.490279000	9.019294000	3.405215000
C	3.015104000	11.585845000	4.820951000
H	4.002706000	12.017672000	5.017693000
H	2.263629000	12.271650000	5.235559000
H	2.863638000	11.601690000	3.733183000

C	0.001351000	10.494031000	5.159431000
H	0.369598000	10.956864000	6.071897000
C	-1.263438000	10.781074000	4.710135000
H	-1.901683000	11.465198000	5.255357000
C	-0.819812000	9.301595000	2.879441000
C	2.032793000	9.170176000	7.256378000
H	0.949090000	9.014807000	7.301193000
H	2.297278000	9.894518000	8.038938000
H	2.509912000	8.226079000	7.551901000
C	4.962858000	10.504141000	7.127630000
H	4.181639000	11.250483000	7.250359000
C	6.104150000	10.578873000	7.886486000
H	6.236780000	11.375084000	8.608366000
C	-1.673004000	10.150969000	3.507665000
H	-2.659903000	10.341870000	3.091841000
H	7.553861000	7.807091000	6.557532000
H	-1.086234000	8.789593000	1.961816000

0:	0.00 cm^{-1}	19:	212.80 cm^{-1}	38:	561.56 cm^{-1}
1:	0.00 cm^{-1}	20:	222.73 cm^{-1}	39:	576.25 cm^{-1}
2:	0.00 cm^{-1}	21:	242.99 cm^{-1}	40:	583.11 cm^{-1}
3:	0.00 cm^{-1}	22:	263.87 cm^{-1}	41:	592.31 cm^{-1}
4:	0.00 cm^{-1}	23:	267.49 cm^{-1}	42:	625.46 cm^{-1}
5:	0.00 cm^{-1}	24:	278.41 cm^{-1}	43:	632.73 cm^{-1}
6:	32.47 cm^{-1}	25:	280.45 cm^{-1}	44:	646.33 cm^{-1}
7:	38.00 cm^{-1}	26:	314.01 cm^{-1}	45:	654.49 cm^{-1}
8:	54.25 cm^{-1}	27:	346.01 cm^{-1}	46:	663.70 cm^{-1}
9:	60.57 cm^{-1}	28:	364.54 cm^{-1}	47:	668.19 cm^{-1}
10:	75.66 cm^{-1}	29:	382.31 cm^{-1}	48:	676.23 cm^{-1}
11:	90.56 cm^{-1}	30:	402.20 cm^{-1}	49:	682.18 cm^{-1}
12:	110.54 cm^{-1}	31:	433.58 cm^{-1}	50:	694.74 cm^{-1}
13:	121.02 cm^{-1}	32:	448.06 cm^{-1}	51:	698.68 cm^{-1}
14:	138.69 cm^{-1}	33:	455.69 cm^{-1}	52:	703.64 cm^{-1}
15:	145.89 cm^{-1}	34:	507.79 cm^{-1}	53:	710.95 cm^{-1}
16:	149.62 cm^{-1}	35:	510.99 cm^{-1}	54:	720.92 cm^{-1}
17:	156.08 cm^{-1}	36:	517.66 cm^{-1}	55:	724.27 cm^{-1}
18:	211.82 cm^{-1}	37:	538.77 cm^{-1}	56:	732.45 cm^{-1}

57:	741.64 cm^{-1}	82:	1147.97 cm^{-1}	107:	1562.14 cm^{-1}
58:	803.32 cm^{-1}	83:	1174.91 cm^{-1}	108:	1593.41 cm^{-1}
59:	804.25 cm^{-1}	84:	1176.19 cm^{-1}	109:	1597.15 cm^{-1}
60:	810.01 cm^{-1}	85:	1200.13 cm^{-1}	110:	1614.50 cm^{-1}
61:	826.72 cm^{-1}	86:	1223.51 cm^{-1}	111:	1632.83 cm^{-1}
62:	869.54 cm^{-1}	87:	1259.46 cm^{-1}	112:	1659.10 cm^{-1}
63:	884.45 cm^{-1}	88:	1266.96 cm^{-1}	113:	1659.78 cm^{-1}
64:	893.18 cm^{-1}	89:	1316.04 cm^{-1}	114:	2993.81 cm^{-1}
65:	895.52 cm^{-1}	90:	1346.52 cm^{-1}	115:	2996.28 cm^{-1}
66:	899.22 cm^{-1}	91:	1350.08 cm^{-1}	116:	3057.47 cm^{-1}
67:	925.72 cm^{-1}	92:	1356.57 cm^{-1}	117:	3059.78 cm^{-1}
68:	926.14 cm^{-1}	93:	1390.52 cm^{-1}	118:	3079.77 cm^{-1}
69:	952.32 cm^{-1}	94:	1390.89 cm^{-1}	119:	3081.60 cm^{-1}
70:	952.89 cm^{-1}	95:	1419.63 cm^{-1}	120:	3131.43 cm^{-1}
71:	1003.55 cm^{-1}	96:	1444.21 cm^{-1}	121:	3136.97 cm^{-1}
72:	1012.79 cm^{-1}	97:	1446.23 cm^{-1}	122:	3139.22 cm^{-1}
73:	1020.67 cm^{-1}	98:	1451.83 cm^{-1}	123:	3152.68 cm^{-1}
74:	1035.33 cm^{-1}	99:	1456.30 cm^{-1}	124:	3153.57 cm^{-1}
75:	1037.22 cm^{-1}	100:	1459.33 cm^{-1}	125:	3156.07 cm^{-1}
76:	1065.71 cm^{-1}	101:	1479.90 cm^{-1}	126:	3179.02 cm^{-1}
77:	1117.43 cm^{-1}	102:	1499.88 cm^{-1}	127:	3180.73 cm^{-1}
78:	1125.69 cm^{-1}	103:	1506.03 cm^{-1}	128:	3195.66 cm^{-1}
79:	1128.71 cm^{-1}	104:	1529.32 cm^{-1}	129:	3196.46 cm^{-1}
80:	1133.96 cm^{-1}	105:	1537.77 cm^{-1}	130:	3200.84 cm^{-1}
81:	1143.98 cm^{-1}	106:	1550.46 cm^{-1}	131:	3203.71 cm^{-1}

This molecular structure was also optimized and the vibrational frequencies calculated using the BP86, the TPSS or the B3LYP density functional. The deviation from the results given above are negligible though. The xyz coordinates and vibrational frequencies can be found in the corresponding Orca output file within the deposited raw data.

[BPI–AlMe$_2$]$^{2–}$ ([**1**]$^{2–}$) – PBE0-D3BJ/def2-TZVP/CPCM(THF) (S_0)

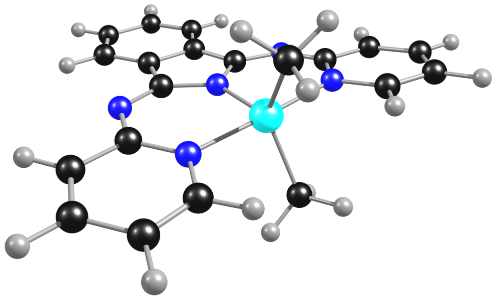

Al	2.770753000	9.649391000	5.421044000
N	3.254530000	8.231365000	4.208510000
N	0.880720000	9.673846000	4.560687000
N	5.516366000	7.660179000	5.057543000
N	1.222056000	8.138542000	2.785851000
N	4.685507000	9.554137000	6.218016000
C	6.848429000	8.627590000	6.747910000
C	5.345848000	5.611945000	2.748365000
H	6.300605000	5.498198000	3.253440000
C	2.464654000	7.749968000	3.175236000
C	7.059048000	9.586373000	7.688153000
H	7.980541000	9.595975000	8.262220000
C	5.023021000	4.818094000	1.675332000
H	5.725408000	4.068098000	1.325306000
C	4.449672000	7.528057000	4.225952000
C	2.856714000	5.898860000	1.421688000
H	1.904161000	6.005183000	0.910828000
C	5.640642000	8.594391000	5.969958000
C	3.166480000	6.724220000	2.519553000
C	3.772354000	4.962762000	1.008181000
H	3.546632000	4.320420000	0.162701000
C	4.422724000	6.579364000	3.189488000
C	0.480368000	9.014849000	3.420239000
C	3.046654000	11.565035000	4.808204000
H	4.069598000	11.945118000	4.913174000
H	2.389566000	12.278575000	5.324352000
H	2.787797000	11.641184000	3.742900000
C	0.027739000	10.523170000	5.154719000

H	0.407481000	10.996638000	6.056117000
C	-1.233440000	10.815847000	4.709338000
H	-1.860423000	11.515445000	5.246541000
C	-0.830481000	9.296359000	2.903041000
C	2.014138000	9.144893000	7.235984000
H	0.951428000	8.875241000	7.234946000
H	2.137847000	9.938070000	7.986368000
H	2.561933000	8.277284000	7.629126000
C	4.929718000	10.490181000	7.148526000
H	4.136572000	11.221186000	7.281064000
C	6.067035000	10.572263000	7.906263000
H	6.187120000	11.359751000	8.638779000
C	-1.665171000	10.169653000	3.526207000
H	-2.653184000	10.365544000	3.120924000
H	7.585871000	7.858771000	6.546513000
H	-1.125522000	8.776686000	1.998413000

0:	0.00 cm^{-1}	20:	230.07 cm^{-1}	40:	592.76 cm^{-1}
1:	0.00 cm^{-1}	21:	243.77 cm^{-1}	41:	609.39 cm^{-1}
2:	0.00 cm^{-1}	22:	265.92 cm^{-1}	42:	619.40 cm^{-1}
3:	0.00 cm^{-1}	23:	268.50 cm^{-1}	43:	628.84 cm^{-1}
4:	0.00 cm^{-1}	24:	280.96 cm^{-1}	44:	641.56 cm^{-1}
5:	0.00 cm^{-1}	25:	282.75 cm^{-1}	45:	647.01 cm^{-1}
6:	33.26 cm^{-1}	26:	311.03 cm^{-1}	46:	665.31 cm^{-1}
7:	40.96 cm^{-1}	27:	343.41 cm^{-1}	47:	673.53 cm^{-1}
8:	58.99 cm^{-1}	28:	361.08 cm^{-1}	48:	686.42 cm^{-1}
9:	90.26 cm^{-1}	29:	386.32 cm^{-1}	49:	697.77 cm^{-1}
10:	95.96 cm^{-1}	30:	394.48 cm^{-1}	50:	706.29 cm^{-1}
11:	105.17 cm^{-1}	31:	438.75 cm^{-1}	51:	719.52 cm^{-1}
12:	124.16 cm^{-1}	32:	448.00 cm^{-1}	52:	733.63 cm^{-1}
13:	139.37 cm^{-1}	33:	453.99 cm^{-1}	53:	735.14 cm^{-1}
14:	141.84 cm^{-1}	34:	509.30 cm^{-1}	54:	747.43 cm^{-1}
15:	148.87 cm^{-1}	35:	529.15 cm^{-1}	55:	751.11 cm^{-1}
16:	153.24 cm^{-1}	36:	534.93 cm^{-1}	56:	758.38 cm^{-1}
17:	160.74 cm^{-1}	37:	539.91 cm^{-1}	57:	778.80 cm^{-1}
18:	211.73 cm^{-1}	38:	557.01 cm^{-1}	58:	822.73 cm^{-1}
19:	212.97 cm^{-1}	39:	578.07 cm^{-1}	59:	832.72 cm^{-1}

60:	833.15 cm^{-1}	84:	1172.00 cm^{-1}	108:	1584.29 cm^{-1}
61:	843.03 cm^{-1}	85:	1194.41 cm^{-1}	109:	1587.32 cm^{-1}
62:	872.13 cm^{-1}	86:	1221.91 cm^{-1}	110:	1598.38 cm^{-1}
63:	895.53 cm^{-1}	87:	1270.15 cm^{-1}	111:	1630.93 cm^{-1}
64:	897.04 cm^{-1}	88:	1279.20 cm^{-1}	112:	1661.91 cm^{-1}
65:	939.34 cm^{-1}	89:	1320.82 cm^{-1}	113:	1662.99 cm^{-1}
66:	954.09 cm^{-1}	90:	1334.53 cm^{-1}	114:	2996.42 cm^{-1}
67:	969.66 cm^{-1}	91:	1335.99 cm^{-1}	115:	2998.75 cm^{-1}
68:	971.98 cm^{-1}	92:	1346.03 cm^{-1}	116:	3060.62 cm^{-1}
69:	978.89 cm^{-1}	93:	1393.55 cm^{-1}	117:	3061.09 cm^{-1}
70:	979.91 cm^{-1}	94:	1394.68 cm^{-1}	118:	3077.03 cm^{-1}
71:	1004.82 cm^{-1}	95:	1417.94 cm^{-1}	119:	3078.87 cm^{-1}
72:	1012.23 cm^{-1}	96:	1431.38 cm^{-1}	120:	3163.30 cm^{-1}
73:	1020.10 cm^{-1}	97:	1435.58 cm^{-1}	121:	3168.87 cm^{-1}
74:	1045.45 cm^{-1}	98:	1437.18 cm^{-1}	122:	3171.44 cm^{-1}
75:	1046.11 cm^{-1}	99:	1439.20 cm^{-1}	123:	3172.04 cm^{-1}
76:	1063.33 cm^{-1}	100:	1454.17 cm^{-1}	124:	3176.48 cm^{-1}
77:	1124.27 cm^{-1}	101:	1476.36 cm^{-1}	125:	3176.74 cm^{-1}
78:	1131.22 cm^{-1}	102:	1500.31 cm^{-1}	126:	3182.17 cm^{-1}
79:	1133.46 cm^{-1}	103:	1503.48 cm^{-1}	127:	3193.47 cm^{-1}
80:	1137.50 cm^{-1}	104:	1526.44 cm^{-1}	128:	3201.00 cm^{-1}
81:	1148.71 cm^{-1}	105:	1533.87 cm^{-1}	129:	3201.67 cm^{-1}
82:	1150.20 cm^{-1}	106:	1555.01 cm^{-1}	130:	3222.11 cm^{-1}
83:	1167.35 cm^{-1}	107:	1567.09 cm^{-1}	131:	3222.65 cm^{-1}

This molecular structure was also optimized and the vibrational frequencies calculated using the BP86, the TPSS or the B3LYP density functional. The deviation from the results given above are negligible though. The xyz coordinates and vibrational frequencies can be found in the corresponding Orca output file within the deposited raw data.

[BPI–AlMe$_2$]$^{2–}$ ([**1**]$^{2–}$) – PBE0-D3BJ/def2-TZVP (T_1)

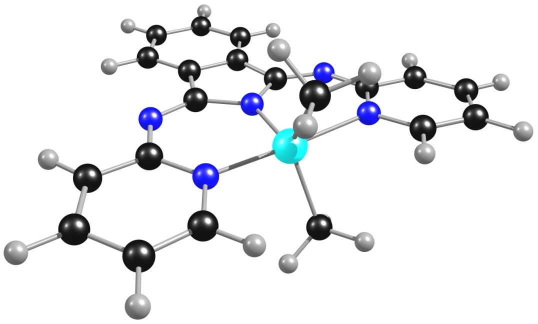

Al	2.686272000	9.689763000	5.416059000
N	3.240344000	8.265959000	4.239831000
N	0.863499000	9.689138000	4.566034000
N	5.512861000	7.687764000	5.013168000
N	1.233994000	8.087353000	2.832131000
N	4.707160000	9.558226000	6.244693000
C	6.870289000	8.604879000	6.704232000
C	5.351656000	5.653020000	2.741472000
H	6.309186000	5.566017000	3.243570000
C	2.435800000	7.769253000	3.215726000
C	7.107077000	9.535390000	7.671751000
H	8.041008000	9.521394000	8.228324000
C	5.018472000	4.849213000	1.667349000
H	5.730179000	4.107748000	1.311729000
C	4.443388000	7.566211000	4.229125000
C	2.845788000	5.912164000	1.457685000
H	1.878347000	6.027886000	0.980185000
C	5.651417000	8.605723000	5.957267000
C	3.174244000	6.719954000	2.532700000
C	3.774585000	4.969995000	1.020907000
H	3.541917000	4.323825000	0.179402000
C	4.417462000	6.600655000	3.181244000
C	0.437299000	9.013520000	3.443808000
C	3.065170000	11.589709000	4.823939000
H	3.968398000	12.068867000	5.218325000
H	2.215478000	12.260337000	5.002124000
H	3.181593000	11.539827000	3.731948000
C	-0.043417000	10.562474000	5.131868000

H	0.302347000	11.065249000	6.028770000
C	-1.293848000	10.806183000	4.635100000
H	-1.930770000	11.516510000	5.156453000
C	-0.820132000	9.223628000	2.900027000
C	2.021145000	9.197112000	7.268382000
H	0.931478000	9.096971000	7.316694000
H	2.327702000	9.888336000	8.064629000
H	2.453634000	8.222185000	7.532121000
C	4.968997000	10.455942000	7.190710000
H	4.177586000	11.182277000	7.354957000
C	6.125654000	10.514324000	7.936571000
H	6.258133000	11.280653000	8.690156000
C	-1.734341000	10.143820000	3.473547000
H	-2.721752000	10.305308000	3.056605000
H	7.590788000	7.834369000	6.455598000
H	-1.066576000	8.639571000	2.020784000

0:	0.00 cm^{-1}	20:	223.13 cm^{-1}	40:	572.85 cm^{-1}
1:	0.00 cm^{-1}	21:	229.86 cm^{-1}	41:	595.03 cm^{-1}
2:	0.00 cm^{-1}	22:	256.57 cm^{-1}	42:	599.43 cm^{-1}
3:	0.00 cm^{-1}	23:	266.49 cm^{-1}	43:	607.87 cm^{-1}
4:	0.00 cm^{-1}	24:	273.23 cm^{-1}	44:	627.15 cm^{-1}
5:	0.00 cm^{-1}	25:	280.29 cm^{-1}	45:	637.16 cm^{-1}
6:	32.60 cm^{-1}	26:	303.88 cm^{-1}	46:	654.99 cm^{-1}
7:	35.78 cm^{-1}	27:	310.68 cm^{-1}	47:	669.11 cm^{-1}
8:	55.68 cm^{-1}	28:	326.45 cm^{-1}	48:	677.26 cm^{-1}
9:	60.31 cm^{-1}	29:	363.83 cm^{-1}	49:	681.75 cm^{-1}
10:	78.62 cm^{-1}	30:	389.46 cm^{-1}	50:	690.43 cm^{-1}
11:	83.49 cm^{-1}	31:	394.17 cm^{-1}	51:	692.38 cm^{-1}
12:	101.23 cm^{-1}	32:	417.48 cm^{-1}	52:	703.19 cm^{-1}
13:	116.70 cm^{-1}	33:	446.17 cm^{-1}	53:	705.28 cm^{-1}
14:	139.61 cm^{-1}	34:	461.59 cm^{-1}	54:	709.55 cm^{-1}
15:	142.52 cm^{-1}	35:	508.15 cm^{-1}	55:	715.02 cm^{-1}
16:	150.12 cm^{-1}	36:	517.87 cm^{-1}	56:	719.87 cm^{-1}
17:	158.11 cm^{-1}	37:	530.42 cm^{-1}	57:	738.37 cm^{-1}
18:	206.10 cm^{-1}	38:	547.30 cm^{-1}	58:	757.74 cm^{-1}
19:	214.08 cm^{-1}	39:	569.30 cm^{-1}	59:	776.17 cm^{-1}

60:	782.04 cm^{-1}	84:	1178.09 cm^{-1}	108:	1560.85 cm^{-1}
61:	812.63 cm^{-1}	85:	1181.46 cm^{-1}	109:	1572.99 cm^{-1}
62:	816.03 cm^{-1}	86:	1207.89 cm^{-1}	110:	1593.45 cm^{-1}
63:	843.68 cm^{-1}	87:	1220.57 cm^{-1}	111:	1637.00 cm^{-1}
64:	865.61 cm^{-1}	88:	1261.97 cm^{-1}	112:	1639.28 cm^{-1}
65:	884.43 cm^{-1}	89:	1279.55 cm^{-1}	113:	1655.62 cm^{-1}
66:	887.77 cm^{-1}	90:	1308.19 cm^{-1}	114:	2995.96 cm^{-1}
67:	893.83 cm^{-1}	91:	1313.31 cm^{-1}	115:	2997.95 cm^{-1}
68:	911.08 cm^{-1}	92:	1344.67 cm^{-1}	116:	3060.04 cm^{-1}
69:	926.53 cm^{-1}	93:	1345.56 cm^{-1}	117:	3064.21 cm^{-1}
70:	936.14 cm^{-1}	94:	1376.75 cm^{-1}	118:	3081.01 cm^{-1}
71:	961.89 cm^{-1}	95:	1377.18 cm^{-1}	119:	3085.83 cm^{-1}
72:	1011.24 cm^{-1}	96:	1420.80 cm^{-1}	120:	3135.23 cm^{-1}
73:	1015.98 cm^{-1}	97:	1440.31 cm^{-1}	121:	3141.74 cm^{-1}
74:	1019.38 cm^{-1}	98:	1443.71 cm^{-1}	122:	3143.90 cm^{-1}
75:	1039.91 cm^{-1}	99:	1446.85 cm^{-1}	123:	3162.48 cm^{-1}
76:	1042.70 cm^{-1}	100:	1450.14 cm^{-1}	124:	3162.89 cm^{-1}
77:	1077.08 cm^{-1}	101:	1455.05 cm^{-1}	125:	3175.27 cm^{-1}
78:	1088.31 cm^{-1}	102:	1459.89 cm^{-1}	126:	3185.91 cm^{-1}
79:	1099.21 cm^{-1}	103:	1481.95 cm^{-1}	127:	3189.53 cm^{-1}
80:	1121.67 cm^{-1}	104:	1489.79 cm^{-1}	128:	3191.04 cm^{-1}
81:	1131.91 cm^{-1}	105:	1501.90 cm^{-1}	129:	3198.07 cm^{-1}
82:	1146.61 cm^{-1}	106:	1513.26 cm^{-1}	130:	3199.75 cm^{-1}
83:	1173.56 cm^{-1}	107:	1529.36 cm^{-1}	131:	3204.94 cm^{-1}

This molecular structure was also optimized and the vibrational frequencies calculated using the BP86, the TPSS or the B3LYP density functional. The deviation from the results given above are negligible though. The xyz coordinates and vibrational frequencies can be found in the corresponding Orca output file within the deposited raw data.

[BPI–AlMe$_2$]$^{2–}$ ([**1**]$^{2–}$) – PBE0-D3BJ/def2-TZVP/CPCM(THF) (T_1)

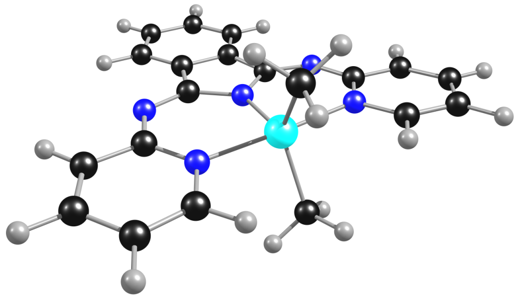

Al	2.763879000	9.663487000	5.435619000
N	3.241552000	8.272004000	4.241850000
N	0.865285000	9.696130000	4.555136000
N	5.513657000	7.699697000	5.001540000
N	1.231585000	8.096544000	2.828680000
N	4.689946000	9.555197000	6.247994000
C	6.865793000	8.633491000	6.695215000
C	5.365408000	5.636349000	2.740690000
H	6.328214000	5.541782000	3.229834000
C	2.457660000	7.744962000	3.205113000
C	7.090629000	9.561800000	7.668316000
H	8.024159000	9.560804000	8.221613000
C	5.014194000	4.809021000	1.643564000
H	5.723460000	4.066506000	1.288400000
C	4.449496000	7.559462000	4.216329000
C	2.830106000	5.881055000	1.445275000
H	1.868511000	5.972237000	0.953089000
C	5.646853000	8.619253000	5.954198000
C	3.170766000	6.704443000	2.530271000
C	3.788595000	4.927697000	1.016920000
H	3.554431000	4.276631000	0.179160000
C	4.422114000	6.583456000	3.170462000
C	0.474208000	9.005166000	3.438148000
C	3.047714000	11.584436000	4.852913000
H	4.093716000	11.911960000	4.886797000
H	2.471571000	12.310196000	5.443490000
H	2.709330000	11.707233000	3.815009000
C	0.010213000	10.561970000	5.114815000

H	0.381821000	11.065335000	6.002998000
C	-1.249792000	10.834345000	4.646281000
H	-1.883336000	11.549199000	5.154943000
C	-0.823297000	9.254666000	2.900366000
C	1.990676000	9.175416000	7.244766000
H	0.946339000	8.842608000	7.209453000
H	2.034388000	9.995954000	7.974417000
H	2.572329000	8.353241000	7.683454000
C	4.939088000	10.459888000	7.203691000
H	4.141888000	11.177508000	7.376267000
C	6.093769000	10.524825000	7.941073000
H	6.221101000	11.287789000	8.697920000
C	-1.670890000	10.148407000	3.485302000
H	-2.655660000	10.325694000	3.065275000
H	7.604831000	7.880103000	6.448189000
H	-1.106790000	8.700753000	2.012867000

0:	0.00 cm^{-1}	20:	232.53 cm^{-1}	40:	590.30 cm^{-1}
1:	0.00 cm^{-1}	21:	246.10 cm^{-1}	41:	600.18 cm^{-1}
2:	0.00 cm^{-1}	22:	250.50 cm^{-1}	42:	617.16 cm^{-1}
3:	0.00 cm^{-1}	23:	273.24 cm^{-1}	43:	632.88 cm^{-1}
4:	0.00 cm^{-1}	24:	281.50 cm^{-1}	44:	633.62 cm^{-1}
5:	0.00 cm^{-1}	25:	288.06 cm^{-1}	45:	646.33 cm^{-1}
6:	33.82 cm^{-1}	26:	298.83 cm^{-1}	46:	647.53 cm^{-1}
7:	43.58 cm^{-1}	27:	314.02 cm^{-1}	47:	661.86 cm^{-1}
8:	62.08 cm^{-1}	28:	362.02 cm^{-1}	48:	669.32 cm^{-1}
9:	80.65 cm^{-1}	29:	384.43 cm^{-1}	49:	677.20 cm^{-1}
10:	96.73 cm^{-1}	30:	390.92 cm^{-1}	50:	684.34 cm^{-1}
11:	110.22 cm^{-1}	31:	435.87 cm^{-1}	51:	694.69 cm^{-1}
12:	111.09 cm^{-1}	32:	450.24 cm^{-1}	52:	701.56 cm^{-1}
13:	121.18 cm^{-1}	33:	459.73 cm^{-1}	53:	713.54 cm^{-1}
14:	135.48 cm^{-1}	34:	515.06 cm^{-1}	54:	737.48 cm^{-1}
15:	143.35 cm^{-1}	35:	523.18 cm^{-1}	55:	738.55 cm^{-1}
16:	147.73 cm^{-1}	36:	537.30 cm^{-1}	56:	748.05 cm^{-1}
17:	158.75 cm^{-1}	37:	540.27 cm^{-1}	57:	752.80 cm^{-1}
18:	207.97 cm^{-1}	38:	564.70 cm^{-1}	58:	770.50 cm^{-1}
19:	221.06 cm^{-1}	39:	569.14 cm^{-1}	59:	781.16 cm^{-1}

60: 815.92 cm^{-1}	84: 1163.55 cm^{-1}	108: 1529.55 cm^{-1}
61: 840.00 cm^{-1}	85: 1174.04 cm^{-1}	109: 1578.41 cm^{-1}
62: 844.89 cm^{-1}	86: 1179.89 cm^{-1}	110: 1582.90 cm^{-1}
63: 880.48 cm^{-1}	87: 1257.70 cm^{-1}	111: 1588.03 cm^{-1}
64: 888.53 cm^{-1}	88: 1264.95 cm^{-1}	112: 1632.35 cm^{-1}
65: 892.92 cm^{-1}	89: 1310.73 cm^{-1}	113: 1635.45 cm^{-1}
66: 897.01 cm^{-1}	90: 1328.13 cm^{-1}	114: 2996.28 cm^{-1}
67: 970.00 cm^{-1}	91: 1329.61 cm^{-1}	115: 2999.36 cm^{-1}
68: 972.36 cm^{-1}	92: 1342.34 cm^{-1}	116: 3061.49 cm^{-1}
69: 978.42 cm^{-1}	93: 1352.51 cm^{-1}	117: 3064.83 cm^{-1}
70: 979.07 cm^{-1}	94: 1364.03 cm^{-1}	118: 3076.95 cm^{-1}
71: 1007.47 cm^{-1}	95: 1431.68 cm^{-1}	119: 3078.48 cm^{-1}
72: 1007.86 cm^{-1}	96: 1433.35 cm^{-1}	120: 3154.48 cm^{-1}
73: 1022.41 cm^{-1}	97: 1433.68 cm^{-1}	121: 3172.06 cm^{-1}
74: 1039.76 cm^{-1}	98: 1434.92 cm^{-1}	122: 3173.41 cm^{-1}
75: 1044.05 cm^{-1}	99: 1435.78 cm^{-1}	123: 3173.61 cm^{-1}
76: 1050.45 cm^{-1}	100: 1439.85 cm^{-1}	124: 3182.59 cm^{-1}
77: 1071.43 cm^{-1}	101: 1452.29 cm^{-1}	125: 3182.76 cm^{-1}
78: 1126.01 cm^{-1}	102: 1461.25 cm^{-1}	126: 3193.92 cm^{-1}
79: 1130.03 cm^{-1}	103: 1464.18 cm^{-1}	127: 3197.15 cm^{-1}
80: 1138.53 cm^{-1}	104: 1486.22 cm^{-1}	128: 3206.22 cm^{-1}
81: 1146.89 cm^{-1}	105: 1498.87 cm^{-1}	129: 3206.40 cm^{-1}
82: 1147.85 cm^{-1}	106: 1510.11 cm^{-1}	130: 3223.23 cm^{-1}
83: 1154.47 cm^{-1}	107: 1526.32 cm^{-1}	131: 3223.97 cm^{-1}

This molecular structure was also optimized and the vibrational frequencies calculated using the BP86, the TPSS or the B3LYP density functional. The deviation from the results given above are negligible though. The xyz coordinates and vibrational frequencies can be found in the corresponding Orca output file within the deposited raw data.

[BPI–AlMe]$^{2-}$ (**9**)$^{2-}$ – PBE0-D3BJ/def2-TZVP (D_1)

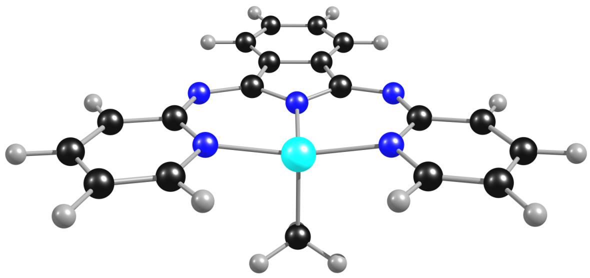

Al	2.837198000	9.802340000	5.276726000
N	3.256039000	8.281997000	4.179696000
N	0.913607000	9.744926000	4.597344000
N	5.555774000	7.818636000	4.947249000
N	1.159256000	8.090461000	2.891406000
N	4.572230000	9.514420000	6.312860000
C	6.823744000	8.724241000	6.708022000
C	5.373039000	5.730008000	2.662990000
H	6.352054000	5.676637000	3.129822000
C	2.435546000	7.755508000	3.201570000
C	6.964294000	9.565592000	7.765107000
H	7.893453000	9.582686000	8.330720000
C	5.030581000	4.900936000	1.620580000
H	5.747347000	4.173500000	1.247269000
C	4.472879000	7.629603000	4.154218000
C	2.811542000	5.888234000	1.465382000
H	1.826267000	5.956284000	1.013864000
C	5.608781000	8.663980000	5.939456000
C	3.143269000	6.744965000	2.526221000
C	3.742955000	4.980471000	1.018569000
H	3.499004000	4.312397000	0.196102000
C	4.437183000	6.665057000	3.131175000
C	0.452976000	8.984890000	3.526276000
C	3.363331000	11.539537000	4.388346000
H	4.113452000	12.097748000	4.960386000
H	2.507809000	12.202006000	4.212987000
H	3.805455000	11.313005000	3.406791000
C	0.079612000	10.617147000	5.178473000
H	0.522494000	11.139966000	6.028133000

C	-1.214243000	10.857315000	4.797967000
H	-1.818972000	11.582039000	5.329795000
C	-0.901544000	9.204290000	3.093138000
C	4.744716000	10.321030000	7.368117000
H	3.865025000	10.923817000	7.601046000
C	5.882612000	10.409841000	8.125708000
H	5.932473000	11.091324000	8.966455000
C	-1.713134000	10.107704000	3.701563000
H	-2.734994000	10.245675000	3.354718000
H	7.618614000	8.058408000	6.391247000
H	-1.233147000	8.605770000	2.252122000

0:	0.00 cm^{-1}	25:	374.64 cm^{-1}	50:	727.52 cm^{-1}
1:	0.00 cm^{-1}	26:	388.11 cm^{-1}	51:	740.00 cm^{-1}
2:	0.00 cm^{-1}	27:	403.44 cm^{-1}	52:	799.47 cm^{-1}
3:	0.00 cm^{-1}	28:	433.42 cm^{-1}	53:	801.17 cm^{-1}
4:	0.00 cm^{-1}	29:	442.57 cm^{-1}	54:	806.75 cm^{-1}
5:	0.00 cm^{-1}	30:	444.58 cm^{-1}	55:	825.49 cm^{-1}
6:	25.24 cm^{-1}	31:	513.37 cm^{-1}	56:	855.66 cm^{-1}
7:	44.38 cm^{-1}	32:	518.66 cm^{-1}	57:	880.42 cm^{-1}
8:	52.98 cm^{-1}	33:	535.89 cm^{-1}	58:	882.90 cm^{-1}
9:	55.62 cm^{-1}	34:	559.16 cm^{-1}	59:	893.15 cm^{-1}
10:	65.96 cm^{-1}	35:	577.45 cm^{-1}	60:	899.24 cm^{-1}
11:	95.90 cm^{-1}	36:	582.44 cm^{-1}	61:	920.96 cm^{-1}
12:	117.28 cm^{-1}	37:	593.25 cm^{-1}	62:	922.00 cm^{-1}
13:	139.25 cm^{-1}	38:	618.48 cm^{-1}	63:	959.27 cm^{-1}
14:	141.50 cm^{-1}	39:	637.95 cm^{-1}	64:	963.37 cm^{-1}
15:	152.84 cm^{-1}	40:	647.09 cm^{-1}	65:	1004.61 cm^{-1}
16:	190.78 cm^{-1}	41:	661.59 cm^{-1}	66:	1007.00 cm^{-1}
17:	218.77 cm^{-1}	42:	668.17 cm^{-1}	67:	1014.08 cm^{-1}
18:	222.58 cm^{-1}	43:	670.58 cm^{-1}	68:	1030.59 cm^{-1}
19:	245.14 cm^{-1}	44:	677.58 cm^{-1}	69:	1031.93 cm^{-1}
20:	258.62 cm^{-1}	45:	695.08 cm^{-1}	70:	1071.84 cm^{-1}
21:	279.33 cm^{-1}	46:	699.56 cm^{-1}	71:	1119.70 cm^{-1}
22:	292.58 cm^{-1}	47:	710.77 cm^{-1}	72:	1122.39 cm^{-1}
23:	336.07 cm^{-1}	48:	719.72 cm^{-1}	73:	1124.65 cm^{-1}
24:	340.22 cm^{-1}	49:	721.75 cm^{-1}	74:	1135.25 cm^{-1}

75: 1141.32 cm^{-1}	90: 1446.68 cm^{-1}	105: 2988.21 cm^{-1}
76: 1143.60 cm^{-1}	91: 1457.44 cm^{-1}	106: 3061.33 cm^{-1}
77: 1159.97 cm^{-1}	92: 1476.32 cm^{-1}	107: 3084.64 cm^{-1}
78: 1201.17 cm^{-1}	93: 1492.69 cm^{-1}	108: 3088.35 cm^{-1}
79: 1227.83 cm^{-1}	94: 1498.07 cm^{-1}	109: 3090.98 cm^{-1}
80: 1247.03 cm^{-1}	95: 1526.12 cm^{-1}	110: 3131.27 cm^{-1}
81: 1256.39 cm^{-1}	96: 1537.34 cm^{-1}	111: 3136.23 cm^{-1}
82: 1317.29 cm^{-1}	97: 1550.51 cm^{-1}	112: 3136.87 cm^{-1}
83: 1338.77 cm^{-1}	98: 1558.81 cm^{-1}	113: 3153.10 cm^{-1}
84: 1340.79 cm^{-1}	99: 1592.11 cm^{-1}	114: 3176.00 cm^{-1}
85: 1359.36 cm^{-1}	100: 1593.91 cm^{-1}	115: 3181.18 cm^{-1}
86: 1386.87 cm^{-1}	101: 1606.12 cm^{-1}	116: 3189.76 cm^{-1}
87: 1388.00 cm^{-1}	102: 1629.50 cm^{-1}	117: 3191.47 cm^{-1}
88: 1416.48 cm^{-1}	103: 1652.90 cm^{-1}	118: 3194.02 cm^{-1}
89: 1443.83 cm^{-1}	104: 1653.88 cm^{-1}	119: 3196.15 cm^{-1}

This molecular structure was also optimized and the vibrational frequencies calculated using the BP86, the TPSS or the B3LYP density functional. The deviation from the results given above are negligible though. The xyz coordinates and vibrational frequencies can be found in the corresponding Orca output file within the deposited raw data.

[BPI–AlMe]$^{2–}$ (**9**)$^{2–}$ – PBE0-D3BJ/def2-TZVP/CPCM(THF) (D_1)

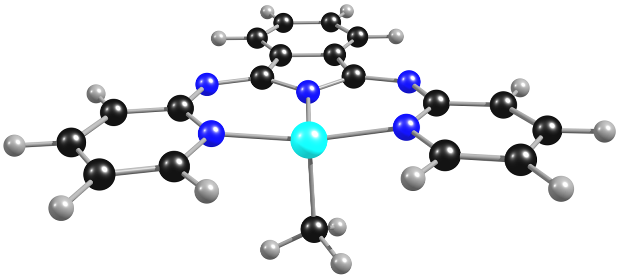

Al	2.708431000	9.514270000	5.539960000
N	3.239895000	8.209512000	4.243253000
N	0.959403000	9.790053000	4.538163000
N	5.535766000	7.753717000	5.028245000
N	1.172906000	8.073842000	2.899923000
N	4.618312000	9.585119000	6.245345000
C	6.863161000	8.806131000	6.667856000
C	5.357186000	5.635210000	2.755278000
H	6.332344000	5.558679000	3.227209000
C	2.430754000	7.697841000	3.247011000
C	7.042230000	9.756743000	7.622304000
H	7.985716000	9.825227000	8.154989000
C	5.020234000	4.813026000	1.707977000
H	5.732039000	4.077807000	1.345975000
C	4.453233000	7.548745000	4.233965000
C	2.815269000	5.824963000	1.512607000
H	1.843511000	5.894470000	1.032602000
C	5.629418000	8.691651000	5.940333000
C	3.136822000	6.676665000	2.586172000
C	3.743932000	4.908417000	1.083761000
H	3.508354000	4.244521000	0.257924000
C	4.420990000	6.581194000	3.213940000
C	0.493706000	9.036883000	3.476123000
C	0.167031000	10.744970000	5.059444000
H	0.619161000	11.296168000	5.883904000
C	-1.092685000	11.045618000	4.624990000
H	-1.666591000	11.831261000	5.099098000
C	-0.820017000	9.331441000	2.974334000
C	1.938803000	8.650059000	7.194633000

H	0.855803000	8.483124000	7.145317000
H	2.136577000	9.218011000	8.112346000
H	2.408772000	7.665072000	7.334582000
C	4.834506000	10.519390000	7.189485000
H	3.992812000	11.193770000	7.347157000
C	5.988831000	10.660709000	7.906551000
H	6.083384000	11.440511000	8.651091000
C	-1.593907000	10.301645000	3.527956000
H	-2.582917000	10.505871000	3.129592000
H	7.645712000	8.098086000	6.419316000
H	-1.167528000	8.739409000	2.135130000

0:	0.00 cm^{-1}	25:	375.78 cm^{-1}	50:	765.75 cm^{-1}
1:	0.00 cm^{-1}	26:	390.35 cm^{-1}	51:	779.05 cm^{-1}
2:	0.00 cm^{-1}	27:	398.64 cm^{-1}	52:	823.11 cm^{-1}
3:	0.00 cm^{-1}	28:	439.74 cm^{-1}	53:	832.81 cm^{-1}
4:	0.00 cm^{-1}	29:	442.82 cm^{-1}	54:	837.78 cm^{-1}
5:	0.00 cm^{-1}	30:	445.29 cm^{-1}	55:	843.14 cm^{-1}
6:	29.84 cm^{-1}	31:	532.76 cm^{-1}	56:	861.58 cm^{-1}
7:	45.07 cm^{-1}	32:	537.28 cm^{-1}	57:	887.55 cm^{-1}
8:	57.02 cm^{-1}	33:	538.35 cm^{-1}	58:	897.98 cm^{-1}
9:	70.69 cm^{-1}	34:	558.15 cm^{-1}	59:	940.29 cm^{-1}
10:	91.70 cm^{-1}	35:	578.59 cm^{-1}	60:	954.71 cm^{-1}
11:	114.92 cm^{-1}	36:	593.76 cm^{-1}	61:	966.04 cm^{-1}
12:	122.12 cm^{-1}	37:	620.35 cm^{-1}	62:	969.26 cm^{-1}
13:	141.51 cm^{-1}	38:	626.02 cm^{-1}	63:	978.83 cm^{-1}
14:	148.91 cm^{-1}	39:	636.46 cm^{-1}	64:	979.23 cm^{-1}
15:	162.61 cm^{-1}	40:	649.32 cm^{-1}	65:	1005.84 cm^{-1}
16:	205.95 cm^{-1}	41:	662.53 cm^{-1}	66:	1007.63 cm^{-1}
17:	222.15 cm^{-1}	42:	689.81 cm^{-1}	67:	1016.58 cm^{-1}
18:	239.15 cm^{-1}	43:	696.83 cm^{-1}	68:	1040.79 cm^{-1}
19:	255.85 cm^{-1}	44:	707.23 cm^{-1}	69:	1041.58 cm^{-1}
20:	266.46 cm^{-1}	45:	721.36 cm^{-1}	70:	1070.52 cm^{-1}
21:	279.01 cm^{-1}	46:	736.22 cm^{-1}	71:	1125.99 cm^{-1}
22:	304.96 cm^{-1}	47:	736.91 cm^{-1}	72:	1129.91 cm^{-1}
23:	337.41 cm^{-1}	48:	745.02 cm^{-1}	73:	1132.42 cm^{-1}
24:	345.10 cm^{-1}	49:	753.32 cm^{-1}	74:	1137.08 cm^{-1}

75: 1147.48 cm^{-1}	90: 1451.28 cm^{-1}	105: 2992.12 cm^{-1}
76: 1149.08 cm^{-1}	91: 1458.07 cm^{-1}	106: 3061.21 cm^{-1}
77: 1184.79 cm^{-1}	92: 1473.04 cm^{-1}	107: 3079.01 cm^{-1}
78: 1196.73 cm^{-1}	93: 1493.29 cm^{-1}	108: 3117.95 cm^{-1}
79: 1226.58 cm^{-1}	94: 1495.70 cm^{-1}	109: 3120.58 cm^{-1}
80: 1260.24 cm^{-1}	95: 1522.21 cm^{-1}	110: 3164.28 cm^{-1}
81: 1270.09 cm^{-1}	96: 1535.43 cm^{-1}	111: 3172.30 cm^{-1}
82: 1319.50 cm^{-1}	97: 1552.24 cm^{-1}	112: 3177.42 cm^{-1}
83: 1326.41 cm^{-1}	98: 1564.39 cm^{-1}	113: 3177.49 cm^{-1}
84: 1328.65 cm^{-1}	99: 1583.27 cm^{-1}	114: 3183.06 cm^{-1}
85: 1350.73 cm^{-1}	100: 1585.47 cm^{-1}	115: 3194.25 cm^{-1}
86: 1390.25 cm^{-1}	101: 1593.13 cm^{-1}	116: 3200.59 cm^{-1}
87: 1392.58 cm^{-1}	102: 1632.06 cm^{-1}	117: 3201.91 cm^{-1}
88: 1415.79 cm^{-1}	103: 1660.68 cm^{-1}	118: 3219.15 cm^{-1}
89: 1428.01 cm^{-1}	104: 1661.34 cm^{-1}	119: 3220.04 cm^{-1}

This molecular structure was also optimized and the vibrational frequencies calculated using the BP86, the TPSS or the B3LYP density functional. The deviation from the results given above are negligible though. The xyz coordinates and vibrational frequencies can be found in the corresponding Orca output file within the deposited raw data.

[CH_3]$^{\bullet}$ – PBE0-D3BJ/def2-TZVP (D_1)

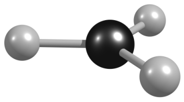

C	1.880223000	9.053569000	7.517038000
H	0.933888000	8.805985000	7.059253000
H	2.042927000	10.042312000	7.919942000
H	2.663894000	8.312442000	7.571849000

0:	0.00 cm^{-1}	4:	0.00 cm^{-1}	8:	1400.70 cm^{-1}
1:	0.00 cm^{-1}	5:	0.00 cm^{-1}	9:	3128.33 cm^{-1}
2:	0.00 cm^{-1}	6:	526.45 cm^{-1}	10:	3311.09 cm^{-1}
3:	0.00 cm^{-1}	7:	1400.64 cm^{-1}	11:	3311.18 cm^{-1}

This molecular structure was also optimized and the vibrational frequencies calculated using the BP86, the TPSS or the B3LYP density functional. The deviation from the results given above are negligible though. The xyz coordinates and vibrational frequencies can be found in the corresponding Orca output file within the deposited raw data.

[CH_3]$^{\bullet}$ – PBE0-D3BJ/def2-TZVP/CPCM(THF) (D_1)

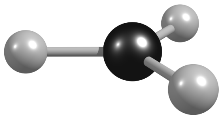

C	1.880236000	9.053578000	7.517046000
H	0.932231000	8.805530000	7.058501000
H	2.043207000	10.044068000	7.920626000
H	2.665260000	8.311132000	7.571910000

0:	0.00 cm^{-1}	4:	0.00 cm^{-1}	8:	1379.41 cm^{-1}
1:	0.00 cm^{-1}	5:	0.00 cm^{-1}	9:	3114.40 cm^{-1}
2:	0.00 cm^{-1}	6:	530.95 cm^{-1}	10:	3298.66 cm^{-1}
3:	0.00 cm^{-1}	7:	1378.21 cm^{-1}	11:	3298.70 cm^{-1}

This molecular structure was also optimized and the vibrational frequencies calculated using the BP86, the TPSS or the B3LYP density functional. The deviation from the results given above are negligible though. The xyz coordinates and vibrational frequencies can be found in the corresponding Orca output file within the deposited raw data.

[CH_3]$^{–}$ – PBE0-D3BJ/def2-TZVP (S_0)

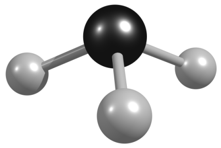

C	2.000107000	9.157678000	7.213500000
H	0.951021000	8.786369000	7.187255000
H	1.993083000	9.948155000	7.997227000
H	2.576720000	8.322108000	7.670099000

0:	0.00 cm^{-1}	4:	0.00 cm^{-1}	8:	1433.82 cm^{-1}
1:	0.00 cm^{-1}	5:	0.00 cm^{-1}	9:	2841.44 cm^{-1}
2:	0.00 cm^{-1}	6:	938.65 cm^{-1}	10:	2901.67 cm^{-1}
3:	0.00 cm^{-1}	7:	1433.44 cm^{-1}	11:	2902.61 cm^{-1}

This molecular structure was also optimized and the vibrational frequencies calculated using the BP86, the TPSS or the B3LYP density functional. The deviation from the results given above are negligible though. The xyz coordinates and vibrational frequencies can be found in the corresponding Orca output file within the deposited raw data.

[CH_3]$^{–}$ – PBE0-D3BJ/def2-TZVP/CPCM(THF) (S_0)

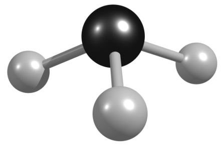

C	1.993959000	9.152214000	7.228923000
H	0.951768000	8.787820000	7.181441000
H	1.995334000	9.951449000	7.992730000
H	2.579870000	8.322829000	7.664987000

0:	0.00 cm^{-1}	4:	0.00 cm^{-1}	8:	1428.42 cm^{-1}
1:	0.00 cm^{-1}	5:	0.00 cm^{-1}	9:	2927.54 cm^{-1}
2:	0.00 cm^{-1}	6:	953.83 cm^{-1}	10:	2991.37 cm^{-1}
3:	0.00 cm^{-1}	7:	1416.14 cm^{-1}	11:	2996.09 cm^{-1}

This molecular structure was also optimized and the vibrational frequencies calculated using the BP86, the TPSS or the B3LYP density functional. The deviation from the results given above are negligible though. The xyz coordinates and vibrational frequencies can be found in the corresponding Orca output file within the deposited raw data.

Al_2Me_6 – PBE0-D3BJ/def2-TZVP (S_0)

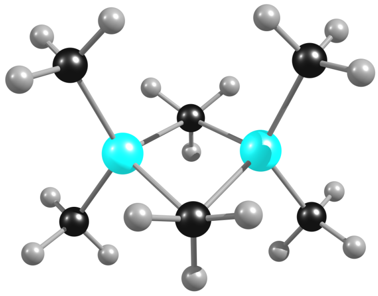

Al	-1.109749000	-0.135361000	0.101555000
C	-1.217186000	1.993870000	0.191190000
Al	0.850551000	1.536828000	-0.071456000
C	0.971919000	-0.561299000	0.298162000
C	1.364158000	1.898835000	-1.927253000
C	1.738906000	2.362292000	1.464090000
C	-1.900343000	-0.741966000	1.785431000
C	-1.732655000	-0.742858000	-1.653950000
H	-1.169303000	-0.295014000	-2.477924000
H	-2.787756000	-0.496696000	-1.813269000
H	-1.645908000	-1.829622000	-1.757077000
H	-2.965298000	-0.494513000	1.840105000
H	-1.416081000	-0.290138000	2.656303000
H	-1.822211000	-1.828202000	1.896414000
H	0.754225000	1.345949000	-2.647785000
H	2.408809000	1.629085000	-2.114821000
H	1.266980000	2.962003000	-2.171064000
H	1.662204000	3.453824000	1.432162000
H	2.806428000	2.120972000	1.486582000
H	1.310790000	2.033659000	2.415822000
H	0.641416000	-1.526825000	-0.109679000
H	1.195164000	-0.713333000	1.355738000
H	1.929850000	-0.428117000	-0.223779000
H	-0.916840000	2.893754000	-0.363580000
H	-1.375748000	2.288799000	1.230084000
H	-2.204803000	1.794643000	-0.247832000

0:	0.00 cm^{-1}	2:	0.00 cm^{-1}	4:	0.00 cm^{-1}
1:	0.00 cm^{-1}	3:	0.00 cm^{-1}	5:	0.00 cm^{-1}

6:	57.72 cm^{-1}
7:	90.42 cm^{-1}
8:	106.45 cm^{-1}
9:	114.53 cm^{-1}
10:	114.81 cm^{-1}
11:	117.70 cm^{-1}
12:	138.09 cm^{-1}
13:	139.95 cm^{-1}
14:	160.15 cm^{-1}
15:	161.84 cm^{-1}
16:	170.77 cm^{-1}
17:	176.00 cm^{-1}
18:	179.34 cm^{-1}
19:	180.07 cm^{-1}
20:	204.03 cm^{-1}
21:	325.19 cm^{-1}
22:	326.61 cm^{-1}
23:	365.89 cm^{-1}
24:	463.96 cm^{-1}
25:	497.61 cm^{-1}
26:	565.87 cm^{-1}
27:	569.30 cm^{-1}
28:	588.91 cm^{-1}
29:	591.27 cm^{-1}
30:	598.70 cm^{-1}
31:	600.95 cm^{-1}
32:	623.06 cm^{-1}
33:	623.78 cm^{-1}
34:	652.94 cm^{-1}
35:	694.77 cm^{-1}
36:	695.82 cm^{-1}
37:	712.45 cm^{-1}
38:	712.83 cm^{-1}
39:	720.55 cm^{-1}
40:	738.11 cm^{-1}
41:	784.88 cm^{-1}
42:	1219.56 cm^{-1}
43:	1220.83 cm^{-1}
44:	1221.49 cm^{-1}
45:	1226.35 cm^{-1}
46:	1268.26 cm^{-1}
47:	1271.20 cm^{-1}
48:	1436.52 cm^{-1}
49:	1436.85 cm^{-1}
50:	1437.20 cm^{-1}
51:	1437.39 cm^{-1}
52:	1440.01 cm^{-1}
53:	1444.46 cm^{-1}
54:	1445.91 cm^{-1}
55:	1446.85 cm^{-1}
56:	1450.82 cm^{-1}
57:	1452.37 cm^{-1}
58:	1458.03 cm^{-1}
59:	1459.97 cm^{-1}
60:	3015.33 cm^{-1}
61:	3015.62 cm^{-1}
62:	3030.60 cm^{-1}
63:	3031.94 cm^{-1}
64:	3033.15 cm^{-1}
65:	3033.60 cm^{-1}
66:	3065.19 cm^{-1}
67:	3066.53 cm^{-1}
68:	3104.46 cm^{-1}
69:	3104.62 cm^{-1}
70:	3109.43 cm^{-1}
71:	3109.79 cm^{-1}
72:	3113.51 cm^{-1}
73:	3114.64 cm^{-1}
74:	3114.78 cm^{-1}
75:	3116.52 cm^{-1}
76:	3135.59 cm^{-1}
77:	3135.78 cm^{-1}

This molecular structure was also optimized and the vibrational frequencies calculated using the BP86, the TPSS or the B3LYP density functional. The deviation from the results given above are negligible though. The xyz coordinates and vibrational frequencies can be found in the corresponding Orca output file within the deposited raw data.

Al$_2$Me$_6$ – PBE0-D3BJ/def2-TZVP/CPCM(THF) (S_0)

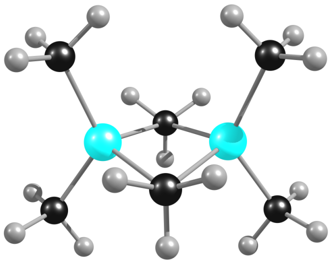

Al	-1.106954000	-0.134776000	0.102324000
C	-1.211998000	1.987118000	0.200152000
Al	0.850555000	1.537009000	-0.067063000
C	0.969046000	-0.554492000	0.302085000
C	1.364930000	1.900084000	-1.927180000
C	1.754081000	2.375013000	1.456848000
C	-1.914955000	-0.753234000	1.777827000
C	-1.735983000	-0.745881000	-1.653825000
H	-1.154979000	-0.312865000	-2.475442000
H	-2.785413000	-0.476585000	-1.820755000
H	-1.667155000	-1.836047000	-1.746274000
H	-2.981716000	-0.503618000	1.815742000
H	-1.443504000	-0.306455000	2.659538000
H	-1.835837000	-1.841622000	1.879730000
H	0.746801000	1.345384000	-2.641683000
H	2.408198000	1.621381000	-2.116280000
H	1.268985000	2.964261000	-2.171958000
H	1.670653000	3.467110000	1.415261000
H	2.823440000	2.134183000	1.462736000
H	1.340759000	2.051407000	2.417916000
H	0.659796000	-1.518741000	-0.126559000
H	1.171250000	-0.719990000	1.363262000
H	1.941506000	-0.408432000	-0.190915000
H	-0.927937000	2.888021000	-0.363078000
H	-1.357200000	2.287392000	1.240814000
H	-2.208848000	1.784945000	-0.219054000

0:	0.00 cm^{-1}	3:	0.00 cm^{-1}	6:	53.73 cm^{-1}
1:	0.00 cm^{-1}	4:	0.00 cm^{-1}	7:	86.45 cm^{-1}
2:	0.00 cm^{-1}	5:	0.00 cm^{-1}	8:	100.94 cm^{-1}

9:	114.59 cm^{-1}	32:	614.09 cm^{-1}	55:	1424.47 cm^{-1}
10:	117.70 cm^{-1}	33:	620.79 cm^{-1}	56:	1424.96 cm^{-1}
11:	128.26 cm^{-1}	34:	645.82 cm^{-1}	57:	1428.37 cm^{-1}
12:	130.10 cm^{-1}	35:	668.58 cm^{-1}	58:	1429.54 cm^{-1}
13:	133.51 cm^{-1}	36:	678.13 cm^{-1}	59:	1430.86 cm^{-1}
14:	159.62 cm^{-1}	37:	688.88 cm^{-1}	60:	3012.44 cm^{-1}
15:	160.31 cm^{-1}	38:	695.48 cm^{-1}	61:	3014.17 cm^{-1}
16:	163.11 cm^{-1}	39:	696.49 cm^{-1}	62:	3020.94 cm^{-1}
17:	178.02 cm^{-1}	40:	707.17 cm^{-1}	63:	3021.31 cm^{-1}
18:	182.54 cm^{-1}	41:	749.35 cm^{-1}	64:	3023.64 cm^{-1}
19:	194.02 cm^{-1}	42:	1190.05 cm^{-1}	65:	3023.88 cm^{-1}
20:	209.69 cm^{-1}	43:	1192.47 cm^{-1}	66:	3063.16 cm^{-1}
21:	325.09 cm^{-1}	44:	1195.58 cm^{-1}	67:	3065.50 cm^{-1}
22:	330.57 cm^{-1}	45:	1197.11 cm^{-1}	68:	3097.24 cm^{-1}
23:	360.64 cm^{-1}	46:	1257.87 cm^{-1}	69:	3097.48 cm^{-1}
24:	466.08 cm^{-1}	47:	1260.12 cm^{-1}	70:	3100.40 cm^{-1}
25:	487.40 cm^{-1}	48:	1415.13 cm^{-1}	71:	3100.56 cm^{-1}
26:	557.98 cm^{-1}	49:	1418.14 cm^{-1}	72:	3102.90 cm^{-1}
27:	576.99 cm^{-1}	50:	1419.90 cm^{-1}	73:	3104.20 cm^{-1}
28:	586.52 cm^{-1}	51:	1420.44 cm^{-1}	74:	3105.97 cm^{-1}
29:	591.00 cm^{-1}	52:	1421.44 cm^{-1}	75:	3106.79 cm^{-1}
30:	596.28 cm^{-1}	53:	1422.08 cm^{-1}	76:	3127.23 cm^{-1}
31:	597.72 cm^{-1}	54:	1423.40 cm^{-1}	77:	3127.70 cm^{-1}

This molecular structure was also optimized and the vibrational frequencies calculated using the BP86, the TPSS or the B3LYP density functional. The deviation from the results given above are negligible though. The xyz coordinates and vibrational frequencies can be found in the corresponding Orca output file within the deposited raw data.

[Al_2Me_5]$^{\bullet}$ – PBE0-D3BJ/def2-TZVP (D_1)

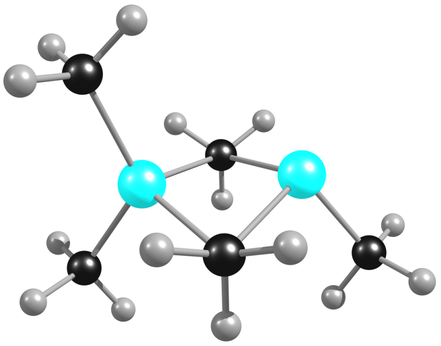

Al	-1.093451000	-0.123885000	0.050622000
C	-1.198176000	2.012516000	0.265887000
Al	0.862199000	1.546892000	-0.068359000
C	0.990380000	-0.541793000	0.373129000
C	1.324984000	1.865068000	-1.943942000
C	1.790114000	2.404243000	1.426190000
C	-1.959318000	-0.796839000	1.682871000
H	-3.024234000	-0.549472000	1.702004000
H	-1.498379000	-0.362719000	2.576708000
H	-1.878361000	-1.884578000	1.759461000
H	0.691914000	1.294743000	-2.630274000
H	2.363514000	1.588234000	-2.153996000
H	1.221446000	2.922501000	-2.209305000
H	1.714357000	3.494847000	1.369800000
H	2.857448000	2.161044000	1.425073000
H	1.387818000	2.099666000	2.396906000
H	0.691587000	-1.535608000	0.013877000
H	1.182434000	-0.630070000	1.445496000
H	1.959391000	-0.414165000	-0.130470000
H	-0.901703000	2.925444000	-0.270356000
H	-1.300570000	2.267228000	1.323849000
H	-2.210253000	1.851464000	-0.128783000

0:	0.00 cm^{-1}	5:	0.00 cm^{-1}	10:	120.37 cm^{-1}
1:	0.00 cm^{-1}	6:	52.62 cm^{-1}	11:	133.63 cm^{-1}
2:	0.00 cm^{-1}	7:	102.24 cm^{-1}	12:	158.10 cm^{-1}
3:	0.00 cm^{-1}	8:	111.06 cm^{-1}	13:	163.13 cm^{-1}
4:	0.00 cm^{-1}	9:	113.70 cm^{-1}	14:	169.35 cm^{-1}

15:	170.20 cm^{-1}	32:	692.39 cm^{-1}	49:	1455.89 cm^{-1}
16:	174.62 cm^{-1}	33:	713.96 cm^{-1}	50:	1456.52 cm^{-1}
17:	195.76 cm^{-1}	34:	725.56 cm^{-1}	51:	3010.05 cm^{-1}
18:	306.63 cm^{-1}	35:	764.51 cm^{-1}	52:	3010.89 cm^{-1}
19:	322.93 cm^{-1}	36:	1205.29 cm^{-1}	53:	3029.88 cm^{-1}
20:	356.87 cm^{-1}	37:	1220.50 cm^{-1}	54:	3031.27 cm^{-1}
21:	455.35 cm^{-1}	38:	1221.98 cm^{-1}	55:	3033.30 cm^{-1}
22:	492.86 cm^{-1}	39:	1259.16 cm^{-1}	56:	3062.57 cm^{-1}
23:	568.07 cm^{-1}	40:	1262.30 cm^{-1}	57:	3065.20 cm^{-1}
24:	577.43 cm^{-1}	41:	1425.43 cm^{-1}	58:	3104.49 cm^{-1}
25:	590.33 cm^{-1}	42:	1430.58 cm^{-1}	59:	3109.77 cm^{-1}
26:	601.22 cm^{-1}	43:	1434.71 cm^{-1}	60:	3112.55 cm^{-1}
27:	605.76 cm^{-1}	44:	1436.90 cm^{-1}	61:	3113.30 cm^{-1}
28:	624.82 cm^{-1}	45:	1441.33 cm^{-1}	62:	3115.18 cm^{-1}
29:	634.48 cm^{-1}	46:	1442.59 cm^{-1}	63:	3120.19 cm^{-1}
30:	660.56 cm^{-1}	47:	1443.71 cm^{-1}	64:	3120.85 cm^{-1}
31:	683.83 cm^{-1}	48:	1446.92 cm^{-1}	65:	3123.57 cm^{-1}

This molecular structure was also optimized and the vibrational frequencies calculated using the BP86, the TPSS or the B3LYP density functional. The deviation from the results given above are negligible though. The xyz coordinates and vibrational frequencies can be found in the corresponding Orca output file within the deposited raw data.

$[Al_2Me_5]^{\bullet}$ – PBE0-D3BJ/def2-TZVP/CPCM(THF) (D_1)

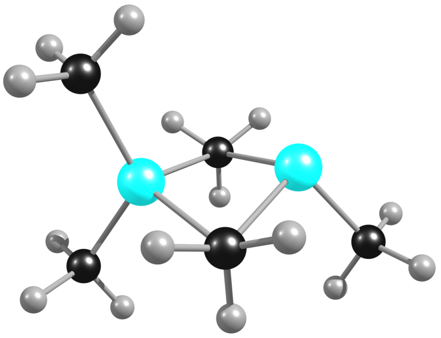

Al	-1.089335000	-0.116579000	0.048783000
C	-1.188082000	2.009525000	0.283697000
Al	0.865237000	1.547211000	-0.057042000
C	0.985964000	-0.533270000	0.384782000
C	1.319258000	1.856616000	-1.941982000
C	1.815914000	2.424507000	1.415097000
C	-1.979827000	-0.810350000	1.661961000
H	-3.046312000	-0.562197000	1.662677000
H	-1.536734000	-0.385814000	2.570469000
H	-1.895803000	-1.900073000	1.725630000
H	0.680777000	1.276335000	-2.617324000
H	2.357632000	1.577101000	-2.155527000
H	1.208458000	2.912484000	-2.215093000
H	1.730507000	3.514984000	1.344994000
H	2.884954000	2.183406000	1.392483000
H	1.434495000	2.128711000	2.397943000
H	0.707019000	-1.523252000	-0.002449000
H	1.145169000	-0.639356000	1.462055000
H	1.973428000	-0.393170000	-0.080469000
H	-0.914343000	2.927919000	-0.256364000
H	-1.278398000	2.262141000	1.344482000
H	-2.206837000	1.837881000	-0.092418000

0:	0.00 cm^{-1}	6:	56.27 cm^{-1}	12:	154.81 cm^{-1}
1:	0.00 cm^{-1}	7:	96.79 cm^{-1}	13:	157.65 cm^{-1}
2:	0.00 cm^{-1}	8:	109.72 cm^{-1}	14:	164.85 cm^{-1}
3:	0.00 cm^{-1}	9:	110.60 cm^{-1}	15:	174.35 cm^{-1}
4:	0.00 cm^{-1}	10:	127.68 cm^{-1}	16:	179.65 cm^{-1}
5:	0.00 cm^{-1}	11:	131.32 cm^{-1}	17:	200.16 cm^{-1}

18:	310.89 cm^{-1}	34:	694.67 cm^{-1}	50:	1428.23 cm^{-1}
19:	323.54 cm^{-1}	35:	728.02 cm^{-1}	51:	3007.45 cm^{-1}
20:	349.35 cm^{-1}	36:	1179.86 cm^{-1}	52:	3008.32 cm^{-1}
21:	457.61 cm^{-1}	37:	1188.74 cm^{-1}	53:	3019.73 cm^{-1}
22:	482.25 cm^{-1}	38:	1190.69 cm^{-1}	54:	3022.02 cm^{-1}
23:	565.89 cm^{-1}	39:	1247.29 cm^{-1}	55:	3023.37 cm^{-1}
24:	579.62 cm^{-1}	40:	1251.72 cm^{-1}	56:	3060.79 cm^{-1}
25:	581.44 cm^{-1}	41:	1407.46 cm^{-1}	57:	3062.52 cm^{-1}
26:	594.64 cm^{-1}	42:	1411.20 cm^{-1}	58:	3097.34 cm^{-1}
27:	606.45 cm^{-1}	43:	1415.49 cm^{-1}	59:	3100.80 cm^{-1}
28:	614.95 cm^{-1}	44:	1417.52 cm^{-1}	60:	3101.38 cm^{-1}
29:	627.59 cm^{-1}	45:	1420.11 cm^{-1}	61:	3103.83 cm^{-1}
30:	652.98 cm^{-1}	46:	1420.74 cm^{-1}	62:	3107.14 cm^{-1}
31:	665.74 cm^{-1}	47:	1422.40 cm^{-1}	63:	3113.44 cm^{-1}
32:	668.13 cm^{-1}	48:	1422.81 cm^{-1}	64:	3114.09 cm^{-1}
33:	686.84 cm^{-1}	49:	1425.16 cm^{-1}	65:	3115.00 cm^{-1}

This molecular structure was also optimized and the vibrational frequencies calculated using the BP86, the TPSS or the B3LYP density functional. The deviation from the results given above are negligible though. The xyz coordinates and vibrational frequencies can be found in the corresponding Orca output file within the deposited raw data.

$[Al_2Me_5]^+$ – PBE0-D3BJ/def2-TZVP (S_0)

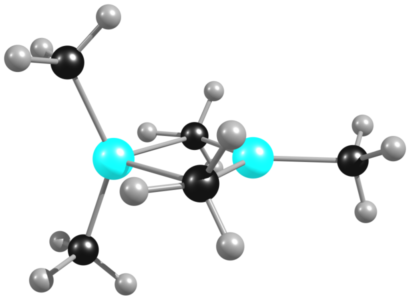

Al	-0.797864000	0.163653000	0.850426000
C	-1.150061000	2.070122000	0.388218000
Al	1.074142000	1.726780000	-0.077181000
C	1.047982000	-0.498776000	0.495140000
C	1.165667000	1.725875000	-2.005065000
C	1.995875000	2.576091000	1.391990000
C	-2.150764000	-0.961692000	1.600063000
H	-3.147163000	-0.673385000	1.257367000
H	-2.143909000	-0.882551000	2.692013000
H	-1.986251000	-2.011277000	1.346160000
H	0.425559000	1.072617000	-2.475425000
H	2.151393000	1.398772000	-2.348956000
H	1.008893000	2.732565000	-2.403702000
H	1.919984000	3.665239000	1.321435000
H	3.061912000	2.330516000	1.376758000
H	1.613071000	2.289357000	2.375523000
H	0.804266000	-1.337664000	-0.171427000
H	1.269487000	-0.922342000	1.484324000
H	2.033583000	-0.172356000	0.135230000
H	-0.659218000	2.976385000	0.007129000
H	-1.576431000	2.399747000	1.345595000
H	-1.987012000	1.927084000	-0.309226000

0:	0.00 cm^{-1}	6:	20.51 cm^{-1}	12:	129.80 cm^{-1}
1:	0.00 cm^{-1}	7:	28.85 cm^{-1}	13:	135.49 cm^{-1}
2:	0.00 cm^{-1}	8:	98.51 cm^{-1}	14:	159.59 cm^{-1}
3:	0.00 cm^{-1}	9:	105.74 cm^{-1}	15:	160.04 cm^{-1}
4:	0.00 cm^{-1}	10:	113.38 cm^{-1}	16:	194.08 cm^{-1}
5:	0.00 cm^{-1}	11:	118.28 cm^{-1}	17:	204.39 cm^{-1}

18:	268.53 cm^{-1}	34:	786.49 cm^{-1}	50:	1449.40 cm^{-1}
19:	277.26 cm^{-1}	35:	787.33 cm^{-1}	51:	3007.62 cm^{-1}
20:	332.00 cm^{-1}	36:	1239.44 cm^{-1}	52:	3008.31 cm^{-1}
21:	483.33 cm^{-1}	37:	1245.50 cm^{-1}	53:	3042.92 cm^{-1}
22:	563.66 cm^{-1}	38:	1246.42 cm^{-1}	54:	3043.74 cm^{-1}
23:	577.77 cm^{-1}	39:	1307.32 cm^{-1}	55:	3049.12 cm^{-1}
24:	579.88 cm^{-1}	40:	1309.90 cm^{-1}	56:	3066.13 cm^{-1}
25:	594.45 cm^{-1}	41:	1389.36 cm^{-1}	57:	3067.72 cm^{-1}
26:	645.28 cm^{-1}	42:	1396.82 cm^{-1}	58:	3085.84 cm^{-1}
27:	646.25 cm^{-1}	43:	1428.21 cm^{-1}	59:	3086.34 cm^{-1}
28:	661.95 cm^{-1}	44:	1429.02 cm^{-1}	60:	3122.80 cm^{-1}
29:	662.22 cm^{-1}	45:	1430.00 cm^{-1}	61:	3123.71 cm^{-1}
30:	699.80 cm^{-1}	46:	1430.90 cm^{-1}	62:	3124.02 cm^{-1}
31:	718.04 cm^{-1}	47:	1437.95 cm^{-1}	63:	3125.60 cm^{-1}
32:	731.66 cm^{-1}	48:	1438.85 cm^{-1}	64:	3127.58 cm^{-1}
33:	735.08 cm^{-1}	49:	1445.13 cm^{-1}	65:	3146.82 cm^{-1}

This molecular structure was also optimized and the vibrational frequencies calculated using the BP86, the TPSS or the B3LYP density functional. The deviation from the results given above are negligible though. The xyz coordinates and vibrational frequencies can be found in the corresponding Orca output file within the deposited raw data.

[Al_2Me_5]$^+$ – PBE0-D3BJ/def2-TZVP/CPCM(THF) (S_0)

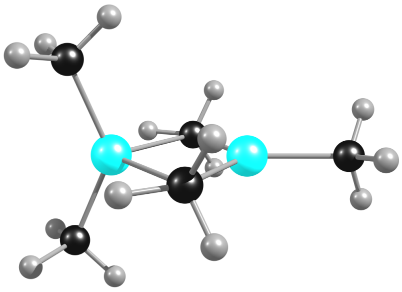

Al	-0.800923000	0.143081000	0.859196000
C	-1.102820000	2.079102000	0.464376000
Al	1.037974000	1.707941000	-0.040293000
C	1.066622000	-0.470285000	0.517718000
C	1.103401000	1.700964000	-1.981025000
C	2.060399000	2.616718000	1.337081000
C	-2.191118000	-0.997605000	1.523283000
H	-3.106074000	-0.865668000	0.939309000
H	-2.415332000	-0.745519000	2.564185000
H	-1.894987000	-2.048392000	1.479131000
H	0.337307000	1.046015000	-2.408640000
H	2.076081000	1.351440000	-2.342398000
H	0.943629000	2.705354000	-2.386593000
H	1.978195000	3.703742000	1.234569000
H	3.123057000	2.360576000	1.274330000
H	1.714465000	2.351522000	2.341250000
H	0.817841000	-1.295956000	-0.162728000
H	1.235568000	-0.892551000	1.518435000
H	2.073190000	-0.164461000	0.199330000
H	-0.633059000	3.009952000	0.117011000
H	-1.500926000	2.351082000	1.451487000
H	-1.949348000	1.947708000	-0.222624000

0:	0.00 cm^{-1}	6:	36.55 cm^{-1}	12:	124.66 cm^{-1}
1:	0.00 cm^{-1}	7:	46.41 cm^{-1}	13:	129.51 cm^{-1}
2:	0.00 cm^{-1}	8:	92.79 cm^{-1}	14:	153.56 cm^{-1}
3:	0.00 cm^{-1}	9:	108.10 cm^{-1}	15:	163.39 cm^{-1}
4:	0.00 cm^{-1}	10:	115.82 cm^{-1}	16:	216.17 cm^{-1}
5:	0.00 cm^{-1}	11:	123.58 cm^{-1}	17:	228.39 cm^{-1}

18:	278.23 cm^{-1}	34:	746.76 cm^{-1}	50:	1426.71 cm^{-1}
19:	298.84 cm^{-1}	35:	762.68 cm^{-1}	51:	3008.95 cm^{-1}
20:	338.29 cm^{-1}	36:	1200.44 cm^{-1}	52:	3013.75 cm^{-1}
21:	479.73 cm^{-1}	37:	1202.40 cm^{-1}	53:	3033.22 cm^{-1}
22:	548.09 cm^{-1}	38:	1202.74 cm^{-1}	54:	3034.67 cm^{-1}
23:	569.32 cm^{-1}	39:	1275.39 cm^{-1}	55:	3048.39 cm^{-1}
24:	584.90 cm^{-1}	40:	1285.09 cm^{-1}	56:	3070.85 cm^{-1}
25:	591.80 cm^{-1}	41:	1375.84 cm^{-1}	57:	3075.57 cm^{-1}
26:	635.96 cm^{-1}	42:	1382.81 cm^{-1}	58:	3093.25 cm^{-1}
27:	636.98 cm^{-1}	43:	1409.81 cm^{-1}	59:	3097.80 cm^{-1}
28:	651.89 cm^{-1}	44:	1412.24 cm^{-1}	60:	3116.08 cm^{-1}
29:	656.05 cm^{-1}	45:	1414.99 cm^{-1}	61:	3116.67 cm^{-1}
30:	676.19 cm^{-1}	46:	1416.32 cm^{-1}	62:	3116.95 cm^{-1}
31:	680.93 cm^{-1}	47:	1419.82 cm^{-1}	63:	3117.11 cm^{-1}
32:	689.80 cm^{-1}	48:	1420.63 cm^{-1}	64:	3133.60 cm^{-1}
33:	717.37 cm^{-1}	49:	1423.54 cm^{-1}	65:	3144.47 cm^{-1}

This molecular structure was also optimized and the vibrational frequencies calculated using the BP86, the TPSS or the B3LYP density functional. The deviation from the results given above are negligible though. The xyz coordinates and vibrational frequencies can be found in the corresponding Orca output file within the deposited raw data.

[CH]$^{\bullet}$ – PBE0-D3BJ/def2-TZVP (D_1)

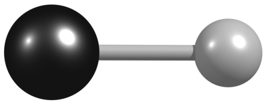

H	-0.930107000	0.753793000	1.053640000
C	0.195287000	0.745747000	1.053640000

0:	0.00 cm^{-1}	2:	0.00 cm^{-1}	4:	0.00 cm^{-1}
1:	0.00 cm^{-1}	3:	0.00 cm^{-1}	5:	2845.25 cm^{-1}

[CH]$^{\bullet}$ – PBE0-D3BJ/def2-TZVP/CPCM(THF) (D_1)

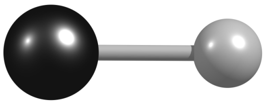

H	-0.926426000	0.753766000	1.053640000
C	0.191606000	0.745774000	1.053640000

0:	0.00 cm^{-1}	2:	0.00 cm^{-1}	4:	0.00 cm^{-1}
1:	0.00 cm^{-1}	3:	0.00 cm^{-1}	5:	2916.13 cm^{-1}

[CH]$^{+}$ – PBE0-D3BJ/def2-TZVP (S_0)

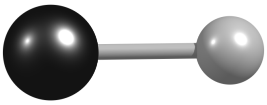

H	-0.937496000	0.753845000	1.053640000
C	0.202676000	0.745695000	1.053640000

0:	0.00 cm^{-1}	2:	0.00 cm^{-1}	4:	0.00 cm^{-1}
1:	0.00 cm^{-1}	3:	0.00 cm^{-1}	5:	2805.67 cm^{-1}

[CH]$^+$ – PBE0-D3BJ/def2-TZVP/CPCM(THF) (S_0)

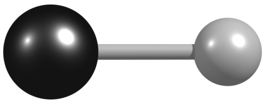

H	-0.932029000	0.753806000	1.053640000
C	0.197209000	0.745734000	1.053640000

0:	0.00 cm^{-1}	2:	0.00 cm^{-1}	4:	0.00 cm^{-1}
1:	0.00 cm^{-1}	3:	0.00 cm^{-1}	5:	2900.59 cm^{-1}

[CH]$^-$ – PBE0-D3BJ/def2-TZVP (T_1)

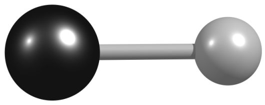

H	-0.930001000	0.753792000	1.053640000
C	0.195181000	0.745748000	1.053640000

0:	0.00 cm^{-1}	2:	0.00 cm^{-1}	4:	0.00 cm^{-1}
1:	0.00 cm^{-1}	3:	0.00 cm^{-1}	5:	2771.53 cm^{-1}

[CH]$^-$ – PBE0-D3BJ/def2-TZVP/CPCM(THF) (T_1)

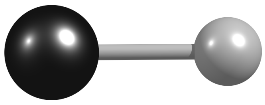

H	-0.930001000	0.753792000	1.053640000
C	0.195181000	0.745748000	1.053640000

0:	0.00 cm^{-1}	2:	0.00 cm^{-1}	4:	0.00 cm^{-1}
1:	0.00 cm^{-1}	3:	0.00 cm^{-1}	5:	2771.53 cm^{-1}

HCCH – PBE0-D3BJ/def2-TZVP (S_0)

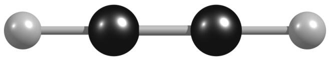

C	0.306346000	1.719097000	0.072146000
C	-0.092636000	1.975703000	-1.026828000
H	-0.447532000	2.203969000	-2.004271000
H	0.661243000	1.490831000	1.049593000

0:	0.00 cm^{-1}	4:	0.00 cm^{-1}	8:	771.04 cm^{-1}
1:	0.00 cm^{-1}	5:	0.00 cm^{-1}	9:	2083.43 cm^{-1}
2:	0.00 cm^{-1}	6:	632.87 cm^{-1}	10:	3428.83 cm^{-1}
3:	0.00 cm^{-1}	7:	767.49 cm^{-1}	11:	3527.04 cm^{-1}

HCCH – PBE0-D3BJ/def2-TZVP/CPCM(THF) (S_0)

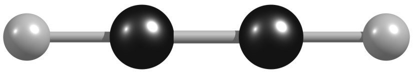

C	0.306668000	1.718888000	0.073050000
C	-0.092958000	1.975912000	-1.027730000
H	-0.449157000	2.205014000	-2.008740000
H	0.662867000	1.489787000	1.054061000

0:	0.00 cm^{-1}	4:	0.00 cm^{-1}	8:	780.82 cm^{-1}
1:	0.00 cm^{-1}	5:	0.00 cm^{-1}	9:	2069.43 cm^{-1}
2:	0.00 cm^{-1}	6:	685.10 cm^{-1}	10:	3389.30 cm^{-1}
3:	0.00 cm^{-1}	7:	778.35 cm^{-1}	11:	3493.79 cm^{-1}

$[HCC]^{\bullet}$ – PBE0-D3BJ/def2-TZVP (D_1)

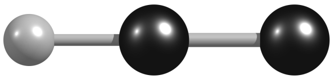

C	0.306657000	1.718898000	0.073005000
C	-0.093391000	1.976187000	-1.028908000
H	-0.448725000	2.204737000	-2.007555000

0:	0.00 cm^{-1}	3:	0.00 cm^{-1}	6:	481.51 cm^{-1}
1:	0.00 cm^{-1}	4:	0.00 cm^{-1}	7:	2106.57 cm^{-1}
2:	0.00 cm^{-1}	5:	480.72 cm^{-1}	8:	3465.39 cm^{-1}

[HCC]$^{\bullet}$ – PBE0-D3BJ/def2-TZVP/CPCM(THF) (D_1)

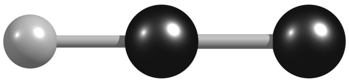

C	0.307407000	1.718413000	0.075095000
C	-0.093008000	1.975946000	-1.027903000
H	-0.449858000	2.205463000	-2.010650000

0:	0.00 cm^{-1}	3:	0.00 cm^{-1}	6:	502.90 cm^{-1}
1:	0.00 cm^{-1}	4:	0.00 cm^{-1}	7:	2098.32 cm^{-1}
2:	0.00 cm^{-1}	5:	0.00 cm^{-1}	8:	3423.79 cm^{-1}

[HCCH]$^{+}$ – PBE0-D3BJ/def2-TZVP (D_1)

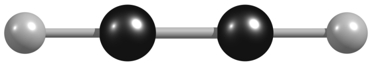

C	0.314050000	1.714142000	0.093362000
C	-0.100339000	1.980657000	-1.048041000
H	-0.460798000	2.212502000	-2.040811000
H	0.674508000	1.482299000	1.086131000

0:	0.00 cm^{-1}	4:	0.00 cm^{-1}	8:	794.66 cm^{-1}
1:	0.00 cm^{-1}	5:	641.03 cm^{-1}	9:	1907.48 cm^{-1}
2:	0.00 cm^{-1}	6:	720.52 cm^{-1}	10:	3266.41 cm^{-1}
3:	0.00 cm^{-1}	7:	763.52 cm^{-1}	11:	3366.63 cm^{-1}

[HCCH]$^{+}$ – PBE0-D3BJ/def2-TZVP/CPCM(THF) (D_1)

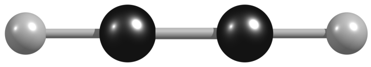

C	0.313700000	1.714456000	0.092344000
C	-0.099990000	1.980343000	-1.047024000
H	-0.460019000	2.212030000	-2.038683000
H	0.673729000	1.482770000	1.084003000

0:	0.00 cm^{-1}	4:	0.00 cm^{-1}	8:	770.46 cm^{-1}
1:	0.00 cm^{-1}	5:	0.00 cm^{-1}	9:	1915.89 cm^{-1}
2:	0.00 cm^{-1}	6:	713.18 cm^{-1}	10:	3276.01 cm^{-1}
3:	0.00 cm^{-1}	7:	745.22 cm^{-1}	11:	3379.83 cm^{-1}

[HCC]$^+$ – PBE0-D3BJ/def2-TZVP (S_0)

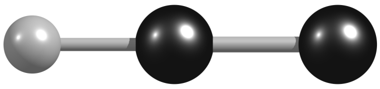

C	0.319760000	1.710471000	0.109091000
C	-0.096508000	1.978191000	-1.037488000
H	-0.458710000	2.211160000	-2.035062000

0:	0.00 cm^{-1}	3:	0.00 cm^{-1}	6:	550.74 cm^{-1}
1:	0.00 cm^{-1}	4:	0.00 cm^{-1}	7:	1909.39 cm^{-1}
2:	0.00 cm^{-1}	5:	398.16 cm^{-1}	8:	3262.65 cm^{-1}

[HCC]$^+$ – PBE0-D3BJ/def2-TZVP/CPCM(THF) (S_0)

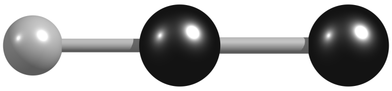

C	0.318957000	1.710992000	0.107050000
C	-0.096157000	1.977955000	-1.036857000
H	-0.458259000	2.210875000	-2.033651000

0:	0.00 cm^{-1}	3:	0.00 cm^{-1}	6:	359.28 cm^{-1}
1:	0.00 cm^{-1}	4:	0.00 cm^{-1}	7:	1924.25 cm^{-1}
2:	0.00 cm^{-1}	5:	0.00 cm^{-1}	8:	3271.73 cm^{-1}

[HCC]$^+$ – PBE0-D3BJ/def2-TZVP (T_1)

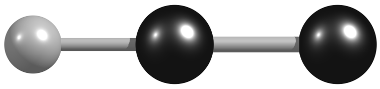

C	0.319633000	1.710552000	0.108740000
C	-0.096628000	1.978269000	-1.037814000
H	-0.458464000	2.211001000	-2.034385000

0:	0.00 cm^{-1}	3:	0.00 cm^{-1}	6:	798.94 cm^{-1}
1:	0.00 cm^{-1}	4:	0.00 cm^{-1}	7:	1913.02 cm^{-1}
2:	0.00 cm^{-1}	5:	560.87 cm^{-1}	8:	3275.21 cm^{-1}

$[HCC]^+$ – PBE0-D3BJ/def2-TZVP/CPCM(THF) (T_1)

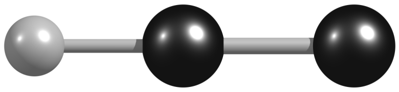

C	0.318861000	1.711089000	0.106712000
C	-0.096339000	1.978002000	-1.037214000
H	-0.457981000	2.210731000	-2.032957000

0:	0.00 cm^{-1}	3:	0.00 cm^{-1}	6:	730.44 cm^{-1}
1:	0.00 cm^{-1}	4:	0.00 cm^{-1}	7:	1928.19 cm^{-1}
2:	0.00 cm^{-1}	5:	0.00 cm^{-1}	8:	3287.52 cm^{-1}

$[HCCH]^–$ – PBE0-D3BJ/def2-TZVP (D_1)

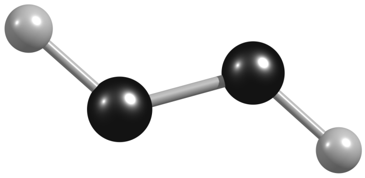

C	0.382165000	1.820825000	0.131230000
C	-0.186302000	1.789972000	-1.040446000
H	-0.131921000	2.628715000	-1.759791000
H	0.327783000	0.982081000	0.850575000

0:	0.00 cm^{-1}	4:	0.00 cm^{-1}	8:	1055.18 cm^{-1}
1:	0.00 cm^{-1}	5:	0.00 cm^{-1}	9:	1638.82 cm^{-1}
2:	0.00 cm^{-1}	6:	771.31 cm^{-1}	10:	2849.23 cm^{-1}
3:	0.00 cm^{-1}	7:	875.33 cm^{-1}	11:	2915.98 cm^{-1}

[HCCH]⁻ – PBE0-D3BJ/def2-TZVP/CPCM(THF) (D_1)

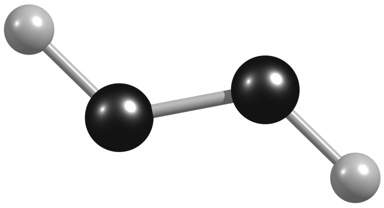

C	0.002125000	1.830213000	0.163773000
C	0.211587000	1.864586000	-1.118455000
H	-0.511755000	2.224067000	-1.865685000
H	0.725463000	1.470734000	0.911008000

0:	0.00 cm^{-1}	4:	0.00 cm^{-1}	8:	1057.17 cm^{-1}
1:	0.00 cm^{-1}	5:	0.00 cm^{-1}	9:	1654.73 cm^{-1}
2:	0.00 cm^{-1}	6:	759.20 cm^{-1}	10:	2958.11 cm^{-1}
3:	0.00 cm^{-1}	7:	894.42 cm^{-1}	11:	3001.78 cm^{-1}

[HCC]⁻ – PBE0-D3BJ/def2-TZVP (S_0)

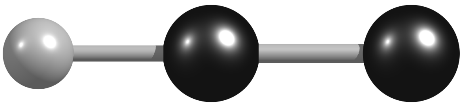

C	0.315880000	1.712966000	0.098407000
C	-0.097219000	1.978649000	-1.039449000
H	-0.454120000	2.208207000	-2.022417000

0:	0.00 cm^{-1}	3:	0.00 cm^{-1}	6:	543.13 cm^{-1}
1:	0.00 cm^{-1}	4:	0.00 cm^{-1}	7:	1909.30 cm^{-1}
2:	0.00 cm^{-1}	5:	542.45 cm^{-1}	8:	3380.28 cm^{-1}

[HCC]$^{-}$ – PBE0-D3BJ/def2-TZVP/CPCM(THF) (S_0)

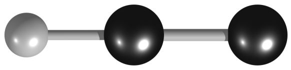

C	0.314588000	1.713775000	0.095121000
C	-0.096223000	1.978052000	-1.037250000
H	-0.453824000	2.207995000	-2.021329000

0: 0.00 cm^{-1}	3: 0.00 cm^{-1}	6: 650.90 cm^{-1}
1: 0.00 cm^{-1}	4: 0.00 cm^{-1}	7: 1939.82 cm^{-1}
2: 0.00 cm^{-1}	5: 0.00 cm^{-1}	8: 3386.90 cm^{-1}

[HCC]$^{-}$ – PBE0-D3BJ/def2-TZVP (T_1)

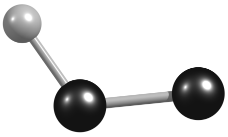

C	0.312244000	1.864890000	0.099004000
C	-0.135631000	1.703015000	-1.166553000
H	-0.382884000	2.620473000	-1.793682000

0: 0.00 cm^{-1}	3: 0.00 cm^{-1}	6: 880.95 cm^{-1}
1: 0.00 cm^{-1}	4: 0.00 cm^{-1}	7: 1415.79 cm^{-1}
2: 0.00 cm^{-1}	5: 0.00 cm^{-1}	8: 2452.52 cm^{-1}

[HCC]$^{-}$ – PBE0-D3BJ/def2-TZVP/CPCM(THF) (T_1)

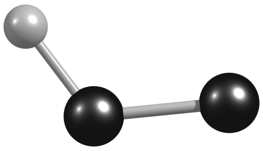

C	0.313844000	1.855793000	0.102836000
C	-0.136008000	1.720927000	-1.166336000
H	-0.384106000	2.611659000	-1.797732000

0: 0.00 cm^{-1}	3: 0.00 cm^{-1}	6: 897.30 cm^{-1}
1: 0.00 cm^{-1}	4: 0.00 cm^{-1}	7: 1463.68 cm^{-1}
2: 0.00 cm^{-1}	5: 0.00 cm^{-1}	8: 2703.42 cm^{-1}

C_2H_4 – PBE0-D3BJ/def2-TZVP (S_0)

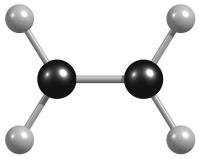

C	-0.287317000	3.212634000	0.345342000
C	-0.287317000	3.212634000	1.668287000
H	-0.287317000	4.135748000	-0.224053000
H	-0.287317000	2.289520000	-0.224053000
H	-0.287317000	4.135749000	2.237681000
H	-0.287317000	2.289520000	2.237681000

0:	0.00 cm^{-1}	6:	828.94 cm^{-1}	12:	1467.05 cm^{-1}
1:	0.00 cm^{-1}	7:	978.53 cm^{-1}	13:	1702.81 cm^{-1}
2:	0.00 cm^{-1}	8:	984.61 cm^{-1}	14:	3148.14 cm^{-1}
3:	0.00 cm^{-1}	9:	1067.60 cm^{-1}	15:	3161.93 cm^{-1}
4:	0.00 cm^{-1}	10:	1238.88 cm^{-1}	16:	3222.68 cm^{-1}
5:	0.00 cm^{-1}	11:	1381.67 cm^{-1}	17:	3248.61 cm^{-1}

C_2H_4 – PBE0-D3BJ/def2-TZVP/CPCM(THF) (S_0)

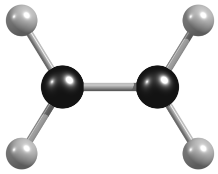

C	-0.287317000	3.212634000	0.344633000
C	-0.287317000	3.212634000	1.668995000
H	-0.287317000	4.137293000	-0.224668000
H	-0.287317000	2.287976000	-0.224668000
H	-0.287317000	4.137293000	2.238296000
H	-0.287317000	2.287976000	2.238296000

0:	0.00 cm^{-1}	6:	823.87 cm^{-1}	12:	1455.35 cm^{-1}
1:	0.00 cm^{-1}	7:	980.96 cm^{-1}	13:	1692.41 cm^{-1}
2:	0.00 cm^{-1}	8:	995.63 cm^{-1}	14:	3139.64 cm^{-1}
3:	0.00 cm^{-1}	9:	1069.90 cm^{-1}	15:	3153.07 cm^{-1}
4:	0.00 cm^{-1}	10:	1230.16 cm^{-1}	16:	3216.83 cm^{-1}
5:	0.00 cm^{-1}	11:	1363.60 cm^{-1}	17:	3242.05 cm^{-1}

[C_2H_3]$^•$ – PBE0-D3BJ/def2-TZVP (D_1)

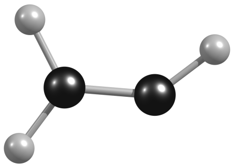

C	-0.287318000	3.224384000	0.317606000
C	-0.287317000	3.304792000	1.616547000
H	-0.287317000	4.116566000	-0.312845000
H	-0.287316000	2.269376000	-0.202391000
H	-0.287317000	4.071394000	2.377124000

0:	0.00 cm^{-1}	5:	0.00 cm^{-1}	10:	1383.48 cm^{-1}
1:	0.00 cm^{-1}	6:	708.90 cm^{-1}	11:	1669.20 cm^{-1}
2:	0.00 cm^{-1}	7:	836.78 cm^{-1}	12:	3082.85 cm^{-1}
3:	0.00 cm^{-1}	8:	927.85 cm^{-1}	13:	3171.83 cm^{-1}
4:	0.00 cm^{-1}	9:	1044.23 cm^{-1}	14:	3266.35 cm^{-1}

[C_2H_3]$^•$ – PBE0-D3BJ/def2-TZVP/CPCM(THF) (D_1)

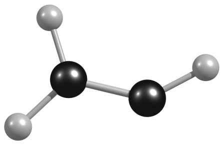

C	-0.287316000	3.222695000	0.317072000
C	-0.287317000	3.307822000	1.616014000
H	-0.287317000	4.116660000	-0.313493000
H	-0.287318000	2.267279000	-0.205245000
H	-0.287317000	4.072057000	2.381693000

0:	0.00 cm^{-1}	5:	0.00 cm^{-1}	10:	1368.52 cm^{-1}
1:	0.00 cm^{-1}	6:	702.87 cm^{-1}	11:	1663.80 cm^{-1}
2:	0.00 cm^{-1}	7:	834.15 cm^{-1}	12:	3067.35 cm^{-1}
3:	0.00 cm^{-1}	8:	934.36 cm^{-1}	13:	3162.11 cm^{-1}
4:	0.00 cm^{-1}	9:	1036.32 cm^{-1}	14:	3249.46 cm^{-1}

$[C_2H_4]^+$ – PBE0-D3BJ/def2-TZVP (D_1)

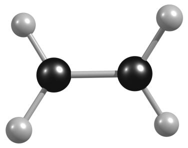

C	-0.110940000	3.087920000	0.244185000
C	-0.135224000	3.006815000	1.627354000
H	0.126254000	4.025282000	-0.260956000
H	-0.328383000	2.216501000	-0.375029000
H	-0.375543000	3.877920000	2.238500000
H	0.085315000	2.069768000	2.140563000

0:	0.00 cm^{-1}	6:	610.04 cm^{-1}	12:	1421.96 cm^{-1}
1:	0.00 cm^{-1}	7:	781.08 cm^{-1}	13:	1505.92 cm^{-1}
2:	0.00 cm^{-1}	8:	882.95 cm^{-1}	14:	3101.71 cm^{-1}
3:	0.00 cm^{-1}	9:	1075.36 cm^{-1}	15:	3104.44 cm^{-1}
4:	0.00 cm^{-1}	10:	1200.82 cm^{-1}	16:	3199.86 cm^{-1}
5:	0.00 cm^{-1}	11:	1286.29 cm^{-1}	17:	3219.66 cm^{-1}

$[C_2H_4]^+$ – PBE0-D3BJ/def2-TZVP/CPCM(THF) (D_1)

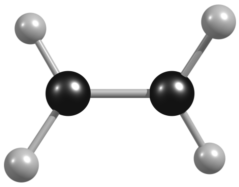

C	-0.110037000	3.087516000	0.247693000
C	-0.135701000	3.007227000	1.623837000
H	0.132404000	4.024424000	-0.251950000
H	-0.332370000	2.215388000	-0.365557000
H	-0.380920000	3.879302000	2.228419000
H	0.088102000	2.070349000	2.132175000

0:	0.00 cm^{-1}	6:	622.67 cm^{-1}	12:	1401.24 cm^{-1}
1:	0.00 cm^{-1}	7:	770.73 cm^{-1}	13:	1498.75 cm^{-1}
2:	0.00 cm^{-1}	8:	870.15 cm^{-1}	14:	3119.69 cm^{-1}
3:	0.00 cm^{-1}	9:	1070.93 cm^{-1}	15:	3125.32 cm^{-1}
4:	0.00 cm^{-1}	10:	1196.51 cm^{-1}	16:	3222.99 cm^{-1}
5:	0.00 cm^{-1}	11:	1284.88 cm^{-1}	17:	3243.52 cm^{-1}

$[C_2H_3]^+$ – PBE0-D3BJ/def2-TZVP (S_0)

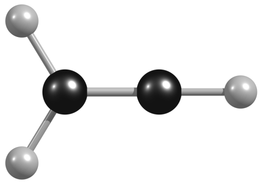

C	-0.287317000	3.286053000	0.284066000
C	-0.287317000	3.571273000	1.501754000
H	-0.287317000	4.072577000	-0.488061000
H	-0.287317000	2.238387000	-0.058263000
H	-0.287317000	3.818223000	2.556546000

0:	0.00 cm^{-1}	5:	0.00 cm^{-1}	10:	1163.57 cm^{-1}
1:	0.00 cm^{-1}	6:	226.80 cm^{-1}	11:	1797.08 cm^{-1}
2:	0.00 cm^{-1}	7:	673.76 cm^{-1}	12:	3015.91 cm^{-1}
3:	0.00 cm^{-1}	8:	842.54 cm^{-1}	13:	3069.52 cm^{-1}
4:	0.00 cm^{-1}	9:	1076.25 cm^{-1}	14:	3298.21 cm^{-1}

$[C_2H_3]^+$ – PBE0-D3BJ/def2-TZVP/CPCM(THF) (S_0)

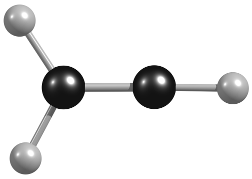

C	-0.287317000	3.266677000	0.286397000
C	-0.287317000	3.565112000	1.498165000
H	-0.287317000	4.083426000	-0.455246000
H	-0.287317000	2.230826000	-0.077909000
H	-0.287317000	3.840471000	2.544635000

0:	0.00 cm^{-1}	5:	0.00 cm^{-1}	10:	1135.71 cm^{-1}
1:	0.00 cm^{-1}	6:	189.44 cm^{-1}	11:	1810.62 cm^{-1}
2:	0.00 cm^{-1}	7:	694.68 cm^{-1}	12:	3022.50 cm^{-1}
3:	0.00 cm^{-1}	8:	839.17 cm^{-1}	13:	3101.59 cm^{-1}
4:	0.00 cm^{-1}	9:	1061.36 cm^{-1}	14:	3319.83 cm^{-1}

$[C_2H_3]^+$ – PBE0-D3BJ/def2-TZVP (T_1)

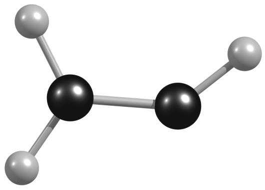

C	-0.287326000	3.223292000	0.276337000
C	-0.287318000	3.304332000	1.660644000
H	-0.287314000	4.125835000	-0.343586000
H	-0.287312000	2.250192000	-0.218553000
H	-0.287314000	4.082861000	2.421200000

0:	0.00 cm^{-1}	5:	0.00 cm^{-1}	10:	1341.24 cm^{-1}
1:	0.00 cm^{-1}	6:	728.27 cm^{-1}	11:	1473.33 cm^{-1}
2:	0.00 cm^{-1}	7:	766.79 cm^{-1}	12:	3072.68 cm^{-1}
3:	0.00 cm^{-1}	8:	1047.71 cm^{-1}	13:	3188.59 cm^{-1}
4:	0.00 cm^{-1}	9:	1056.01 cm^{-1}	14:	3206.07 cm^{-1}

$[C_2H_3]^+$ – PBE0-D3BJ/def2-TZVP/CPCM(THF) (T_1)

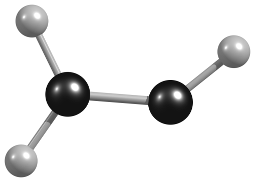

C	-0.287353000	3.220193000	0.279192000
C	-0.287321000	3.299489000	1.657094000
H	-0.287306000	4.128527000	-0.328360000
H	-0.287298000	2.251118000	-0.218246000
H	-0.287307000	4.087185000	2.406362000

0:	0.00 cm^{-1}	5:	0.00 cm^{-1}	10:	1342.49 cm^{-1}
1:	0.00 cm^{-1}	6:	725.62 cm^{-1}	11:	1475.23 cm^{-1}
2:	0.00 cm^{-1}	7:	764.86 cm^{-1}	12:	3101.19 cm^{-1}
3:	0.00 cm^{-1}	8:	1048.66 cm^{-1}	13:	3216.26 cm^{-1}
4:	0.00 cm^{-1}	9:	1057.42 cm^{-1}	14:	3224.91 cm^{-1}

$[C_2H_4]^–$ – PBE0-D3BJ/def2-TZVP (D_1)

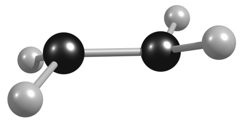

C	-0.193680000	3.210691000	0.263733000
C	0.702350000	3.225760000	1.388193000
H	-0.227709000	4.102288000	-0.371856000
H	-0.263990000	2.285470000	-0.318591000
H	0.772634000	4.150987000	1.970518000
H	0.736368000	2.334151000	2.023776000

0:	0.00 cm^{-1}	6:	462.43 cm^{-1}	12:	1408.12 cm^{-1}
1:	0.00 cm^{-1}	7:	578.88 cm^{-1}	13:	1465.22 cm^{-1}
2:	0.00 cm^{-1}	8:	654.07 cm^{-1}	14:	2998.28 cm^{-1}
3:	0.00 cm^{-1}	9:	842.45 cm^{-1}	15:	3014.41 cm^{-1}
4:	0.00 cm^{-1}	10:	1182.62 cm^{-1}	16:	3058.85 cm^{-1}
5:	0.00 cm^{-1}	11:	1197.35 cm^{-1}	17:	3080.44 cm^{-1}

$[C_2H_4]^–$ – PBE0-D3BJ/def2-TZVP/CPCM(THF) (D_1)

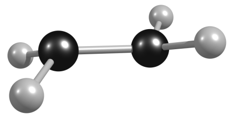

C	-0.182524000	3.210276000	0.255736000
C	0.691188000	3.226157000	1.396183000
H	-0.231800000	4.103847000	-0.371443000
H	-0.267965000	2.284126000	-0.318118000
H	0.776643000	4.152334000	1.970005000
H	0.740430000	2.332608000	2.023411000

0:	0.00 cm^{-1}	6:	479.66 cm^{-1}	12:	1422.82 cm^{-1}
1:	0.00 cm^{-1}	7:	561.81 cm^{-1}	13:	1474.75 cm^{-1}
2:	0.00 cm^{-1}	8:	649.05 cm^{-1}	14:	3044.33 cm^{-1}
3:	0.00 cm^{-1}	9:	863.19 cm^{-1}	15:	3058.95 cm^{-1}
4:	0.00 cm^{-1}	10:	1189.98 cm^{-1}	16:	3109.60 cm^{-1}
5:	0.00 cm^{-1}	11:	1221.55 cm^{-1}	17:	3128.23 cm^{-1}

$[C_2H_3]^-$ – PBE0-D3BJ/def2-TZVP (S_0)

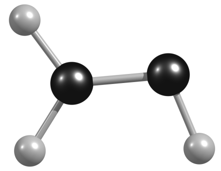

C	-0.287317000	3.218501000	0.387168000
C	-0.287317000	3.128594000	1.734453000
H	-0.287317000	4.148485000	-0.219677000
H	-0.287317000	2.318900000	-0.249064000
H	-0.287317000	4.172034000	2.143162000

0:	0.00 cm^{-1}	5:	0.00 cm^{-1}	10:	1393.48 cm^{-1}
1:	0.00 cm^{-1}	6:	860.48 cm^{-1}	11:	1544.69 cm^{-1}
2:	0.00 cm^{-1}	7:	933.60 cm^{-1}	12:	2749.34 cm^{-1}
3:	0.00 cm^{-1}	8:	1097.85 cm^{-1}	13:	2853.56 cm^{-1}
4:	0.00 cm^{-1}	9:	1224.00 cm^{-1}	14:	2952.20 cm^{-1}

$[C_2H_3]^-$ – PBE0-D3BJ/def2-TZVP/CPCM(THF) (S_0)

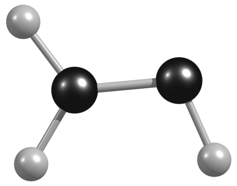

C	-0.287317000	3.215380000	0.381879000
C	-0.287317000	3.138931000	1.727133000
H	-0.287317000	4.145134000	-0.209091000
H	-0.287317000	2.317562000	-0.248915000
H	-0.287317000	4.169506000	2.145035000

0:	0.00 cm^{-1}	5:	0.00 cm^{-1}	10:	1404.15 cm^{-1}
1:	0.00 cm^{-1}	6:	910.52 cm^{-1}	11:	1571.72 cm^{-1}
2:	0.00 cm^{-1}	7:	969.02 cm^{-1}	12:	2855.48 cm^{-1}
3:	0.00 cm^{-1}	8:	1109.21 cm^{-1}	13:	2965.03 cm^{-1}
4:	0.00 cm^{-1}	9:	1234.55 cm^{-1}	14:	3026.38 cm^{-1}

$[C_2H_3]^-$ – PBE0-D3BJ/def2-TZVP (T_1)

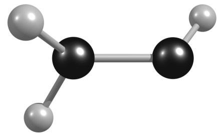

C	-0.555646000	3.304309000	0.311756000
C	0.023752000	3.527820000	1.588617000
H	-0.350281000	4.021736000	-0.497269000
H	-0.598397000	2.276313000	-0.079136000
H	-0.569919000	3.825284000	2.478138000

0:	0.00 cm^{-1}	5:	0.00 cm^{-1}	10:	1209.23 cm^{-1}
1:	0.00 cm^{-1}	6:	522.10 cm^{-1}	11:	1412.53 cm^{-1}
2:	0.00 cm^{-1}	7:	618.18 cm^{-1}	12:	2814.15 cm^{-1}
3:	0.00 cm^{-1}	8:	914.14 cm^{-1}	13:	2958.32 cm^{-1}
4:	0.00 cm^{-1}	9:	957.98 cm^{-1}	14:	2974.40 cm^{-1}

$[C_2H_3]^-$ – PBE0-D3BJ/def2-TZVP/CPCM(THF) (T_1)

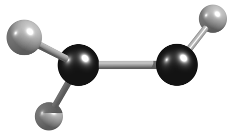

C	-0.535424000	3.300678000	0.309409000
C	0.023676000	3.531442000	1.591204000
H	-0.363232000	4.024928000	-0.496851000
H	-0.603523000	2.275383000	-0.076032000
H	-0.571989000	3.823030000	2.474376000

0:	0.00 cm^{-1}	5:	0.00 cm^{-1}	10:	1219.29 cm^{-1}
1:	0.00 cm^{-1}	6:	485.64 cm^{-1}	11:	1404.59 cm^{-1}
2:	0.00 cm^{-1}	7:	611.29 cm^{-1}	12:	2906.20 cm^{-1}
3:	0.00 cm^{-1}	8:	900.51 cm^{-1}	13:	3004.27 cm^{-1}
4:	0.00 cm^{-1}	9:	948.68 cm^{-1}	14:	3039.72 cm^{-1}

C_6H_6 – PBE0-D3BJ/def2-TZVP (S_0)

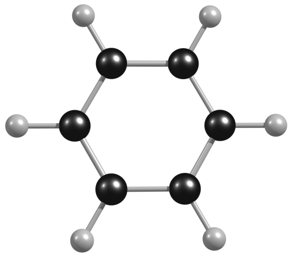

C	0.316430000	0.731143000	0.598579000
C	-0.832265000	0.378722000	-0.095129000
C	-1.980961000	0.026302000	0.598579000
C	-1.980961000	0.026302000	1.986004000
C	-0.832265000	0.378722000	2.679711000
C	0.316430000	0.731142000	1.986004000
H	1.213898000	1.006505000	2.527998000
H	1.213898000	1.006505000	0.056585000
H	-0.832266000	0.378723000	-1.179118000
H	-2.878428000	-0.249060000	0.056585000
H	-2.878429000	-0.249060000	2.527998000
H	-0.832266000	0.378723000	3.763701000

0:	0.00 cm^{-1}	12:	866.10 cm^{-1}	24:	1370.54 cm^{-1}
1:	0.00 cm^{-1}	13:	866.39 cm^{-1}	25:	1373.30 cm^{-1}
2:	0.00 cm^{-1}	14:	969.03 cm^{-1}	26:	1517.95 cm^{-1}
3:	0.00 cm^{-1}	15:	969.06 cm^{-1}	27:	1528.81 cm^{-1}
4:	0.00 cm^{-1}	16:	997.39 cm^{-1}	28:	1664.10 cm^{-1}
5:	0.00 cm^{-1}	17:	1020.89 cm^{-1}	29:	1664.50 cm^{-1}
6:	409.19 cm^{-1}	18:	1027.96 cm^{-1}	30:	3178.04 cm^{-1}
7:	409.23 cm^{-1}	19:	1063.65 cm^{-1}	31:	3188.65 cm^{-1}
8:	606.61 cm^{-1}	20:	1067.88 cm^{-1}	32:	3189.10 cm^{-1}
9:	618.38 cm^{-1}	21:	1163.95 cm^{-1}	33:	3208.06 cm^{-1}
10:	687.92 cm^{-1}	22:	1187.88 cm^{-1}	34:	3209.79 cm^{-1}
11:	720.78 cm^{-1}	23:	1196.11 cm^{-1}	35:	3213.93 cm^{-1}

C_6H_6 – PBE0-D3BJ/def2-TZVP/CPCM(THF) (S_0)

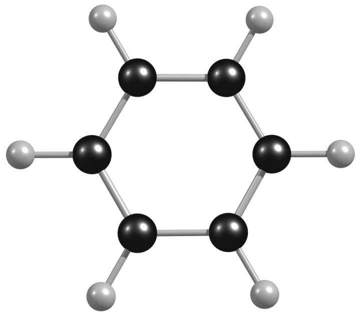

C	0.317550000	0.731375000	0.597914000
C	-0.832265000	0.378722000	-0.096458000
C	-1.982081000	0.026070000	0.597914000
C	-1.982081000	0.026070000	1.986669000
C	-0.832266000	0.378722000	2.681041000
C	0.317550000	0.731375000	1.986669000
H	1.215366000	1.007018000	2.528936000
H	1.215366000	1.007018000	0.055647000
H	-0.832266000	0.378722000	-1.180918000
H	-2.879897000	-0.249573000	0.055647000
H	-2.879897000	-0.249573000	2.528936000
H	-0.832265000	0.378723000	3.765501000

0:	0.00 cm^{-1}	12:	873.69 cm^{-1}	24:	1363.47 cm^{-1}
1:	0.00 cm^{-1}	13:	874.84 cm^{-1}	25:	1373.58 cm^{-1}
2:	0.00 cm^{-1}	14:	993.97 cm^{-1}	26:	1510.82 cm^{-1}
3:	0.00 cm^{-1}	15:	994.89 cm^{-1}	27:	1513.48 cm^{-1}
4:	0.00 cm^{-1}	16:	1021.57 cm^{-1}	28:	1657.33 cm^{-1}
5:	0.00 cm^{-1}	17:	1025.95 cm^{-1}	29:	1657.71 cm^{-1}
6:	410.56 cm^{-1}	18:	1027.70 cm^{-1}	30:	3180.71 cm^{-1}
7:	410.68 cm^{-1}	19:	1064.19 cm^{-1}	31:	3188.64 cm^{-1}
8:	617.89 cm^{-1}	20:	1065.55 cm^{-1}	32:	3188.77 cm^{-1}
9:	618.63 cm^{-1}	21:	1156.52 cm^{-1}	33:	3206.71 cm^{-1}
10:	688.99 cm^{-1}	22:	1183.84 cm^{-1}	34:	3207.45 cm^{-1}
11:	730.72 cm^{-1}	23:	1184.19 cm^{-1}	35:	3213.86 cm^{-1}

$[C_6H_5]^{\bullet}$ – PBE0-D3BJ/def2-TZVP (D_1)

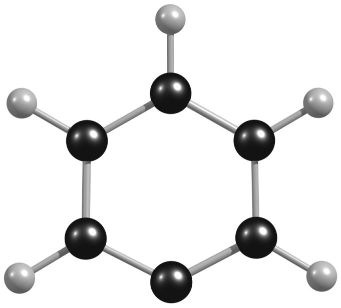

C	0.330835000	0.735569000	0.586804000
C	-0.830156000	0.379372000	-0.099478000
C	-1.975555000	0.027957000	0.601844000
C	-1.983508000	0.025522000	1.990091000
C	-0.834810000	0.377937000	2.698651000
C	0.262072000	0.714449000	1.953171000
H	1.233677000	1.012584000	0.054891000
H	-0.833344000	0.378391000	-1.184140000
H	-2.872713000	-0.247316000	0.060035000
H	-2.883131000	-0.250490000	2.529530000
H	-0.823781000	0.381331000	3.782468000

0:	0.00 cm^{-1}	11:	724.15 cm^{-1}	22:	1307.00 cm^{-1}
1:	0.00 cm^{-1}	12:	823.33 cm^{-1}	23:	1354.48 cm^{-1}
2:	0.00 cm^{-1}	13:	894.71 cm^{-1}	24:	1473.26 cm^{-1}
3:	0.00 cm^{-1}	14:	961.20 cm^{-1}	25:	1479.27 cm^{-1}
4:	0.00 cm^{-1}	15:	982.34 cm^{-1}	26:	1602.34 cm^{-1}
5:	0.00 cm^{-1}	16:	988.91 cm^{-1}	27:	1659.98 cm^{-1}
6:	399.01 cm^{-1}	17:	1033.39 cm^{-1}	28:	3180.02 cm^{-1}
7:	427.18 cm^{-1}	18:	1060.31 cm^{-1}	29:	3186.05 cm^{-1}
8:	596.31 cm^{-1}	19:	1080.42 cm^{-1}	30:	3201.62 cm^{-1}
9:	610.93 cm^{-1}	20:	1172.11 cm^{-1}	31:	3202.13 cm^{-1}
10:	680.85 cm^{-1}	21:	1174.39 cm^{-1}	32:	3216.22 cm^{-1}

$[C_6H_5]^{\bullet}$ – PBE0-D3BJ/def2-TZVP/CPCM(THF) (D_1)

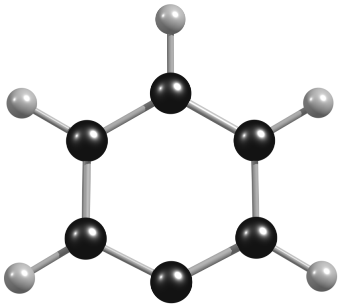

C	0.332787000	0.736109000	0.585805000
C	-0.829666000	0.379525000	-0.100607000
C	-1.975535000	0.028058000	0.601873000
C	-1.984237000	0.025409000	1.991108000
C	-0.834673000	0.377978000	2.700905000
C	0.260979000	0.714055000	1.952510000
H	1.234971000	1.013063000	0.051463000
H	-0.832273000	0.378679000	-1.185556000
H	-2.873038000	-0.247495000	0.059778000
H	-2.883790000	-0.250715000	2.531250000
H	-0.825939000	0.380639000	3.785339000

0:	0.00 cm^{-1}	11:	727.19 cm^{-1}	22:	1300.81 cm^{-1}
1:	0.00 cm^{-1}	12:	827.56 cm^{-1}	23:	1348.90 cm^{-1}
2:	0.00 cm^{-1}	13:	907.21 cm^{-1}	24:	1466.84 cm^{-1}
3:	0.00 cm^{-1}	14:	980.41 cm^{-1}	25:	1474.16 cm^{-1}
4:	0.00 cm^{-1}	15:	985.75 cm^{-1}	26:	1594.79 cm^{-1}
5:	0.00 cm^{-1}	16:	1007.50 cm^{-1}	27:	1655.87 cm^{-1}
6:	399.24 cm^{-1}	17:	1028.47 cm^{-1}	28:	3181.97 cm^{-1}
7:	427.72 cm^{-1}	18:	1054.14 cm^{-1}	29:	3185.70 cm^{-1}
8:	593.37 cm^{-1}	19:	1075.92 cm^{-1}	30:	3196.97 cm^{-1}
9:	615.06 cm^{-1}	20:	1160.24 cm^{-1}	31:	3204.45 cm^{-1}
10:	682.27 cm^{-1}	21:	1162.60 cm^{-1}	32:	3211.88 cm^{-1}

$[C_6H_6]^+$ – PBE0-D3BJ/def2-TZVP (D_1)

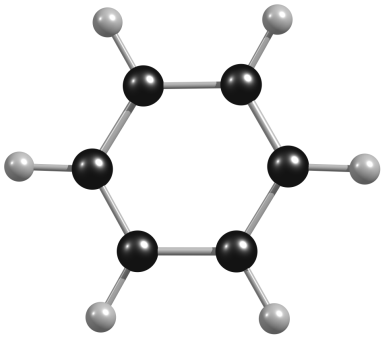

C	0.355119000	0.743015000	0.611530000
C	-0.832265000	0.378722000	-0.081065000
C	-2.019649000	0.014430000	0.611530000
C	-2.019649000	0.014430000	1.973052000
C	-0.832265000	0.378722000	2.665648000
C	0.355118000	0.743015000	1.973052000
H	1.238626000	1.014091000	2.537593000
H	1.238625000	1.014089000	0.046990000
H	-0.832266000	0.378723000	-1.166202000
H	-2.903156000	-0.256645000	0.046990000
H	-2.903157000	-0.256646000	2.537593000
H	-0.832265000	0.378723000	3.750786000

0:	0.00 cm^{-1}	12:	803.24 cm^{-1}	24:	1377.98 cm^{-1}
1:	0.00 cm^{-1}	13:	909.53 cm^{-1}	25:	1386.64 cm^{-1}
2:	0.00 cm^{-1}	14:	967.90 cm^{-1}	26:	1432.04 cm^{-1}
3:	0.00 cm^{-1}	15:	992.63 cm^{-1}	27:	1464.17 cm^{-1}
4:	0.00 cm^{-1}	16:	993.63 cm^{-1}	28:	1558.09 cm^{-1}
5:	0.00 cm^{-1}	17:	1009.43 cm^{-1}	29:	1698.14 cm^{-1}
6:	281.12 cm^{-1}	18:	1013.54 cm^{-1}	30:	3202.39 cm^{-1}
7:	295.40 cm^{-1}	19:	1029.17 cm^{-1}	31:	3205.25 cm^{-1}
8:	337.17 cm^{-1}	20:	1047.53 cm^{-1}	32:	3220.07 cm^{-1}
9:	412.34 cm^{-1}	21:	1079.34 cm^{-1}	33:	3224.96 cm^{-1}
10:	598.21 cm^{-1}	22:	1198.15 cm^{-1}	34:	3231.28 cm^{-1}
11:	677.99 cm^{-1}	23:	1216.59 cm^{-1}	35:	3236.20 cm^{-1}

$[C_6H_6]^+$ – PBE0-D3BJ/def2-TZVP/CPCM(THF) (D_1)

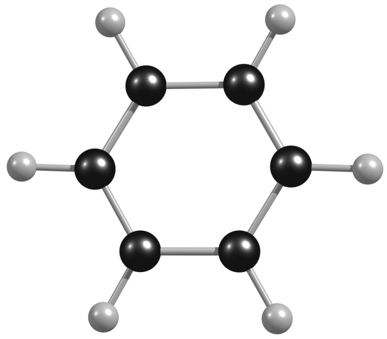

C	0.353267000	0.742540000	0.612078000
C	-0.832265000	0.378723000	-0.079936000
C	-2.017798000	0.014904000	0.612078000
C	-2.017798000	0.014904000	1.972504000
C	-0.832265000	0.378722000	2.664518000
C	0.353267000	0.742541000	1.972505000
H	1.235285000	1.013013000	2.538727000
H	1.235285000	1.013013000	0.045856000
H	-0.832265000	0.378723000	-1.164034000
H	-2.899816000	-0.255568000	0.045856000
H	-2.899816000	-0.255569000	2.538727000
H	-0.832265000	0.378723000	3.748617000

0:	0.00 cm^{-1}	12:	790.60 cm^{-1}	24:	1352.21 cm^{-1}
1:	0.00 cm^{-1}	13:	910.72 cm^{-1}	25:	1378.60 cm^{-1}
2:	0.00 cm^{-1}	14:	974.92 cm^{-1}	26:	1433.33 cm^{-1}
3:	0.00 cm^{-1}	15:	989.60 cm^{-1}	27:	1446.59 cm^{-1}
4:	0.00 cm^{-1}	16:	1000.00 cm^{-1}	28:	1556.60 cm^{-1}
5:	0.00 cm^{-1}	17:	1008.57 cm^{-1}	29:	1700.21 cm^{-1}
6:	282.28 cm^{-1}	18:	1012.77 cm^{-1}	30:	3217.66 cm^{-1}
7:	297.18 cm^{-1}	19:	1024.15 cm^{-1}	31:	3223.31 cm^{-1}
8:	341.78 cm^{-1}	20:	1043.23 cm^{-1}	32:	3229.24 cm^{-1}
9:	404.04 cm^{-1}	21:	1076.78 cm^{-1}	33:	3238.78 cm^{-1}
10:	598.88 cm^{-1}	22:	1174.47 cm^{-1}	34:	3239.17 cm^{-1}
11:	669.97 cm^{-1}	23:	1198.23 cm^{-1}	35:	3244.00 cm^{-1}

$[C_6H_5]^+$ – PBE0-D3BJ/def2-TZVP (S_0)

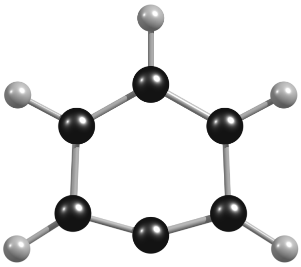

C	0.376268000	0.749512000	0.559423000
C	-0.831928000	0.378825000	-0.100243000
C	-1.972062000	0.029026000	0.603956000
C	-1.985032000	0.025045000	1.988871000
C	-0.834769000	0.377947000	2.753489000
C	0.065267000	0.654120000	1.834326000
H	1.291953000	1.030442000	0.057554000
H	-0.794464000	0.390312000	-1.185481000
H	-2.868406000	-0.245979000	0.062639000
H	-2.864807000	-0.244874000	2.565423000
H	-0.792433000	0.390931000	3.833912000

0:	0.00 cm^{-1}	11:	653.22 cm^{-1}	22:	1194.29 cm^{-1}
1:	0.00 cm^{-1}	12:	705.42 cm^{-1}	23:	1294.23 cm^{-1}
2:	0.00 cm^{-1}	13:	855.54 cm^{-1}	24:	1369.01 cm^{-1}
3:	0.00 cm^{-1}	14:	890.47 cm^{-1}	25:	1501.92 cm^{-1}
4:	0.00 cm^{-1}	15:	945.06 cm^{-1}	26:	1506.48 cm^{-1}
5:	0.00 cm^{-1}	16:	977.99 cm^{-1}	27:	1834.56 cm^{-1}
6:	383.82 cm^{-1}	17:	996.39 cm^{-1}	28:	3190.08 cm^{-1}
7:	412.27 cm^{-1}	18:	1008.94 cm^{-1}	29:	3193.61 cm^{-1}
8:	414.50 cm^{-1}	19:	1079.60 cm^{-1}	30:	3232.60 cm^{-1}
9:	477.28 cm^{-1}	20:	1110.34 cm^{-1}	31:	3246.89 cm^{-1}
10:	552.49 cm^{-1}	21:	1143.47 cm^{-1}	32:	3252.01 cm^{-1}

$[C_6H_5]^+$ – PBE0-D3BJ/def2-TZVP/CPCM(THF) (S_0)

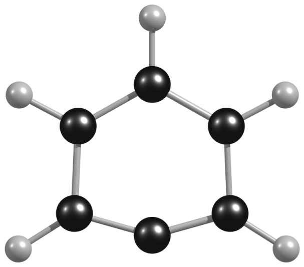

C	0.374030000	0.748944000	0.559890000
C	-0.831562000	0.379062000	-0.099716000
C	-1.971362000	0.029271000	0.604512000
C	-1.984753000	0.025141000	1.989185000
C	-0.835618000	0.377798000	2.750996000
C	0.064492000	0.654351000	1.833850000
H	1.290362000	1.029434000	0.058823000
H	-0.793092000	0.390809000	-1.184249000
H	-2.867404000	-0.245721000	0.063148000
H	-2.863596000	-0.244711000	2.566106000
H	-0.791911000	0.390930000	3.831321000

0:	0.00 cm^{-1}	11:	653.11 cm^{-1}	22:	1188.30 cm^{-1}
1:	0.00 cm^{-1}	12:	695.86 cm^{-1}	23:	1287.26 cm^{-1}
2:	0.00 cm^{-1}	13:	853.37 cm^{-1}	24:	1373.26 cm^{-1}
3:	0.00 cm^{-1}	14:	891.72 cm^{-1}	25:	1498.68 cm^{-1}
4:	0.00 cm^{-1}	15:	944.47 cm^{-1}	26:	1504.27 cm^{-1}
5:	0.00 cm^{-1}	16:	978.34 cm^{-1}	27:	1835.21 cm^{-1}
6:	386.21 cm^{-1}	17:	992.50 cm^{-1}	28:	3197.00 cm^{-1}
7:	416.41 cm^{-1}	18:	1000.89 cm^{-1}	29:	3202.63 cm^{-1}
8:	420.09 cm^{-1}	19:	1080.88 cm^{-1}	30:	3235.41 cm^{-1}
9:	488.02 cm^{-1}	20:	1095.86 cm^{-1}	31:	3244.17 cm^{-1}
10:	565.31 cm^{-1}	21:	1128.08 cm^{-1}	32:	3254.70 cm^{-1}

$[C_6H_5]^+$ – PBE0-D3BJ/def2-TZVP (T_1)

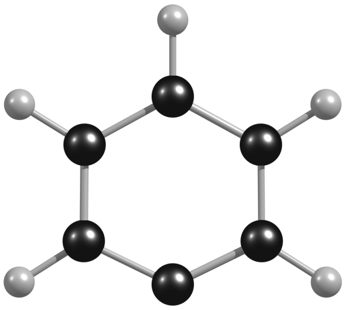

C	0.337383000	0.737580000	0.543054000
C	-0.799640000	0.388737000	-0.130089000
C	-1.963224000	0.031737000	0.609297000
C	-1.993604000	0.022427000	2.033039000
C	-0.867759000	0.367826000	2.726450000
C	0.243654000	0.708806000	1.942048000
H	1.255987000	1.019423000	0.041450000
H	-0.831381000	0.378999000	-1.213422000
H	-2.861678000	-0.243940000	0.066695000
H	-2.906401000	-0.257620000	2.545956000
H	-0.823752000	0.381332000	3.809389000

0:	0.00 cm^{-1}	11:	744.39 cm^{-1}	22:	1271.39 cm^{-1}
1:	0.00 cm^{-1}	12:	772.88 cm^{-1}	23:	1376.72 cm^{-1}
2:	0.00 cm^{-1}	13:	939.41 cm^{-1}	24:	1420.92 cm^{-1}
3:	0.00 cm^{-1}	14:	952.73 cm^{-1}	25:	1428.87 cm^{-1}
4:	0.00 cm^{-1}	15:	989.23 cm^{-1}	26:	1508.58 cm^{-1}
5:	0.00 cm^{-1}	16:	1005.33 cm^{-1}	27:	1641.71 cm^{-1}
6:	318.02 cm^{-1}	17:	1018.52 cm^{-1}	28:	3204.15 cm^{-1}
7:	324.79 cm^{-1}	18:	1036.15 cm^{-1}	29:	3209.13 cm^{-1}
8:	438.79 cm^{-1}	19:	1065.44 cm^{-1}	30:	3221.61 cm^{-1}
9:	587.36 cm^{-1}	20:	1146.95 cm^{-1}	31:	3225.19 cm^{-1}
10:	592.32 cm^{-1}	21:	1191.75 cm^{-1}	32:	3230.24 cm^{-1}

$[C_6H_5]^+$ – PBE0-D3BJ/def2-TZVP/CPCM(THF) (T_1)

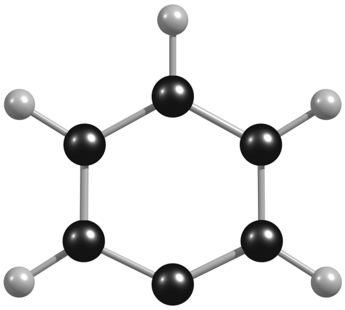

C	0.336399000	0.737432000	0.544803000
C	-0.799491000	0.388597000	-0.127767000
C	-1.962353000	0.031884000	0.609786000
C	-1.991598000	0.022423000	2.031952000
C	-0.866834000	0.368041000	2.724626000
C	0.241166000	0.708950000	1.940739000
H	1.253731000	1.018417000	0.041696000
H	-0.832147000	0.378231000	-1.210323000
H	-2.860353000	-0.242276000	0.067637000
H	-2.904265000	-0.257538000	2.543769000
H	-0.824668000	0.381146000	3.806949000

0:	0.00 cm^{-1}	11:	738.35 cm^{-1}	22:	1265.77 cm^{-1}
1:	0.00 cm^{-1}	12:	758.17 cm^{-1}	23:	1361.47 cm^{-1}
2:	0.00 cm^{-1}	13:	940.79 cm^{-1}	24:	1414.74 cm^{-1}
3:	0.00 cm^{-1}	14:	948.32 cm^{-1}	25:	1431.18 cm^{-1}
4:	0.00 cm^{-1}	15:	991.09 cm^{-1}	26:	1502.60 cm^{-1}
5:	0.00 cm^{-1}	16:	1009.23 cm^{-1}	27:	1642.56 cm^{-1}
6:	312.34 cm^{-1}	17:	1013.53 cm^{-1}	28:	3215.14 cm^{-1}
7:	328.61 cm^{-1}	18:	1033.22 cm^{-1}	29:	3221.00 cm^{-1}
8:	429.94 cm^{-1}	19:	1061.21 cm^{-1}	30:	3229.28 cm^{-1}
9:	588.60 cm^{-1}	20:	1137.97 cm^{-1}	31:	3233.28 cm^{-1}
10:	594.13 cm^{-1}	21:	1176.55 cm^{-1}	32:	3238.72 cm^{-1}

$[C_6H_6]^-$ – PBE0-D3BJ/def2-TZVP (D_1)

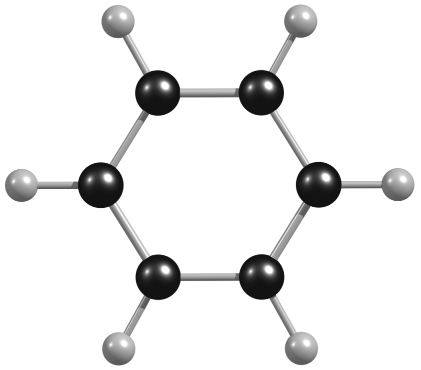

C	0.337708000	0.673887000	0.602077000
C	-0.804696000	0.240960000	-0.141706000
C	-1.992095000	-0.035812000	0.605784000
C	-2.004179000	0.010690000	1.975772000
C	-0.829693000	0.338344000	2.723399000
C	0.325630000	0.720386000	1.972071000
H	1.225097000	1.038079000	2.497863000
H	1.246473000	0.955588000	0.071806000
H	-0.827602000	0.310437000	-1.223992000
H	-2.905108000	-0.309012000	0.078338000
H	-2.926429000	-0.226705000	2.504438000
H	-0.871400000	0.481047000	3.797917000

0:	0.00 cm^{-1}	12:	616.13 cm^{-1}	24:	1268.38 cm^{-1}
1:	0.00 cm^{-1}	13:	668.14 cm^{-1}	25:	1343.98 cm^{-1}
2:	0.00 cm^{-1}	14:	673.34 cm^{-1}	26:	1377.04 cm^{-1}
3:	0.00 cm^{-1}	15:	727.05 cm^{-1}	27:	1442.42 cm^{-1}
4:	0.00 cm^{-1}	16:	860.05 cm^{-1}	28:	1515.61 cm^{-1}
5:	0.00 cm^{-1}	17:	868.01 cm^{-1}	29:	1649.90 cm^{-1}
6:	201.08 cm^{-1}	18:	940.21 cm^{-1}	30:	3102.18 cm^{-1}
7:	414.79 cm^{-1}	19:	978.05 cm^{-1}	31:	3105.23 cm^{-1}
8:	418.70 cm^{-1}	20:	1013.47 cm^{-1}	32:	3129.21 cm^{-1}
9:	426.34 cm^{-1}	21:	1037.03 cm^{-1}	33:	3134.89 cm^{-1}
10:	447.48 cm^{-1}	22:	1139.78 cm^{-1}	34:	3169.55 cm^{-1}
11:	572.40 cm^{-1}	23:	1179.14 cm^{-1}	35:	3176.17 cm^{-1}

$[C_6H_6]^-$ – PBE0-D3BJ/def2-TZVP/CPCM(THF) (D_1)

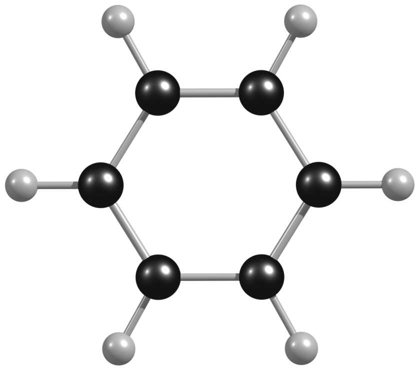

C	0.324823000	0.677608000	0.590554000
C	-0.805682000	0.307150000	-0.131572000
C	-2.015595000	-0.034299000	0.596713000
C	-1.995881000	0.022035000	1.986729000
C	-0.865375000	0.392517000	2.708865000
C	0.344554000	0.733922000	1.980581000
H	1.246498000	1.029214000	2.506530000
H	1.232131000	0.934051000	0.044522000
H	-0.780398000	0.275003000	-1.215832000
H	-2.917540000	-0.329570000	0.070769000
H	-2.903184000	-0.234394000	2.532776000
H	-0.890645000	0.424653000	3.793134000

0:	0.00 cm^{-1}	12:	567.08 cm^{-1}	24:	1271.71 cm^{-1}
1:	0.00 cm^{-1}	13:	612.96 cm^{-1}	25:	1345.75 cm^{-1}
2:	0.00 cm^{-1}	14:	641.29 cm^{-1}	26:	1378.91 cm^{-1}
3:	0.00 cm^{-1}	15:	646.78 cm^{-1}	27:	1468.82 cm^{-1}
4:	0.00 cm^{-1}	16:	912.80 cm^{-1}	28:	1476.92 cm^{-1}
5:	0.00 cm^{-1}	17:	919.96 cm^{-1}	29:	1543.25 cm^{-1}
6:	239.62 cm^{-1}	18:	921.58 cm^{-1}	30:	3126.34 cm^{-1}
7:	240.52 cm^{-1}	19:	971.53 cm^{-1}	31:	3127.95 cm^{-1}
8:	365.60 cm^{-1}	20:	1005.78 cm^{-1}	32:	3159.60 cm^{-1}
9:	468.92 cm^{-1}	21:	1046.22 cm^{-1}	33:	3172.00 cm^{-1}
10:	536.25 cm^{-1}	22:	1133.31 cm^{-1}	34:	3186.18 cm^{-1}
11:	545.19 cm^{-1}	23:	1134.68 cm^{-1}	35:	3192.15 cm^{-1}

$[C_6H_5]^–$ – PBE0-D3BJ/def2-TZVP (S_0)

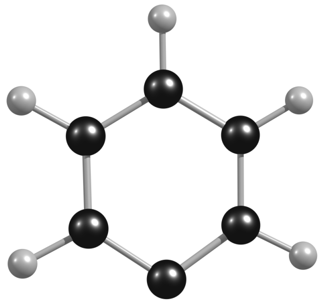

C	0.305259000	0.727714000	0.630821000
C	-0.838271000	0.376880000	-0.087724000
C	-2.000567000	0.020284000	0.586736000
C	-1.977840000	0.027265000	1.976859000
C	-0.811162000	0.385203000	2.653469000
C	0.405011000	0.758312000	2.039498000
H	1.183075000	0.997051000	0.031293000
H	-0.829161000	0.379682000	-1.179631000
H	-2.900838000	-0.255943000	0.043050000
H	-2.877303000	-0.248714000	2.531071000
H	-0.868616000	0.367573000	3.748424000

0:	0.00 cm^{-1}	11:	718.06 cm^{-1}	22:	1286.30 cm^{-1}
1:	0.00 cm^{-1}	12:	838.48 cm^{-1}	23:	1337.65 cm^{-1}
2:	0.00 cm^{-1}	13:	845.33 cm^{-1}	24:	1434.41 cm^{-1}
3:	0.00 cm^{-1}	14:	934.84 cm^{-1}	25:	1451.27 cm^{-1}
4:	0.00 cm^{-1}	15:	940.13 cm^{-1}	26:	1589.08 cm^{-1}
5:	0.00 cm^{-1}	16:	979.10 cm^{-1}	27:	1593.18 cm^{-1}
6:	345.74 cm^{-1}	17:	1013.40 cm^{-1}	28:	3016.92 cm^{-1}
7:	397.55 cm^{-1}	18:	1053.43 cm^{-1}	29:	3019.54 cm^{-1}
8:	594.02 cm^{-1}	19:	1068.60 cm^{-1}	30:	3084.70 cm^{-1}
9:	632.84 cm^{-1}	20:	1150.96 cm^{-1}	31:	3091.42 cm^{-1}
10:	671.58 cm^{-1}	21:	1194.74 cm^{-1}	32:	3150.92 cm^{-1}

$[C_6H_5]^-$ – PBE0-D3BJ/def2-TZVP/CPCM(THF) (S_0)

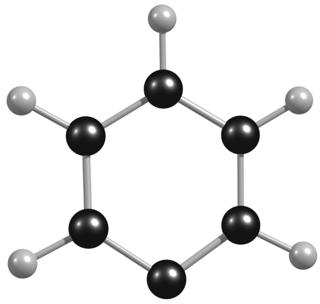

C	0.308034000	0.728454000	0.625589000
C	-0.837741000	0.377236000	-0.089524000
C	-1.997214000	0.021551000	0.588767000
C	-1.979218000	0.027155000	1.978262000
C	-0.814324000	0.384286000	2.658527000
C	0.395799000	0.755035000	2.033942000
H	1.184098000	0.997385000	0.030061000
H	-0.829170000	0.379858000	-1.177838000
H	-2.895952000	-0.254912000	0.045799000
H	-2.875770000	-0.248362000	2.530325000
H	-0.868955000	0.367620000	3.749957000

0:	0.00 cm^{-1}	11:	742.95 cm^{-1}	22:	1286.48 cm^{-1}
1:	0.00 cm^{-1}	12:	880.28 cm^{-1}	23:	1346.66 cm^{-1}
2:	0.00 cm^{-1}	13:	903.89 cm^{-1}	24:	1439.68 cm^{-1}
3:	0.00 cm^{-1}	14:	985.73 cm^{-1}	25:	1478.33 cm^{-1}
4:	0.00 cm^{-1}	15:	992.38 cm^{-1}	26:	1602.35 cm^{-1}
5:	0.00 cm^{-1}	16:	997.74 cm^{-1}	27:	1606.97 cm^{-1}
6:	366.22 cm^{-1}	17:	1011.47 cm^{-1}	28:	3073.69 cm^{-1}
7:	405.07 cm^{-1}	18:	1065.99 cm^{-1}	29:	3075.03 cm^{-1}
8:	602.05 cm^{-1}	19:	1073.04 cm^{-1}	30:	3140.02 cm^{-1}
9:	630.85 cm^{-1}	20:	1158.13 cm^{-1}	31:	3147.14 cm^{-1}
10:	704.84 cm^{-1}	21:	1205.05 cm^{-1}	32:	3181.44 cm^{-1}

$[C_6H_5]^-$ – PBE0-D3BJ/def2-TZVP (T_1)

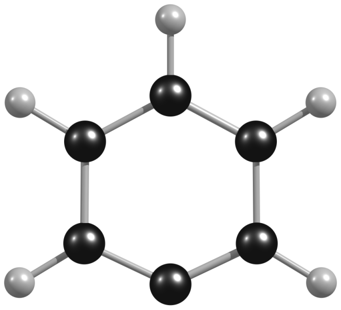

C	0.369019000	0.747292000	0.589830000
C	-0.847674000	0.374000000	-0.131056000
C	-1.977958000	0.027225000	0.600401000
C	-2.018392000	0.014822000	1.990000000
C	-0.813205000	0.384547000	2.731745000
C	0.263323000	0.714836000	1.953913000
H	1.277599000	1.026062000	0.067429000
H	-0.864785000	0.368727000	-1.217133000
H	-2.880728000	-0.249760000	0.055218000
H	-2.926157000	-0.263701000	2.517518000
H	-0.791457000	0.391257000	3.816002000

0:	0.00 cm^{-1}	11:	475.16 cm^{-1}	22:	1175.34 cm^{-1}
1:	0.00 cm^{-1}	12:	518.43 cm^{-1}	23:	1285.19 cm^{-1}
2:	0.00 cm^{-1}	13:	592.81 cm^{-1}	24:	1340.55 cm^{-1}
3:	0.00 cm^{-1}	14:	610.60 cm^{-1}	25:	1411.91 cm^{-1}
4:	0.00 cm^{-1}	15:	827.50 cm^{-1}	26:	1476.32 cm^{-1}
5:	0.00 cm^{-1}	16:	870.53 cm^{-1}	27:	1486.06 cm^{-1}
6:	219.02 cm^{-1}	17:	963.97 cm^{-1}	28:	3104.56 cm^{-1}
7:	348.59 cm^{-1}	18:	966.37 cm^{-1}	29:	3141.85 cm^{-1}
8:	366.63 cm^{-1}	19:	1002.15 cm^{-1}	30:	3154.60 cm^{-1}
9:	402.96 cm^{-1}	20:	1043.49 cm^{-1}	31:	3169.66 cm^{-1}
10:	407.01 cm^{-1}	21:	1120.13 cm^{-1}	32:	3178.54 cm^{-1}

$[C_6H_5]^-$ – PBE0-D3BJ/def2-TZVP/CPCM(THF) (T_1)

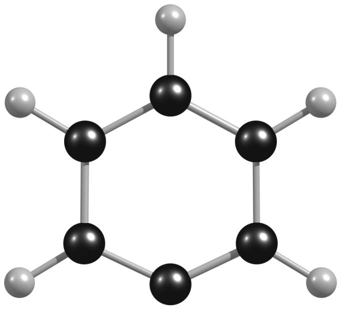

C	0.371390000	0.747898000	0.589989000
C	-0.847170000	0.373459000	-0.131719000
C	-1.979578000	0.026755000	0.598531000
C	-2.020021000	0.014425000	1.989672000
C	-0.813572000	0.384347000	2.733734000
C	0.261678000	0.714698000	1.954178000
H	1.278976000	1.026901000	0.066700000
H	-0.861163000	0.369839000	-1.216992000
H	-2.880767000	-0.249657000	0.053331000
H	-2.926021000	-0.263377000	2.518681000
H	-0.794165000	0.390018000	3.817762000

0:	0.00 cm^{-1}	11:	534.25 cm^{-1}	22:	1168.71 cm^{-1}
1:	0.00 cm^{-1}	12:	546.62 cm^{-1}	23:	1281.87 cm^{-1}
2:	0.00 cm^{-1}	13:	608.78 cm^{-1}	24:	1340.13 cm^{-1}
3:	0.00 cm^{-1}	14:	611.88 cm^{-1}	25:	1412.23 cm^{-1}
4:	0.00 cm^{-1}	15:	851.47 cm^{-1}	26:	1465.50 cm^{-1}
5:	0.00 cm^{-1}	16:	905.21 cm^{-1}	27:	1481.13 cm^{-1}
6:	269.39 cm^{-1}	17:	964.40 cm^{-1}	28:	3135.52 cm^{-1}
7:	408.49 cm^{-1}	18:	971.17 cm^{-1}	29:	3164.11 cm^{-1}
8:	441.02 cm^{-1}	19:	979.41 cm^{-1}	30:	3174.84 cm^{-1}
9:	477.00 cm^{-1}	20:	1041.84 cm^{-1}	31:	3188.36 cm^{-1}
10:	522.09 cm^{-1}	21:	1115.33 cm^{-1}	32:	3193.33 cm^{-1}

CH_4 – PBE0-D3BJ/def2-TZVP (S_0)

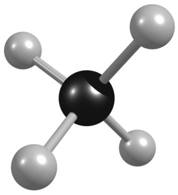

C	0.211448000	-0.005186000	0.123359000
H	0.740756000	-0.954500000	0.199385000
H	0.926477000	0.814927000	0.180739000
H	-0.503582000	0.078477000	0.941208000
H	-0.317860000	0.040346000	-0.827905000

0:	0.00 cm^{-1}	5:	0.00 cm^{-1}	10:	1551.25 cm^{-1}
1:	0.00 cm^{-1}	6:	1326.68 cm^{-1}	11:	3047.41 cm^{-1}
2:	0.00 cm^{-1}	7:	1326.80 cm^{-1}	12:	3167.31 cm^{-1}
3:	0.00 cm^{-1}	8:	1326.99 cm^{-1}	13:	3167.34 cm^{-1}
4:	0.00 cm^{-1}	9:	1551.17 cm^{-1}	14:	3167.37 cm^{-1}

CH_4 – PBE0-D3BJ/def2-TZVP/CPCM(THF) (S_0)

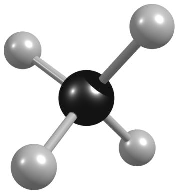

C	0.211443000	-0.005169000	0.123371000
H	0.741382000	-0.955655000	0.199489000
H	0.927359000	0.815935000	0.180777000
H	-0.504460000	0.078545000	0.942221000
H	-0.318486000	0.040410000	-0.829073000

0:	0.00 cm^{-1}	5:	0.00 cm^{-1}	10:	1521.97 cm^{-1}
1:	0.00 cm^{-1}	6:	1306.40 cm^{-1}	11:	3039.64 cm^{-1}
2:	0.00 cm^{-1}	7:	1306.95 cm^{-1}	12:	3159.48 cm^{-1}
3:	0.00 cm^{-1}	8:	1307.58 cm^{-1}	13:	3159.52 cm^{-1}
4:	0.00 cm^{-1}	9:	1521.04 cm^{-1}	14:	3159.55 cm^{-1}

$[CH_4]^+$ – PBE0-D3BJ/def2-TZVP (D_1)

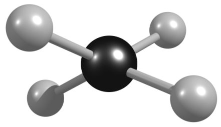

C	0.211447000	-0.005186000	0.123356000
H	0.840870000	-0.861254000	0.477957000
H	1.061718000	0.697244000	-0.072592000
H	-0.638820000	-0.178503000	0.831714000
H	-0.417976000	0.321763000	-0.743648000

0:	0.00 cm^{-1}	5:	0.00 cm^{-1}	10:	1432.03 cm^{-1}
1:	0.00 cm^{-1}	6:	297.57 cm^{-1}	11:	2717.57 cm^{-1}
2:	0.00 cm^{-1}	7:	301.81 cm^{-1}	12:	2822.13 cm^{-1}
3:	0.00 cm^{-1}	8:	1042.05 cm^{-1}	13:	2910.71 cm^{-1}
4:	0.00 cm^{-1}	9:	1286.48 cm^{-1}	14:	2910.99 cm^{-1}

$[CH_4]^+$ – PBE0-D3BJ/def2-TZVP/CPCM(THF) (D_1)

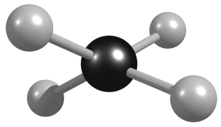

C	0.211285000	-0.005664000	0.122634000
H	0.840159000	-0.855235000	0.481460000
H	1.059987000	0.691414000	-0.075376000
H	-0.636851000	-0.181761000	0.826276000
H	-0.417342000	0.325309000	-0.738208000

0:	0.00 cm^{-1}	5:	0.00 cm^{-1}	10:	1433.08 cm^{-1}
1:	0.00 cm^{-1}	6:	115.97 cm^{-1}	11:	2762.65 cm^{-1}
2:	0.00 cm^{-1}	7:	191.40 cm^{-1}	12:	2862.71 cm^{-1}
3:	0.00 cm^{-1}	8:	1014.09 cm^{-1}	13:	2954.30 cm^{-1}
4:	0.00 cm^{-1}	9:	1271.73 cm^{-1}	14:	2955.30 cm^{-1}

$[CH_3]^+$ – PBE0-D3BJ/def2-TZVP (S_0)

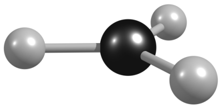

C	0.344056000	-0.016595000	0.361679000
H	0.721718000	-1.010810000	0.109057000
H	0.916102000	0.872350000	0.083375000
H	-0.605652000	0.088677000	0.892607000

0:	0.00 cm^{-1}	4:	0.00 cm^{-1}	8:	1417.22 cm^{-1}
1:	0.00 cm^{-1}	5:	0.00 cm^{-1}	9:	3033.34 cm^{-1}
2:	0.00 cm^{-1}	6:	1404.75 cm^{-1}	10:	3230.47 cm^{-1}
3:	0.00 cm^{-1}	7:	1404.83 cm^{-1}	11:	3230.69 cm^{-1}

$[CH_3]^+$ – PBE0-D3BJ/def2-TZVP/CPCM(THF) (S_0)

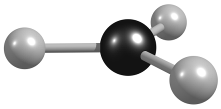

C	0.343868000	-0.016708000	0.361495000
H	0.720569000	-1.007401000	0.109986000
H	0.914226000	0.869344000	0.084347000
H	-0.602437000	0.088387000	0.890889000

0:	0.00 cm^{-1}	4:	0.00 cm^{-1}	8:	1413.93 cm^{-1}
1:	0.00 cm^{-1}	5:	0.00 cm^{-1}	9:	3072.60 cm^{-1}
2:	0.00 cm^{-1}	6:	1378.75 cm^{-1}	10:	3273.95 cm^{-1}
3:	0.00 cm^{-1}	7:	1383.41 cm^{-1}	11:	3277.46 cm^{-1}

$[CH_4]^{-}$ – PBE0-D3BJ/def2-TZVP (D_1)

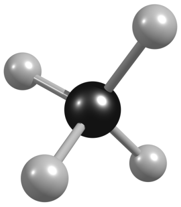

C	0.211446000	-0.005206000	0.123337000
H	0.752185000	-0.974980000	0.201043000
H	0.941884000	0.832612000	0.181992000
H	-0.518987000	0.080295000	0.958847000
H	-0.329289000	0.041344000	-0.848433000

0:	0.00 cm^{-1}	5:	0.00 cm^{-1}	10:	1557.27 cm^{-1}
1:	0.00 cm^{-1}	6:	1113.48 cm^{-1}	11:	2696.77 cm^{-1}
2:	0.00 cm^{-1}	7:	1113.70 cm^{-1}	12:	2813.07 cm^{-1}
3:	0.00 cm^{-1}	8:	1114.07 cm^{-1}	13:	2813.87 cm^{-1}
4:	0.00 cm^{-1}	9:	1557.17 cm^{-1}	14:	2814.65 cm^{-1}

$[CH_4]^{-}$ – PBE0-D3BJ/def2-TZVP/CPCM(THF) (D_1)

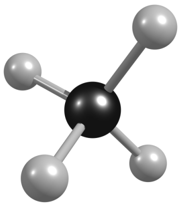

C	0.211204000	-0.005183000	0.123233000
H	0.753550000	-0.978003000	0.201909000
H	0.944164000	0.835375000	0.181499000
H	-0.521131000	0.079828000	0.961917000
H	-0.330548000	0.042047000	-0.851771000

0:	0.00 cm^{-1}	5:	0.00 cm^{-1}	10:	1473.92 cm^{-1}
1:	0.00 cm^{-1}	6:	1095.08 cm^{-1}	11:	2430.91 cm^{-1}
2:	0.00 cm^{-1}	7:	1100.36 cm^{-1}	12:	2438.47 cm^{-1}
3:	0.00 cm^{-1}	8:	1113.48 cm^{-1}	13:	2445.73 cm^{-1}
4:	0.00 cm^{-1}	9:	1462.43 cm^{-1}	14:	2647.71 cm^{-1}

Et$_2$O – PBE0-D3BJ/def2-TZVP (S$_0$)

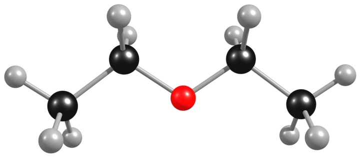

C	-1.226871000	0.391814000	0.053500000
O	-0.879129000	-0.960692000	-0.094980000
C	0.498865000	-1.187358000	0.053454000
C	0.773881000	-2.658905000	-0.121420000
H	0.830083000	-0.847336000	1.047408000
H	1.058256000	-0.598186000	-0.690335000
C	-2.716967000	0.535472000	-0.121579000
H	-0.689383000	1.001171000	-0.690172000
H	-0.917615000	0.751703000	1.047527000
H	0.230904000	-3.241647000	0.624983000
H	1.841050000	-2.862517000	-0.010626000
H	0.458742000	-2.992634000	-1.111792000
H	-3.021349000	0.192365000	-1.112113000
H	-3.014344000	1.580354000	-0.010408000
H	-3.249395000	-0.057294000	0.624535000

0:	0.00 cm^{-1}	15:	870.90 cm^{-1}	30:	1475.23 cm^{-1}
1:	0.00 cm^{-1}	16:	957.85 cm^{-1}	31:	1491.24 cm^{-1}
2:	0.00 cm^{-1}	17:	1081.47 cm^{-1}	32:	1494.40 cm^{-1}
3:	0.00 cm^{-1}	18:	1106.28 cm^{-1}	33:	1510.21 cm^{-1}
4:	0.00 cm^{-1}	19:	1167.43 cm^{-1}	34:	1529.76 cm^{-1}
5:	0.00 cm^{-1}	20:	1189.33 cm^{-1}	35:	2966.15 cm^{-1}
6:	104.86 cm^{-1}	21:	1194.23 cm^{-1}	36:	2977.22 cm^{-1}
7:	107.66 cm^{-1}	22:	1196.98 cm^{-1}	37:	2993.28 cm^{-1}
8:	193.29 cm^{-1}	23:	1296.70 cm^{-1}	38:	2997.49 cm^{-1}
9:	248.67 cm^{-1}	24:	1301.68 cm^{-1}	39:	3058.91 cm^{-1}
10:	253.66 cm^{-1}	25:	1376.70 cm^{-1}	40:	3059.08 cm^{-1}
11:	436.39 cm^{-1}	26:	1394.29 cm^{-1}	41:	3136.87 cm^{-1}
12:	443.33 cm^{-1}	27:	1407.11 cm^{-1}	42:	3137.52 cm^{-1}
13:	814.23 cm^{-1}	28:	1450.67 cm^{-1}	43:	3137.97 cm^{-1}
14:	826.37 cm^{-1}	29:	1474.65 cm^{-1}	44:	3138.39 cm^{-1}

Et$_2$O – PBE0-D3BJ/def2-TZVP/CPCM(THF) (S$_0$)

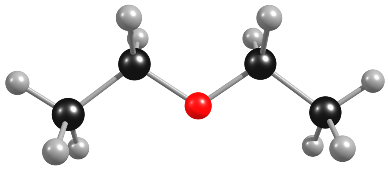

C	-1.231747000	0.386823000	0.051889000
O	-0.887587000	-0.969930000	-0.097515000
C	0.494257000	-1.192734000	0.052534000
C	0.781534000	-2.660693000	-0.121006000
H	0.822091000	-0.851435000	1.046553000
H	1.051059000	-0.601384000	-0.690644000
C	-2.719499000	0.542844000	-0.120634000
H	-0.692582000	0.993257000	-0.692123000
H	-0.920055000	0.744232000	1.045438000
H	0.248161000	-3.252927000	0.626586000
H	1.851702000	-2.850081000	-0.007303000
H	0.477328000	-3.002343000	-1.113391000
H	-3.033620000	0.207932000	-1.112242000
H	-3.002641000	1.592286000	-0.008523000
H	-3.261674000	-0.039538000	0.628363000

0:	0.00 cm^{-1}	15:	853.41 cm^{-1}	30:	1456.56 cm^{-1}
1:	0.00 cm^{-1}	16:	951.68 cm^{-1}	31:	1469.50 cm^{-1}
2:	0.00 cm^{-1}	17:	1066.90 cm^{-1}	32:	1471.41 cm^{-1}
3:	0.00 cm^{-1}	18:	1103.40 cm^{-1}	33:	1501.17 cm^{-1}
4:	0.00 cm^{-1}	19:	1123.48 cm^{-1}	34:	1520.29 cm^{-1}
5:	0.00 cm^{-1}	20:	1166.35 cm^{-1}	35:	2974.26 cm^{-1}
6:	100.56 cm^{-1}	21:	1171.01 cm^{-1}	36:	2982.65 cm^{-1}
7:	105.13 cm^{-1}	22:	1193.00 cm^{-1}	37:	2999.56 cm^{-1}
8:	193.38 cm^{-1}	23:	1298.77 cm^{-1}	38:	3007.20 cm^{-1}
9:	253.91 cm^{-1}	24:	1302.11 cm^{-1}	39:	3051.75 cm^{-1}
10:	259.32 cm^{-1}	25:	1374.52 cm^{-1}	40:	3051.80 cm^{-1}
11:	438.74 cm^{-1}	26:	1386.95 cm^{-1}	41:	3127.41 cm^{-1}
12:	439.67 cm^{-1}	27:	1399.77 cm^{-1}	42:	3127.59 cm^{-1}
13:	812.52 cm^{-1}	28:	1442.94 cm^{-1}	43:	3130.88 cm^{-1}
14:	823.83 cm^{-1}	29:	1456.37 cm^{-1}	44:	3131.19 cm^{-1}

[EtOCHCH$_3$]$^\bullet$ – PBE0-D3BJ/def2-TZVP (D$_1$)

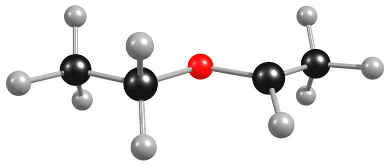

C	-1.281224000	0.312676000	-0.024941000
O	-0.889793000	-0.981995000	-0.073737000
C	0.507376000	-1.188040000	0.011516000
C	0.785472000	-2.663402000	-0.104439000
H	0.873788000	-0.791064000	0.966415000
H	1.005066000	-0.630839000	-0.794008000
C	-2.734383000	0.531419000	-0.165940000
H	-0.559109000	1.048926000	-0.374398000
H	0.292212000	-3.212262000	0.699722000
H	1.859524000	-2.849631000	-0.041303000
H	0.422775000	-3.051062000	-1.058172000
H	-3.105220000	0.251285000	-1.163722000
H	-2.975941000	1.582981000	-0.003927000
H	-3.291443000	-0.066492000	0.561274000

0:	0.00 cm^{-1}	14:	816.98 cm^{-1}	28:	1460.10 cm^{-1}
1:	0.00 cm^{-1}	15:	874.62 cm^{-1}	29:	1475.55 cm^{-1}
2:	0.00 cm^{-1}	16:	973.03 cm^{-1}	30:	1484.64 cm^{-1}
3:	0.00 cm^{-1}	17:	1015.68 cm^{-1}	31:	1492.92 cm^{-1}
4:	0.00 cm^{-1}	18:	1103.57 cm^{-1}	32:	1515.83 cm^{-1}
5:	0.00 cm^{-1}	19:	1124.40 cm^{-1}	33:	2979.53 cm^{-1}
6:	79.19 cm^{-1}	20:	1176.92 cm^{-1}	34:	3005.15 cm^{-1}
7:	108.72 cm^{-1}	21:	1183.10 cm^{-1}	35:	3041.49 cm^{-1}
8:	184.16 cm^{-1}	22:	1275.06 cm^{-1}	36:	3060.60 cm^{-1}
9:	194.31 cm^{-1}	23:	1298.75 cm^{-1}	37:	3071.74 cm^{-1}
10:	248.88 cm^{-1}	24:	1367.64 cm^{-1}	38:	3124.75 cm^{-1}
11:	432.34 cm^{-1}	25:	1392.48 cm^{-1}	39:	3138.98 cm^{-1}
12:	449.05 cm^{-1}	26:	1410.40 cm^{-1}	40:	3140.52 cm^{-1}
13:	560.25 cm^{-1}	27:	1449.49 cm^{-1}	41:	3141.90 cm^{-1}

[EtOCHCH$_3$]$^•$ – PBE0-D3BJ/def2-TZVP/CPCM(THF) (D$_1$)

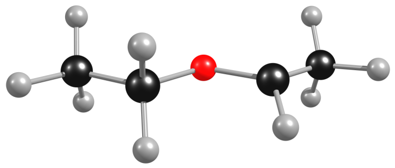

C	-1.287560000	0.306514000	-0.006328000
O	-0.899778000	-0.988716000	-0.059893000
C	0.505380000	-1.192650000	0.005679000
C	0.789854000	-2.665141000	-0.103685000
H	0.882714000	-0.788618000	0.952937000
H	0.986272000	-0.638802000	-0.810689000
C	-2.736357000	0.536555000	-0.169077000
H	-0.558732000	1.043405000	-0.341181000
H	0.318589000	-3.216589000	0.713216000
H	1.867605000	-2.837380000	-0.054675000
H	0.422285000	-3.065730000	-1.051349000
H	-3.092555000	0.264586000	-1.175489000
H	-2.971172000	1.590940000	-0.010492000
H	-3.317446000	-0.055875000	0.545367000

0:	0.00 cm^{-1}	14:	814.99 cm^{-1}	28:	1445.13 cm^{-1}
1:	0.00 cm^{-1}	15:	872.45 cm^{-1}	29:	1457.25 cm^{-1}
2:	0.00 cm^{-1}	16:	972.79 cm^{-1}	30:	1466.34 cm^{-1}
3:	0.00 cm^{-1}	17:	1015.70 cm^{-1}	31:	1470.68 cm^{-1}
4:	0.00 cm^{-1}	18:	1098.64 cm^{-1}	32:	1505.43 cm^{-1}
5:	0.00 cm^{-1}	19:	1116.18 cm^{-1}	33:	2973.60 cm^{-1}
6:	73.75 cm^{-1}	20:	1171.71 cm^{-1}	34:	3016.88 cm^{-1}
7:	104.20 cm^{-1}	21:	1175.35 cm^{-1}	35:	3053.01 cm^{-1}
8:	191.94 cm^{-1}	22:	1268.17 cm^{-1}	36:	3054.51 cm^{-1}
9:	198.86 cm^{-1}	23:	1300.56 cm^{-1}	37:	3063.64 cm^{-1}
10:	253.99 cm^{-1}	24:	1361.30 cm^{-1}	38:	3124.68 cm^{-1}
11:	436.05 cm^{-1}	25:	1386.21 cm^{-1}	39:	3131.78 cm^{-1}
12:	449.39 cm^{-1}	26:	1403.21 cm^{-1}	40:	3134.53 cm^{-1}
13:	568.79 cm^{-1}	27:	1434.31 cm^{-1}	41:	3139.96 cm^{-1}

$[Et_2O]^+$ – PBE0-D3BJ/def2-TZVP (D_1)

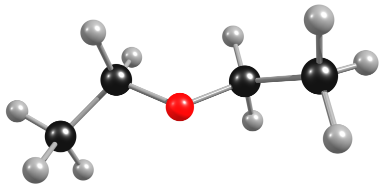

C	-1.308710000	0.301208000	0.345359000
O	-0.889704000	-0.965138000	-0.090698000
C	0.474008000	-1.229793000	-0.289750000
C	0.804859000	-2.657381000	0.064123000
H	1.081783000	-0.460448000	0.197834000
H	0.587326000	-1.087160000	-1.388906000
C	-2.641776000	0.666506000	-0.256180000
H	-0.503788000	1.031154000	0.209767000
H	-1.428841000	0.148618000	1.442495000
H	0.704417000	-2.810284000	1.139865000
H	1.839968000	-2.844684000	-0.224552000
H	0.162425000	-3.361610000	-0.464421000
H	-2.537694000	0.825747000	-1.330658000
H	-2.977812000	1.594733000	0.207867000
H	-3.389733000	-0.105157000	-0.074164000

0:	0.00 cm^{-1}	15:	845.08 cm^{-1}	30:	1414.92 cm^{-1}
1:	0.00 cm^{-1}	16:	893.79 cm^{-1}	31:	1459.63 cm^{-1}
2:	0.00 cm^{-1}	17:	964.23 cm^{-1}	32:	1465.89 cm^{-1}
3:	0.00 cm^{-1}	18:	1003.78 cm^{-1}	33:	1474.74 cm^{-1}
4:	0.00 cm^{-1}	19:	1027.83 cm^{-1}	34:	1476.07 cm^{-1}
5:	0.00 cm^{-1}	20:	1077.79 cm^{-1}	35:	2845.25 cm^{-1}
6:	38.68 cm^{-1}	21:	1093.01 cm^{-1}	36:	2872.27 cm^{-1}
7:	70.34 cm^{-1}	22:	1141.08 cm^{-1}	37:	3068.94 cm^{-1}
8:	189.95 cm^{-1}	23:	1165.62 cm^{-1}	38:	3070.14 cm^{-1}
9:	222.77 cm^{-1}	24:	1222.25 cm^{-1}	39:	3075.17 cm^{-1}
10:	235.60 cm^{-1}	25:	1321.55 cm^{-1}	40:	3081.39 cm^{-1}
11:	412.40 cm^{-1}	26:	1327.98 cm^{-1}	41:	3157.95 cm^{-1}
12:	458.94 cm^{-1}	27:	1365.52 cm^{-1}	42:	3158.12 cm^{-1}
13:	703.19 cm^{-1}	28:	1392.75 cm^{-1}	43:	3166.78 cm^{-1}
14:	741.94 cm^{-1}	29:	1404.18 cm^{-1}	44:	3168.02 cm^{-1}

$[Et_2O]^+$ – PBE0-D3BJ/def2-TZVP/CPCM(THF) (D_1)

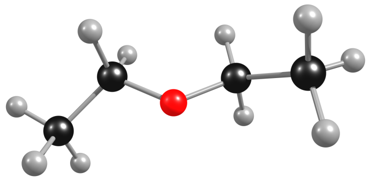

C	-1.291609000	0.310574000	0.261582000
O	-0.881471000	-0.952097000	-0.174172000
C	0.492231000	-1.221832000	-0.248583000
C	0.774206000	-2.664683000	0.054195000
H	1.049655000	-0.490116000	0.345358000
H	0.727199000	-1.003312000	-1.314987000
C	-2.668022000	0.641434000	-0.221959000
H	-0.509141000	1.047563000	0.035994000
H	-1.270418000	0.212486000	1.373050000
H	0.551540000	-2.879088000	1.101214000
H	1.835529000	-2.841352000	-0.125939000
H	0.195669000	-3.331235000	-0.586408000
H	-2.672767000	0.753471000	-1.307949000
H	-2.966344000	1.588586000	0.229542000
H	-3.389526000	-0.124090000	0.067043000

0:	0.00 cm^{-1}	15:	834.56 cm^{-1}	30:	1411.69 cm^{-1}
1:	0.00 cm^{-1}	16:	839.84 cm^{-1}	31:	1446.54 cm^{-1}
2:	0.00 cm^{-1}	17:	972.82 cm^{-1}	32:	1450.90 cm^{-1}
3:	0.00 cm^{-1}	18:	1025.28 cm^{-1}	33:	1454.10 cm^{-1}
4:	0.00 cm^{-1}	19:	1046.46 cm^{-1}	34:	1457.78 cm^{-1}
5:	0.00 cm^{-1}	20:	1064.76 cm^{-1}	35:	2825.91 cm^{-1}
6:	44.70 cm^{-1}	21:	1108.81 cm^{-1}	36:	2879.44 cm^{-1}
7:	91.29 cm^{-1}	22:	1150.82 cm^{-1}	37:	3045.05 cm^{-1}
8:	192.23 cm^{-1}	23:	1156.35 cm^{-1}	38:	3067.66 cm^{-1}
9:	221.21 cm^{-1}	24:	1222.55 cm^{-1}	39:	3069.54 cm^{-1}
10:	245.25 cm^{-1}	25:	1305.74 cm^{-1}	40:	3082.75 cm^{-1}
11:	424.19 cm^{-1}	26:	1314.35 cm^{-1}	41:	3153.38 cm^{-1}
12:	453.18 cm^{-1}	27:	1353.38 cm^{-1}	42:	3155.26 cm^{-1}
13:	678.39 cm^{-1}	28:	1383.42 cm^{-1}	43:	3161.11 cm^{-1}
14:	732.22 cm^{-1}	29:	1398.20 cm^{-1}	44:	3163.09 cm^{-1}

[$EtOCHCH_3$]$^+$ – PBE0-D3BJ/def2-TZVP (S_0)

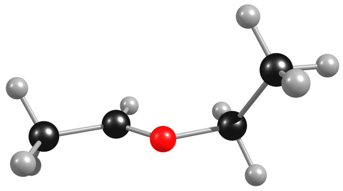

C	-1.307437000	0.133533000	-0.076736000
O	-0.832580000	-0.883360000	-0.612108000
C	0.608495000	-1.229886000	-0.493915000
C	0.727034000	-2.532555000	0.235408000
H	0.933319000	-1.290658000	-1.531812000
C	-2.717567000	0.450519000	-0.191674000
H	-0.631593000	0.787990000	0.485308000
H	0.378604000	-2.453392000	1.265997000
H	1.783349000	-2.809452000	0.255433000
H	0.180646000	-3.325014000	-0.276229000
H	-2.807581000	1.454583000	-0.628371000
H	-3.130791000	0.548951000	0.821322000
H	-3.269133000	-0.283551000	-0.774165000
H	1.099201000	-0.394242000	0.009071000

0:	0.00 cm^{-1}	14:	819.64 cm^{-1}	28:	1445.25 cm^{-1}
1:	0.00 cm^{-1}	15:	831.50 cm^{-1}	29:	1471.70 cm^{-1}
2:	0.00 cm^{-1}	16:	956.10 cm^{-1}	30:	1481.05 cm^{-1}
3:	0.00 cm^{-1}	17:	999.33 cm^{-1}	31:	1494.55 cm^{-1}
4:	0.00 cm^{-1}	18:	1122.27 cm^{-1}	32:	1702.45 cm^{-1}
5:	0.00 cm^{-1}	19:	1137.55 cm^{-1}	33:	3027.91 cm^{-1}
6:	72.90 cm^{-1}	20:	1164.22 cm^{-1}	34:	3070.76 cm^{-1}
7:	165.85 cm^{-1}	21:	1215.04 cm^{-1}	35:	3076.55 cm^{-1}
8:	172.25 cm^{-1}	22:	1294.33 cm^{-1}	36:	3092.69 cm^{-1}
9:	217.03 cm^{-1}	23:	1352.92 cm^{-1}	37:	3102.94 cm^{-1}
10:	314.34 cm^{-1}	24:	1379.52 cm^{-1}	38:	3149.20 cm^{-1}
11:	418.39 cm^{-1}	25:	1413.30 cm^{-1}	39:	3157.31 cm^{-1}
12:	513.90 cm^{-1}	26:	1421.08 cm^{-1}	40:	3177.90 cm^{-1}
13:	784.28 cm^{-1}	27:	1426.53 cm^{-1}	41:	3191.68 cm^{-1}

[EtOCHCH$_3$]$^+$ – PBE0-D3BJ/def2-TZVP/CPCM(THF) (S_0)

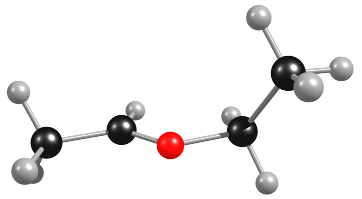

C	-1.301854000	0.101491000	-0.078107000
O	-0.828743000	-0.882299000	-0.682245000
C	0.603023000	-1.227187000	-0.534779000
C	0.707498000	-2.496543000	0.251132000
H	0.947149000	-1.334607000	-1.562150000
C	-2.701971000	0.436711000	-0.187219000
H	-0.632331000	0.707255000	0.538305000
H	0.335812000	-2.365184000	1.269114000
H	1.763365000	-2.771390000	0.303421000
H	0.166960000	-3.310987000	-0.233422000
H	-2.765700000	1.473717000	-0.542855000
H	-3.112614000	0.463062000	0.831027000
H	-3.259696000	-0.242287000	-0.827500000
H	1.093068000	-0.378287000	-0.057191000

0:	0.00 cm^{-1}	14:	821.21 cm^{-1}	28:	1441.40 cm^{-1}
1:	0.00 cm^{-1}	15:	831.92 cm^{-1}	29:	1455.11 cm^{-1}
2:	0.00 cm^{-1}	16:	956.94 cm^{-1}	30:	1461.99 cm^{-1}
3:	0.00 cm^{-1}	17:	1010.89 cm^{-1}	31:	1487.57 cm^{-1}
4:	0.00 cm^{-1}	18:	1123.99 cm^{-1}	32:	1679.90 cm^{-1}
5:	0.00 cm^{-1}	19:	1139.61 cm^{-1}	33:	3036.64 cm^{-1}
6:	74.24 cm^{-1}	20:	1166.62 cm^{-1}	34:	3065.37 cm^{-1}
7:	166.84 cm^{-1}	21:	1217.97 cm^{-1}	35:	3087.54 cm^{-1}
8:	177.36 cm^{-1}	22:	1296.60 cm^{-1}	36:	3112.25 cm^{-1}
9:	222.52 cm^{-1}	23:	1332.75 cm^{-1}	37:	3145.35 cm^{-1}
10:	328.48 cm^{-1}	24:	1381.38 cm^{-1}	38:	3146.12 cm^{-1}
11:	423.66 cm^{-1}	25:	1402.78 cm^{-1}	39:	3151.08 cm^{-1}
12:	530.78 cm^{-1}	26:	1406.02 cm^{-1}	40:	3187.10 cm^{-1}
13:	799.09 cm^{-1}	27:	1416.24 cm^{-1}	41:	3196.79 cm^{-1}

[EtOCHCH$_3$]$^+$ – PBE0-D3BJ/def2-TZVP (T_1)

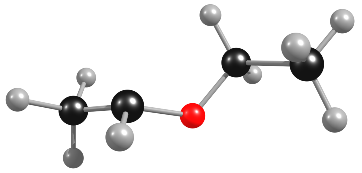

C	-1.599000000	-0.330062000	0.379861000
O	-1.010208000	-1.250533000	-0.447511000
C	0.371385000	-1.215459000	-0.664344000
C	0.993998000	-2.285875000	0.288933000
H	0.522314000	-1.555768000	-1.692826000
C	-2.482609000	0.656096000	-0.309378000
H	-1.902027000	-0.794085000	1.324492000
H	0.848103000	-1.980512000	1.323811000
H	2.056509000	-2.287309000	0.034956000
H	0.562787000	-3.266761000	0.100748000
H	-1.931015000	1.203148000	-1.076281000
H	-2.845337000	1.348495000	0.451070000
H	-3.339670000	0.155489000	-0.775457000
H	0.768735000	-0.223397000	-0.450546000

0:	0.00 cm^{-1}	14:	732.82 cm^{-1}	28:	1423.23 cm^{-1}
1:	0.00 cm^{-1}	15:	827.34 cm^{-1}	29:	1431.96 cm^{-1}
2:	0.00 cm^{-1}	16:	860.86 cm^{-1}	30:	1449.18 cm^{-1}
3:	0.00 cm^{-1}	17:	910.47 cm^{-1}	31:	1465.49 cm^{-1}
4:	0.00 cm^{-1}	18:	971.29 cm^{-1}	32:	1484.11 cm^{-1}
5:	0.00 cm^{-1}	19:	996.36 cm^{-1}	33:	3034.12 cm^{-1}
6:	57.39 cm^{-1}	20:	1036.03 cm^{-1}	34:	3055.24 cm^{-1}
7:	118.43 cm^{-1}	21:	1059.70 cm^{-1}	35:	3066.72 cm^{-1}
8:	160.53 cm^{-1}	22:	1116.73 cm^{-1}	36:	3071.53 cm^{-1}
9:	232.82 cm^{-1}	23:	1199.38 cm^{-1}	37:	3119.95 cm^{-1}
10:	271.29 cm^{-1}	24:	1279.15 cm^{-1}	38:	3158.79 cm^{-1}
11:	378.86 cm^{-1}	25:	1294.17 cm^{-1}	39:	3163.81 cm^{-1}
12:	477.29 cm^{-1}	26:	1361.93 cm^{-1}	40:	3168.07 cm^{-1}
13:	626.59 cm^{-1}	27:	1372.32 cm^{-1}	41:	3200.54 cm^{-1}

[$EtOCHCH_3$]$^+$ – PBE0-D3BJ/def2-TZVP/CPCM(THF) (T_1)

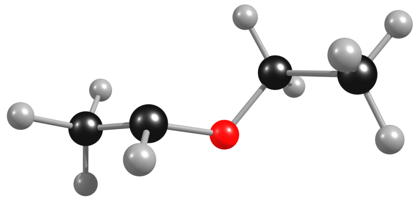

C	-1.896221000	-0.276106000	0.577744000
O	-1.095544000	-1.361347000	0.340198000
C	0.202320000	-1.185695000	-0.143715000
C	1.055627000	-2.391176000	0.067058000
H	0.030025000	-1.012878000	-1.242999000
C	-2.409936000	0.475985000	-0.591042000
H	-1.692042000	0.175351000	1.551999000
H	1.258681000	-2.521855000	1.133049000
H	2.000150000	-2.232523000	-0.454391000
H	0.583609000	-3.293538000	-0.324305000
H	-1.603822000	1.041731000	-1.077876000
H	-3.160659000	1.178519000	-0.229291000
H	-2.858858000	-0.202472000	-1.319835000
H	0.600636000	-0.220527000	0.200934000

0:	0.00 cm^{-1}	14:	668.17 cm^{-1}	28:	1400.44 cm^{-1}
1:	0.00 cm^{-1}	15:	775.48 cm^{-1}	29:	1421.52 cm^{-1}
2:	0.00 cm^{-1}	16:	877.36 cm^{-1}	30:	1426.02 cm^{-1}
3:	0.00 cm^{-1}	17:	974.94 cm^{-1}	31:	1441.46 cm^{-1}
4:	0.00 cm^{-1}	18:	1000.83 cm^{-1}	32:	1445.59 cm^{-1}
5:	0.00 cm^{-1}	19:	1029.46 cm^{-1}	33:	2726.68 cm^{-1}
6:	48.19 cm^{-1}	20:	1036.88 cm^{-1}	34:	3016.22 cm^{-1}
7:	128.78 cm^{-1}	21:	1081.55 cm^{-1}	35:	3017.75 cm^{-1}
8:	161.18 cm^{-1}	22:	1129.13 cm^{-1}	36:	3063.74 cm^{-1}
9:	204.48 cm^{-1}	23:	1134.07 cm^{-1}	37:	3102.26 cm^{-1}
10:	317.38 cm^{-1}	24:	1217.51 cm^{-1}	38:	3112.66 cm^{-1}
11:	364.48 cm^{-1}	25:	1258.10 cm^{-1}	39:	3146.43 cm^{-1}
12:	478.40 cm^{-1}	26:	1352.14 cm^{-1}	40:	3168.46 cm^{-1}
13:	515.31 cm^{-1}	27:	1369.89 cm^{-1}	41:	3169.66 cm^{-1}

[$Et_2O]^-$ – PBE0-D3BJ/def2-TZVP (D_1)

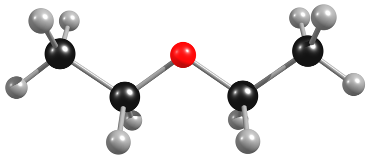

C	-1.241352000	0.347954000	0.045205000
O	-0.928101000	-1.014133000	-0.108833000
C	0.456345000	-1.205745000	0.045510000
C	0.802958000	-2.662151000	-0.118294000
H	0.776131000	-0.851921000	1.049662000
H	1.006840000	-0.599566000	-0.706333000
C	-2.722802000	0.564128000	-0.118102000
H	-0.686510000	0.949586000	-0.707102000
H	-0.916797000	0.698062000	1.049119000
H	0.297304000	-3.271125000	0.639156000
H	1.891426000	-2.791957000	0.006068000
H	0.528007000	-3.018639000	-1.117076000
H	-3.053897000	0.258251000	-1.116642000
H	-2.948592000	1.636838000	0.005880000
H	-3.284234000	0.006728000	0.639763000

0:	0.00 cm^{-1}	15:	865.25 cm^{-1}	30:	1437.73 cm^{-1}
1:	0.00 cm^{-1}	16:	941.07 cm^{-1}	31:	1447.32 cm^{-1}
2:	0.00 cm^{-1}	17:	1090.63 cm^{-1}	32:	1451.86 cm^{-1}
3:	0.00 cm^{-1}	18:	1102.61 cm^{-1}	33:	1483.75 cm^{-1}
4:	0.00 cm^{-1}	19:	1133.97 cm^{-1}	34:	1499.66 cm^{-1}
5:	0.00 cm^{-1}	20:	1151.63 cm^{-1}	35:	2789.71 cm^{-1}
6:	116.60 cm^{-1}	21:	1181.85 cm^{-1}	36:	2811.25 cm^{-1}
7:	118.35 cm^{-1}	22:	1183.59 cm^{-1}	37:	2813.56 cm^{-1}
8:	185.84 cm^{-1}	23:	1310.19 cm^{-1}	38:	2852.98 cm^{-1}
9:	255.48 cm^{-1}	24:	1332.08 cm^{-1}	39:	2909.04 cm^{-1}
10:	258.06 cm^{-1}	25:	1340.55 cm^{-1}	40:	2917.77 cm^{-1}
11:	409.10 cm^{-1}	26:	1342.38 cm^{-1}	41:	3025.46 cm^{-1}
12:	434.87 cm^{-1}	27:	1380.80 cm^{-1}	42:	3030.74 cm^{-1}
13:	760.79 cm^{-1}	28:	1382.99 cm^{-1}	43:	3071.54 cm^{-1}
14:	787.67 cm^{-1}	29:	1437.12 cm^{-1}	44:	3074.29 cm^{-1}

$[Et_2O]^{–}$ – PBE0-D3BJ/def2-TZVP/CPCM(THF) (D_1)

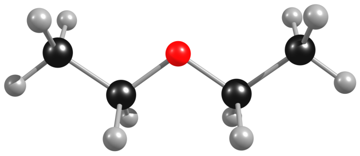

C	-1.242997000	0.344052000	0.059701000
O	-0.938705000	-1.025093000	-0.057074000
C	0.451812000	-1.208225000	0.062830000
C	0.806259000	-2.659158000	-0.119706000
H	0.789209000	-0.834910000	1.058848000
H	0.962927000	-0.582752000	-0.716869000
C	-2.720315000	0.567838000	-0.118764000
H	-0.666354000	0.904755000	-0.723752000
H	-0.897812000	0.715897000	1.053459000
H	0.303236000	-3.293329000	0.614743000
H	1.889264000	-2.788526000	-0.001318000
H	0.528535000	-2.999416000	-1.121893000
H	-3.037940000	0.258518000	-1.119142000
H	-2.945818000	1.635078000	-0.001825000
H	-3.304573000	0.011580000	0.618743000

0:	0.00 cm^{-1}	15:	850.34 cm^{-1}	30:	1430.68 cm^{-1}
1:	0.00 cm^{-1}	16:	929.43 cm^{-1}	31:	1448.05 cm^{-1}
2:	0.00 cm^{-1}	17:	1073.52 cm^{-1}	32:	1451.88 cm^{-1}
3:	0.00 cm^{-1}	18:	1091.97 cm^{-1}	33:	1472.62 cm^{-1}
4:	0.00 cm^{-1}	19:	1099.16 cm^{-1}	34:	1475.72 cm^{-1}
5:	0.00 cm^{-1}	20:	1141.20 cm^{-1}	35:	2424.67 cm^{-1}
6:	130.96 cm^{-1}	21:	1158.00 cm^{-1}	36:	2604.56 cm^{-1}
7:	142.03 cm^{-1}	22:	1163.99 cm^{-1}	37:	2700.36 cm^{-1}
8:	192.78 cm^{-1}	23:	1270.30 cm^{-1}	38:	2730.64 cm^{-1}
9:	222.34 cm^{-1}	24:	1310.39 cm^{-1}	39:	3003.60 cm^{-1}
10:	247.50 cm^{-1}	25:	1318.81 cm^{-1}	40:	3011.05 cm^{-1}
11:	413.06 cm^{-1}	26:	1355.12 cm^{-1}	41:	3083.84 cm^{-1}
12:	435.26 cm^{-1}	27:	1364.73 cm^{-1}	42:	3087.06 cm^{-1}
13:	746.11 cm^{-1}	28:	1367.49 cm^{-1}	43:	3111.10 cm^{-1}
14:	757.82 cm^{-1}	29:	1421.89 cm^{-1}	44:	3111.58 cm^{-1}

[EtOCHCH$_3$]$^–$ – PBE0-D3BJ/def2-TZVP (S_0)

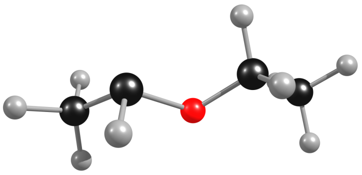

C	-1.221437000	0.388259000	0.057847000
O	-0.814140000	-0.855479000	-0.359535000
C	0.616624000	-1.102109000	-0.049746000
C	0.740642000	-2.608771000	-0.049521000
H	1.151462000	-0.750849000	-0.965605000
C	-2.722895000	0.519467000	-0.134262000
H	-0.714711000	1.198304000	-0.508399000
H	-0.939424000	0.550654000	1.114372000
H	0.270154000	-3.062370000	0.836090000
H	1.804954000	-2.879574000	-0.017460000
H	0.298659000	-3.139408000	-0.931034000
H	-2.990596000	0.389761000	-1.187401000
H	-3.078692000	1.502049000	0.196202000
H	-3.245859000	-0.251752000	0.439202000

0:	0.00 cm^{-1}	14:	798.46 cm^{-1}	28:	1432.61 cm^{-1}
1:	0.00 cm^{-1}	15:	866.74 cm^{-1}	29:	1461.84 cm^{-1}
2:	0.00 cm^{-1}	16:	915.47 cm^{-1}	30:	1484.38 cm^{-1}
3:	0.00 cm^{-1}	17:	998.78 cm^{-1}	31:	1486.73 cm^{-1}
4:	0.00 cm^{-1}	18:	1054.26 cm^{-1}	32:	1510.33 cm^{-1}
5:	0.00 cm^{-1}	19:	1089.69 cm^{-1}	33:	2730.97 cm^{-1}
6:	43.80 cm^{-1}	20:	1143.95 cm^{-1}	34:	2779.62 cm^{-1}
7:	88.22 cm^{-1}	21:	1183.58 cm^{-1}	35:	2862.76 cm^{-1}
8:	202.34 cm^{-1}	22:	1215.29 cm^{-1}	36:	2924.18 cm^{-1}
9:	250.78 cm^{-1}	23:	1248.21 cm^{-1}	37:	2964.28 cm^{-1}
10:	294.22 cm^{-1}	24:	1323.01 cm^{-1}	38:	3021.26 cm^{-1}
11:	414.87 cm^{-1}	25:	1346.20 cm^{-1}	39:	3033.34 cm^{-1}
12:	439.78 cm^{-1}	26:	1359.94 cm^{-1}	40:	3088.73 cm^{-1}
13:	773.76 cm^{-1}	27:	1384.81 cm^{-1}	41:	3098.08 cm^{-1}

[EtOCHCH$_3$]$^–$ – PBE0-D3BJ/def2-TZVP/CPCM(THF) (S$_0$)

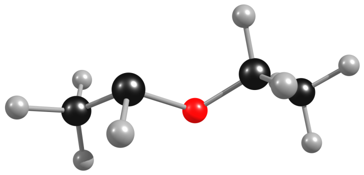

C	-1.232674000	0.386323000	0.071112000
O	-0.838201000	-0.904083000	-0.231299000
C	0.591611000	-1.127351000	0.015077000
C	0.764427000	-2.628207000	-0.073966000
H	1.100783000	-0.707258000	-0.885921000
C	-2.722671000	0.532945000	-0.143206000
H	-0.699651000	1.124690000	-0.558937000
H	-0.964217000	0.635162000	1.114483000
H	0.322056000	-3.151607000	0.785018000
H	1.831984000	-2.881406000	-0.080681000
H	0.317318000	-3.093139000	-0.979019000
H	-2.990262000	0.334719000	-1.185120000
H	-3.050470000	1.546678000	0.105471000
H	-3.275291000	-0.169285000	0.487737000

0:	0.00 cm^{-1}	14:	824.59 cm^{-1}	28:	1433.02 cm^{-1}
1:	0.00 cm^{-1}	15:	884.75 cm^{-1}	29:	1453.50 cm^{-1}
2:	0.00 cm^{-1}	16:	935.96 cm^{-1}	30:	1468.09 cm^{-1}
3:	0.00 cm^{-1}	17:	1023.87 cm^{-1}	31:	1477.80 cm^{-1}
4:	0.00 cm^{-1}	18:	1071.52 cm^{-1}	32:	1504.71 cm^{-1}
5:	0.00 cm^{-1}	19:	1106.63 cm^{-1}	33:	2760.75 cm^{-1}
6:	80.75 cm^{-1}	20:	1150.54 cm^{-1}	34:	2845.27 cm^{-1}
7:	121.84 cm^{-1}	21:	1179.71 cm^{-1}	35:	2903.94 cm^{-1}
8:	207.10 cm^{-1}	22:	1198.54 cm^{-1}	36:	2922.40 cm^{-1}
9:	259.25 cm^{-1}	23:	1270.07 cm^{-1}	37:	2998.00 cm^{-1}
10:	300.50 cm^{-1}	24:	1326.06 cm^{-1}	38:	3037.20 cm^{-1}
11:	430.32 cm^{-1}	25:	1361.44 cm^{-1}	39:	3059.15 cm^{-1}
12:	447.93 cm^{-1}	26:	1374.05 cm^{-1}	40:	3109.22 cm^{-1}
13:	807.04 cm^{-1}	27:	1400.93 cm^{-1}	41:	3112.36 cm^{-1}

[EtOCHCH$_3$]$^{-}$ – PBE0-D3BJ/def2-TZVP (T$_1$)

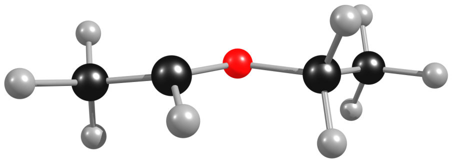

C	-1.247635000	0.356732000	0.020381000
O	-0.917118000	-1.029714000	0.061764000
C	0.407918000	-1.269747000	0.062023000
C	0.798396000	-2.675253000	-0.182982000
H	1.044600000	-0.464651000	-0.302374000
C	-2.730783000	0.552887000	-0.100574000
H	-0.754372000	0.819898000	-0.861678000
H	-0.864231000	0.855086000	0.928660000
H	0.258183000	-3.369075000	0.471330000
H	1.876024000	-2.809418000	-0.010672000
H	0.582725000	-2.974275000	-1.227342000
H	-3.097563000	0.136809000	-1.051012000
H	-2.924874000	1.646191000	-0.113860000
H	-3.276529000	0.122711000	0.747085000

0:	0.00 cm^{-1}	14:	741.32 cm^{-1}	28:	1421.97 cm^{-1}
1:	0.00 cm^{-1}	15:	866.21 cm^{-1}	29:	1432.82 cm^{-1}
2:	0.00 cm^{-1}	16:	959.16 cm^{-1}	30:	1442.55 cm^{-1}
3:	0.00 cm^{-1}	17:	1008.15 cm^{-1}	31:	1471.23 cm^{-1}
4:	0.00 cm^{-1}	18:	1086.60 cm^{-1}	32:	1494.67 cm^{-1}
5:	0.00 cm^{-1}	19:	1111.61 cm^{-1}	33:	2783.03 cm^{-1}
6:	84.07 cm^{-1}	20:	1153.65 cm^{-1}	34:	2803.92 cm^{-1}
7:	109.31 cm^{-1}	21:	1163.98 cm^{-1}	35:	2864.50 cm^{-1}
8:	181.01 cm^{-1}	22:	1260.08 cm^{-1}	36:	2891.85 cm^{-1}
9:	207.00 cm^{-1}	23:	1315.31 cm^{-1}	37:	2946.51 cm^{-1}
10:	272.89 cm^{-1}	24:	1324.44 cm^{-1}	38:	2973.31 cm^{-1}
11:	408.29 cm^{-1}	25:	1343.23 cm^{-1}	39:	3043.86 cm^{-1}
12:	434.02 cm^{-1}	26:	1346.01 cm^{-1}	40:	3047.94 cm^{-1}
13:	567.01 cm^{-1}	27:	1385.68 cm^{-1}	41:	3109.57 cm^{-1}

[$EtOCHCH_3$]$^-$ – PBE0-D3BJ/def2-TZVP/CPCM(THF) (T_1)

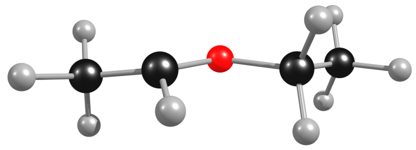

C	-1.239022000	0.379021000	0.017601000
O	-0.909474000	-1.007962000	0.058652000
C	0.415226000	-1.260557000	0.061964000
C	0.784635000	-2.674696000	-0.157867000
H	1.063099000	-0.463485000	-0.298380000
C	-2.724880000	0.551289000	-0.066834000
H	-0.833353000	0.793071000	-0.974025000
H	-0.777478000	0.900787000	0.868062000
H	0.256940000	-3.342490000	0.530967000
H	1.858990000	-2.816874000	-0.019279000
H	0.530152000	-3.002730000	-1.179905000
H	-2.998506000	0.298695000	-1.125610000
H	-2.976896000	1.608737000	0.094592000
H	-3.294690000	-0.064624000	0.630811000

0:	0.00 cm^{-1}	14:	728.78 cm^{-1}	28:	1419.67 cm^{-1}
1:	0.00 cm^{-1}	15:	864.17 cm^{-1}	29:	1431.11 cm^{-1}
2:	0.00 cm^{-1}	16:	967.89 cm^{-1}	30:	1443.34 cm^{-1}
3:	0.00 cm^{-1}	17:	1009.27 cm^{-1}	31:	1456.26 cm^{-1}
4:	0.00 cm^{-1}	18:	1096.20 cm^{-1}	32:	1466.81 cm^{-1}
5:	0.00 cm^{-1}	19:	1103.76 cm^{-1}	33:	2160.79 cm^{-1}
6:	95.76 cm^{-1}	20:	1117.17 cm^{-1}	34:	2541.87 cm^{-1}
7:	105.94 cm^{-1}	21:	1163.76 cm^{-1}	35:	2950.72 cm^{-1}
8:	188.24 cm^{-1}	22:	1246.03 cm^{-1}	36:	2974.03 cm^{-1}
9:	204.81 cm^{-1}	23:	1262.26 cm^{-1}	37:	2993.96 cm^{-1}
10:	241.00 cm^{-1}	24:	1295.19 cm^{-1}	38:	3059.70 cm^{-1}
11:	421.63 cm^{-1}	25:	1326.48 cm^{-1}	39:	3116.65 cm^{-1}
12:	440.99 cm^{-1}	26:	1355.08 cm^{-1}	40:	3120.94 cm^{-1}
13:	580.44 cm^{-1}	27:	1376.39 cm^{-1}	41:	3138.64 cm^{-1}

H_2CO – PBE0-D3BJ/def2-TZVP (S_0)

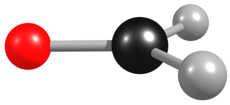

O	-0.650335000	-0.322278000	-0.973533000
C	-0.650332000	-0.322270000	0.221837000
H	-0.650331000	0.617692000	0.809739000
H	-0.650331000	-1.262225000	0.809750000

0:	0.00 cm^{-1}	4:	0.00 cm^{-1}	8:	1531.54 cm^{-1}
1:	0.00 cm^{-1}	5:	0.00 cm^{-1}	9:	1853.05 cm^{-1}
2:	0.00 cm^{-1}	6:	1206.27 cm^{-1}	10:	2900.32 cm^{-1}
3:	0.00 cm^{-1}	7:	1266.55 cm^{-1}	11:	2959.63 cm^{-1}

H_2CO – PBE0-D3BJ/def2-TZVP/CPCM(THF) (S_0)

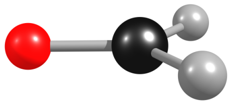

O	-0.650335000	-0.322278000	-0.975969000
C	-0.650332000	-0.322270000	0.224628000
H	-0.650331000	0.617068000	0.809561000
H	-0.650331000	-1.261601000	0.809573000

0:	0.00 cm^{-1}	4:	0.00 cm^{-1}	8:	1524.91 cm^{-1}
1:	0.00 cm^{-1}	5:	0.00 cm^{-1}	9:	1827.50 cm^{-1}
2:	0.00 cm^{-1}	6:	1219.83 cm^{-1}	10:	2929.96 cm^{-1}
3:	0.00 cm^{-1}	7:	1261.78 cm^{-1}	11:	2997.00 cm^{-1}

[HCO]$^{\bullet}$ – PBE0-D3BJ/def2-TZVP (D_1)

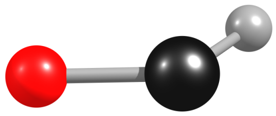

O	-0.650332000	-0.330169000	-0.971760000
C	-0.650339000	-0.315453000	0.196889000
H	-0.650329000	0.616468000	0.826608000

0:	0.00 cm^{-1}	3:	0.00 cm^{-1}	6:	1109.73 cm^{-1}
1:	0.00 cm^{-1}	4:	0.00 cm^{-1}	7:	1970.90 cm^{-1}
2:	0.00 cm^{-1}	5:	0.00 cm^{-1}	8:	2679.10 cm^{-1}

[HCO]$^{\bullet}$ – PBE0-D3BJ/def2-TZVP/CPCM(THF) (D_1)

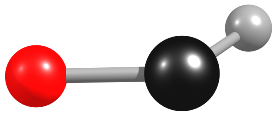

O	-0.650332000	-0.333773000	-0.974872000
C	-0.650339000	-0.309927000	0.196611000
H	-0.650329000	0.614546000	0.829998000

0:	0.00 cm^{-1}	3:	0.00 cm^{-1}	6:	1106.72 cm^{-1}
1:	0.00 cm^{-1}	4:	0.00 cm^{-1}	7:	1954.69 cm^{-1}
2:	0.00 cm^{-1}	5:	0.00 cm^{-1}	8:	2756.38 cm^{-1}

[H_2CO]$^{+}$ – PBE0-D3BJ/def2-TZVP (D_1)

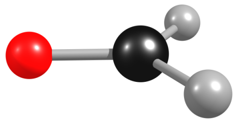

O	-0.650335000	-0.322277000	-0.951371000
C	-0.650332000	-0.322270000	0.233630000
H	-0.650331000	0.647631000	0.792760000
H	-0.650331000	-1.292164000	0.792773000

0:	0.00 cm^{-1}	4:	0.00 cm^{-1}	8:	1245.55 cm^{-1}
1:	0.00 cm^{-1}	5:	0.00 cm^{-1}	9:	1728.95 cm^{-1}
2:	0.00 cm^{-1}	6:	838.56 cm^{-1}	10:	2784.51 cm^{-1}
3:	0.00 cm^{-1}	7:	1072.71 cm^{-1}	11:	2878.61 cm^{-1}

[$H_2CO]^+$ – PBE0-D3BJ/def2-TZVP/CPCM(THF) (D_1)

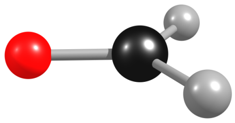

O	-0.650335000	-0.322277000	-0.959523000
C	-0.650332000	-0.322270000	0.220724000
H	-0.650331000	0.639216000	0.803290000
H	-0.650331000	-1.283749000	0.803303000

0:	0.00 cm^{-1}	4:	0.00 cm^{-1}	8:	1174.86 cm^{-1}
1:	0.00 cm^{-1}	5:	0.00 cm^{-1}	9:	1758.25 cm^{-1}
2:	0.00 cm^{-1}	6:	816.17 cm^{-1}	10:	2770.46 cm^{-1}
3:	0.00 cm^{-1}	7:	1058.81 cm^{-1}	11:	2838.37 cm^{-1}

$[HCO]^+$ – PBE0-D3BJ/def2-TZVP (S_0)

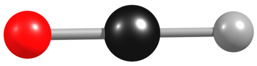

O	-0.649949000	-0.521908000	-0.955140000
C	-0.650333000	-0.009399000	0.017855000
H	-0.650718000	0.502153000	0.989022000

0:	0.00 cm^{-1}	3:	0.00 cm^{-1}	6:	874.65 cm^{-1}
1:	0.00 cm^{-1}	4:	0.00 cm^{-1}	7:	2290.36 cm^{-1}
2:	0.00 cm^{-1}	5:	874.20 cm^{-1}	8:	3227.13 cm^{-1}

$[HCO]^+$ – PBE0-D3BJ/def2-TZVP/CPCM(THF) (S_0)

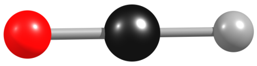

O	-0.651043000	-0.526909000	-0.956090000
C	-0.650334000	-0.008378000	0.016244000
H	-0.649623000	0.506134000	0.991583000

0:	0.00 cm^{-1}	3:	0.00 cm^{-1}	6:	835.15 cm^{-1}
1:	0.00 cm^{-1}	4:	0.00 cm^{-1}	7:	2268.16 cm^{-1}
2:	0.00 cm^{-1}	5:	0.00 cm^{-1}	8:	3175.91 cm^{-1}

[HCO]$^+$ – PBE0-D3BJ/def2-TZVP (T_1)

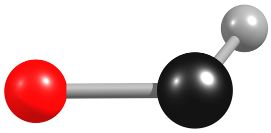

O	-0.650332000	-0.308831000	-1.006175000
C	-0.650340000	-0.344432000	0.253810000
H	-0.650328000	0.624108000	0.804101000

0:	0.00 cm^{-1}	3:	0.00 cm^{-1}	6:	960.64 cm^{-1}
1:	0.00 cm^{-1}	4:	0.00 cm^{-1}	7:	1596.63 cm^{-1}
2:	0.00 cm^{-1}	5:	0.00 cm^{-1}	8:	2918.10 cm^{-1}

[HCO]$^+$ – PBE0-D3BJ/def2-TZVP/CPCM(THF) (T_1)

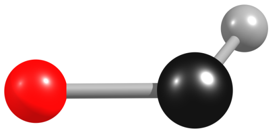

O	-0.650332000	-0.309172000	-1.007102000
C	-0.650340000	-0.344189000	0.254180000
H	-0.650328000	0.624208000	0.804658000

0:	0.00 cm^{-1}	3:	0.00 cm^{-1}	6:	937.77 cm^{-1}
1:	0.00 cm^{-1}	4:	0.00 cm^{-1}	7:	1591.97 cm^{-1}
2:	0.00 cm^{-1}	5:	0.00 cm^{-1}	8:	2944.98 cm^{-1}

$[H_2CO]^{-}$ – PBE0-D3BJ/def2-TZVP (D_1)

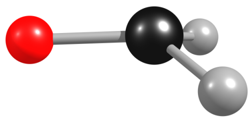

O	0.119666000	-0.210823000	-0.824925000
C	-0.601040000	-0.280974000	0.242109000
H	-0.706538000	0.610559000	0.913348000
H	-0.656306000	-1.236321000	0.825867000

0:	0.00 cm^{-1}	4:	0.00 cm^{-1}	8:	1371.33 cm^{-1}
1:	0.00 cm^{-1}	5:	0.00 cm^{-1}	9:	1496.61 cm^{-1}
2:	0.00 cm^{-1}	6:	661.50 cm^{-1}	10:	2720.74 cm^{-1}
3:	0.00 cm^{-1}	7:	1172.40 cm^{-1}	11:	2726.06 cm^{-1}

$[H_2CO]^{-}$ – PBE0-D3BJ/def2-TZVP/CPCM(THF) (D_1)

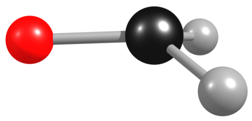

O	0.117197000	-0.211057000	-0.825392000
C	-0.594032000	-0.280963000	0.250474000
H	-0.708842000	0.610851000	0.909691000
H	-0.658542000	-1.236390000	0.821628000

0:	0.00 cm^{-1}	4:	0.00 cm^{-1}	8:	1370.68 cm^{-1}
1:	0.00 cm^{-1}	5:	0.00 cm^{-1}	9:	1502.72 cm^{-1}
2:	0.00 cm^{-1}	6:	620.15 cm^{-1}	10:	2797.11 cm^{-1}
3:	0.00 cm^{-1}	7:	1170.97 cm^{-1}	11:	2799.21 cm^{-1}

[HCO]$^-$ – PBE0-D3BJ/def2-TZVP (S_0)

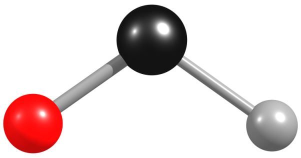

O	-0.650332000	-0.277826000	-0.982583000
C	-0.650341000	-0.436624000	0.234111000
H	-0.650327000	0.685296000	0.800208000

0:	0.00 cm^{-1}	3:	0.00 cm^{-1}	6:	1308.76 cm^{-1}
1:	0.00 cm^{-1}	4:	0.00 cm^{-1}	7:	1350.76 cm^{-1}
2:	0.00 cm^{-1}	5:	0.00 cm^{-1}	8:	1790.74 cm^{-1}

[HCO]$^-$ – PBE0-D3BJ/def2-TZVP/CPCM(THF) (S_0)

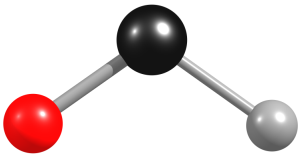

O	-0.650332000	-0.276233000	-0.976907000
C	-0.650341000	-0.420210000	0.240668000
H	-0.650328000	0.667289000	0.787975000

0:	0.00 cm^{-1}	3:	0.00 cm^{-1}	6:	1343.35 cm^{-1}
1:	0.00 cm^{-1}	4:	0.00 cm^{-1}	7:	1408.06 cm^{-1}
2:	0.00 cm^{-1}	5:	0.00 cm^{-1}	8:	1880.52 cm^{-1}

[HCO]⁻ – PBE0-D3BJ/def2-TZVP (T_1)

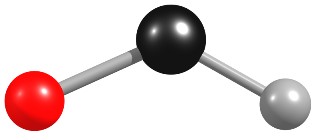

O	-0.650332000	-0.345750000	-1.031527000
C	-0.650339000	-0.307794000	0.231397000
H	-0.650329000	0.624391000	0.851867000

0:	0.00 cm^{-1}	3:	0.00 cm^{-1}	6:	1042.45 cm^{-1}
1:	0.00 cm^{-1}	4:	0.00 cm^{-1}	7:	1504.15 cm^{-1}
2:	0.00 cm^{-1}	5:	0.00 cm^{-1}	8:	2699.31 cm^{-1}

[HCO]⁻ – PBE0-D3BJ/def2-TZVP/CPCM(THF) (T_1)

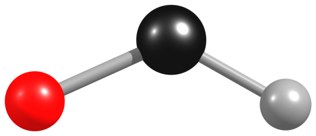

O	-0.650332000	-0.343665000	-1.028074000
C	-0.650339000	-0.306967000	0.232418000
H	-0.650329000	0.621478000	0.847392000

0:	0.00 cm^{-1}	3:	0.00 cm^{-1}	6:	1028.25 cm^{-1}
1:	0.00 cm^{-1}	4:	0.00 cm^{-1}	7:	1522.44 cm^{-1}
2:	0.00 cm^{-1}	5:	0.00 cm^{-1}	8:	2794.50 cm^{-1}

C_4H_5N – PBE0-D3BJ/def2-TZVP (S_0)

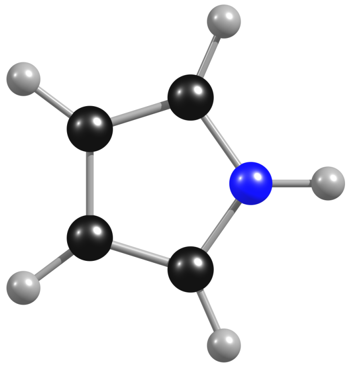

N	0.008955000	0.621183000	0.985507000
C	0.008955000	-0.741594000	1.021766000
C	0.008956000	-1.192848000	-0.272981000
C	0.008954000	-0.054702000	-1.116320000
C	0.008956000	1.052597000	-0.307688000
H	0.008957000	2.103282000	-0.547847000
H	0.008953000	-0.047324000	-2.194723000
H	0.008957000	-2.226695000	-0.579860000
H	0.008954000	-1.277223000	1.957027000
H	0.008955000	1.218218000	1.791248000

0:	0.00 cm^{-1}	10:	733.95 cm^{-1}	20:	1313.90 cm^{-1}
1:	0.00 cm^{-1}	11:	833.40 cm^{-1}	21:	1440.12 cm^{-1}
2:	0.00 cm^{-1}	12:	875.12 cm^{-1}	22:	1478.74 cm^{-1}
3:	0.00 cm^{-1}	13:	878.86 cm^{-1}	23:	1519.37 cm^{-1}
4:	0.00 cm^{-1}	14:	903.42 cm^{-1}	24:	1590.13 cm^{-1}
5:	0.00 cm^{-1}	15:	1044.83 cm^{-1}	25:	3257.30 cm^{-1}
6:	488.82 cm^{-1}	16:	1076.44 cm^{-1}	26:	3267.61 cm^{-1}
7:	631.47 cm^{-1}	17:	1101.68 cm^{-1}	27:	3283.49 cm^{-1}
8:	648.28 cm^{-1}	18:	1169.19 cm^{-1}	28:	3287.72 cm^{-1}
9:	695.37 cm^{-1}	19:	1195.42 cm^{-1}	29:	3704.70 cm^{-1}

C_4H_5N – PBE0-D3BJ/def2-TZVP/CPCM(THF) (S_0)

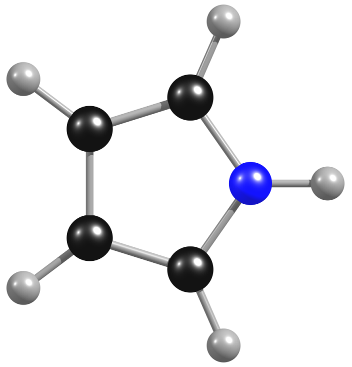

N	0.008955000	0.619907000	0.983664000
C	0.008954000	-0.740235000	1.022237000
C	0.008957000	-1.195428000	-0.274875000
C	0.008953000	-0.055673000	-1.119487000
C	0.008957000	1.052676000	-0.306345000
H	0.008958000	2.105308000	-0.542023000
H	0.008952000	-0.047466000	-2.199251000
H	0.008958000	-2.230915000	-0.581066000
H	0.008954000	-1.272223000	1.960619000
H	0.008954000	1.218944000	1.792656000

0:	0.00 cm^{-1}	10:	738.35 cm^{-1}	20:	1311.93 cm^{-1}
1:	0.00 cm^{-1}	11:	855.36 cm^{-1}	21:	1436.63 cm^{-1}
2:	0.00 cm^{-1}	12:	878.11 cm^{-1}	22:	1476.13 cm^{-1}
3:	0.00 cm^{-1}	13:	889.39 cm^{-1}	23:	1514.66 cm^{-1}
4:	0.00 cm^{-1}	14:	898.08 cm^{-1}	24:	1580.81 cm^{-1}
5:	0.00 cm^{-1}	15:	1033.09 cm^{-1}	25:	3244.50 cm^{-1}
6:	560.86 cm^{-1}	16:	1067.61 cm^{-1}	26:	3259.16 cm^{-1}
7:	636.20 cm^{-1}	17:	1092.82 cm^{-1}	27:	3277.49 cm^{-1}
8:	659.05 cm^{-1}	18:	1154.39 cm^{-1}	28:	3281.96 cm^{-1}
9:	718.93 cm^{-1}	19:	1190.72 cm^{-1}	29:	3663.55 cm^{-1}

[C_4H_4N]$^{\bullet}$ – PBE0-D3BJ/def2-TZVP (D_1)

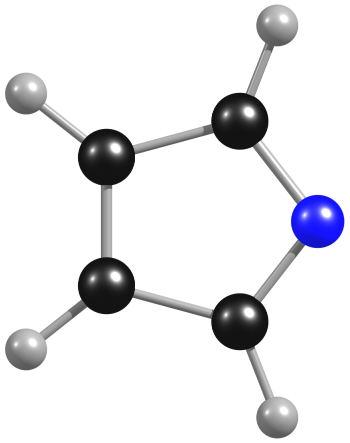

N	0.008957000	0.666417000	1.046541000
C	0.008955000	-0.669697000	1.019091000
C	0.008955000	-1.198485000	-0.332764000
C	0.008956000	-0.110263000	-1.139118000
C	0.008954000	1.029109000	-0.239681000
H	0.008954000	2.074346000	-0.525086000
H	0.008957000	-0.074110000	-2.218060000
H	0.008954000	-2.241222000	-0.612279000
H	0.008956000	-1.247027000	1.935967000

0:	0.00 cm^{-1}	9:	716.41 cm^{-1}	18:	1217.60 cm^{-1}
1:	0.00 cm^{-1}	10:	835.41 cm^{-1}	19:	1294.78 cm^{-1}
2:	0.00 cm^{-1}	11:	848.52 cm^{-1}	20:	1367.68 cm^{-1}
3:	0.00 cm^{-1}	12:	881.28 cm^{-1}	21:	1462.60 cm^{-1}
4:	0.00 cm^{-1}	13:	916.87 cm^{-1}	22:	1580.72 cm^{-1}
5:	0.00 cm^{-1}	14:	948.51 cm^{-1}	23:	3214.85 cm^{-1}
6:	496.59 cm^{-1}	15:	1055.91 cm^{-1}	24:	3218.84 cm^{-1}
7:	540.52 cm^{-1}	16:	1099.32 cm^{-1}	25:	3250.53 cm^{-1}
8:	691.99 cm^{-1}	17:	1100.74 cm^{-1}	26:	3270.35 cm^{-1}

$[C_4H_4N]^{\bullet}$ – PBE0-D3BJ/def2-TZVP/CPCM(THF) (D_1)

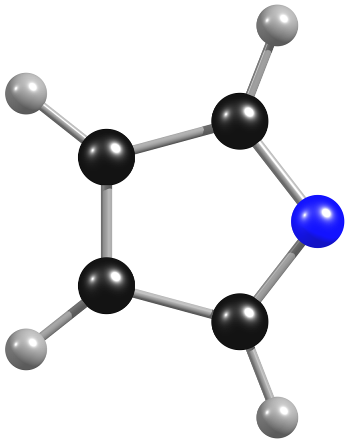

N	0.008952000	0.665702000	1.045569000
C	0.008958000	-0.673818000	1.020460000
C	0.008954000	-1.197419000	-0.329974000
C	0.008957000	-0.107923000	-1.137314000
C	0.008955000	1.031616000	-0.243217000
H	0.008955000	2.075808000	-0.532637000
H	0.008958000	-0.068697000	-2.216743000
H	0.008952000	-2.241516000	-0.606719000
H	0.008957000	-1.254686000	1.935186000

0:	0.00 cm^{-1}	9:	720.64 cm^{-1}	18:	1208.26 cm^{-1}
1:	0.00 cm^{-1}	10:	850.11 cm^{-1}	19:	1296.87 cm^{-1}
2:	0.00 cm^{-1}	11:	867.83 cm^{-1}	20:	1364.86 cm^{-1}
3:	0.00 cm^{-1}	12:	885.14 cm^{-1}	21:	1460.56 cm^{-1}
4:	0.00 cm^{-1}	13:	937.56 cm^{-1}	22:	1575.91 cm^{-1}
5:	0.00 cm^{-1}	14:	957.74 cm^{-1}	23:	3219.83 cm^{-1}
6:	500.50 cm^{-1}	15:	1053.61 cm^{-1}	24:	3222.78 cm^{-1}
7:	540.83 cm^{-1}	16:	1094.43 cm^{-1}	25:	3253.32 cm^{-1}
8:	709.76 cm^{-1}	17:	1098.38 cm^{-1}	26:	3269.93 cm^{-1}

$[C_4H_5N]^+$ – PBE0-D3BJ/def2-TZVP (D_1)

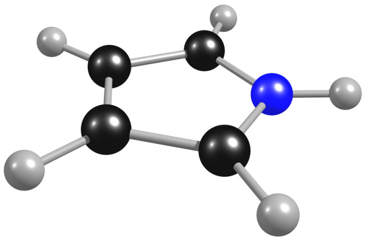

N	0.008955000	0.634523000	1.003505000
C	0.008956000	-0.716771000	1.029116000
C	0.008955000	-1.191619000	-0.314991000
C	0.008956000	-0.095256000	-1.127374000
C	0.008955000	1.052411000	-0.281792000
H	0.008955000	2.102511000	-0.539884000
H	0.008956000	-0.065021000	-2.206115000
H	0.008955000	-2.232443000	-0.600101000
H	0.008956000	-1.269380000	1.958600000
H	0.008955000	1.235940000	1.815165000

0:	0.00 cm^{-1}	10:	754.45 cm^{-1}	20:	1292.35 cm^{-1}
1:	0.00 cm^{-1}	11:	860.49 cm^{-1}	21:	1336.93 cm^{-1}
2:	0.00 cm^{-1}	12:	896.22 cm^{-1}	22:	1487.37 cm^{-1}
3:	0.00 cm^{-1}	13:	910.83 cm^{-1}	23:	1503.23 cm^{-1}
4:	0.00 cm^{-1}	14:	981.16 cm^{-1}	24:	1570.95 cm^{-1}
5:	0.00 cm^{-1}	15:	1008.19 cm^{-1}	25:	3258.16 cm^{-1}
6:	490.85 cm^{-1}	16:	1052.69 cm^{-1}	26:	3263.45 cm^{-1}
7:	509.95 cm^{-1}	17:	1086.49 cm^{-1}	27:	3273.79 cm^{-1}
8:	680.20 cm^{-1}	18:	1104.10 cm^{-1}	28:	3285.91 cm^{-1}
9:	750.48 cm^{-1}	19:	1195.79 cm^{-1}	29:	3626.09 cm^{-1}

$[C_4H_5N]^+$ – PBE0-D3BJ/def2-TZVP/CPCM(THF) (D_1)

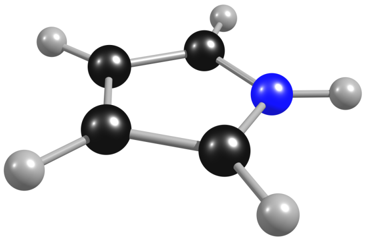

N	0.008955000	0.631390000	0.998771000
C	0.008955000	-0.714981000	1.027990000
C	0.008955000	-1.191549000	-0.315862000
C	0.008956000	-0.096233000	-1.127743000
C	0.008955000	1.051000000	-0.280704000
H	0.008955000	2.101095000	-0.534165000
H	0.008956000	-0.062735000	-2.205886000
H	0.008955000	-2.232822000	-0.597455000
H	0.008956000	-1.263347000	1.958672000
H	0.008955000	1.233077000	1.812511000

0:	0.00 cm^{-1}	10:	761.58 cm^{-1}	20:	1273.75 cm^{-1}
1:	0.00 cm^{-1}	11:	868.33 cm^{-1}	21:	1326.10 cm^{-1}
2:	0.00 cm^{-1}	12:	896.05 cm^{-1}	22:	1488.11 cm^{-1}
3:	0.00 cm^{-1}	13:	915.61 cm^{-1}	23:	1497.20 cm^{-1}
4:	0.00 cm^{-1}	14:	973.81 cm^{-1}	24:	1569.98 cm^{-1}
5:	0.00 cm^{-1}	15:	1006.17 cm^{-1}	25:	3271.05 cm^{-1}
6:	493.11 cm^{-1}	16:	1062.73 cm^{-1}	26:	3277.10 cm^{-1}
7:	516.73 cm^{-1}	17:	1078.75 cm^{-1}	27:	3286.74 cm^{-1}
8:	698.70 cm^{-1}	18:	1088.01 cm^{-1}	28:	3294.57 cm^{-1}
9:	742.56 cm^{-1}	19:	1197.43 cm^{-1}	29:	3606.23 cm^{-1}

$[C_4H_4N]^+$ – PBE0-D3BJ/def2-TZVP (S_0)

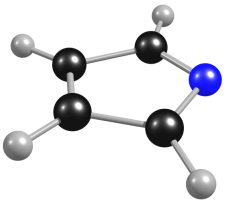

N	0.095620000	0.641856000	1.061531000
C	0.022249000	-0.647847000	1.039924000
C	-0.019226000	-1.201578000	-0.410916000
C	0.090954000	-0.123608000	-1.189979000
C	0.029840000	0.992972000	-0.255636000
H	-0.140670000	2.035440000	-0.523107000
H	0.097028000	-0.046187000	-2.266693000
H	-0.099636000	-2.246942000	-0.678748000
H	0.022466000	-1.249077000	1.946301000

0:	0.00 cm^{-1}	9:	702.53 cm^{-1}	18:	1281.28 cm^{-1}
1:	0.00 cm^{-1}	10:	824.89 cm^{-1}	19:	1299.46 cm^{-1}
2:	0.00 cm^{-1}	11:	867.91 cm^{-1}	20:	1327.71 cm^{-1}
3:	0.00 cm^{-1}	12:	939.05 cm^{-1}	21:	1568.20 cm^{-1}
4:	0.00 cm^{-1}	13:	997.75 cm^{-1}	22:	1635.77 cm^{-1}
5:	0.00 cm^{-1}	14:	1026.36 cm^{-1}	23:	3191.53 cm^{-1}
6:	121.63 cm^{-1}	15:	1054.47 cm^{-1}	24:	3205.87 cm^{-1}
7:	337.89 cm^{-1}	16:	1091.39 cm^{-1}	25:	3252.12 cm^{-1}
8:	537.29 cm^{-1}	17:	1156.01 cm^{-1}	26:	3280.74 cm^{-1}

$[C_4H_4N]^+$ – PBE0-D3BJ/def2-TZVP/CPCM(THF) (S_0)

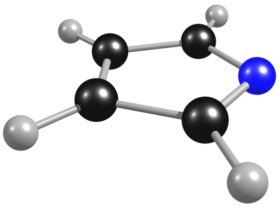

e

N	0.008924000	0.664086000	1.078527000
C	0.008954000	-0.635750000	1.055352000
C	0.008962000	-1.198957000	-0.375332000
C	0.008967000	-0.126822000	-1.161488000
C	0.008970000	1.007179000	-0.238524000
H	0.008935000	2.055015000	-0.528372000
H	0.008969000	-0.047738000	-2.237021000
H	0.008968000	-2.251361000	-0.618929000
H	0.008948000	-1.236584000	1.960399000

0:	0.00 cm^{-1}	9:	710.32 cm^{-1}	18:	1246.82 cm^{-1}
1:	0.00 cm^{-1}	10:	841.92 cm^{-1}	19:	1295.10 cm^{-1}
2:	0.00 cm^{-1}	11:	882.33 cm^{-1}	20:	1320.29 cm^{-1}
3:	0.00 cm^{-1}	12:	933.45 cm^{-1}	21:	1547.70 cm^{-1}
4:	0.00 cm^{-1}	13:	1014.99 cm^{-1}	22:	1657.83 cm^{-1}
5:	0.00 cm^{-1}	14:	1018.28 cm^{-1}	23:	3222.44 cm^{-1}
6:	135.18 cm^{-1}	15:	1077.48 cm^{-1}	24:	3226.10 cm^{-1}
7:	319.35 cm^{-1}	16:	1081.85 cm^{-1}	25:	3275.21 cm^{-1}
8:	383.34 cm^{-1}	17:	1130.81 cm^{-1}	26:	3296.94 cm^{-1}

$[C_4H_4N]^+$ – PBE0-D3BJ/def2-TZVP (T_1)

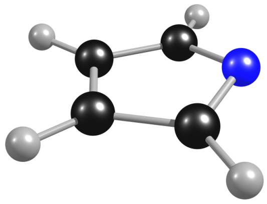

N	-0.243429000	0.635459000	0.984770000
C	-0.030781000	-0.700617000	1.021612000
C	0.029171000	-1.209719000	-0.300255000
C	0.030159000	-0.071217000	-1.149737000
C	-0.029296000	1.051014000	-0.285330000
H	0.010561000	2.103561000	-0.537159000
H	0.005002000	-0.058718000	-2.231432000
H	0.003044000	-2.250158000	-0.596335000
H	0.007753000	-1.241835000	1.958873000

0:	0.00 cm^{-1}	9:	807.51 cm^{-1}	18:	1250.30 cm^{-1}
1:	0.00 cm^{-1}	10:	857.49 cm^{-1}	19:	1323.07 cm^{-1}
2:	0.00 cm^{-1}	11:	869.93 cm^{-1}	20:	1355.99 cm^{-1}
3:	0.00 cm^{-1}	12:	912.09 cm^{-1}	21:	1411.21 cm^{-1}
4:	0.00 cm^{-1}	13:	951.64 cm^{-1}	22:	1419.52 cm^{-1}
5:	0.00 cm^{-1}	14:	1020.74 cm^{-1}	23:	3241.92 cm^{-1}
6:	370.98 cm^{-1}	15:	1042.01 cm^{-1}	24:	3245.30 cm^{-1}
7:	387.41 cm^{-1}	16:	1105.46 cm^{-1}	25:	3250.22 cm^{-1}
8:	667.16 cm^{-1}	17:	1174.88 cm^{-1}	26:	3262.01 cm^{-1}

$[C_4H_4N]^+$ – PBE0-D3BJ/def2-TZVP/CPCM(THF) (T_1)

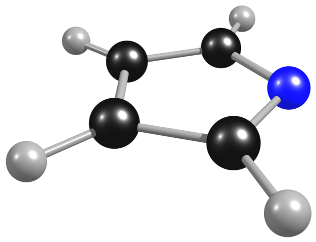

N	0.008955000	0.650695000	1.025147000
C	0.008955000	-0.700861000	1.025928000
C	0.008955000	-1.218705000	-0.292065000
C	0.008956000	-0.065648000	-1.146683000
C	0.008955000	1.044403000	-0.267762000
H	0.008955000	2.091703000	-0.538300000
H	0.008955000	-0.050988000	-2.227374000
H	0.008955000	-2.256721000	-0.593232000
H	0.008956000	-1.264810000	1.948951000

0:	0.00 cm^{-1}	9:	803.78 cm^{-1}	18:	1274.89 cm^{-1}
1:	0.00 cm^{-1}	10:	871.77 cm^{-1}	19:	1342.31 cm^{-1}
2:	0.00 cm^{-1}	11:	876.49 cm^{-1}	20:	1409.91 cm^{-1}
3:	0.00 cm^{-1}	12:	918.88 cm^{-1}	21:	1427.88 cm^{-1}
4:	0.00 cm^{-1}	13:	972.59 cm^{-1}	22:	1474.46 cm^{-1}
5:	0.00 cm^{-1}	14:	1016.19 cm^{-1}	23:	3261.24 cm^{-1}
6:	221.22 cm^{-1}	15:	1037.02 cm^{-1}	24:	3261.48 cm^{-1}
7:	362.29 cm^{-1}	16:	1090.52 cm^{-1}	25:	3274.13 cm^{-1}
8:	685.78 cm^{-1}	17:	1182.27 cm^{-1}	26:	3283.98 cm^{-1}

$[C_4H_5N]^-$ – PBE0-D3BJ/def2-TZVP (D_1)

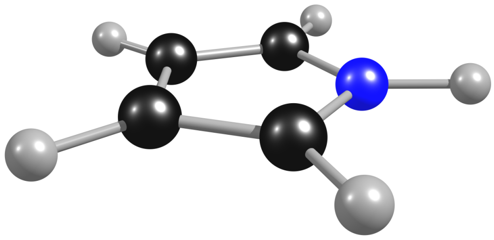

N	-0.132734000	0.598944000	0.982539000
C	-0.079091000	-0.745209000	1.004738000
C	0.010162000	-1.214255000	-0.292777000
C	0.008803000	-0.071098000	-1.130658000
C	-0.081200000	1.024875000	-0.292631000
H	-0.113851000	2.093568000	-0.494488000
H	0.066781000	-0.055186000	-2.215747000
H	0.069460000	-2.253712000	-0.604330000
H	-0.109850000	-1.259525000	1.963098000
H	-0.210340000	1.279531000	1.911335000

0:	0.00 cm^{-1}	10:	714.27 cm^{-1}	20:	1273.03 cm^{-1}
1:	0.00 cm^{-1}	11:	714.58 cm^{-1}	21:	1306.49 cm^{-1}
2:	0.00 cm^{-1}	12:	763.78 cm^{-1}	22:	1425.38 cm^{-1}
3:	0.00 cm^{-1}	13:	774.28 cm^{-1}	23:	1436.54 cm^{-1}
4:	0.00 cm^{-1}	14:	779.94 cm^{-1}	24:	1508.38 cm^{-1}
5:	0.00 cm^{-1}	15:	889.33 cm^{-1}	25:	1523.38 cm^{-1}
6:	308.04 cm^{-1}	16:	993.32 cm^{-1}	26:	3109.56 cm^{-1}
7:	620.73 cm^{-1}	17:	1043.41 cm^{-1}	27:	3112.14 cm^{-1}
8:	654.78 cm^{-1}	18:	1085.49 cm^{-1}	28:	3132.58 cm^{-1}
9:	701.95 cm^{-1}	19:	1155.19 cm^{-1}	29:	3137.51 cm^{-1}

[C_4H_5N]$^-$ – PBE0-D3BJ/def2-TZVP/CPCM(THF) (D_1)

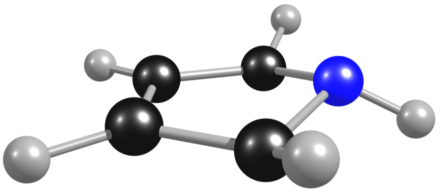

N	-0.267112000	0.649897000	1.052944000
C	0.023119000	-0.759777000	1.085544000
C	0.043477000	-1.179195000	-0.267102000
C	0.040454000	-0.057262000	-1.089485000
C	0.015780000	1.104573000	-0.282107000
H	-0.407668000	2.060405000	-0.592054000
H	0.110033000	-0.059777000	-2.171300000
H	0.115846000	-2.209753000	-0.595656000
H	-0.406771000	-1.343577000	1.900595000
H	0.160985000	1.192396000	1.789701000

0:	0.00 cm^{-1}	10:	652.74 cm^{-1}	20:	1255.00 cm^{-1}
1:	0.00 cm^{-1}	11:	770.61 cm^{-1}	21:	1319.48 cm^{-1}
2:	0.00 cm^{-1}	12:	793.97 cm^{-1}	22:	1384.82 cm^{-1}
3:	0.00 cm^{-1}	13:	844.20 cm^{-1}	23:	1418.48 cm^{-1}
4:	0.00 cm^{-1}	14:	844.92 cm^{-1}	24:	1471.00 cm^{-1}
5:	0.00 cm^{-1}	15:	982.97 cm^{-1}	25:	3079.06 cm^{-1}
6:	361.09 cm^{-1}	16:	998.65 cm^{-1}	26:	3087.44 cm^{-1}
7:	375.50 cm^{-1}	17:	1062.46 cm^{-1}	27:	3188.12 cm^{-1}
8:	550.52 cm^{-1}	18:	1108.32 cm^{-1}	28:	3209.76 cm^{-1}
9:	572.16 cm^{-1}	19:	1123.87 cm^{-1}	29:	3537.47 cm^{-1}

$[C_4H_4N]^-$ – PBE0-D3BJ/def2-TZVP (S_0)

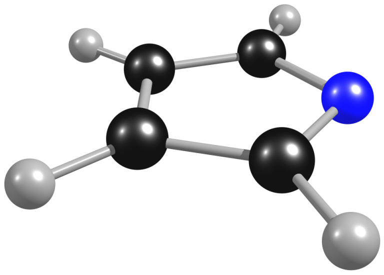

N	0.008956000	0.656049000	1.032558000
C	0.008950000	-0.697199000	1.009311000
C	0.008962000	-1.203519000	-0.291429000
C	0.008949000	-0.069245000	-1.131905000
C	0.008960000	1.027755000	-0.268841000
H	0.008963000	2.079592000	-0.543376000
H	0.008943000	-0.052616000	-2.216199000
H	0.008967000	-2.245695000	-0.591172000
H	0.008947000	-1.266052000	1.935665000

0:	0.00 cm^{-1}	9:	692.43 cm^{-1}	18:	1241.16 cm^{-1}
1:	0.00 cm^{-1}	10:	749.26 cm^{-1}	19:	1330.88 cm^{-1}
2:	0.00 cm^{-1}	11:	756.65 cm^{-1}	20:	1409.41 cm^{-1}
3:	0.00 cm^{-1}	12:	874.89 cm^{-1}	21:	1470.33 cm^{-1}
4:	0.00 cm^{-1}	13:	893.88 cm^{-1}	22:	1491.31 cm^{-1}
5:	0.00 cm^{-1}	14:	1023.83 cm^{-1}	23:	3142.73 cm^{-1}
6:	606.16 cm^{-1}	15:	1037.70 cm^{-1}	24:	3146.83 cm^{-1}
7:	653.40 cm^{-1}	16:	1111.61 cm^{-1}	25:	3175.41 cm^{-1}
8:	686.82 cm^{-1}	17:	1194.40 cm^{-1}	26:	3197.92 cm^{-1}

$[C_4H_4N]^-$ – PBE0-D3BJ/def2-TZVP/CPCM(THF) (S_0)

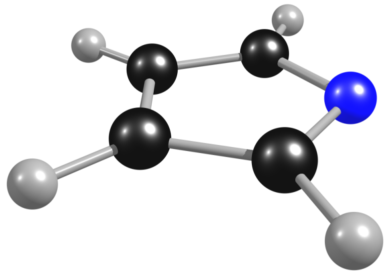

N	0.008960000	0.654228000	1.030126000
C	0.008958000	-0.701928000	1.011949000
C	0.008951000	-1.202989000	-0.289896000
C	0.008960000	-0.067760000	-1.131260000
C	0.008951000	1.031436000	-0.272549000
H	0.008948000	2.081983000	-0.546233000
H	0.008964000	-0.052381000	-2.214526000
H	0.008947000	-2.243910000	-0.590257000
H	0.008959000	-1.269611000	1.937257000

0:	0.00 cm^{-1}	9:	732.85 cm^{-1}	18:	1249.12 cm^{-1}
1:	0.00 cm^{-1}	10:	799.95 cm^{-1}	19:	1332.27 cm^{-1}
2:	0.00 cm^{-1}	11:	828.64 cm^{-1}	20:	1411.30 cm^{-1}
3:	0.00 cm^{-1}	12:	886.38 cm^{-1}	21:	1477.52 cm^{-1}
4:	0.00 cm^{-1}	13:	893.14 cm^{-1}	22:	1487.03 cm^{-1}
5:	0.00 cm^{-1}	14:	1028.12 cm^{-1}	23:	3172.69 cm^{-1}
6:	638.08 cm^{-1}	15:	1044.22 cm^{-1}	24:	3176.28 cm^{-1}
7:	687.74 cm^{-1}	16:	1111.29 cm^{-1}	25:	3202.15 cm^{-1}
8:	711.50 cm^{-1}	17:	1189.53 cm^{-1}	26:	3221.17 cm^{-1}

$[C_4H_4N]^–$ – PBE0-D3BJ/def2-TZVP (T_1)

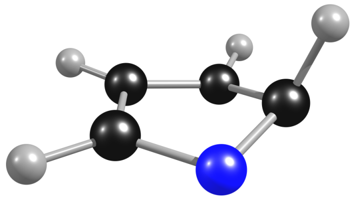

N	-0.242203000	0.703349000	1.078360000
C	-0.147902000	-0.722765000	1.095782000
C	-0.335167000	-1.230228000	-0.312231000
C	0.091349000	-0.121992000	-1.056639000
C	0.088484000	0.996119000	-0.179840000
H	0.249841000	2.029855000	-0.489904000
H	0.317432000	-0.086375000	-2.118970000
H	-0.216789000	-2.267133000	-0.608653000
H	0.803816000	-1.070927000	1.593388000

0:	0.00 cm^{-1}	9:	680.70 cm^{-1}	18:	1154.21 cm^{-1}
1:	0.00 cm^{-1}	10:	713.70 cm^{-1}	19:	1237.94 cm^{-1}
2:	0.00 cm^{-1}	11:	776.28 cm^{-1}	20:	1322.21 cm^{-1}
3:	0.00 cm^{-1}	12:	833.77 cm^{-1}	21:	1398.46 cm^{-1}
4:	0.00 cm^{-1}	13:	872.08 cm^{-1}	22:	1453.29 cm^{-1}
5:	0.00 cm^{-1}	14:	968.56 cm^{-1}	23:	2556.01 cm^{-1}
6:	409.07 cm^{-1}	15:	998.24 cm^{-1}	24:	3098.43 cm^{-1}
7:	445.17 cm^{-1}	16:	1044.16 cm^{-1}	25:	3154.37 cm^{-1}
8:	524.35 cm^{-1}	17:	1082.97 cm^{-1}	26:	3181.98 cm^{-1}

$[C_4H_4N]^-$ – PBE0-D3BJ/def2-TZVP/CPCM(THF) (T_1)

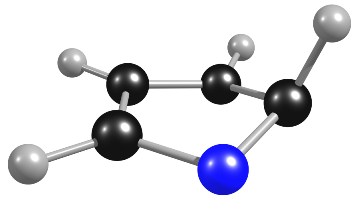

N	-0.250324000	0.700307000	1.079134000
C	-0.140703000	-0.726322000	1.095365000
C	-0.324766000	-1.230851000	-0.312988000
C	0.090342000	-0.118904000	-1.056082000
C	0.086244000	1.000526000	-0.183902000
H	0.249410000	2.031909000	-0.491539000
H	0.317569000	-0.083275000	-2.116799000
H	-0.213228000	-2.266457000	-0.613276000
H	0.794316000	-1.077029000	1.601380000

0:	0.00 cm^{-1}	9:	701.16 cm^{-1}	18:	1147.53 cm^{-1}
1:	0.00 cm^{-1}	10:	766.83 cm^{-1}	19:	1237.50 cm^{-1}
2:	0.00 cm^{-1}	11:	786.66 cm^{-1}	20:	1322.24 cm^{-1}
3:	0.00 cm^{-1}	12:	853.82 cm^{-1}	21:	1400.69 cm^{-1}
4:	0.00 cm^{-1}	13:	864.39 cm^{-1}	22:	1450.23 cm^{-1}
5:	0.00 cm^{-1}	14:	967.75 cm^{-1}	23:	2679.60 cm^{-1}
6:	391.14 cm^{-1}	15:	989.88 cm^{-1}	24:	3142.31 cm^{-1}
7:	426.76 cm^{-1}	16:	1048.06 cm^{-1}	25:	3181.96 cm^{-1}
8:	527.08 cm^{-1}	17:	1088.54 cm^{-1}	26:	3203.35 cm^{-1}

PhF – PBE0-D3BJ/def2-TZVP (S_0)

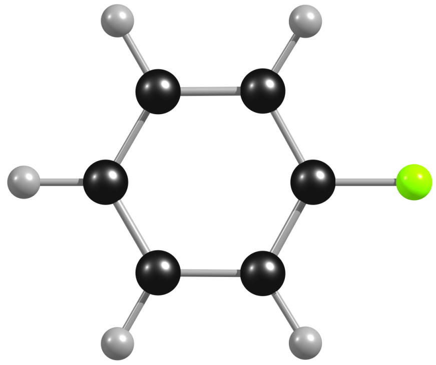

C	0.060346000	-2.791279000	-1.598979000
C	-1.092353000	-3.144930000	-0.913592000
C	1.210106000	-2.438531000	-0.907417000
H	-2.001986000	-3.424025000	-1.430311000
H	2.106818000	-2.163398000	-1.448956000
C	-1.066151000	-3.136886000	0.467248000
C	1.207798000	-2.439241000	0.479900000
H	2.103090000	-2.164549000	1.025069000
C	0.063989000	-2.790158000	1.181402000
H	0.036990000	-2.798443000	2.263774000
H	0.056623000	-2.792418000	-2.682585000
F	-2.175258000	-3.477180000	1.137030000

0:	0.00 cm^{-1}	12:	699.65 cm^{-1}	24:	1274.41 cm^{-1}
1:	0.00 cm^{-1}	13:	771.53 cm^{-1}	25:	1329.02 cm^{-1}
2:	0.00 cm^{-1}	14:	834.82 cm^{-1}	26:	1373.29 cm^{-1}
3:	0.00 cm^{-1}	15:	837.24 cm^{-1}	27:	1498.56 cm^{-1}
4:	0.00 cm^{-1}	16:	915.58 cm^{-1}	28:	1537.71 cm^{-1}
5:	0.00 cm^{-1}	17:	965.18 cm^{-1}	29:	1666.81 cm^{-1}
6:	239.06 cm^{-1}	18:	972.48 cm^{-1}	30:	1671.05 cm^{-1}
7:	411.02 cm^{-1}	19:	1026.77 cm^{-1}	31:	3193.93 cm^{-1}
8:	423.24 cm^{-1}	20:	1051.36 cm^{-1}	32:	3202.73 cm^{-1}
9:	513.35 cm^{-1}	21:	1096.54 cm^{-1}	33:	3215.03 cm^{-1}
10:	526.75 cm^{-1}	22:	1177.39 cm^{-1}	34:	3223.42 cm^{-1}
11:	625.37 cm^{-1}	23:	1179.56 cm^{-1}	35:	3226.09 cm^{-1}

PhF – PBE0-D3BJ/def2-TZVP/CPCM(THF) (S_0)

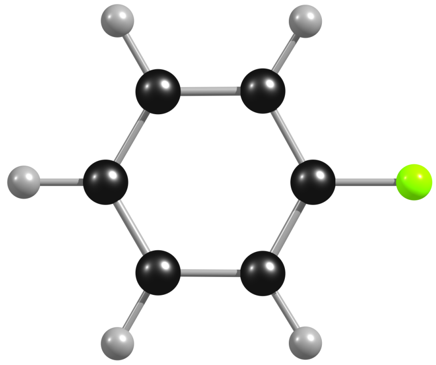

C	0.059753000	-2.791467000	-1.600301000
C	-1.094196000	-3.145495000	-0.914801000
C	1.209910000	-2.438682000	-0.907282000
H	-2.003346000	-3.424539000	-1.433856000
H	2.107002000	-2.163242000	-1.449129000
C	-1.062991000	-3.135903000	0.465341000
C	1.208615000	-2.439107000	0.481112000
H	2.103955000	-2.164268000	1.026862000
C	0.064066000	-2.790159000	1.183707000
H	0.039289000	-2.797692000	2.266788000
H	0.055583000	-2.792655000	-2.684239000
F	-2.177628000	-3.477828000	1.138379000

0:	0.00 cm^{-1}	12:	703.06 cm^{-1}	24:	1248.71 cm^{-1}
1:	0.00 cm^{-1}	13:	773.34 cm^{-1}	25:	1324.22 cm^{-1}
2:	0.00 cm^{-1}	14:	828.21 cm^{-1}	26:	1370.13 cm^{-1}
3:	0.00 cm^{-1}	15:	839.04 cm^{-1}	27:	1491.18 cm^{-1}
4:	0.00 cm^{-1}	16:	920.45 cm^{-1}	28:	1526.35 cm^{-1}
5:	0.00 cm^{-1}	17:	983.89 cm^{-1}	29:	1656.98 cm^{-1}
6:	238.12 cm^{-1}	18:	998.38 cm^{-1}	30:	1670.04 cm^{-1}
7:	409.40 cm^{-1}	19:	1024.15 cm^{-1}	31:	3194.55 cm^{-1}
8:	423.87 cm^{-1}	20:	1046.52 cm^{-1}	32:	3202.30 cm^{-1}
9:	513.34 cm^{-1}	21:	1091.14 cm^{-1}	33:	3214.39 cm^{-1}
10:	525.56 cm^{-1}	22:	1164.88 cm^{-1}	34:	3222.09 cm^{-1}
11:	623.23 cm^{-1}	23:	1166.44 cm^{-1}	35:	3222.75 cm^{-1}

[PhF]$^+$ – PBE0-D3BJ/def2-TZVP (D_1)

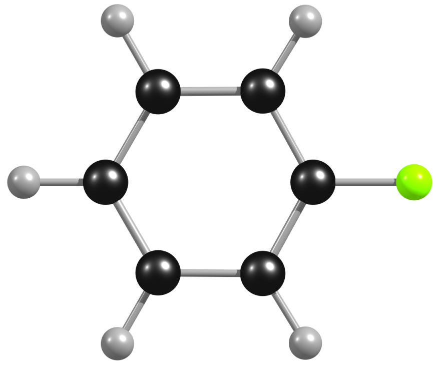

C	0.027854000	-2.801245000	-1.624456000
C	-1.104462000	-3.148634000	-0.954673000
C	1.192560000	-2.443916000	-0.896818000
H	-2.025967000	-3.431365000	-1.449382000
H	2.090417000	-2.168446000	-1.439051000
C	-1.060573000	-3.135175000	0.463880000
C	1.212646000	-2.437748000	0.522072000
H	2.120522000	-2.159208000	1.042554000
C	0.091945000	-2.781567000	1.212910000
H	0.040791000	-2.797258000	2.295030000
H	0.050861000	-2.794189000	-2.707113000
F	-2.126581000	-3.462287000	1.107631000

0:	0.00 cm^{-1}	12:	615.07 cm^{-1}	24:	1310.51 cm^{-1}
1:	0.00 cm^{-1}	13:	789.45 cm^{-1}	25:	1377.06 cm^{-1}
2:	0.00 cm^{-1}	14:	816.86 cm^{-1}	26:	1413.05 cm^{-1}
3:	0.00 cm^{-1}	15:	822.16 cm^{-1}	27:	1424.33 cm^{-1}
4:	0.00 cm^{-1}	16:	961.16 cm^{-1}	28:	1499.52 cm^{-1}
5:	0.00 cm^{-1}	17:	987.40 cm^{-1}	29:	1552.18 cm^{-1}
6:	184.00 cm^{-1}	18:	1003.97 cm^{-1}	30:	1696.53 cm^{-1}
7:	355.89 cm^{-1}	19:	1020.62 cm^{-1}	31:	3214.44 cm^{-1}
8:	405.32 cm^{-1}	20:	1031.37 cm^{-1}	32:	3222.77 cm^{-1}
9:	409.54 cm^{-1}	21:	1095.52 cm^{-1}	33:	3226.66 cm^{-1}
10:	514.98 cm^{-1}	22:	1161.23 cm^{-1}	34:	3233.61 cm^{-1}
11:	523.09 cm^{-1}	23:	1192.38 cm^{-1}	35:	3237.22 cm^{-1}

[PhF]$^+$ – PBE0-D3BJ/def2-TZVP/CPCM(THF) (D_1)

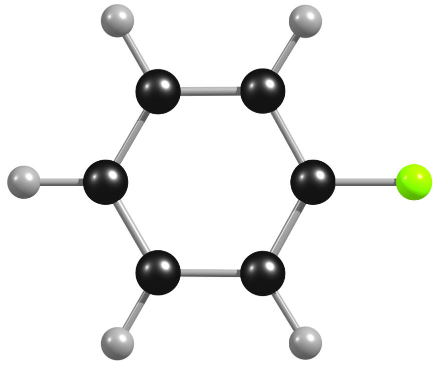

C	0.027743000	-2.801351000	-1.622953000
C	-1.103343000	-3.148382000	-0.953636000
C	1.191399000	-2.444095000	-0.896192000
H	-2.024934000	-3.430688000	-1.447173000
H	2.088723000	-2.169356000	-1.438060000
C	-1.055522000	-3.133853000	0.460989000
C	1.211260000	-2.437851000	0.521437000
H	2.119277000	-2.159384000	1.040438000
C	0.091681000	-2.781695000	1.211567000
H	0.039503000	-2.797768000	2.292947000
H	0.051928000	-2.793882000	-2.704873000
F	-2.127703000	-3.462734000	1.108090000

0:	0.00 cm^{-1}	12:	607.64 cm^{-1}	24:	1305.10 cm^{-1}
1:	0.00 cm^{-1}	13:	777.28 cm^{-1}	25:	1349.71 cm^{-1}
2:	0.00 cm^{-1}	14:	814.26 cm^{-1}	26:	1397.35 cm^{-1}
3:	0.00 cm^{-1}	15:	819.80 cm^{-1}	27:	1427.95 cm^{-1}
4:	0.00 cm^{-1}	16:	955.60 cm^{-1}	28:	1469.76 cm^{-1}
5:	0.00 cm^{-1}	17:	988.59 cm^{-1}	29:	1550.68 cm^{-1}
6:	180.06 cm^{-1}	18:	1006.44 cm^{-1}	30:	1696.98 cm^{-1}
7:	356.87 cm^{-1}	19:	1015.36 cm^{-1}	31:	3220.51 cm^{-1}
8:	399.79 cm^{-1}	20:	1028.79 cm^{-1}	32:	3232.97 cm^{-1}
9:	404.26 cm^{-1}	21:	1089.96 cm^{-1}	33:	3238.35 cm^{-1}
10:	514.26 cm^{-1}	22:	1146.35 cm^{-1}	34:	3242.85 cm^{-1}
11:	517.09 cm^{-1}	23:	1176.09 cm^{-1}	35:	3246.85 cm^{-1}

[PhF]⁻ – PBE0-D3BJ/def2-TZVP (D_1)

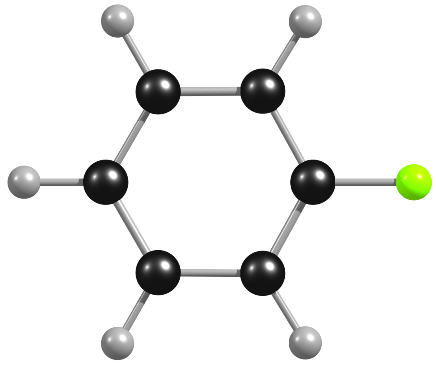

C	0.079412000	-2.785433000	-1.632366000
C	-1.125577000	-3.155128000	-0.913356000
C	1.213635000	-2.437451000	-0.909543000
H	-2.044642000	-3.437129000	-1.411451000
H	2.115896000	-2.160620000	-1.454436000
C	-1.061596000	-3.135486000	0.464487000
C	1.244977000	-2.427842000	0.479328000
H	2.148512000	-2.150615000	1.011498000
C	0.047175000	-2.795308000	1.211367000
H	0.000036000	-2.809777000	2.292974000
H	0.090568000	-2.781979000	-2.716941000
F	-2.198383000	-3.484271000	1.151022000

0:	0.00 cm^{-1}	12:	514.38 cm^{-1}	24:	1169.18 cm^{-1}
1:	0.00 cm^{-1}	13:	529.72 cm^{-1}	25:	1224.65 cm^{-1}
2:	0.00 cm^{-1}	14:	551.10 cm^{-1}	26:	1318.71 cm^{-1}
3:	0.00 cm^{-1}	15:	587.15 cm^{-1}	27:	1372.65 cm^{-1}
4:	0.00 cm^{-1}	16:	642.57 cm^{-1}	28:	1483.95 cm^{-1}
5:	0.00 cm^{-1}	17:	811.04 cm^{-1}	29:	1487.62 cm^{-1}
6:	50.65 cm^{-1}	18:	826.84 cm^{-1}	30:	1545.49 cm^{-1}
7:	207.97 cm^{-1}	19:	930.18 cm^{-1}	31:	3115.00 cm^{-1}
8:	236.38 cm^{-1}	20:	991.50 cm^{-1}	32:	3164.40 cm^{-1}
9:	350.13 cm^{-1}	21:	1031.20 cm^{-1}	33:	3176.59 cm^{-1}
10:	398.19 cm^{-1}	22:	1061.11 cm^{-1}	34:	3201.47 cm^{-1}
11:	418.61 cm^{-1}	23:	1126.58 cm^{-1}	35:	3208.18 cm^{-1}

[PhF]$^{–}$ – PBE0-D3BJ/def2-TZVP/CPCM(THF) (D_1)

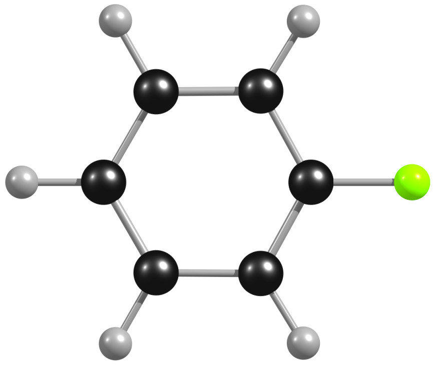

C	0.078764000	-2.784799000	-1.632960000
C	-1.127838000	-3.155264000	-0.913789000
C	1.215156000	-2.437169000	-0.910465000
H	-2.045021000	-3.438261000	-1.415161000
H	2.116343000	-2.160888000	-1.454816000
C	-1.061625000	-3.135349000	0.464516000
C	1.245330000	-2.427973000	0.480070000
H	2.147669000	-2.151635000	1.013628000
C	0.046390000	-2.795343000	1.213481000
H	0.002562000	-2.807938000	2.295459000
H	0.088300000	-2.782166000	-2.717046000
F	-2.196017000	-3.484252000	1.149667000

0:	0.00 cm^{-1}	12:	519.95 cm^{-1}	24:	1161.42 cm^{-1}
1:	0.00 cm^{-1}	13:	546.66 cm^{-1}	25:	1219.63 cm^{-1}
2:	0.00 cm^{-1}	14:	553.25 cm^{-1}	26:	1315.08 cm^{-1}
3:	0.00 cm^{-1}	15:	606.80 cm^{-1}	27:	1375.53 cm^{-1}
4:	0.00 cm^{-1}	16:	658.16 cm^{-1}	28:	1470.41 cm^{-1}
5:	0.00 cm^{-1}	17:	811.42 cm^{-1}	29:	1480.39 cm^{-1}
6:	214.46 cm^{-1}	18:	900.11 cm^{-1}	30:	1538.10 cm^{-1}
7:	239.03 cm^{-1}	19:	918.33 cm^{-1}	31:	3141.66 cm^{-1}
8:	363.74 cm^{-1}	20:	990.10 cm^{-1}	32:	3181.24 cm^{-1}
9:	399.42 cm^{-1}	21:	1028.80 cm^{-1}	33:	3190.28 cm^{-1}
10:	490.91 cm^{-1}	22:	1045.81 cm^{-1}	34:	3211.80 cm^{-1}
11:	515.75 cm^{-1}	23:	1123.25 cm^{-1}	35:	3213.84 cm^{-1}

$PhCH_3$ – PBE0-D3BJ/def2-TZVP (S_0)

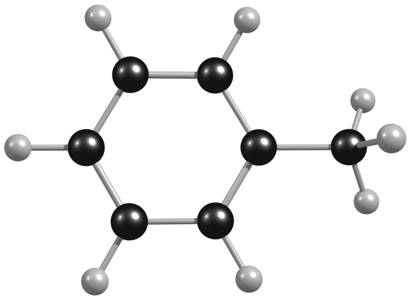

H	2.905048000	-0.974466000	-1.964903000
C	5.059683000	-0.836697000	-0.320857000
H	5.237596000	-1.377796000	-1.251885000
H	5.680376000	-1.283286000	0.457939000
H	5.408079000	0.190348000	-0.467905000
C	3.609681000	-0.860038000	0.055976000
C	3.216776000	-0.799188000	1.389787000
C	1.875386000	-0.790847000	1.739987000
C	0.898377000	-0.845665000	0.757455000
C	1.275725000	-0.910932000	-0.575340000
C	2.618644000	-0.918956000	-0.919523000
H	3.974851000	-0.760224000	2.165489000
H	1.592322000	-0.745287000	2.785512000
H	-0.150736000	-0.842351000	1.029008000
H	0.520774000	-0.959914000	-1.351851000

0:	0.00 cm^{-1}	13:	712.04 cm^{-1}	26:	1202.31 cm^{-1}
1:	0.00 cm^{-1}	14:	742.84 cm^{-1}	27:	1248.65 cm^{-1}
2:	0.00 cm^{-1}	15:	808.57 cm^{-1}	28:	1347.90 cm^{-1}
3:	0.00 cm^{-1}	16:	856.91 cm^{-1}	29:	1368.42 cm^{-1}
4:	0.00 cm^{-1}	17:	909.72 cm^{-1}	30:	1406.36 cm^{-1}
5:	0.00 cm^{-1}	18:	961.91 cm^{-1}	31:	1467.74 cm^{-1}
6:	-25.11 cm^{-1} *	19:	980.34 cm^{-1}	32:	1480.03 cm^{-1}
7:	207.83 cm^{-1}	20:	1002.04 cm^{-1}	33:	1508.27 cm^{-1}
8:	339.87 cm^{-1}	21:	1022.33 cm^{-1}	34:	1538.96 cm^{-1}
9:	410.86 cm^{-1}	22:	1058.65 cm^{-1}	35:	1653.25 cm^{-1}
10:	472.53 cm^{-1}	23:	1060.74 cm^{-1}	36:	1676.01 cm^{-1}
11:	529.18 cm^{-1}	24:	1114.16 cm^{-1}	37:	3048.85 cm^{-1}
12:	630.69 cm^{-1}	25:	1176.47 cm^{-1}	38:	3109.35 cm^{-1}

39:	3135.87 cm^{-1}	41:	3178.83 cm^{-1}	43:	3201.72 cm^{-1}
40:	3174.44 cm^{-1}	42:	3190.36 cm^{-1}	44:	3212.42 cm^{-1}

* One negative frequency < 50 cm^{-1} due to methyl group rotation could not be removed by very tight convergence criteria. The structure was used without further optimization as the energy difference to the real local minimum was expected to be negligible.

$PhCH_3$ – PBE0-D3BJ/def2-TZVP/CPCM(THF) (S_0)

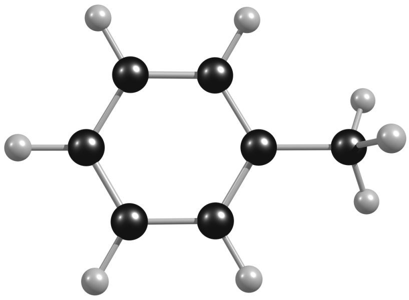

H	2.905265000	-0.849514000	-1.969186000
C	5.060737000	-0.841866000	-0.319729000
H	5.235845000	-1.371598000	-1.258448000
H	5.679339000	-1.298036000	0.455583000
H	5.413059000	0.185900000	-0.456407000
C	3.611493000	-0.862706000	0.056318000
C	3.218167000	-0.871506000	1.392910000
C	1.875171000	-0.861771000	1.743380000
C	0.897064000	-0.844815000	0.758881000
C	1.274991000	-0.839780000	-0.577046000
C	2.618919000	-0.849551000	-0.922005000
H	3.976533000	-0.888830000	2.169486000
H	1.592191000	-0.871065000	2.790437000
H	-0.152605000	-0.840066000	1.030416000
H	0.519527000	-0.831770000	-1.355337000

0:	0.00 cm^{-1}	13:	719.40 cm^{-1}	26:	1189.55 cm^{-1}
1:	0.00 cm^{-1}	14:	749.95 cm^{-1}	27:	1245.67 cm^{-1}
2:	0.00 cm^{-1}	15:	807.87 cm^{-1}	28:	1341.10 cm^{-1}
3:	0.00 cm^{-1}	16:	866.65 cm^{-1}	29:	1363.37 cm^{-1}
4:	0.00 cm^{-1}	17:	922.07 cm^{-1}	30:	1397.89 cm^{-1}
5:	0.00 cm^{-1}	18:	988.65 cm^{-1}	31:	1450.16 cm^{-1}
6:	20.39 cm^{-1}	19:	1000.42 cm^{-1}	32:	1457.79 cm^{-1}
7:	210.24 cm^{-1}	20:	1007.53 cm^{-1}	33:	1494.06 cm^{-1}
8:	342.17 cm^{-1}	21:	1020.68 cm^{-1}	34:	1534.16 cm^{-1}
9:	416.15 cm^{-1}	22:	1058.28 cm^{-1}	35:	1648.13 cm^{-1}
10:	477.14 cm^{-1}	23:	1061.81 cm^{-1}	36:	1669.39 cm^{-1}
11:	527.80 cm^{-1}	24:	1109.36 cm^{-1}	37:	3047.00 cm^{-1}
12:	632.68 cm^{-1}	25:	1164.09 cm^{-1}	38:	3109.47 cm^{-1}

39: 3137.48 cm^{-1}

40: 3177.49 cm^{-1}

41: 3178.22 cm^{-1}

42: 3190.81 cm^{-1}

43: 3199.78 cm^{-1}

44: 3211.67 cm^{-1}

$[PhCH_3]^+$ – PBE0-D3BJ/def2-TZVP (D_1)

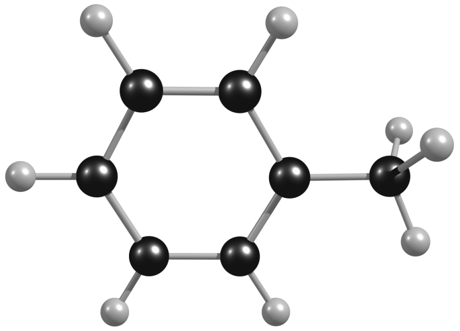

H	2.907494000	-0.953657000	-2.000121000
C	5.030663000	-0.871028000	-0.318463000
H	5.215360000	-1.343946000	-1.283603000
H	5.667464000	-1.295666000	0.458758000
H	5.342523000	0.183959000	-0.426955000
C	3.613707000	-0.877918000	0.050490000
C	3.225179000	-0.819252000	1.426254000
C	1.908079000	-0.804825000	1.768150000
C	0.924855000	-0.842864000	0.750720000
C	1.286241000	-0.897298000	-0.616901000
C	2.602799000	-0.911819000	-0.960972000
H	3.998802000	-0.791296000	2.184669000
H	1.601994000	-0.765230000	2.806108000
H	-0.124636000	-0.827998000	1.023740000
H	0.512053000	-0.927336000	-1.373445000

0:	0.00 cm^{-1}	13:	578.44 cm^{-1}	26:	1216.54 cm^{-1}
1:	0.00 cm^{-1}	14:	743.04 cm^{-1}	27:	1274.34 cm^{-1}
2:	0.00 cm^{-1}	15:	789.81 cm^{-1}	28:	1307.33 cm^{-1}
3:	0.00 cm^{-1}	16:	803.33 cm^{-1}	29:	1343.89 cm^{-1}
4:	0.00 cm^{-1}	17:	938.41 cm^{-1}	30:	1391.73 cm^{-1}
5:	0.00 cm^{-1}	18:	982.98 cm^{-1}	31:	1413.63 cm^{-1}
6:	-48.56 cm^{-1} *	19:	995.81 cm^{-1}	32:	1432.67 cm^{-1}
7:	142.01 cm^{-1}	20:	999.55 cm^{-1}	33:	1472.09 cm^{-1}
8:	343.26 cm^{-1}	21:	1009.76 cm^{-1}	34:	1480.71 cm^{-1}
9:	350.79 cm^{-1}	22:	1023.05 cm^{-1}	35:	1550.70 cm^{-1}
10:	380.47 cm^{-1}	23:	1041.49 cm^{-1}	36:	1701.31 cm^{-1}
11:	500.70 cm^{-1}	24:	1100.39 cm^{-1}	37:	2967.86 cm^{-1}
12:	512.99 cm^{-1}	25:	1167.72 cm^{-1}	38:	3100.78 cm^{-1}

39:	3165.38 cm^{-1}	41:	3213.84 cm^{-1}	43:	3230.70 cm^{-1}
40:	3211.92 cm^{-1}	42:	3218.34 cm^{-1}	44:	3234.75 cm^{-1}

* One negative frequency < 50 cm^{-1} due to methyl group rotation could not be removed by very tight convergence criteria. The structure was used without further optimization as the energy difference to the real local minimum was expected to be negligible.

[PhCH$_3$]$^+$ – PBE0-D3BJ/def2-TZVP/CPCM(THF) (D$_1$)

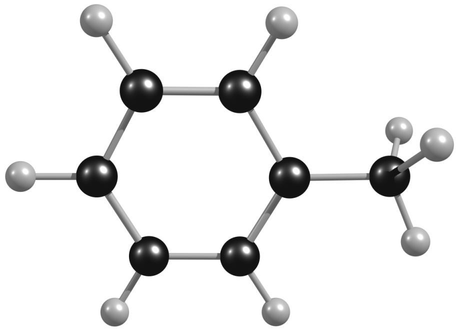

H	2.939926000	-0.687756000	-1.973788000
C	5.032121000	-0.840074000	-0.298434000
H	5.233890000	-1.722397000	-0.925149000
H	5.703225000	-0.838768000	0.557275000
H	5.242275000	0.012803000	-0.957024000
C	3.620970000	-0.842336000	0.080515000
C	3.223571000	-0.935585000	1.444591000
C	1.902493000	-0.941198000	1.770128000
C	0.927231000	-0.854663000	0.745728000
C	1.298731000	-0.762536000	-0.611559000
C	2.618274000	-0.756482000	-0.941211000
H	3.991172000	-0.999668000	2.206162000
H	1.582651000	-1.009765000	2.802229000
H	-0.123088000	-0.860583000	1.013044000
H	0.532253000	-0.697964000	-1.373252000

0:	0.00 cm^{-1}	13:	575.03 cm^{-1}	26:	1201.07 cm^{-1}
1:	0.00 cm^{-1}	14:	744.51 cm^{-1}	27:	1271.55 cm^{-1}
2:	0.00 cm^{-1}	15:	787.86 cm^{-1}	28:	1284.91 cm^{-1}
3:	0.00 cm^{-1}	16:	801.87 cm^{-1}	29:	1335.17 cm^{-1}
4:	0.00 cm^{-1}	17:	942.45 cm^{-1}	30:	1391.53 cm^{-1}
5:	0.00 cm^{-1}	18:	974.17 cm^{-1}	31:	1398.07 cm^{-1}
6:	44.59 cm^{-1}	19:	1000.62 cm^{-1}	32:	1416.72 cm^{-1}
7:	148.98 cm^{-1}	20:	1001.52 cm^{-1}	33:	1450.34 cm^{-1}
8:	345.78 cm^{-1}	21:	1012.84 cm^{-1}	34:	1477.91 cm^{-1}
9:	350.76 cm^{-1}	22:	1021.56 cm^{-1}	35:	1547.69 cm^{-1}
10:	383.72 cm^{-1}	23:	1040.42 cm^{-1}	36:	1699.74 cm^{-1}
11:	498.63 cm^{-1}	24:	1099.60 cm^{-1}	37:	3007.41 cm^{-1}
12:	521.07 cm^{-1}	25:	1151.80 cm^{-1}	38:	3056.74 cm^{-1}

39:	3182.15 cm^{-1}	41:	3220.85 cm^{-1}	43:	3234.75 cm^{-1}
40:	3218.19 cm^{-1}	42:	3227.94 cm^{-1}	44:	3241.36 cm^{-1}

[PhCH$_3$]$^-$ – PBE0-D3BJ/def2-TZVP (D$_1$)

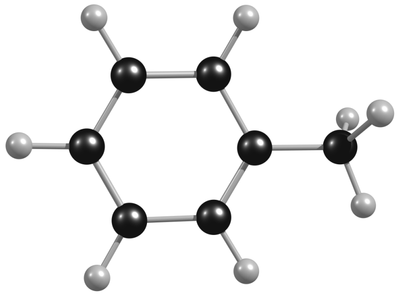

H	2.921080000	-0.639603000	-1.992419000
C	5.062574000	-0.845211000	-0.306957000
H	5.320608000	-1.705210000	-0.946793000
H	5.711661000	-0.869940000	0.574031000
H	5.317347000	0.052307000	-0.893699000
C	3.619209000	-0.860206000	0.075243000
C	3.230958000	-0.933121000	1.389414000
C	1.864189000	-0.974147000	1.780235000
C	0.884984000	-0.857385000	0.746726000
C	1.260035000	-0.783002000	-0.570863000
C	2.627513000	-0.828449000	-0.963959000
H	4.006798000	-0.981595000	2.154409000
H	1.582833000	-0.961283000	2.827383000
H	-0.172872000	-0.845218000	1.002534000
H	0.488777000	-0.704908000	-1.336031000

0:	0.00 cm^{-1}	13:	503.72 cm^{-1}	26:	1156.47 cm^{-1}
1:	0.00 cm^{-1}	14:	598.11 cm^{-1}	27:	1215.09 cm^{-1}
2:	0.00 cm^{-1}	15:	625.29 cm^{-1}	28:	1283.73 cm^{-1}
3:	0.00 cm^{-1}	16:	686.49 cm^{-1}	29:	1320.36 cm^{-1}
4:	0.00 cm^{-1}	17:	751.25 cm^{-1}	30:	1376.32 cm^{-1}
5:	0.00 cm^{-1}	18:	804.66 cm^{-1}	31:	1390.61 cm^{-1}
6:	147.43 cm^{-1}	19:	830.65 cm^{-1}	32:	1419.38 cm^{-1}
7:	188.28 cm^{-1}	20:	864.84 cm^{-1}	33:	1436.35 cm^{-1}
8:	305.81 cm^{-1}	21:	946.12 cm^{-1}	34:	1478.67 cm^{-1}
9:	335.15 cm^{-1}	22:	992.41 cm^{-1}	35:	1540.19 cm^{-1}
10:	392.98 cm^{-1}	23:	1022.07 cm^{-1}	36:	1650.20 cm^{-1}
11:	421.55 cm^{-1}	24:	1038.34 cm^{-1}	37:	2933.73 cm^{-1}
12:	471.42 cm^{-1}	25:	1063.91 cm^{-1}	38:	2944.53 cm^{-1}

39:	3076.27 cm^{-1}	41:	3108.98 cm^{-1}	43:	3160.28 cm^{-1}
40:	3105.26 cm^{-1}	42:	3136.52 cm^{-1}	44:	3179.93 cm^{-1}

[$PhCH_3$]$^-$ – PBE0-D3BJ/def2-TZVP/CPCM(THF) (D_1)

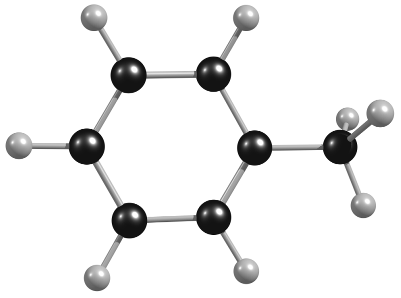

H	2.943132000	-0.966467000	-1.976768000
C	5.062091000	-0.843783000	-0.332029000
H	5.205735000	-0.909553000	-1.413588000
H	5.594814000	-1.683836000	0.132554000
H	5.562671000	0.067122000	0.021655000
C	3.609876000	-0.847232000	0.035247000
C	3.247441000	-0.778474000	1.415167000
C	1.851977000	-0.786779000	1.752337000
C	0.889612000	-0.854413000	0.773510000
C	1.238409000	-0.917214000	-0.601707000
C	2.637560000	-0.914777000	-0.933337000
H	4.012577000	-0.732238000	2.183154000
H	1.549870000	-0.737593000	2.795862000
H	-0.159960000	-0.857791000	1.061121000
H	0.479889000	-0.973944000	-1.373923000

0:	0.00 cm^{-1}	13:	510.36 cm^{-1}	26:	1161.85 cm^{-1}
1:	0.00 cm^{-1}	14:	576.13 cm^{-1}	27:	1210.50 cm^{-1}
2:	0.00 cm^{-1}	15:	643.82 cm^{-1}	28:	1256.06 cm^{-1}
3:	0.00 cm^{-1}	16:	690.41 cm^{-1}	29:	1319.93 cm^{-1}
4:	0.00 cm^{-1}	17:	733.23 cm^{-1}	30:	1377.30 cm^{-1}
5:	0.00 cm^{-1}	18:	780.11 cm^{-1}	31:	1389.34 cm^{-1}
6:	75.43 cm^{-1}	19:	802.09 cm^{-1}	32:	1423.72 cm^{-1}
7:	148.31 cm^{-1}	20:	890.85 cm^{-1}	33:	1438.43 cm^{-1}
8:	272.78 cm^{-1}	21:	936.87 cm^{-1}	34:	1463.60 cm^{-1}
9:	316.02 cm^{-1}	22:	989.07 cm^{-1}	35:	1532.66 cm^{-1}
10:	338.95 cm^{-1}	23:	1030.58 cm^{-1}	36:	1647.21 cm^{-1}
11:	382.62 cm^{-1}	24:	1042.33 cm^{-1}	37:	2998.09 cm^{-1}
12:	501.62 cm^{-1}	25:	1058.18 cm^{-1}	38:	3036.67 cm^{-1}

39:	3105.55 cm^{-1}	41:	3137.12 cm^{-1}	43:	3179.98 cm^{-1}
40:	3132.42 cm^{-1}	42:	3154.88 cm^{-1}	44:	3192.57 cm^{-1}

SF_6 – PBE0-D3BJ/def2-TZVP (S_0)

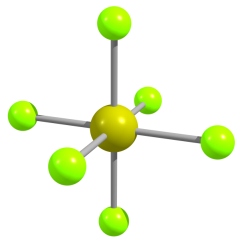

F	0.183866000	-0.344804000	-2.473133000
S	0.183866000	-0.344804000	-0.907964000
F	0.183866000	-0.344804000	0.657204000
F	0.183866000	1.220365000	-0.907964000
F	0.183866000	-1.909972000	-0.907964000
F	-1.381303000	-0.344803000	-0.907965000
F	1.749035000	-0.344804000	-0.907965000

0:	0.00 cm^{-1}	7:	343.69 cm^{-1}	14:	610.52 cm^{-1}
1:	0.00 cm^{-1}	8:	343.69 cm^{-1}	15:	647.24 cm^{-1}
2:	0.00 cm^{-1}	9:	518.54 cm^{-1}	16:	647.26 cm^{-1}
3:	0.00 cm^{-1}	10:	518.56 cm^{-1}	17:	779.65 cm^{-1}
4:	0.00 cm^{-1}	11:	518.57 cm^{-1}	18:	961.22 cm^{-1}
5:	0.00 cm^{-1}	12:	610.48 cm^{-1}	19:	961.23 cm^{-1}
6:	343.67 cm^{-1}	13:	610.50 cm^{-1}	20:	961.24 cm^{-1}

SF_6 – PBE0-D3BJ/def2-TZVP/CPCM(THF) (S_0)

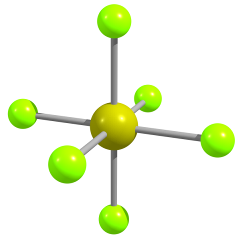

F	0.183866000	-0.344803000	-2.472969000
S	0.183866000	-0.344804000	-0.907964000
F	0.183866000	-0.344803000	0.657040000
F	0.183866000	1.220201000	-0.907964000
F	0.183866000	-1.909808000	-0.907964000
F	-1.381138000	-0.344804000	-0.907964000
F	1.748870000	-0.344804000	-0.907964000

0:	0.00 cm^{-1}	7:	343.37 cm^{-1}	14:	605.29 cm^{-1}
1:	0.00 cm^{-1}	8:	343.39 cm^{-1}	15:	638.90 cm^{-1}
2:	0.00 cm^{-1}	9:	518.03 cm^{-1}	16:	638.91 cm^{-1}
3:	0.00 cm^{-1}	10:	518.04 cm^{-1}	17:	779.97 cm^{-1}
4:	0.00 cm^{-1}	11:	518.06 cm^{-1}	18:	919.85 cm^{-1}
5:	0.00 cm^{-1}	12:	605.25 cm^{-1}	19:	919.85 cm^{-1}
6:	343.36 cm^{-1}	13:	605.28 cm^{-1}	20:	919.86 cm^{-1}

[SF_5]$^•$ – PBE0-D3BJ/def2-TZVP (D_1)

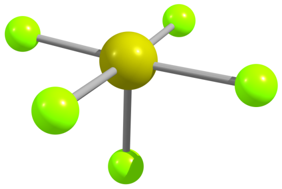

F	0.183866000	-0.340471000	-2.509959000
S	0.183866000	-0.386455000	-0.907964000
F	0.183866000	-0.340471000	0.694030000
F	0.183866000	-1.930444000	-0.907964000
F	-1.418128000	-0.340471000	-0.907964000
F	1.785861000	-0.340471000	-0.907964000

0:	0.00 cm^{-1}	6:	237.30 cm^{-1}	12:	551.52 cm^{-1}
1:	0.00 cm^{-1}	7:	356.64 cm^{-1}	13:	589.76 cm^{-1}
2:	0.00 cm^{-1}	8:	356.67 cm^{-1}	14:	642.72 cm^{-1}
3:	0.00 cm^{-1}	9:	461.10 cm^{-1}	15:	838.60 cm^{-1}
4:	0.00 cm^{-1}	10:	523.18 cm^{-1}	16:	838.61 cm^{-1}
5:	0.00 cm^{-1}	11:	523.20 cm^{-1}	17:	904.05 cm^{-1}

[SF_5]$^•$ – PBE0-D3BJ/def2-TZVP/CPCM(THF) (D_1)

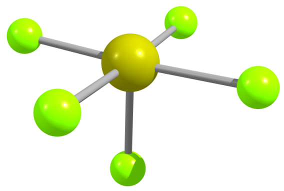

F	0.183866000	-0.341125000	-2.511122000
S	0.183866000	-0.385760000	-0.907964000
F	0.183866000	-0.341125000	0.695194000
F	0.183866000	-1.928524000	-0.907964000
F	-1.419292000	-0.341124000	-0.907964000
F	1.787024000	-0.341124000	-0.907964000

0:	0.00 cm^{-1}	6:	237.61 cm^{-1}	12:	544.28 cm^{-1}
1:	0.00 cm^{-1}	7:	355.05 cm^{-1}	13:	578.08 cm^{-1}
2:	0.00 cm^{-1}	8:	355.06 cm^{-1}	14:	635.86 cm^{-1}
3:	0.00 cm^{-1}	9:	458.59 cm^{-1}	15:	780.91 cm^{-1}
4:	0.00 cm^{-1}	10:	522.10 cm^{-1}	16:	780.91 cm^{-1}
5:	0.00 cm^{-1}	11:	522.11 cm^{-1}	17:	887.37 cm^{-1}

$[SF_6]^+$ – PBE0-D3BJ/def2-TZVP (D_1)

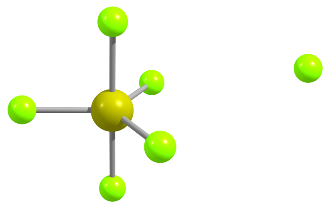

F	-0.119277000	-0.368179000	-2.358927000
S	-0.322179000	-0.370902000	-0.843295000
F	-0.512135000	-0.373492000	0.674975000
F	0.387430000	0.943306000	-0.747731000
F	0.412599000	-1.671291000	-0.749048000
F	-1.805795000	-0.385107000	-1.034977000
F	3.002449000	-0.338776000	-1.212881000

0:	0.00 cm^{-1}	7:	68.00 cm^{-1}	14:	577.74 cm^{-1}
1:	0.00 cm^{-1}	8:	79.83 cm^{-1}	15:	632.12 cm^{-1}
2:	0.00 cm^{-1}	9:	180.16 cm^{-1}	16:	725.53 cm^{-1}
3:	0.00 cm^{-1}	10:	195.91 cm^{-1}	17:	845.93 cm^{-1}
4:	0.00 cm^{-1}	11:	561.01 cm^{-1}	18:	1036.94 cm^{-1}
5:	0.00 cm^{-1}	12:	569.24 cm^{-1}	19:	1090.50 cm^{-1}
6:	24.11 cm^{-1}	13:	572.57 cm^{-1}	20:	1094.31 cm^{-1}

$[SF_6]^+$ – PBE0-D3BJ/def2-TZVP/CPCM(THF) (D_1)

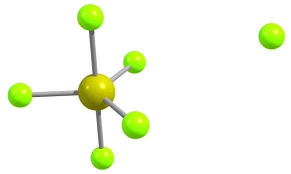

F	-0.086535000	-0.367834000	-2.339282000
S	-0.335379000	-0.371376000	-0.829405000
F	-0.579206000	-0.374877000	0.681528000
F	0.395426000	0.925551000	-0.706452000
F	0.396442000	-1.668286000	-0.712382000
F	-1.808979000	-0.371386000	-1.069517000
F	3.061322000	-0.336231000	-1.296374000

0:	0.00 cm^{-1}	7:	34.19 cm^{-1}	14:	574.43 cm^{-1}
1:	0.00 cm^{-1}	8:	64.03 cm^{-1}	15:	626.85 cm^{-1}
2:	0.00 cm^{-1}	9:	182.98 cm^{-1}	16:	714.95 cm^{-1}
3:	0.00 cm^{-1}	10:	191.21 cm^{-1}	17:	851.65 cm^{-1}
4:	0.00 cm^{-1}	11:	565.06 cm^{-1}	18:	999.44 cm^{-1}
5:	0.00 cm^{-1}	12:	567.35 cm^{-1}	19:	1079.41 cm^{-1}
6:	32.59 cm^{-1}	13:	572.87 cm^{-1}	20:	1080.00 cm^{-1}

$[SF_5]^+$ – PBE0-D3BJ/def2-TZVP (S_0)

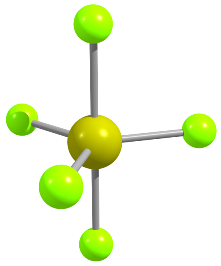

F	0.163121000	0.178890000	-2.182489000
S	0.180097000	-0.593564000	-0.900804000
F	0.169853000	0.129886000	0.409213000
F	0.207244000	-2.089600000	-0.929277000
F	-1.349288000	-0.621409000	-0.897343000
F	1.709451000	-0.565699000	-0.904239000

0:	0.00 cm^{-1}	6:	182.05 cm^{-1}	12:	632.05 cm^{-1}
1:	0.00 cm^{-1}	7:	182.90 cm^{-1}	13:	726.90 cm^{-1}
2:	0.00 cm^{-1}	8:	565.52 cm^{-1}	14:	846.56 cm^{-1}
3:	0.00 cm^{-1}	9:	565.62 cm^{-1}	15:	1036.99 cm^{-1}
4:	0.00 cm^{-1}	10:	575.93 cm^{-1}	16:	1093.38 cm^{-1}
5:	0.00 cm^{-1}	11:	576.07 cm^{-1}	17:	1093.96 cm^{-1}

$[SF_5]^+$ – PBE0-D3BJ/def2-TZVP/CPCM(THF) (S_0)

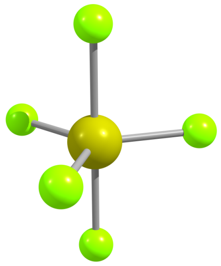

F	0.163117000	0.177624000	-2.179821000
S	0.180123000	-0.593609000	-0.900889000
F	0.169859000	0.128298000	0.406575000
F	0.207191000	-2.086618000	-0.928812000
F	-1.350205000	-0.621454000	-0.897552000
F	1.710393000	-0.565736000	-0.904439000

0:	0.00 cm^{-1}	7:	185.82 cm^{-1}	14:	851.83 cm^{-1}
1:	0.00 cm^{-1}	8:	565.79 cm^{-1}	15:	1000.87 cm^{-1}
2:	0.00 cm^{-1}	9:	566.02 cm^{-1}	16:	1079.43 cm^{-1}
3:	0.00 cm^{-1}	10:	573.05 cm^{-1}	17:	1079.92 cm^{-1}
4:	0.00 cm^{-1}	11:	573.84 cm^{-1}		
5:	0.00 cm^{-1}	12:	626.63 cm^{-1}		
6:	182.30 cm^{-1}	13:	714.21 cm^{-1}		

$[SF_5]^+$ – PBE0-D3BJ/def2-TZVP (T_1)

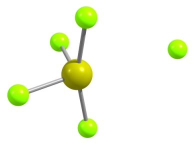

F	0.174288000	-0.286208000	-3.218213000
S	0.185253000	-0.472694000	-0.575127000
F	0.191235000	-0.364407000	0.917551000
F	0.184202000	-1.948516000	-0.813446000
F	-1.356495000	-0.293596000	-0.735055000
F	1.725694000	-0.293817000	-0.747415000

0:	0.00 cm^{-1}	6:	73.66 cm^{-1}	12:	512.98 cm^{-1}
1:	0.00 cm^{-1}	7:	76.30 cm^{-1}	13:	517.47 cm^{-1}
2:	0.00 cm^{-1}	8:	112.35 cm^{-1}	14:	678.42 cm^{-1}
3:	0.00 cm^{-1}	9:	270.11 cm^{-1}	15:	857.75 cm^{-1}
4:	0.00 cm^{-1}	10:	327.01 cm^{-1}	16:	980.49 cm^{-1}
5:	0.00 cm^{-1}	11:	421.83 cm^{-1}	17:	1036.63 cm^{-1}

$[SF_5]^+$ – PBE0-D3BJ/def2-TZVP/CPCM(THF) (T_1)

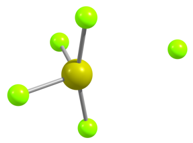

F	0.174471000	-0.290267000	-3.235330000
S	0.185210000	-0.472761000	-0.568512000
F	0.191306000	-0.369139000	0.921621000
F	0.184134000	-1.944474000	-0.815622000
F	-1.354882000	-0.291223000	-0.730580000
F	1.723937000	-0.291373000	-0.743282000

0:	0.00 cm^{-1}	6:	68.87 cm^{-1}	12:	510.02 cm^{-1}
1:	0.00 cm^{-1}	7:	74.71 cm^{-1}	13:	517.98 cm^{-1}
2:	0.00 cm^{-1}	8:	101.27 cm^{-1}	14:	675.95 cm^{-1}
3:	0.00 cm^{-1}	9:	269.17 cm^{-1}	15:	825.05 cm^{-1}
4:	0.00 cm^{-1}	10:	322.00 cm^{-1}	16:	974.46 cm^{-1}
5:	0.00 cm^{-1}	11:	422.18 cm^{-1}	17:	1026.50 cm^{-1}

[SF_6]$^-$ – PBE0-D3BJ/def2-TZVP (D_1)

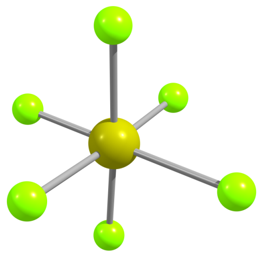

F	0.166480000	-0.347503000	-2.755602000
S	0.169581000	-0.347141000	-0.900227000
F	0.172246000	-0.346811000	0.731307000
F	0.169663000	1.352831000	-0.842547000
F	0.169665000	-2.047115000	-0.841851000
F	-1.530263000	-0.347126000	-0.839424000
F	1.869677000	-0.347127000	-0.844975000

0:	0.00 cm^{-1}	7:	103.72 cm^{-1}	14:	394.08 cm^{-1}
1:	0.00 cm^{-1}	8:	153.82 cm^{-1}	15:	457.83 cm^{-1}
2:	0.00 cm^{-1}	9:	231.13 cm^{-1}	16:	468.75 cm^{-1}
3:	0.00 cm^{-1}	10:	231.17 cm^{-1}	17:	604.61 cm^{-1}
4:	0.00 cm^{-1}	11:	248.20 cm^{-1}	18:	666.54 cm^{-1}
5:	0.00 cm^{-1}	12:	351.84 cm^{-1}	19:	666.55 cm^{-1}
6:	103.66 cm^{-1}	13:	394.07 cm^{-1}	20:	719.77 cm^{-1}

$[SF_6]^-$ – PBE0-D3BJ/def2-TZVP/CPCM(THF) (D_1)

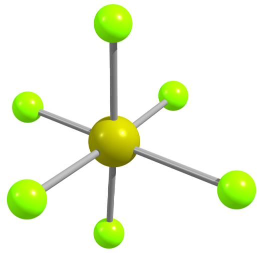

F	0.166366000	-0.347523000	-2.822566000
S	0.169609000	-0.347138000	-0.884381000
F	0.172230000	-0.346815000	0.717707000
F	0.169700000	1.338356000	-0.826362000
F	0.169701000	-2.032622000	-0.825678000
F	-1.515766000	-0.347125000	-0.823271000
F	1.855208000	-0.347125000	-0.828768000

0:	0.00 cm^{-1}	7:	122.37 cm^{-1}	14:	420.38 cm^{-1}
1:	0.00 cm^{-1}	8:	205.07 cm^{-1}	15:	465.62 cm^{-1}
2:	0.00 cm^{-1}	9:	240.16 cm^{-1}	16:	483.99 cm^{-1}
3:	0.00 cm^{-1}	10:	240.17 cm^{-1}	17:	590.28 cm^{-1}
4:	0.00 cm^{-1}	11:	257.95 cm^{-1}	18:	613.29 cm^{-1}
5:	0.00 cm^{-1}	12:	364.53 cm^{-1}	19:	613.30 cm^{-1}
6:	122.35 cm^{-1}	13:	420.35 cm^{-1}	20:	723.29 cm^{-1}

$[SF_5]^-$ – PBE0-D3BJ/def2-TZVP (S_0)

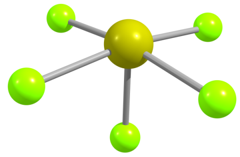

F	0.183866000	-0.395762000	-2.631776000
S	0.183866000	-0.249121000	-0.907964000
F	0.183866000	-0.395762000	0.815847000
F	0.183866000	-1.846614000	-0.907964000
F	-1.539945000	-0.395762000	-0.907964000
F	1.907677000	-0.395762000	-0.907965000

0:	0.00 cm^{-1}	6:	243.27 cm^{-1}	12:	469.05 cm^{-1}
1:	0.00 cm^{-1}	7:	243.32 cm^{-1}	13:	469.08 cm^{-1}
2:	0.00 cm^{-1}	8:	260.96 cm^{-1}	14:	526.52 cm^{-1}
3:	0.00 cm^{-1}	9:	334.33 cm^{-1}	15:	631.47 cm^{-1}
4:	0.00 cm^{-1}	10:	431.22 cm^{-1}	16:	631.49 cm^{-1}
5:	0.00 cm^{-1}	11:	451.80 cm^{-1}	17:	812.73 cm^{-1}

$[SF_5]^-$ – PBE0-D3BJ/def2-TZVP/CPCM(THF) (S_0)

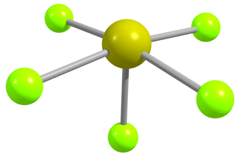

F	0.183866000	-0.398124000	-2.630257000
S	0.183866000	-0.244226000	-0.907964000
F	0.183866000	-0.398124000	0.814329000
F	0.183866000	-1.842060000	-0.907964000
F	-1.538427000	-0.398124000	-0.907965000
F	1.906159000	-0.398124000	-0.907964000

0:	0.00 cm^{-1}	6:	238.74 cm^{-1}	12:	461.92 cm^{-1}
1:	0.00 cm^{-1}	7:	238.78 cm^{-1}	13:	461.94 cm^{-1}
2:	0.00 cm^{-1}	8:	264.43 cm^{-1}	14:	523.93 cm^{-1}
3:	0.00 cm^{-1}	9:	334.91 cm^{-1}	15:	559.72 cm^{-1}
4:	0.00 cm^{-1}	10:	424.79 cm^{-1}	16:	559.72 cm^{-1}
5:	0.00 cm^{-1}	11:	445.59 cm^{-1}	17:	792.95 cm^{-1}

$[SF_5]^-$ – PBE0-D3BJ/def2-TZVP (T_1)

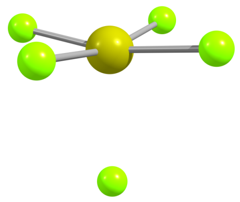

F	0.183866000	-0.196397000	-2.592585000
S	0.183866000	-0.386547000	-0.907964000
F	0.183866000	-0.196397000	0.776657000
F	0.183867000	-2.506645000	-0.907965000
F	-1.500755000	-0.196397000	-0.907964000
F	1.868487000	-0.196398000	-0.907964000

0:	0.00 cm^{-1}	6:	77.17 cm^{-1}	12:	368.33 cm^{-1}
1:	0.00 cm^{-1}	7:	77.22 cm^{-1}	13:	476.44 cm^{-1}
2:	0.00 cm^{-1}	8:	168.12 cm^{-1}	14:	550.72 cm^{-1}
3:	0.00 cm^{-1}	9:	293.87 cm^{-1}	15:	570.21 cm^{-1}
4:	0.00 cm^{-1}	10:	293.91 cm^{-1}	16:	648.28 cm^{-1}
5:	0.00 cm^{-1}	11:	296.20 cm^{-1}	17:	648.29 cm^{-1}

$[SF_5]^–$ – PBE0-D3BJ/def2-TZVP/CPCM(THF) (T_1)

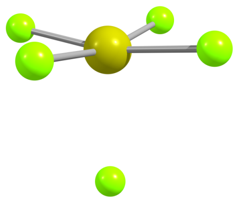

F	0.183866000	-0.204187000	-2.585569000
S	0.183866000	-0.362604000	-0.907964000
F	0.183866000	-0.204187000	0.769640000
F	0.183865000	-2.499428000	-0.907964000
F	-1.493738000	-0.204188000	-0.907965000
F	1.861470000	-0.204187000	-0.907964000

0:	0.00 cm^{-1}	6:	88.69 cm^{-1}	12:	374.30 cm^{-1}
1:	0.00 cm^{-1}	7:	88.71 cm^{-1}	13:	482.73 cm^{-1}
2:	0.00 cm^{-1}	8:	167.26 cm^{-1}	14:	517.45 cm^{-1}
3:	0.00 cm^{-1}	9:	300.75 cm^{-1}	15:	577.54 cm^{-1}
4:	0.00 cm^{-1}	10:	300.77 cm^{-1}	16:	599.16 cm^{-1}
5:	0.00 cm^{-1}	11:	308.52 cm^{-1}	17:	599.17 cm^{-1}

H_2O – PBE0-D3BJ/def2-TZVP (S_0)

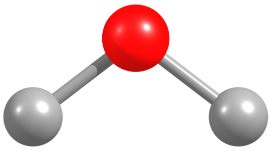

O	-0.007818000	0.013542000	-0.057310000
H	-0.889927000	0.018253000	0.320220000
H	0.429156000	0.779826000	0.320220000

0:	0.00 cm^{-1}	3:	0.00 cm^{-1}	6:	1623.13 cm^{-1}
1:	0.00 cm^{-1}	4:	0.00 cm^{-1}	7:	3850.77 cm^{-1}
2:	0.00 cm^{-1}	5:	0.00 cm^{-1}	8:	3957.29 cm^{-1}

H_2O – PBE0-D3BJ/def2-TZVP/CPCM(THF) (S_0)

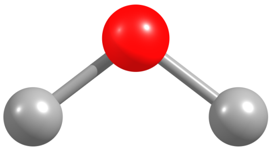

O	-0.006332000	0.010971000	-0.059829000
H	-0.889676000	0.020113000	0.321480000
H	0.427418000	0.780537000	0.321479000

0:	0.00 cm^{-1}	3:	0.00 cm^{-1}	6:	1609.02 cm^{-1}
1:	0.00 cm^{-1}	4:	0.00 cm^{-1}	7:	3811.39 cm^{-1}
2:	0.00 cm^{-1}	5:	0.00 cm^{-1}	8:	3900.65 cm^{-1}

$[OH]^{•}$ – PBE0-D3BJ/def2-TZVP (D_1)

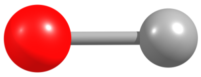

O	-0.001325000	0.010919000	-0.061990000
H	-0.894683000	0.020165000	0.323641000

0:	0.00 cm^{-1}	2:	0.00 cm^{-1}	4:	0.00 cm^{-1}
1:	0.00 cm^{-1}	3:	0.00 cm^{-1}	5:	3747.39 cm^{-1}

1

[OH]$^{\bullet}$ – PBE0-D3BJ/def2-TZVP/CPCM(THF) (D_1)

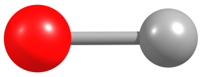

O	0.000111000	0.010904000	-0.062610000
H	-0.896119000	0.020180000	0.324261000

0: 0.00 cm^{-1}	2: 0.00 cm^{-1}	4: 0.00 cm^{-1}
1: 0.00 cm^{-1}	3: 0.00 cm^{-1}	5: 3695.67 cm^{-1}

[H_2O]$^+$ – PBE0-D3BJ/def2-TZVP (D_1)

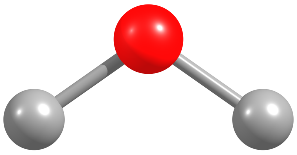

O	-0.009412000	0.016303000	-0.054606000
H	-0.940698000	-0.012899000	0.318868000
H	0.481520000	0.808218000	0.318868000

0: 0.00 cm^{-1}	3: 0.00 cm^{-1}	6: 1440.60 cm^{-1}
1: 0.00 cm^{-1}	4: 0.00 cm^{-1}	7: 3374.98 cm^{-1}
2: 0.00 cm^{-1}	5: 0.00 cm^{-1}	8: 3420.73 cm^{-1}

[H_2O]$^+$ – PBE0-D3BJ/def2-TZVP/CPCM(THF) (D_1)

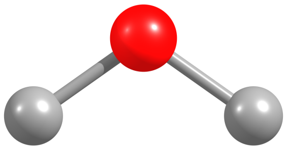

O	-0.004167000	0.007320000	-0.063429000
H	-0.940750000	-0.006907000	0.323292000
H	0.476327000	0.811208000	0.323266000

0: 0.00 cm^{-1}	3: 0.00 cm^{-1}	6: 1417.95 cm^{-1}
1: 0.00 cm^{-1}	4: 0.00 cm^{-1}	7: 3241.90 cm^{-1}
2: 0.00 cm^{-1}	5: 0.00 cm^{-1}	8: 3254.09 cm^{-1}

[OH]$^+$ – PBE0-D3BJ/def2-TZVP (S_0)

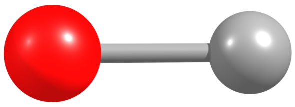

O	0.008573000	0.000000000	-0.074207000
H	-0.945752000	0.000000000	0.330486000

0: 0.00 cm^{-1}	2: 0.00 cm^{-1}	4: 0.00 cm^{-1}
1: 0.00 cm^{-1}	3: 0.00 cm^{-1}	5: 3095.26 cm^{-1}

[OH]$^+$ – PBE0-D3BJ/def2-TZVP/CPCM(THF) (S_0)

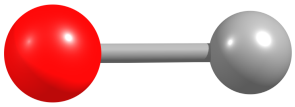

O 0.017285000 H -0.954465000
0.000000000 - 0.000000000
0.077902000 0.334181000

0: 0.00 cm^{-1}	2: 0.00 cm^{-1}	4: 0.00 cm^{-1}
1: 0.00 cm^{-1}	3: 0.00 cm^{-1}	5: 2855.89 cm^{-1}

[OH]$^+$ – PBE0-D3BJ/def2-TZVP (T_1)

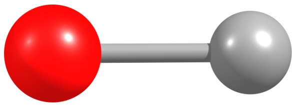

O	0.007920000	0.000000000	-0.073930000
H	-0.945099000	0.000000000	0.330209000

0: 0.00 cm^{-1}	2: 0.00 cm^{-1}	4: 0.00 cm^{-1}
1: 0.00 cm^{-1}	3: 0.00 cm^{-1}	5: 3097.27 cm^{-1}

[OH]$^+$ – PBE0-D3BJ/def2-TZVP/CPCM(THF) (T_1)

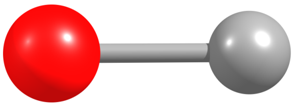

O	0.015988000	0.000000000	-0.077352000
H	-0.953168000	0.000000000	0.333631000

0: 0.00 cm^{-1}
1: 0.00 cm^{-1}
2: 0.00 cm^{-1}
3: 0.00 cm^{-1}
4: 0.00 cm^{-1}
5: 2876.87 cm^{-1}

[H_2O]$^-$ – PBE0-D3BJ/def2-TZVP (D_1)

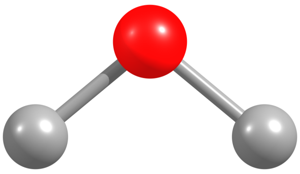

O	0.007491000	-0.012972000	-0.083277000
H	-0.907410000	0.025837000	0.333204000
H	0.431329000	0.798757000	0.333203000

0: 0.00 cm^{-1}
1: 0.00 cm^{-1}
2: 0.00 cm^{-1}
3: 0.00 cm^{-1}
4: 0.00 cm^{-1}
5: 0.00 cm^{-1}
6: 1344.04 cm^{-1}
7: 2692.03 cm^{-1}
8: 3060.05 cm^{-1}

[H_2O]$^-$ – PBE0-D3BJ/def2-TZVP/CPCM(THF) (D_1)

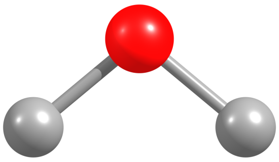

O	0.019204000	-0.006792000	-0.083704000
H	-0.914496000	0.026989000	0.337055000
H	0.426702000	0.791424000	0.329779000

0: 0.00 cm^{-1}
1: 0.00 cm^{-1}
2: 0.00 cm^{-1}
3: 0.00 cm^{-1}
4: 0.00 cm^{-1}
5: 0.00 cm^{-1}
6: 1289.37 cm^{-1}
7: 1983.34 cm^{-1}
8: 3175.88 cm^{-1}

[OH]⁻ – PBE0-D3BJ/def2-TZVP (S_0)

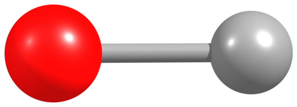

O	-0.023925000	0.000000000	-0.060426000
H	-0.913255000	0.000000000	0.316705000

0:	0.00 cm^{-1}	2:	0.00 cm^{-1}	4:	0.00 cm^{-1}
1:	0.00 cm^{-1}	3:	0.00 cm^{-1}	5:	3722.93 cm^{-1}

[OH]⁻ – PBE0-D3BJ/def2-TZVP/CPCM(THF) (S_0)

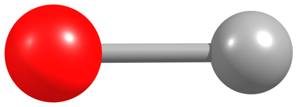

O	-0.025225000	0.000000000	-0.059875000
H	-0.911955000	0.000000000	0.316154000

0:	0.00 cm^{-1}	2:	0.00 cm^{-1}	4:	0.00 cm^{-1}
1:	0.00 cm^{-1}	3:	0.00 cm^{-1}	5:	3773.05 cm^{-1}

NH_3 – PBE0-D3BJ/def2-TZVP (S_0)

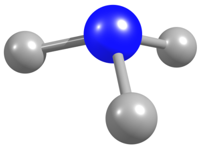

N	0.000009000	0.000000000	-0.053859000
H	-0.940297000	0.000000000	0.321283000
H	0.470144000	0.814337000	0.321278000
H	0.470144000	-0.814337000	0.321278000

0:	0.00 cm^{-1}	4:	0.00 cm^{-1}	8:	1670.24 cm^{-1}
1:	0.00 cm^{-1}	5:	0.00 cm^{-1}	9:	3503.26 cm^{-1}
2:	0.00 cm^{-1}	6:	1030.21 cm^{-1}	10:	3630.86 cm^{-1}
3:	0.00 cm^{-1}	7:	1670.03 cm^{-1}	11:	3630.91 cm^{-1}

NH_3 – PBE0-D3BJ/def2-TZVP/CPCM(THF) (S_0)

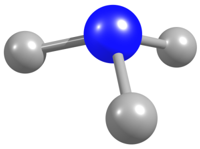

N	-0.000018000	0.000000000	-0.062256000
H	-0.937869000	0.000000000	0.324074000
H	0.468943000	0.812195000	0.324081000
H	0.468943000	-0.812195000	0.324081000

0:	0.00 cm^{-1}	4:	0.00 cm^{-1}	8:	1638.80 cm^{-1}
1:	0.00 cm^{-1}	5:	0.00 cm^{-1}	9:	3490.56 cm^{-1}
2:	0.00 cm^{-1}	6:	1064.66 cm^{-1}	10:	3607.66 cm^{-1}
3:	0.00 cm^{-1}	7:	1637.34 cm^{-1}	11:	3608.11 cm^{-1}

$[NH_2]^•$ – PBE0-D3BJ/def2-TZVP (D_1)

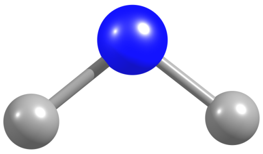

N	0.006065000	-0.010504000	-0.080859000
H	-0.933790000	0.008960000	0.331995000
H	0.459136000	0.813166000	0.331994000

0:	0.00 cm^{-1}	3:	0.00 cm^{-1}	6:	1529.56 cm^{-1}
1:	0.00 cm^{-1}	4:	0.00 cm^{-1}	7:	3383.85 cm^{-1}
2:	0.00 cm^{-1}	5:	0.00 cm^{-1}	8:	3479.34 cm^{-1}

$[NH_2]^{\bullet}$ – PBE0-D3BJ/def2-TZVP/CPCM(THF) (D_1)

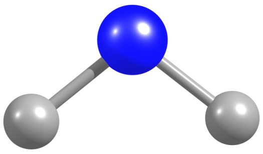

N	0.005734000	-0.009940000	-0.080305000
H	-0.933873000	0.008533000	0.331716000
H	0.459549000	0.813028000	0.331718000

0: 0.00 cm^{-1}	3: 0.00 cm^{-1}	6: 1505.16 cm^{-1}
1: 0.00 cm^{-1}	4: 0.00 cm^{-1}	7: 3401.75 cm^{-1}
2: 0.00 cm^{-1}	5: 0.00 cm^{-1}	8: 3488.46 cm^{-1}

$[NH_3]^{+}$ – PBE0-D3BJ/def2-TZVP (D_1)

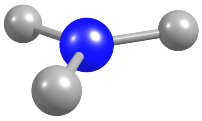

N	-0.000003000	0.000000000	0.227494000
H	-1.023977000	0.000000000	0.230802000
H	0.511990000	0.886789000	0.225842000
H	0.511990000	-0.886789000	0.225842000

0: 0.00 cm^{-1}	4: 0.00 cm^{-1}	8: 1530.95 cm^{-1}
1: 0.00 cm^{-1}	5: 0.00 cm^{-1}	9: 3369.15 cm^{-1}
2: 0.00 cm^{-1}	6: 868.76 cm^{-1}	10: 3543.99 cm^{-1}
3: 0.00 cm^{-1}	7: 1530.81 cm^{-1}	11: 3544.01 cm^{-1}

$[NH_3]^+$ – PBE0-D3BJ/def2-TZVP/CPCM(THF) (D_1)

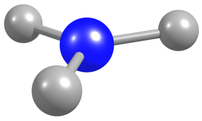

N	-0.000137000	0.000000000	0.227389000
H	-1.022138000	0.000000000	0.230643000
H	0.511138000	0.885077000	0.225975000
H	0.511138000	-0.885077000	0.225975000

0:	0.00 cm^{-1}	4:	0.00 cm^{-1}	8:	1515.51 cm^{-1}
1:	0.00 cm^{-1}	5:	0.00 cm^{-1}	9:	3400.78 cm^{-1}
2:	0.00 cm^{-1}	6:	863.89 cm^{-1}	10:	3573.59 cm^{-1}
3:	0.00 cm^{-1}	7:	1494.33 cm^{-1}	11:	3577.13 cm^{-1}

$[NH_2]^+$ – PBE0-D3BJ/def2-TZVP (S_0)

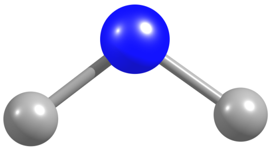

N	0.002963000	-0.005133000	-0.075598000
H	-0.970509000	-0.015821000	0.329364000
H	0.498956000	0.832575000	0.329364000

0:	0.00 cm^{-1}	3:	0.00 cm^{-1}	6:	1418.47 cm^{-1}
1:	0.00 cm^{-1}	4:	0.00 cm^{-1}	7:	3159.89 cm^{-1}
2:	0.00 cm^{-1}	5:	0.00 cm^{-1}	8:	3238.22 cm^{-1}

$[NH_2]^+$ – PBE0-D3BJ/def2-TZVP/CPCM(THF) (S_0)

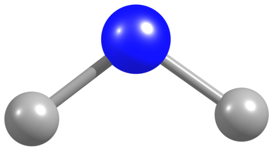

N	0.005306000	-0.009086000	-0.079496000
H	-0.963632000	-0.009180000	0.331325000
H	0.489736000	0.829888000	0.331300000

0:	0.00 cm^{-1}	3:	0.00 cm^{-1}	6:	1411.35 cm^{-1}
1:	0.00 cm^{-1}	4:	0.00 cm^{-1}	7:	3203.87 cm^{-1}
2:	0.00 cm^{-1}	5:	0.00 cm^{-1}	8:	3262.10 cm^{-1}

$[NH_2]^+$ – PBE0-D3BJ/def2-TZVP (T_1)

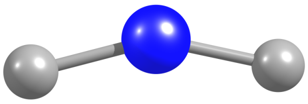

N	-0.095849000	0.166015000	0.092012000
H	-1.058124000	-0.180504000	0.245559000
H	0.685383000	0.826110000	0.245559000

0:	0.00 cm^{-1}	3:	0.00 cm^{-1}	6:	707.78 cm^{-1}
1:	0.00 cm^{-1}	4:	0.00 cm^{-1}	7:	3240.82 cm^{-1}
2:	0.00 cm^{-1}	5:	0.00 cm^{-1}	8:	3501.50 cm^{-1}

$[NH_2]^+$ – PBE0-D3BJ/def2-TZVP/CPCM(THF) (T_1)

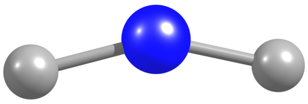

N	-0.095544000	0.165058000	0.091180000
H	-1.057603000	-0.179669000	0.245951000
H	0.684557000	0.826232000	0.245999000

0:	0.00 cm^{-1}	3:	0.00 cm^{-1}	6:	707.26 cm^{-1}
1:	0.00 cm^{-1}	4:	0.00 cm^{-1}	7:	3246.36 cm^{-1}
2:	0.00 cm^{-1}	5:	0.00 cm^{-1}	8:	3494.86 cm^{-1}

$[NH_3]^-$ – PBE0-D3BJ/def2-TZVP (D_1)

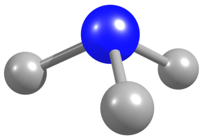

N	0.000006000	0.000000000	-0.116952000
H	-0.935234000	0.000000000	0.342314000
H	0.467614000	0.809950000	0.342310000
H	0.467614000	-0.809950000	0.342310000

0: 0.00 cm^{-1}	4: 0.00 cm^{-1}	8: 1533.62 cm^{-1}
1: 0.00 cm^{-1}	5: 0.00 cm^{-1}	9: 3027.42 cm^{-1}
2: 0.00 cm^{-1}	6: 1095.89 cm^{-1}	10: 3054.24 cm^{-1}
3: 0.00 cm^{-1}	7: 1533.42 cm^{-1}	11: 3054.56 cm^{-1}

$[NH_3]^-$ – PBE0-D3BJ/def2-TZVP/CPCM(THF) (D_1)

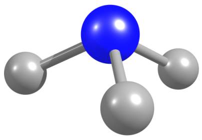

N	-0.000063000	0.000000000	-0.106212000
H	-0.945729000	0.000000000	0.338715000
H	0.472896000	0.818877000	0.338739000
H	0.472896000	-0.818877000	0.338739000

0: 0.00 cm^{-1}	4: 0.00 cm^{-1}	8: 1483.89 cm^{-1}
1: 0.00 cm^{-1}	5: 0.00 cm^{-1}	9: 2786.06 cm^{-1}
2: 0.00 cm^{-1}	6: 1048.97 cm^{-1}	10: 2788.57 cm^{-1}
3: 0.00 cm^{-1}	7: 1483.22 cm^{-1}	11: 2969.09 cm^{-1}

[NH_2]$^-$ – PBE0-D3BJ/def2-TZVP (S_0)

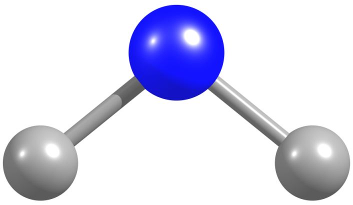

N	0.011967000	-0.020726000	-0.090870000
H	-0.927073000	0.019653000	0.337000000
H	0.446517000	0.812695000	0.337000000

0:	0.00 cm^{-1}	3:	0.00 cm^{-1}	6:	1520.66 cm^{-1}
1:	0.00 cm^{-1}	4:	0.00 cm^{-1}	7:	3253.49 cm^{-1}
2:	0.00 cm^{-1}	5:	0.00 cm^{-1}	8:	3325.87 cm^{-1}

[NH_2]$^-$ – PBE0-D3BJ/def2-TZVP/CPCM(THF) (S_0)

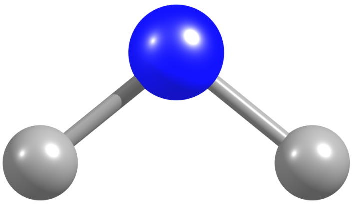

N	0.007846000	-0.013558000	-0.083857000
H	-0.927044000	0.014901000	0.333497000
H	0.450608000	0.810279000	0.333490000

0:	0.00 cm^{-1}	3:	0.00 cm^{-1}	6:	1503.47 cm^{-1}
1:	0.00 cm^{-1}	4:	0.00 cm^{-1}	7:	3364.33 cm^{-1}
2:	0.00 cm^{-1}	5:	0.00 cm^{-1}	8:	3442.87 cm^{-1}

$[NH_2]^-$ – PBE0-D3BJ/def2-TZVP (T_1)

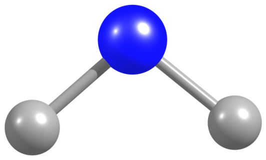

N	0.018105000	-0.031359000	-0.101283000
H	-0.951369000	0.012713000	0.342206000
H	0.464675000	0.830267000	0.342207000

0:	0.00 cm^{-1}	3:	0.00 cm^{-1}	6:	1310.17 cm^{-1}
1:	0.00 cm^{-1}	4:	0.00 cm^{-1}	7:	2554.04 cm^{-1}
2:	0.00 cm^{-1}	5:	0.00 cm^{-1}	8:	2689.04 cm^{-1}

$[NH_2]^-$ – PBE0-D3BJ/def2-TZVP/CPCM(THF) (T_1)

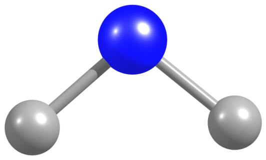

N	0.040725000	-0.020006000	-0.102536000
H	-0.985402000	0.002826000	0.349376000
H	0.476088000	0.828802000	0.336290000

0:	0.00 cm^{-1}	3:	0.00 cm^{-1}	6:	1140.59 cm^{-1}
1:	0.00 cm^{-1}	4:	0.00 cm^{-1}	7:	1266.30 cm^{-1}
2:	0.00 cm^{-1}	5:	0.00 cm^{-1}	8:	2876.31 cm^{-1}

SiH_4 – PBE0-D3BJ/def2-TZVP (S_0)

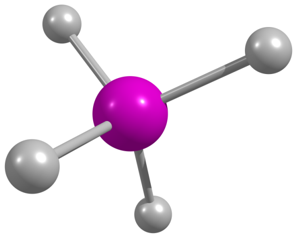

Si	0.211328000	-0.005131000	0.123268000
H	0.932711000	-1.298883000	0.226905000
H	1.185820000	1.112506000	0.201490000
H	-0.763086000	0.108860000	1.237874000
H	-0.509982000	0.056899000	-1.173108000

0:	0.00 cm^{-1}	5:	0.00 cm^{-1}	10:	968.23 cm^{-1}
1:	0.00 cm^{-1}	6:	906.02 cm^{-1}	11:	2234.58 cm^{-1}
2:	0.00 cm^{-1}	7:	906.56 cm^{-1}	12:	2242.09 cm^{-1}
3:	0.00 cm^{-1}	8:	906.68 cm^{-1}	13:	2242.11 cm^{-1}
4:	0.00 cm^{-1}	9:	967.99 cm^{-1}	14:	2242.40 cm^{-1}

SiH_4 – PBE0-D3BJ/def2-TZVP/CPCM(THF) (S_0)

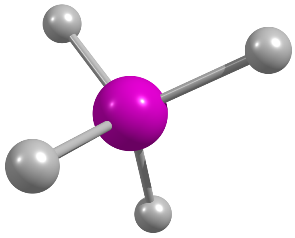

Si	0.211324000	-0.005119000	0.123273000
H	0.932549000	-1.298611000	0.226879000
H	1.185626000	1.112279000	0.201470000
H	-0.762885000	0.108826000	1.237655000
H	-0.509823000	0.056876000	-1.172848000

0:	0.00 cm^{-1}	5:	0.00 cm^{-1}	10:	966.87 cm^{-1}
1:	0.00 cm^{-1}	6:	885.25 cm^{-1}	11:	2233.63 cm^{-1}
2:	0.00 cm^{-1}	7:	885.34 cm^{-1}	12:	2238.53 cm^{-1}
3:	0.00 cm^{-1}	8:	885.62 cm^{-1}	13:	2238.59 cm^{-1}
4:	0.00 cm^{-1}	9:	966.48 cm^{-1}	14:	2238.69 cm^{-1}

[SiH$_3$]$^•$ – PBE0-D3BJ/def2-TZVP (D$_1$)

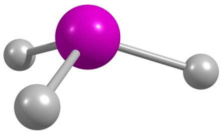

Si	0.179416000	-0.002260000	0.066049000
H	0.885127000	-1.308638000	0.127994000
H	1.141223000	1.129928000	0.102013000
H	-0.829988000	0.114780000	1.150304000

0:	0.00 cm^{-1}	4:	0.00 cm^{-1}	8:	926.23 cm^{-1}
1:	0.00 cm^{-1}	5:	0.00 cm^{-1}	9:	2203.18 cm^{-1}
2:	0.00 cm^{-1}	6:	746.40 cm^{-1}	10:	2240.74 cm^{-1}
3:	0.00 cm^{-1}	7:	926.08 cm^{-1}	11:	2241.53 cm^{-1}

[SiH$_3$]$^•$ – PBE0-D3BJ/def2-TZVP/CPCM(THF) (D$_1$)

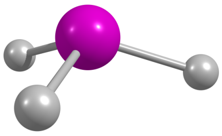

Si	0.180552000	-0.002327000	0.068091000
H	0.884949000	-1.309169000	0.127194000
H	1.141176000	1.130465000	0.101160000
H	-0.830900000	0.114840000	1.149916000

0:	0.00 cm^{-1}	4:	0.00 cm^{-1}	8:	915.40 cm^{-1}
1:	0.00 cm^{-1}	5:	0.00 cm^{-1}	9:	2202.68 cm^{-1}
2:	0.00 cm^{-1}	6:	730.39 cm^{-1}	10:	2237.38 cm^{-1}
3:	0.00 cm^{-1}	7:	914.88 cm^{-1}	11:	2238.25 cm^{-1}

[SiH_4]$^-$ – PBE0-D3BJ/def2-TZVP (D_1)

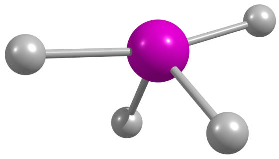

Si	0.240108000	0.212478000	-0.081407000
H	1.014043000	-1.095727000	0.008709000
H	1.368018000	1.067194000	0.714041000
H	-0.713213000	0.163527000	1.104823000
H	-0.852165000	-0.373220000	-1.129737000

0:	0.00 cm^{-1}	5:	0.00 cm^{-1}	10:	1033.39 cm^{-1}
1:	0.00 cm^{-1}	6:	715.80 cm^{-1}	11:	1405.05 cm^{-1}
2:	0.00 cm^{-1}	7:	770.19 cm^{-1}	12:	1496.77 cm^{-1}
3:	0.00 cm^{-1}	8:	827.78 cm^{-1}	13:	2009.29 cm^{-1}
4:	0.00 cm^{-1}	9:	983.59 cm^{-1}	14:	2011.46 cm^{-1}

[SiH_4]$^-$ – PBE0-D3BJ/def2-TZVP/CPCM(THF) (D_1)

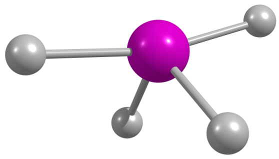

Si	0.200737000	0.215648000	0.324140000
H	0.937744000	-1.104009000	0.430890000
H	1.492325000	1.022590000	-0.221481000
H	-1.103618000	-0.321345000	1.116233000
H	-0.470395000	0.161368000	-1.033353000

0:	0.00 cm^{-1}	5:	0.00 cm^{-1}	10:	1043.15 cm^{-1}
1:	0.00 cm^{-1}	6:	711.00 cm^{-1}	11:	1384.80 cm^{-1}
2:	0.00 cm^{-1}	7:	775.06 cm^{-1}	12:	1512.44 cm^{-1}
3:	0.00 cm^{-1}	8:	830.99 cm^{-1}	13:	2040.69 cm^{-1}
4:	0.00 cm^{-1}	9:	950.64 cm^{-1}	14:	2046.64 cm^{-1}

$[SiH_3]^-$ – PBE0-D3BJ/def2-TZVP (S_0)

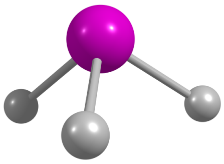

Si	0.041472000	0.008754000	-0.182657000
H	0.893169000	-1.210163000	0.238473000
H	1.127680000	1.034764000	0.212855000
H	-0.686543000	0.100454000	1.177690000

0:	0.00 cm^{-1}	4:	0.00 cm^{-1}	8:	954.80 cm^{-1}
1:	0.00 cm^{-1}	5:	0.00 cm^{-1}	9:	1900.29 cm^{-1}
2:	0.00 cm^{-1}	6:	866.82 cm^{-1}	10:	1900.91 cm^{-1}
3:	0.00 cm^{-1}	7:	954.47 cm^{-1}	11:	1916.31 cm^{-1}

$[SiH_3]^-$ – PBE0-D3BJ/def2-TZVP/CPCM(THF) (S_0)

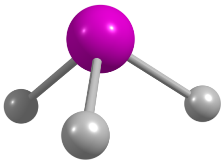

Si	0.045027000	0.008212000	-0.176004000
H	0.890907000	-1.206745000	0.236783000
H	1.124327000	1.031675000	0.211884000
H	-0.684484000	0.100667000	1.173697000

0:	0.00 cm^{-1}	4:	0.00 cm^{-1}	8:	960.95 cm^{-1}
1:	0.00 cm^{-1}	5:	0.00 cm^{-1}	9:	1929.35 cm^{-1}
2:	0.00 cm^{-1}	6:	855.23 cm^{-1}	10:	1933.35 cm^{-1}
3:	0.00 cm^{-1}	7:	959.68 cm^{-1}	11:	1953.78 cm^{-1}

[SiH_3]$^-$ – PBE0-D3BJ/def2-TZVP (T_1)

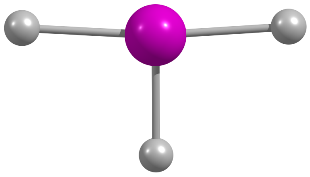

Si	0.187712000	0.278168000	0.351858000
H	0.902336000	-1.069585000	0.396448000
H	1.459371000	1.037442000	-0.351185000
H	-1.173642000	-0.312216000	1.049239000

0:	0.00 cm^{-1}	4:	0.00 cm^{-1}	8:	1018.26 cm^{-1}
1:	0.00 cm^{-1}	5:	0.00 cm^{-1}	9:	1301.74 cm^{-1}
2:	0.00 cm^{-1}	6:	729.42 cm^{-1}	10:	1494.36 cm^{-1}
3:	0.00 cm^{-1}	7:	797.65 cm^{-1}	11:	1991.29 cm^{-1}

[SiH_3]$^-$ – PBE0-D3BJ/def2-TZVP/CPCM(THF) (T_1)

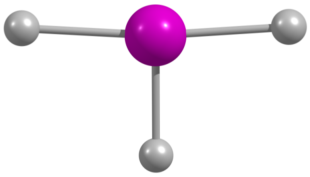

Si	0.150697000	-0.284069000	0.404324000
H	1.245493000	-1.296182000	-0.254779000
H	1.032486000	0.937605000	0.210139000
H	-1.052900000	0.576455000	1.086676000

0:	0.00 cm^{-1}	4:	0.00 cm^{-1}	8:	1008.96 cm^{-1}
1:	0.00 cm^{-1}	5:	0.00 cm^{-1}	9:	1308.57 cm^{-1}
2:	0.00 cm^{-1}	6:	729.53 cm^{-1}	10:	1537.58 cm^{-1}
3:	0.00 cm^{-1}	7:	801.50 cm^{-1}	11:	2017.62 cm^{-1}

Cr(C_6H_6)(CO)$_3$ – PBE0-D3BJ/def2-TZVP (S_0)

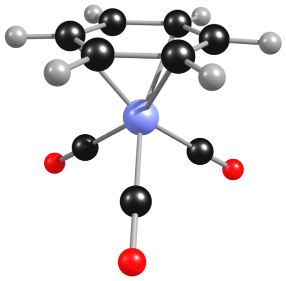

Cr	2.979601000	-0.594914000	-2.605362000
C	2.645627000	-2.235970000	-1.850000000
C	1.608837000	0.101173000	-1.599563000
C	4.126761000	-0.234755000	-1.216305000
O	2.438535000	-3.268418000	-1.394441000
O	0.746936000	0.544657000	-0.985713000
O	4.854954000	-0.003315000	-0.360424000
C	2.453911000	-1.386054000	-4.581540000
C	1.888478000	-0.114691000	-4.445647000
C	3.841206000	-1.569787000	-4.370314000
H	0.821539000	0.017952000	-4.562598000
H	4.269922000	-2.560798000	-4.431341000
C	2.703386000	0.988512000	-4.096652000
C	4.646147000	-0.479942000	-4.025577000
H	2.258001000	1.962667000	-3.947336000
H	5.697542000	-0.627918000	-3.819891000
C	4.073804000	0.807064000	-3.887953000
H	4.688568000	1.640842000	-3.577293000
H	1.824381000	-2.236923000	-4.803693000

0:	0.00 cm^{-1}	9:	96.79 cm^{-1}	19:	492.03 cm^{-1}
1:	0.00 cm^{-1}	10:	120.50 cm^{-1}	20:	493.85 cm^{-1}
2:	0.00 cm^{-1}	11:	120.68 cm^{-1}	21:	556.86 cm^{-1}
3:	0.00 cm^{-1}	12:	315.38 cm^{-1}	22:	557.35 cm^{-1}
4:	0.00 cm^{-1}	13:	340.31 cm^{-1}	23:	625.14 cm^{-1}
5:	0.00 cm^{-1}	14:	340.69 cm^{-1}	24:	627.57 cm^{-1}
6:	31.36 cm^{-1}	15:	423.88 cm^{-1}	25:	666.54 cm^{-1}
7:	85.03 cm^{-1}	16:	434.80 cm^{-1}	26:	667.08 cm^{-1}
8:	85.80 cm^{-1}	17:	435.45 cm^{-1}	27:	674.00 cm^{-1}
		18:	490.55 cm^{-1}	28:	694.60 cm^{-1}

29: 809.12 cm^{-1}
30: 897.18 cm^{-1}
31: 897.99 cm^{-1}
32: 955.55 cm^{-1}
33: 955.92 cm^{-1}
34: 986.53 cm^{-1}
35: 1008.69 cm^{-1}
36: 1035.64 cm^{-1}
37: 1039.46 cm^{-1}
38: 1042.03 cm^{-1}
39: 1171.52 cm^{-1}
40: 1171.87 cm^{-1}
41: 1174.68 cm^{-1}
42: 1359.52 cm^{-1}
43: 1445.46 cm^{-1}
44: 1480.50 cm^{-1}
45: 1482.83 cm^{-1}
46: 1567.47 cm^{-1}
47: 1568.90 cm^{-1}
48: 2037.55 cm^{-1}
49: 2037.91 cm^{-1}
50: 2092.02 cm^{-1}
51: 3215.71 cm^{-1}
52: 3221.72 cm^{-1}
53: 3223.02 cm^{-1}
54: 3233.93 cm^{-1}
55: 3235.74 cm^{-1}
56: 3238.87 cm^{-1}

$Cr(C_6H_6)(CO)_3$ – PBE0-D3BJ/def2-TZVP/CPCM(THF) (S_0)

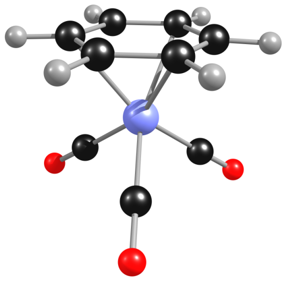

Cr	2.977309000	-0.597041000	-2.593882000
C	2.639983000	-2.220203000	-1.840481000
C	1.623333000	0.094420000	-1.591673000
C	4.111969000	-0.245905000	-1.213588000
O	2.426580000	-3.247364000	-1.364025000
O	0.766555000	0.532050000	-0.957601000
O	4.829929000	-0.023068000	-0.340378000
C	2.456573000	-1.385323000	-4.587940000
C	1.890665000	-0.114285000	-4.452945000
C	3.843091000	-1.568499000	-4.376306000
H	0.824157000	0.020043000	-4.575581000
H	4.272290000	-2.559412000	-4.442576000
C	2.704605000	0.988600000	-4.103843000
C	4.647257000	-0.478417000	-4.031286000
H	2.258469000	1.963809000	-3.960585000
H	5.700299000	-0.623843000	-3.830061000
C	4.074814000	0.807803000	-3.894482000
H	4.691390000	1.642634000	-3.588841000
H	1.828866000	-2.236617000	-4.815568000

0:	0.00 cm^{-1}	10:	115.46 cm^{-1}	20:	505.95 cm^{-1}
1:	0.00 cm^{-1}	11:	119.77 cm^{-1}	21:	561.65 cm^{-1}
2:	0.00 cm^{-1}	12:	302.23 cm^{-1}	22:	562.43 cm^{-1}
3:	0.00 cm^{-1}	13:	331.86 cm^{-1}	23:	621.54 cm^{-1}
4:	0.00 cm^{-1}	14:	334.02 cm^{-1}	24:	621.79 cm^{-1}
5:	0.00 cm^{-1}	15:	432.52 cm^{-1}	25:	668.53 cm^{-1}
6:	15.53 cm^{-1}	16:	435.78 cm^{-1}	26:	669.73 cm^{-1}
7:	84.57 cm^{-1}	17:	436.37 cm^{-1}	27:	683.77 cm^{-1}
8:	85.61 cm^{-1}	18:	502.81 cm^{-1}	28:	700.04 cm^{-1}
9:	98.29 cm^{-1}	19:	505.05 cm^{-1}	29:	802.95 cm^{-1}

30:	907.00 cm^{-1}	39:	1154.27 cm^{-1}	48:	1979.73 cm^{-1}
31:	907.72 cm^{-1}	40:	1161.90 cm^{-1}	49:	1980.45 cm^{-1}
32:	972.33 cm^{-1}	41:	1164.23 cm^{-1}	50:	2046.59 cm^{-1}
33:	972.55 cm^{-1}	42:	1354.95 cm^{-1}	51:	3220.07 cm^{-1}
34:	997.92 cm^{-1}	43:	1435.86 cm^{-1}	52:	3225.99 cm^{-1}
35:	1006.44 cm^{-1}	44:	1476.74 cm^{-1}	53:	3227.41 cm^{-1}
36:	1036.71 cm^{-1}	45:	1480.22 cm^{-1}	54:	3235.80 cm^{-1}
37:	1038.46 cm^{-1}	46:	1570.28 cm^{-1}	55:	3237.44 cm^{-1}
38:	1040.23 cm^{-1}	47:	1571.03 cm^{-1}	56:	3244.70 cm^{-1}

[Cr(C_6H_5)(CO)$_3$]$^•$ – PBE0-D3BJ/def2-TZVP (D_1)

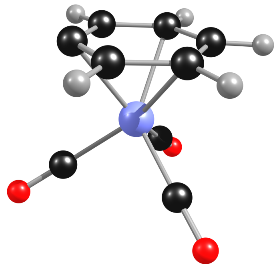

Cr	2.999336000	-0.603634000	-2.607388000
C	2.188381000	-2.111255000	-1.921494000
C	1.958110000	0.427213000	-1.490419000
C	4.265849000	-0.715221000	-1.274005000
O	1.683721000	-3.051928000	-1.507134000
O	1.307598000	1.082493000	-0.811622000
O	5.068866000	-0.779421000	-0.458920000
C	2.732310000	-1.488033000	-4.523121000
C	1.892454000	-0.382574000	-4.489821000
C	4.100794000	-1.491821000	-4.285215000
H	0.826676000	-0.456626000	-4.657249000
H	4.690988000	-2.397840000	-4.298940000
C	2.501243000	0.850662000	-4.155200000
C	4.677334000	-0.242638000	-3.954081000
H	1.894923000	1.742547000	-4.055744000
H	5.728810000	-0.183461000	-3.701335000
C	3.887644000	0.922880000	-3.920656000
H	4.334232000	1.864658000	-3.633731000

0:	0.00 cm^{-1}	11:	121.48 cm^{-1}	22:	561.59 cm^{-1}
1:	0.00 cm^{-1}	12:	317.29 cm^{-1}	23:	614.76 cm^{-1}
2:	0.00 cm^{-1}	13:	335.50 cm^{-1}	24:	619.20 cm^{-1}
3:	0.00 cm^{-1}	14:	348.51 cm^{-1}	25:	655.15 cm^{-1}
4:	0.00 cm^{-1}	15:	401.03 cm^{-1}	26:	658.63 cm^{-1}
5:	0.00 cm^{-1}	16:	418.00 cm^{-1}	27:	659.95 cm^{-1}
6:	19.09 cm^{-1}	17:	420.00 cm^{-1}	28:	686.94 cm^{-1}
7:	84.93 cm^{-1}	18:	483.73 cm^{-1}	29:	810.22 cm^{-1}
8:	86.07 cm^{-1}	19:	485.57 cm^{-1}	30:	857.11 cm^{-1}
9:	98.81 cm^{-1}	20:	487.65 cm^{-1}	31:	880.59 cm^{-1}
10:	119.77 cm^{-1}	21:	561.02 cm^{-1}	32:	963.47 cm^{-1}

33:	973.87 cm^{-1}	40:	1288.74 cm^{-1}	47:	2050.66 cm^{-1}
34:	999.40 cm^{-1}	41:	1406.62 cm^{-1}	48:	2099.71 cm^{-1}
35:	1011.81 cm^{-1}	42:	1432.07 cm^{-1}	49:	3203.16 cm^{-1}
36:	1037.45 cm^{-1}	43:	1457.94 cm^{-1}	50:	3207.20 cm^{-1}
37:	1055.87 cm^{-1}	44:	1509.00 cm^{-1}	51:	3227.18 cm^{-1}
38:	1149.25 cm^{-1}	45:	1554.86 cm^{-1}	52:	3231.93 cm^{-1}
39:	1165.73 cm^{-1}	46:	2047.03 cm^{-1}	53:	3236.91 cm^{-1}

[Cr(C_6H_5)($CO)_3$]$^•$ – PBE0-D3BJ/def2-TZVP/CPCM(THF) (D_1)

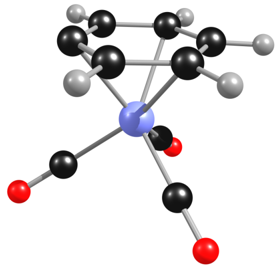

Cr	2.993195000	-0.605248000	-2.598446000
C	2.256326000	-2.124760000	-1.902564000
C	1.908313000	0.373073000	-1.501072000
C	4.239560000	-0.647274000	-1.263238000
O	1.792701000	-3.082651000	-1.465805000
O	1.224268000	0.991161000	-0.813300000
O	5.027166000	-0.672365000	-0.425320000
C	2.697869000	-1.463341000	-4.539158000
C	1.886938000	-0.340582000	-4.490891000
C	4.066602000	-1.507618000	-4.302378000
H	0.819036000	-0.382155000	-4.660104000
H	4.634996000	-2.427950000	-4.326957000
C	2.530064000	0.875942000	-4.152090000
C	4.677217000	-0.277586000	-3.970752000
H	1.946228000	1.782300000	-4.049066000
H	5.732366000	-0.248981000	-3.728223000
C	3.915923000	0.907477000	-3.921003000
H	4.390500000	1.836555000	-3.635707000

0:	0.00 cm^{-1}	4:	0.00 cm^{-1}	8:	85.55 cm^{-1}
1:	0.00 cm^{-1}	5:	0.00 cm^{-1}	9:	94.77 cm^{-1}
2:	0.00 cm^{-1}	6:	-24.91 cm^{-1} *	10:	118.36 cm^{-1}
3:	0.00 cm^{-1}	7:	82.98 cm^{-1}	11:	120.25 cm^{-1}

12:	306.68 cm^{-1}	26:	662.89 cm^{-1}	40:	1284.67 cm^{-1}
13:	331.94 cm^{-1}	27:	664.20 cm^{-1}	41:	1397.94 cm^{-1}
14:	343.85 cm^{-1}	28:	693.60 cm^{-1}	42:	1432.14 cm^{-1}
15:	407.73 cm^{-1}	29:	808.79 cm^{-1}	43:	1454.69 cm^{-1}
16:	424.43 cm^{-1}	30:	868.47 cm^{-1}	44:	1508.17 cm^{-1}
17:	427.11 cm^{-1}	31:	896.32 cm^{-1}	45:	1559.29 cm^{-1}
18:	491.59 cm^{-1}	32:	977.24 cm^{-1}	46:	1991.80 cm^{-1}
19:	494.98 cm^{-1}	33:	979.11 cm^{-1}	47:	1994.44 cm^{-1}
20:	502.09 cm^{-1}	34:	1007.41 cm^{-1}	48:	2055.17 cm^{-1}
21:	565.32 cm^{-1}	35:	1010.94 cm^{-1}	49:	3208.13 cm^{-1}
22:	568.59 cm^{-1}	36:	1032.73 cm^{-1}	50:	3216.90 cm^{-1}
23:	606.96 cm^{-1}	37:	1047.97 cm^{-1}	51:	3225.69 cm^{-1}
24:	617.50 cm^{-1}	38:	1135.86 cm^{-1}	52:	3229.96 cm^{-1}
25:	659.06 cm^{-1}	39:	1144.62 cm^{-1}	53:	3242.11 cm^{-1}

* One negative frequency < 50 cm^{-1} due to arene rotation could not be removed by very tight convergence criteria. The structure was used without further optimization as the energy difference to the real local minimum was expected to be negligible.

$[Cr(C_6H_6)(CO)_3]^+$ – PBE0-D3BJ/def2-TZVP (D_1)

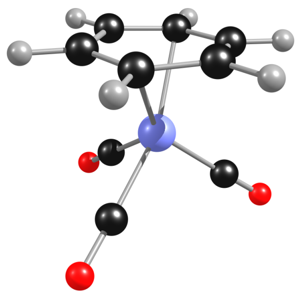

Cr	3.011573000	-0.503186000	-2.532086000
C	2.254434000	-2.074267000	-1.794168000
C	1.712351000	0.372462000	-1.452650000
C	4.334572000	-0.765134000	-1.188329000
O	1.803479000	-3.009245000	-1.345221000
O	0.923341000	0.893494000	-0.834013000
O	5.134631000	-0.935178000	-0.409003000
C	2.638902000	-1.468835000	-4.575019000
C	1.895611000	-0.291747000	-4.484732000
C	4.013169000	-1.469951000	-4.287669000
H	0.825523000	-0.306987000	-4.643733000
H	4.573325000	-2.395201000	-4.298626000
C	2.550849000	0.923404000	-4.176340000
C	4.666224000	-0.247988000	-4.022397000
H	1.978945000	1.839408000	-4.101922000
H	5.730992000	-0.238081000	-3.826602000
C	3.941960000	0.949146000	-4.015295000
H	4.442914000	1.882313000	-3.788663000
H	2.135340000	-2.405042000	-4.785174000

0:	0.00 cm^{-1}	9:	97.65 cm^{-1}	18:	416.81 cm^{-1}
1:	0.00 cm^{-1}	10:	107.91 cm^{-1}	19:	435.54 cm^{-1}
2:	0.00 cm^{-1}	11:	116.29 cm^{-1}	20:	444.74 cm^{-1}
3:	0.00 cm^{-1}	12:	270.98 cm^{-1}	21:	480.66 cm^{-1}
4:	0.00 cm^{-1}	13:	289.90 cm^{-1}	22:	516.93 cm^{-1}
5:	0.00 cm^{-1}	14:	314.77 cm^{-1}	23:	532.42 cm^{-1}
6:	18.10 cm^{-1}	15:	358.57 cm^{-1}	24:	589.84 cm^{-1}
7:	77.17 cm^{-1}	16:	391.22 cm^{-1}	25:	609.67 cm^{-1}
8:	90.70 cm^{-1}	17:	413.21 cm^{-1}	26:	618.49 cm^{-1}

27:	623.95 cm^{-1}	37:	1053.57 cm^{-1}	47:	1597.66 cm^{-1}
28:	687.57 cm^{-1}	38:	1059.12 cm^{-1}	48:	2144.58 cm^{-1}
29:	813.43 cm^{-1}	39:	1182.70 cm^{-1}	49:	2148.99 cm^{-1}
30:	933.16 cm^{-1}	40:	1183.83 cm^{-1}	50:	2198.13 cm^{-1}
31:	940.27 cm^{-1}	41:	1190.53 cm^{-1}	51:	3212.60 cm^{-1}
32:	1004.09 cm^{-1}	42:	1370.35 cm^{-1}	52:	3216.34 cm^{-1}
33:	1009.36 cm^{-1}	43:	1404.50 cm^{-1}	53:	3223.67 cm^{-1}
34:	1019.60 cm^{-1}	44:	1493.72 cm^{-1}	54:	3230.25 cm^{-1}
35:	1032.39 cm^{-1}	45:	1500.09 cm^{-1}	55:	3235.96 cm^{-1}
36:	1041.59 cm^{-1}	46:	1575.42 cm^{-1}	56:	3238.89 cm^{-1}

[Cr(C6H6)(CO)3]+ – PBE0-D3BJ/def2-TZVP/CPCM(THF) (D1)

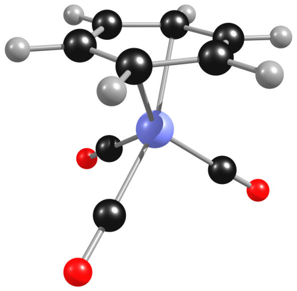

Cr	2.999756000	-0.494473000	-2.532629000
C	2.457177000	-2.121923000	-1.787027000
C	1.566638000	0.198670000	-1.527501000
C	4.303316000	-0.544796000	-1.171423000
O	2.127236000	-3.104866000	-1.321363000
O	0.687174000	0.607543000	-0.938423000
O	5.099269000	-0.588940000	-0.364407000
C	2.536657000	-1.413352000	-4.557836000
C	1.896444000	-0.179041000	-4.497103000
C	3.921366000	-1.512185000	-4.298753000
H	0.826248000	-0.112782000	-4.642859000
H	4.405337000	-2.479377000	-4.298308000
C	2.636203000	0.976048000	-4.166633000
C	4.666993000	-0.350166000	-4.057604000
H	2.130633000	1.926784000	-4.060010000
H	5.730383000	-0.420229000	-3.867359000
C	4.025181000	0.893748000	-3.992597000
H	4.591996000	1.779634000	-3.736533000
H	1.960127000	-2.310911000	-4.743274000

0:	0.00 cm^{-1}	9:	99.48 cm^{-1}	18:	428.23 cm^{-1}
1:	0.00 cm^{-1}	10:	105.44 cm^{-1}	19:	449.80 cm^{-1}
2:	0.00 cm^{-1}	11:	120.96 cm^{-1}	20:	455.00 cm^{-1}
3:	0.00 cm^{-1}	12:	280.80 cm^{-1}	21:	486.91 cm^{-1}
4:	0.00 cm^{-1}	13:	295.02 cm^{-1}	22:	516.71 cm^{-1}
5:	0.00 cm^{-1}	14:	312.76 cm^{-1}	23:	543.39 cm^{-1}
6:	-15.01 cm^{-1} *	15:	372.15 cm^{-1}	24:	605.59 cm^{-1}
7:	77.70 cm^{-1}	16:	408.40 cm^{-1}	25:	611.19 cm^{-1}
8:	86.58 cm^{-1}	17:	420.32 cm^{-1}	26:	620.10 cm^{-1}

27:	636.38 cm^{-1}	37:	1052.98 cm^{-1}	47:	1600.62 cm^{-1}
28:	691.98 cm^{-1}	38:	1058.81 cm^{-1}	48:	2082.47 cm^{-1}
29:	802.90 cm^{-1}	39:	1167.20 cm^{-1}	49:	2088.04 cm^{-1}
30:	932.22 cm^{-1}	40:	1174.08 cm^{-1}	50:	2157.38 cm^{-1}
31:	941.06 cm^{-1}	41:	1181.44 cm^{-1}	51:	3231.00 cm^{-1}
32:	1009.23 cm^{-1}	42:	1365.63 cm^{-1}	52:	3233.83 cm^{-1}
33:	1012.16 cm^{-1}	43:	1407.46 cm^{-1}	53:	3238.26 cm^{-1}
34:	1018.57 cm^{-1}	44:	1485.49 cm^{-1}	54:	3241.95 cm^{-1}
35:	1027.28 cm^{-1}	45:	1494.38 cm^{-1}	55:	3247.00 cm^{-1}
36:	1044.36 cm^{-1}	46:	1573.97 cm^{-1}	56:	3250.77 cm^{-1}

* One negative frequency < 50 cm^{-1} due to arene rotation could not be removed by very tight convergence criteria. The structure was used without further optimization as the energy difference to the real local minimum was expected to be negligible.

$[Cr(C_6H_5)(CO)_3]^+$ – PBE0-D3BJ/def2-TZVP (S_0)

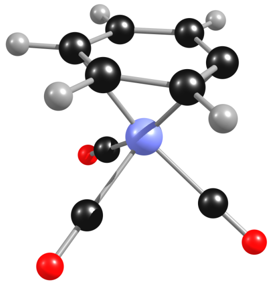

Cr	2.879207000	-0.622580000	-2.588048000
C	2.344250000	-2.304443000	-1.862843000
C	1.918983000	0.509420000	-1.392763000
C	4.168204000	-0.659381000	-1.191781000
O	2.044323000	-3.313080000	-1.454621000
O	1.362782000	1.205978000	-0.699192000
O	4.927618000	-0.681324000	-0.355380000
C	2.680189000	-1.448774000	-4.582341000
C	1.897182000	-0.311267000	-4.623519000
C	4.044202000	-1.501018000	-4.293321000
H	0.844393000	-0.346828000	-4.879724000
H	4.607430000	-2.425520000	-4.307880000
C	2.504315000	0.883724000	-4.197711000
C	4.652182000	-0.272154000	-3.990247000
H	1.924143000	1.796051000	-4.130477000
H	5.708186000	-0.243586000	-3.745470000
C	3.881975000	0.897674000	-3.879605000
H	4.349705000	1.823109000	-3.571150000

0:	0.00 cm^{-1}	11:	115.25 cm^{-1}	22:	514.52 cm^{-1}
1:	0.00 cm^{-1}	12:	267.50 cm^{-1}	23:	536.69 cm^{-1}
2:	0.00 cm^{-1}	13:	286.98 cm^{-1}	24:	585.86 cm^{-1}
3:	0.00 cm^{-1}	14:	316.13 cm^{-1}	25:	600.75 cm^{-1}
4:	0.00 cm^{-1}	15:	353.81 cm^{-1}	26:	612.74 cm^{-1}
5:	0.00 cm^{-1}	16:	386.75 cm^{-1}	27:	617.39 cm^{-1}
6:	22.36 cm^{-1}	17:	405.42 cm^{-1}	28:	662.21 cm^{-1}
7:	77.81 cm^{-1}	18:	407.20 cm^{-1}	29:	833.67 cm^{-1}
8:	90.23 cm^{-1}	19:	430.82 cm^{-1}	30:	908.52 cm^{-1}
9:	97.19 cm^{-1}	20:	437.51 cm^{-1}	31:	948.16 cm^{-1}
10:	108.68 cm^{-1}	21:	475.07 cm^{-1}	32:	982.99 cm^{-1}

33: 1011.53 cm^{-1}
34: 1012.50 cm^{-1}
35: 1031.34 cm^{-1}
36: 1037.60 cm^{-1}
37: 1073.50 cm^{-1}
38: 1164.14 cm^{-1}
39: 1177.75 cm^{-1}
40: 1302.34 cm^{-1}
41: 1393.14 cm^{-1}
42: 1442.84 cm^{-1}
43: 1457.88 cm^{-1}
44: 1533.50 cm^{-1}
45: 1567.16 cm^{-1}
46: 2149.77 cm^{-1}
47: 2156.71 cm^{-1}
48: 2203.74 cm^{-1}
49: 3206.20 cm^{-1}
50: 3206.27 cm^{-1}
51: 3220.95 cm^{-1}
52: 3227.11 cm^{-1}
53: 3236.92 cm^{-1}

$[Cr(C_6H_5)(CO)_3]^+$ – PBE0-D3BJ/def2-TZVP/CPCM(THF) (S_0)

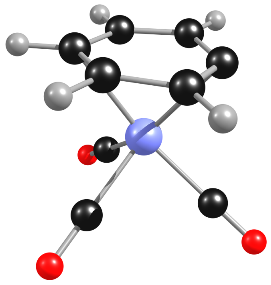

Cr	2.875093000	-0.623577000	-2.588348000
C	2.308246000	-2.278096000	-1.879385000
C	1.989995000	0.535530000	-1.390620000
C	4.155398000	-0.713557000	-1.219296000
O	1.985303000	-3.283832000	-1.467597000
O	1.474994000	1.253989000	-0.680626000
O	4.918293000	-0.766678000	-0.380294000
C	2.709662000	-1.455870000	-4.604965000
C	1.900073000	-0.338916000	-4.634469000
C	4.063277000	-1.486786000	-4.282587000
H	0.846610000	-0.394268000	-4.882034000
H	4.641230000	-2.401775000	-4.268458000
C	2.482203000	0.867664000	-4.203739000
C	4.648611000	-0.244062000	-3.976155000
H	1.879283000	1.763669000	-4.125364000
H	5.697275000	-0.200695000	-3.705684000
C	3.859260000	0.910639000	-3.888732000
H	4.304462000	1.842621000	-3.567721000

0:	0.00 cm^{-1}	11:	111.05 cm^{-1}	22:	516.83 cm^{-1}
1:	0.00 cm^{-1}	12:	272.83 cm^{-1}	23:	545.81 cm^{-1}
2:	0.00 cm^{-1}	13:	296.23 cm^{-1}	24:	595.28 cm^{-1}
3:	0.00 cm^{-1}	14:	312.79 cm^{-1}	25:	603.83 cm^{-1}
4:	0.00 cm^{-1}	15:	371.71 cm^{-1}	26:	615.75 cm^{-1}
5:	0.00 cm^{-1}	16:	395.78 cm^{-1}	27:	631.22 cm^{-1}
6:	28.29 cm^{-1}	17:	410.15 cm^{-1}	28:	663.11 cm^{-1}
7:	78.09 cm^{-1}	18:	429.22 cm^{-1}	29:	816.47 cm^{-1}
8:	88.59 cm^{-1}	19:	446.19 cm^{-1}	30:	897.69 cm^{-1}
9:	97.80 cm^{-1}	20:	448.38 cm^{-1}	31:	941.04 cm^{-1}
10:	105.27 cm^{-1}	21:	484.67 cm^{-1}	32:	985.68 cm^{-1}

33: 1011.90 cm^{-1}
34: 1014.07 cm^{-1}
35: 1034.36 cm^{-1}
36: 1040.46 cm^{-1}
37: 1071.48 cm^{-1}
38: 1154.26 cm^{-1}
39: 1163.98 cm^{-1}
40: 1295.06 cm^{-1}
41: 1389.82 cm^{-1}
42: 1438.46 cm^{-1}
43: 1454.78 cm^{-1}
44: 1528.61 cm^{-1}
45: 1577.81 cm^{-1}
46: 2097.29 cm^{-1}
47: 2104.63 cm^{-1}
48: 2163.71 cm^{-1}
49: 3219.01 cm^{-1}
50: 3219.61 cm^{-1}
51: 3236.14 cm^{-1}
52: 3237.41 cm^{-1}
53: 3244.92 cm^{-1}

[Cr(C_6H_5)(CO)$_3$]$^+$ – PBE0-D3BJ/def2-TZVP (T_1)

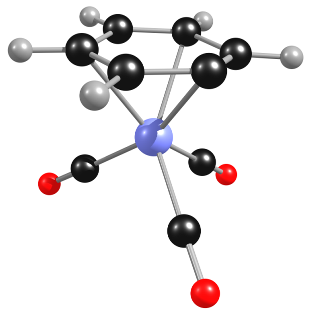

Cr	3.050387000	-0.679980000	-2.569055000
C	2.376595000	-2.321363000	-1.840257000
C	1.714558000	0.123939000	-1.499235000
C	4.204567000	-0.215476000	-1.117235000
O	1.967046000	-3.285442000	-1.422351000
O	0.914332000	0.604321000	-0.860219000
O	4.888885000	0.077310000	-0.268638000
C	2.585272000	-1.373133000	-4.622004000
C	1.889121000	-0.199178000	-4.415648000
C	3.942763000	-1.558180000	-4.434377000
H	0.813451000	-0.136530000	-4.523893000
H	4.410860000	-2.530203000	-4.537729000
C	2.671921000	0.952617000	-4.111714000
C	4.689049000	-0.420950000	-4.056646000
H	2.188750000	1.911173000	-3.964315000
H	5.753203000	-0.503166000	-3.872836000
C	4.048874000	0.834562000	-3.953570000
H	4.629633000	1.705677000	-3.676355000

0:	0.00 cm^{-1}	10:	104.71 cm^{-1}	20:	451.87 cm^{-1}
1:	0.00 cm^{-1}	11:	116.51 cm^{-1}	21:	481.45 cm^{-1}
2:	0.00 cm^{-1}	12:	276.45 cm^{-1}	22:	504.60 cm^{-1}
3:	0.00 cm^{-1}	13:	291.93 cm^{-1}	23:	505.39 cm^{-1}
4:	0.00 cm^{-1}	14:	304.29 cm^{-1}	24:	585.40 cm^{-1}
5:	0.00 cm^{-1}	15:	353.72 cm^{-1}	25:	599.78 cm^{-1}
6:	24.49 cm^{-1}	16:	377.88 cm^{-1}	26:	609.44 cm^{-1}
7:	74.09 cm^{-1}	17:	403.79 cm^{-1}	27:	622.93 cm^{-1}
8:	89.02 cm^{-1}	18:	417.26 cm^{-1}	28:	665.64 cm^{-1}
9:	97.29 cm^{-1}	19:	431.30 cm^{-1}	29:	834.29 cm^{-1}

30: 910.73 cm^{-1}
31: 951.45 cm^{-1}
32: 983.34 cm^{-1}
33: 994.08 cm^{-1}
34: 1013.85 cm^{-1}
35: 1035.81 cm^{-1}
36: 1046.37 cm^{-1}
37: 1059.46 cm^{-1}
38: 1162.40 cm^{-1}
39: 1183.73 cm^{-1}
40: 1295.50 cm^{-1}
41: 1386.66 cm^{-1}
42: 1452.48 cm^{-1}
43: 1462.57 cm^{-1}
44: 1519.32 cm^{-1}
45: 1591.01 cm^{-1}
46: 2146.25 cm^{-1}
47: 2164.33 cm^{-1}
48: 2204.54 cm^{-1}
49: 3207.69 cm^{-1}
50: 3212.64 cm^{-1}
51: 3221.15 cm^{-1}
52: 3226.01 cm^{-1}
53: 3231.48 cm^{-1}

$[Cr(C_6H_5)(CO)_3]^+$ – PBE0-D3BJ/def2-TZVP/CPCM(THF) (T_1)

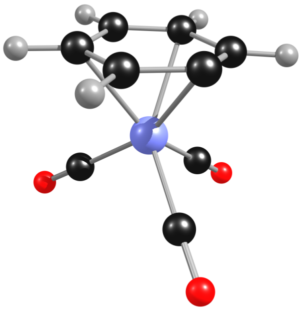

Cr	3.053869000	-0.682140000	-2.567188000
C	2.393047000	-2.306656000	-1.849420000
C	1.717522000	0.096752000	-1.516550000
C	4.186275000	-0.181264000	-1.136357000
O	1.982670000	-3.275686000	-1.430077000
O	0.907053000	0.565222000	-0.872325000
O	4.864570000	0.138709000	-0.286999000
C	2.575809000	-1.366048000	-4.626791000
C	1.891527000	-0.187767000	-4.417340000
C	3.932011000	-1.564476000	-4.439385000
H	0.814727000	-0.115138000	-4.505027000
H	4.391083000	-2.541778000	-4.528108000
C	2.681603000	0.952721000	-4.097632000
C	4.689060000	-0.433010000	-4.070445000
H	2.201350000	1.907952000	-3.924811000
H	5.749904000	-0.528749000	-3.876617000
C	4.060007000	0.823643000	-3.948590000
H	4.647184000	1.683713000	-3.652413000

0:	0.00 cm^{-1}	10:	112.36 cm^{-1}	20:	462.15 cm^{-1}
1:	0.00 cm^{-1}	11:	115.30 cm^{-1}	21:	490.76 cm^{-1}
2:	0.00 cm^{-1}	12:	279.30 cm^{-1}	22:	510.30 cm^{-1}
3:	0.00 cm^{-1}	13:	295.09 cm^{-1}	23:	524.39 cm^{-1}
4:	0.00 cm^{-1}	14:	308.43 cm^{-1}	24:	593.93 cm^{-1}
5:	0.00 cm^{-1}	15:	364.90 cm^{-1}	25:	605.07 cm^{-1}
6:	18.34 cm^{-1}	16:	391.95 cm^{-1}	26:	613.55 cm^{-1}
7:	80.32 cm^{-1}	17:	420.93 cm^{-1}	27:	631.66 cm^{-1}
8:	95.40 cm^{-1}	18:	435.33 cm^{-1}	28:	668.33 cm^{-1}
9:	105.76 cm^{-1}	19:	441.17 cm^{-1}	29:	819.10 cm^{-1}

30:	904.39 cm^{-1}	38:	1154.09 cm^{-1}	46:	2097.79 cm^{-1}
31:	946.95 cm^{-1}	39:	1165.97 cm^{-1}	47:	2107.97 cm^{-1}
32:	986.24 cm^{-1}	40:	1286.28 cm^{-1}	48:	2165.72 cm^{-1}
33:	993.46 cm^{-1}	41:	1383.85 cm^{-1}	49:	3218.95 cm^{-1}
34:	1013.62 cm^{-1}	42:	1446.43 cm^{-1}	50:	3224.41 cm^{-1}
35:	1031.52 cm^{-1}	43:	1459.12 cm^{-1}	51:	3230.51 cm^{-1}
36:	1048.65 cm^{-1}	44:	1521.70 cm^{-1}	52:	3233.97 cm^{-1}
37:	1058.92 cm^{-1}	45:	1590.53 cm^{-1}	53:	3240.08 cm^{-1}

[Cr(C$_6$H$_6$)(CO)$_3$]$^-$ – PBE0-D3BJ/def2-TZVP (D$_1$)

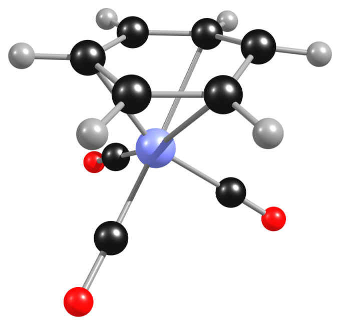

Cr	3.027302000	-0.714989000	-2.517064000
C	2.233756000	-2.176133000	-1.811767000
C	2.008378000	0.387252000	-1.482208000
C	4.255826000	-0.877611000	-1.203119000
O	1.739511000	-3.123638000	-1.348009000
O	1.362353000	1.074917000	-0.802847000
O	5.042910000	-1.001792000	-0.353310000
C	2.624210000	-1.455453000	-4.555527000
C	1.880550000	-0.276864000	-4.530546000
C	4.024851000	-1.437753000	-4.297984000
H	0.805421000	-0.344678000	-4.653162000
H	4.614645000	-2.342265000	-4.333904000
C	2.517442000	1.009319000	-4.494514000
C	4.609983000	-0.184400000	-3.959000000
H	1.910380000	1.895067000	-4.361367000
H	5.641303000	-0.151650000	-3.628644000
C	3.841942000	0.978629000	-3.941232000
H	4.307288000	1.896904000	-3.600994000
H	2.120084000	-2.405478000	-4.686445000

0:	0.00 cm^{-1}	9:	89.90 cm^{-1}	18:	501.63 cm^{-1}
1:	0.00 cm^{-1}	10:	103.08 cm^{-1}	19:	505.46 cm^{-1}
2:	0.00 cm^{-1}	11:	104.01 cm^{-1}	20:	515.44 cm^{-1}
3:	0.00 cm^{-1}	12:	187.62 cm^{-1}	21:	547.99 cm^{-1}
4:	0.00 cm^{-1}	13:	208.63 cm^{-1}	22:	553.50 cm^{-1}
5:	0.00 cm^{-1}	14:	310.91 cm^{-1}	23:	564.02 cm^{-1}
6:	41.02 cm^{-1}	15:	374.14 cm^{-1}	24:	600.86 cm^{-1}
7:	75.80 cm^{-1}	16:	437.71 cm^{-1}	25:	612.47 cm^{-1}
8:	78.90 cm^{-1}	17:	460.49 cm^{-1}	26:	651.93 cm^{-1}

27: 653.76 cm^{-1}
28: 681.90 cm^{-1}
29: 711.01 cm^{-1}
30: 752.06 cm^{-1}
31: 795.70 cm^{-1}
32: 830.14 cm^{-1}
33: 935.50 cm^{-1}
34: 940.30 cm^{-1}
35: 961.70 cm^{-1}
36: 997.94 cm^{-1}
37: 1008.29 cm^{-1}
38: 1018.70 cm^{-1}
39: 1079.80 cm^{-1}
40: 1149.60 cm^{-1}
41: 1166.59 cm^{-1}
42: 1334.78 cm^{-1}
43: 1355.17 cm^{-1}
44: 1452.20 cm^{-1}
45: 1455.45 cm^{-1}
46: 1482.36 cm^{-1}
47: 1560.36 cm^{-1}
48: 1916.41 cm^{-1}
49: 1925.04 cm^{-1}
50: 2000.16 cm^{-1}
51: 3170.82 cm^{-1}
52: 3178.63 cm^{-1}
53: 3190.82 cm^{-1}
54: 3199.13 cm^{-1}
55: 3210.03 cm^{-1}
56: 3231.13 cm^{-1}

[Cr(C6H6)(CO)3]⁻ – PBE0-D3BJ/def2-TZVP/CPCM(THF) (D1)

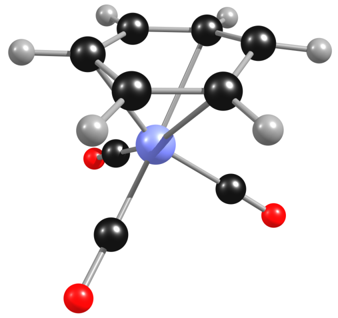

Cr	3.026276000	-0.719058000	-2.512076000
C	2.227514000	-2.166288000	-1.794698000
C	2.021370000	0.374125000	-1.463786000
C	4.237985000	-0.900753000	-1.190849000
O	1.728535000	-3.095340000	-1.298376000
O	1.386873000	1.039939000	-0.749897000
O	4.996432000	-1.039907000	-0.316753000
C	2.625110000	-1.451435000	-4.561719000
C	1.881717000	-0.271111000	-4.539787000
C	4.023269000	-1.433626000	-4.295094000
H	0.809426000	-0.336260000	-4.689485000
H	4.618062000	-2.334210000	-4.357379000
C	2.520415000	1.011582000	-4.494633000
C	4.611854000	-0.180756000	-3.961436000
H	1.919215000	1.906318000	-4.398338000
H	5.648091000	-0.145503000	-3.647164000
C	3.844818000	0.984199000	-3.947618000
H	4.317917000	1.907246000	-3.630266000
H	2.123258000	-2.399778000	-4.712288000

0:	0.00 cm^{-1}	9:	83.71 cm^{-1}	18:	504.37 cm^{-1}
1:	0.00 cm^{-1}	10:	97.75 cm^{-1}	19:	512.34 cm^{-1}
2:	0.00 cm^{-1}	11:	99.84 cm^{-1}	20:	516.05 cm^{-1}
3:	0.00 cm^{-1}	12:	174.40 cm^{-1}	21:	544.23 cm^{-1}
4:	0.00 cm^{-1}	13:	193.22 cm^{-1}	22:	550.73 cm^{-1}
5:	0.00 cm^{-1}	14:	313.16 cm^{-1}	23:	565.70 cm^{-1}
6:	36.05 cm^{-1}	15:	377.44 cm^{-1}	24:	597.55 cm^{-1}
7:	71.49 cm^{-1}	16:	428.10 cm^{-1}	25:	604.80 cm^{-1}
8:	73.76 cm^{-1}	17:	462.05 cm^{-1}	26:	645.39 cm^{-1}

27:	654.86 cm^{-1}	37:	1011.81 cm^{-1}	47:	1550.13 cm^{-1}
28:	671.15 cm^{-1}	38:	1020.98 cm^{-1}	48:	1883.96 cm^{-1}
29:	707.53 cm^{-1}	39:	1079.53 cm^{-1}	49:	1888.58 cm^{-1}
30:	773.49 cm^{-1}	40:	1141.67 cm^{-1}	50:	1970.48 cm^{-1}
31:	820.96 cm^{-1}	41:	1159.14 cm^{-1}	51:	3182.00 cm^{-1}
32:	850.30 cm^{-1}	42:	1334.18 cm^{-1}	52:	3187.59 cm^{-1}
33:	956.19 cm^{-1}	43:	1353.43 cm^{-1}	53:	3198.57 cm^{-1}
34:	957.04 cm^{-1}	44:	1454.30 cm^{-1}	54:	3204.84 cm^{-1}
35:	960.48 cm^{-1}	45:	1458.24 cm^{-1}	55:	3213.04 cm^{-1}
36:	999.01 cm^{-1}	46:	1477.19 cm^{-1}	56:	3231.80 cm^{-1}

$[Cr(C_6H_5)(CO)_3]^-$ – PBE0-D3BJ/def2-TZVP (S_0)

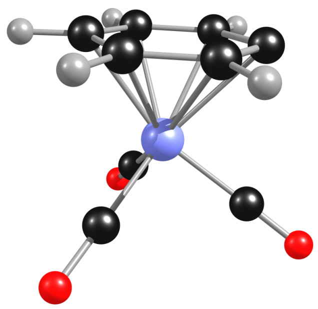

Cr	3.008488000	-0.566762000	-2.563737000
C	2.297930000	-2.130443000	-1.949304000
C	1.909376000	0.368236000	-1.473575000
C	4.209913000	-0.570456000	-1.215131000
O	1.853550000	-3.116583000	-1.545332000
O	1.214566000	0.977819000	-0.769878000
O	4.982161000	-0.556835000	-0.346207000
C	2.605464000	-1.602192000	-4.623146000
C	1.932552000	-0.356951000	-4.505805000
C	3.997662000	-1.499315000	-4.325006000
H	0.853084000	-0.321457000	-4.642814000
H	4.605017000	-2.402818000	-4.318258000
C	2.558628000	0.864297000	-4.159769000
C	4.657716000	-0.302026000	-3.981648000
H	1.971212000	1.771199000	-4.050354000
H	5.714282000	-0.307844000	-3.730892000
C	3.940598000	0.908275000	-3.918841000
H	4.427070000	1.829858000	-3.626378000

0:	0.00 cm^{-1}	10:	113.97 cm^{-1}	20:	515.79 cm^{-1}
1:	0.00 cm^{-1}	11:	117.74 cm^{-1}	21:	567.19 cm^{-1}
2:	0.00 cm^{-1}	12:	289.95 cm^{-1}	22:	574.96 cm^{-1}
3:	0.00 cm^{-1}	13:	332.88 cm^{-1}	23:	599.77 cm^{-1}
4:	0.00 cm^{-1}	14:	334.92 cm^{-1}	24:	633.28 cm^{-1}
5:	0.00 cm^{-1}	15:	391.38 cm^{-1}	25:	678.34 cm^{-1}
6:	36.63 cm^{-1}	16:	434.13 cm^{-1}	26:	685.28 cm^{-1}
7:	84.40 cm^{-1}	17:	440.97 cm^{-1}	27:	694.73 cm^{-1}
8:	85.09 cm^{-1}	18:	503.50 cm^{-1}	28:	718.16 cm^{-1}
9:	97.36 cm^{-1}	19:	511.44 cm^{-1}	29:	801.72 cm^{-1}

30:	892.59 cm^{-1}	38:	1151.35 cm^{-1}	46:	1937.54 cm^{-1}
31:	913.74 cm^{-1}	39:	1179.63 cm^{-1}	47:	1965.80 cm^{-1}
32:	965.07 cm^{-1}	40:	1297.90 cm^{-1}	48:	2033.26 cm^{-1}
33:	967.55 cm^{-1}	41:	1359.21 cm^{-1}	49:	3118.67 cm^{-1}
34:	982.33 cm^{-1}	42:	1416.88 cm^{-1}	50:	3122.78 cm^{-1}
35:	1022.64 cm^{-1}	43:	1429.37 cm^{-1}	51:	3159.57 cm^{-1}
36:	1039.98 cm^{-1}	44:	1526.83 cm^{-1}	52:	3165.52 cm^{-1}
37:	1050.55 cm^{-1}	45:	1539.23 cm^{-1}	53:	3208.85 cm^{-1}

[Cr(C_6H_5)($CO)_3$]$^-$ – PBE0-D3BJ/def2-TZVP/CPCM(THF) (S_0)

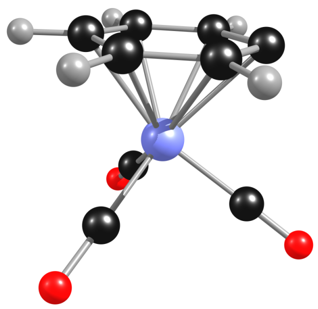

Cr	2.987958000	-0.585248000	-2.573443000
C	2.606006000	-2.205952000	-1.859134000
C	1.671341000	0.138030000	-1.565015000
C	4.117082000	-0.286741000	-1.199018000
O	2.370620000	-3.238300000	-1.391526000
O	0.835180000	0.599795000	-0.908904000
O	4.835695000	-0.088838000	-0.309441000
C	2.428874000	-1.472261000	-4.642847000
C	1.910147000	-0.166366000	-4.475136000
C	3.834278000	-1.550655000	-4.376945000
H	0.842712000	0.006282000	-4.594101000
H	4.329734000	-2.517798000	-4.424050000
C	2.688502000	0.965311000	-4.114665000
C	4.640545000	-0.455323000	-4.024462000
H	2.218550000	1.933373000	-3.979652000
H	5.696276000	-0.590409000	-3.817245000
C	4.062021000	0.826190000	-3.894872000
H	4.663747000	1.674912000	-3.595620000

0:	0.00 cm^{-1}	10:	110.99 cm^{-1}	20:	520.24 cm^{-1}
1:	0.00 cm^{-1}	11:	116.31 cm^{-1}	21:	567.55 cm^{-1}
2:	0.00 cm^{-1}	12:	295.74 cm^{-1}	22:	571.73 cm^{-1}
3:	0.00 cm^{-1}	13:	326.25 cm^{-1}	23:	606.61 cm^{-1}
4:	0.00 cm^{-1}	14:	334.35 cm^{-1}	24:	632.36 cm^{-1}
5:	0.00 cm^{-1}	15:	408.10 cm^{-1}	25:	677.74 cm^{-1}
6:	-14.53 cm^{-1} *	16:	440.14 cm^{-1}	26:	679.60 cm^{-1}
7:	82.34 cm^{-1}	17:	444.75 cm^{-1}	27:	697.56 cm^{-1}
8:	82.68 cm^{-1}	18:	511.51 cm^{-1}	28:	714.87 cm^{-1}
9:	93.94 cm^{-1}	19:	516.85 cm^{-1}	29:	814.80 cm^{-1}

30:	920.20 cm^{-1}	38:	1146.71 cm^{-1}	46:	1925.19 cm^{-1}
31:	933.77 cm^{-1}	39:	1177.99 cm^{-1}	47:	1938.21 cm^{-1}
32:	974.81 cm^{-1}	40:	1304.49 cm^{-1}	48:	2009.61 cm^{-1}
33:	980.84 cm^{-1}	41:	1362.11 cm^{-1}	49:	3142.63 cm^{-1}
34:	993.58 cm^{-1}	42:	1414.36 cm^{-1}	50:	3145.12 cm^{-1}
35:	1025.85 cm^{-1}	43:	1450.60 cm^{-1}	51:	3189.66 cm^{-1}
36:	1040.99 cm^{-1}	44:	1527.52 cm^{-1}	52:	3194.97 cm^{-1}
37:	1050.10 cm^{-1}	45:	1548.85 cm^{-1}	53:	3221.10 cm^{-1}

* One negative frequency < 50 cm^{-1} due to arene rotation could not be removed by very tight convergence criteria. The structure was used without further optimization as the energy difference to the real local minimum was expected to be negligible.

[Cr(C_6H_5)(CO)$_3$]$^-$ – PBE0-D3BJ/def2-TZVP (T_1)

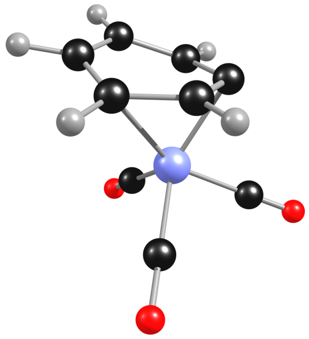

Cr	2.999849000	-0.763682000	-2.359013000
C	2.026801000	-2.226683000	-1.822312000
C	1.959216000	0.402108000	-1.350840000
C	4.283564000	-1.199945000	-1.126587000
O	1.401555000	-3.143969000	-1.490195000
O	1.262347000	1.074273000	-0.715403000
O	5.126392000	-1.482258000	-0.377500000
C	2.790511000	-1.493671000	-4.254839000
C	1.963863000	-0.348855000	-4.308493000
C	4.173818000	-1.321062000	-4.086569000
H	0.887442000	-0.464987000	-4.375618000
H	4.845494000	-2.170485000	-4.031187000
C	2.541557000	0.951600000	-4.553146000
C	4.667942000	-0.003759000	-3.897472000
H	1.914365000	1.799955000	-4.807625000
H	5.704982000	0.154729000	-3.621452000
C	3.868203000	1.118224000	-4.270763000
H	4.321369000	2.104469000	-4.297061000

0:	0.00 cm^{-1}	9:	72.84 cm^{-1}	18:	460.89 cm^{-1}
1:	0.00 cm^{-1}	10:	81.02 cm^{-1}	19:	471.60 cm^{-1}
2:	0.00 cm^{-1}	11:	87.63 cm^{-1}	20:	480.95 cm^{-1}
3:	0.00 cm^{-1}	12:	140.84 cm^{-1}	21:	506.78 cm^{-1}
4:	0.00 cm^{-1}	13:	212.47 cm^{-1}	22:	522.57 cm^{-1}
5:	0.00 cm^{-1}	14:	293.23 cm^{-1}	23:	548.48 cm^{-1}
6:	50.07 cm^{-1}	15:	354.72 cm^{-1}	24:	572.85 cm^{-1}
7:	64.14 cm^{-1}	16:	414.26 cm^{-1}	25:	597.89 cm^{-1}
8:	69.30 cm^{-1}	17:	446.54 cm^{-1}	26:	628.24 cm^{-1}

27:	646.67 cm^{-1}	36:	1000.30 cm^{-1}	45:	1567.64 cm^{-1}
28:	656.86 cm^{-1}	37:	1046.56 cm^{-1}	46:	1927.67 cm^{-1}
29:	695.63 cm^{-1}	38:	1130.47 cm^{-1}	47:	1952.13 cm^{-1}
30:	798.87 cm^{-1}	39:	1149.33 cm^{-1}	48:	1996.06 cm^{-1}
31:	814.40 cm^{-1}	40:	1276.70 cm^{-1}	49:	3154.76 cm^{-1}
32:	863.24 cm^{-1}	41:	1368.09 cm^{-1}	50:	3164.61 cm^{-1}
33:	905.79 cm^{-1}	42:	1390.15 cm^{-1}	51:	3169.66 cm^{-1}
34:	927.49 cm^{-1}	43:	1403.09 cm^{-1}	52:	3182.31 cm^{-1}
35:	977.51 cm^{-1}	44:	1460.86 cm^{-1}	53:	3189.70 cm^{-1}

[Cr(C_6H_5)($CO)_3$]$^-$ – PBE0-D3BJ/def2-TZVP/CPCM(THF) (T_1)

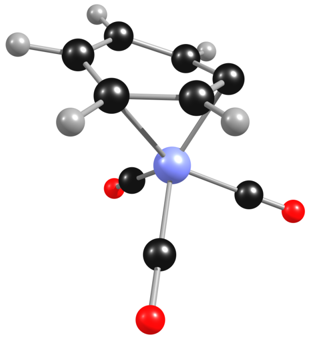

Cr	3.038606000	-0.928402000	-2.440060000
C	1.499840000	-1.794240000	-1.878813000
C	2.579726000	0.410398000	-1.194441000
C	4.305377000	-1.555054000	-1.220818000
O	0.483362000	-2.258426000	-1.588484000
O	2.303226000	1.115028000	-0.324115000
O	5.144424000	-1.840143000	-0.482564000
C	3.146269000	-1.691930000	-4.449723000
C	2.101835000	-0.721407000	-4.484349000
C	4.412048000	-1.146901000	-4.114311000
H	1.077995000	-1.049578000	-4.645610000
H	5.280164000	-1.796108000	-4.031139000
C	2.348902000	0.688986000	-4.547017000
C	4.572152000	0.218233000	-3.781372000
H	1.548203000	1.371901000	-4.816312000
H	5.527140000	0.576049000	-3.411184000
C	3.582165000	1.162215000	-4.194923000
H	3.787833000	2.225379000	-4.140839000

0:	0.00 cm^{-1}	9:	71.55 cm^{-1}	18:	428.99 cm^{-1}
1:	0.00 cm^{-1}	10:	80.71 cm^{-1}	19:	453.40 cm^{-1}
2:	0.00 cm^{-1}	11:	94.23 cm^{-1}	20:	457.92 cm^{-1}
3:	0.00 cm^{-1}	12:	165.89 cm^{-1}	21:	464.99 cm^{-1}
4:	0.00 cm^{-1}	13:	232.41 cm^{-1}	22:	484.48 cm^{-1}
5:	0.00 cm^{-1}	14:	268.12 cm^{-1}	23:	540.66 cm^{-1}
6:	33.48 cm^{-1}	15:	372.00 cm^{-1}	24:	566.90 cm^{-1}
7:	63.98 cm^{-1}	16:	407.48 cm^{-1}	25:	581.88 cm^{-1}
8:	68.91 cm^{-1}	17:	413.39 cm^{-1}	26:	610.75 cm^{-1}

27: 615.76 cm^{-1}
28: 668.40 cm^{-1}
29: 730.26 cm^{-1}
30: 860.70 cm^{-1}
31: 892.64 cm^{-1}
32: 952.86 cm^{-1}
33: 961.33 cm^{-1}
34: 969.32 cm^{-1}
35: 990.95 cm^{-1}
36: 1014.52 cm^{-1}
37: 1055.89 cm^{-1}
38: 1141.32 cm^{-1}
39: 1155.76 cm^{-1}
40: 1290.96 cm^{-1}
41: 1339.79 cm^{-1}
42: 1396.80 cm^{-1}
43: 1435.97 cm^{-1}
44: 1479.75 cm^{-1}
45: 1582.24 cm^{-1}
46: 1917.93 cm^{-1}
47: 1945.77 cm^{-1}
48: 2019.97 cm^{-1}
49: 3142.44 cm^{-1}
50: 3143.45 cm^{-1}
51: 3173.31 cm^{-1}
52: 3183.32 cm^{-1}
53: 3203.93 cm^{-1}

[Mo(ter)$(PH_3)_2(NH_3)]^{•+}$ – PBE0-D3BJ/def2-TZVP (D_1)

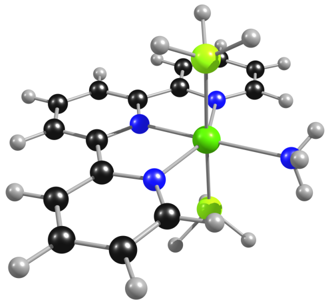

C	0.182060000	-3.121362000	0.229306000
C	0.259183000	-4.420383000	-0.209043000
C	0.287365000	-4.667960000	-1.579061000
C	0.240063000	-3.592990000	-2.437472000
C	0.162435000	-2.296814000	-1.935308000
C	0.117141000	-1.110292000	-2.767751000
C	0.134929000	-1.093284000	-4.152349000
C	0.090189000	0.115356000	-4.826378000
C	0.027835000	1.290563000	-4.096685000
C	0.011396000	1.241079000	-2.713013000
C	-0.052317000	2.385366000	-1.825206000
C	-0.094928000	3.705688000	-2.264123000
C	-0.150165000	4.737725000	-1.354749000
C	-0.158824000	4.422584000	0.001444000
C	-0.114799000	3.102222000	0.376491000
H	0.103590000	0.141181000	-5.907780000
H	3.096190000	0.069172000	1.383255000
H	3.208037000	-0.881078000	-0.498459000
H	3.109341000	1.231131000	-0.379588000
H	-3.229191000	-0.301099000	1.052726000
H	-3.153620000	0.962971000	-0.637500000
H	-3.020930000	-1.136605000	-0.875933000
N	0.129486000	-2.058390000	-0.595024000
N	0.056200000	0.048988000	-2.054371000
N	-0.065022000	2.080176000	-0.498001000
P	2.414015000	0.107845000	0.136566000
P	-2.413825000	-0.119323000	-0.095544000
Mo	0.003375000	-0.002275000	-0.016517000

N	-0.112161000	-0.080689000	2.303746000
H	0.164348000	-2.899950000	1.289846000
H	0.298212000	-5.226697000	0.511596000
H	0.347598000	-5.678656000	-1.961273000
H	0.264370000	-3.749813000	-3.507188000
H	0.183689000	-2.021425000	-4.704902000
H	-0.008308000	2.244101000	-4.605258000
H	-0.083168000	3.915666000	-3.324927000
H	-0.183613000	5.766946000	-1.687618000
H	-0.197489000	5.193169000	0.760193000
H	-0.113558000	2.829792000	1.425192000
H	-0.656253000	-0.874496000	2.628206000
H	-0.573360000	0.737704000	2.689554000
H	0.791496000	-0.142717000	2.762580000

0: 0.00 cm^{-1}
1: 0.00 cm^{-1}
2: 0.00 cm^{-1}
3: 0.00 cm^{-1}
4: 0.00 cm^{-1}
5: 0.00 cm^{-1}
6: -34.51 cm^{-1} *
7: 19.30 cm^{-1}
8: 31.30 cm^{-1}
9: 50.78 cm^{-1}
10: 53.27 cm^{-1}
11: 66.13 cm^{-1}
12: 78.10 cm^{-1}
13: 86.41 cm^{-1}
14: 88.78 cm^{-1}
15: 101.04 cm^{-1}
16: 106.98 cm^{-1}
17: 136.73 cm^{-1}
18: 138.94 cm^{-1}
19: 149.33 cm^{-1}
20: 203.98 cm^{-1}
21: 223.12 cm^{-1}
22: 233.65 cm^{-1}
23: 249.52 cm^{-1}
24: 287.56 cm^{-1}
25: 293.29 cm^{-1}
26: 295.93 cm^{-1}
27: 309.08 cm^{-1}
28: 330.82 cm^{-1}
29: 358.07 cm^{-1}
30: 363.75 cm^{-1}
31: 430.16 cm^{-1}
32: 437.61 cm^{-1}
33: 446.96 cm^{-1}
34: 461.05 cm^{-1}
35: 463.79 cm^{-1}
36: 471.70 cm^{-1}
37: 499.92 cm^{-1}
38: 524.50 cm^{-1}
39: 527.99 cm^{-1}
40: 531.27 cm^{-1}
41: 536.26 cm^{-1}
42: 553.08 cm^{-1}
43: 577.31 cm^{-1}
44: 631.32 cm^{-1}
45: 640.16 cm^{-1}
46: 641.87 cm^{-1}
47: 660.36 cm^{-1}
48: 676.60 cm^{-1}
49: 738.78 cm^{-1}
50: 740.91 cm^{-1}
51: 752.12 cm^{-1}
52: 754.07 cm^{-1}
53: 765.87 cm^{-1}
54: 792.35 cm^{-1}
55: 830.64 cm^{-1}
56: 854.42 cm^{-1}
57: 883.53 cm^{-1}
58: 884.78 cm^{-1}
59: 907.32 cm^{-1}
60: 959.13 cm^{-1}
61: 963.17 cm^{-1}
62: 969.06 cm^{-1}
63: 995.30 cm^{-1}
64: 997.41 cm^{-1}
65: 1013.76 cm^{-1}

66: 1022.05 cm^{-1}	86: 1301.62 cm^{-1}	106: 2374.00 cm^{-1}
67: 1026.57 cm^{-1}	87: 1318.46 cm^{-1}	107: 2389.16 cm^{-1}
68: 1030.28 cm^{-1}	88: 1324.49 cm^{-1}	108: 2389.91 cm^{-1}
69: 1063.71 cm^{-1}	89: 1339.04 cm^{-1}	109: 2393.48 cm^{-1}
70: 1066.51 cm^{-1}	90: 1351.45 cm^{-1}	110: 2395.31 cm^{-1}
71: 1082.41 cm^{-1}	91: 1374.94 cm^{-1}	111: 2401.63 cm^{-1}
72: 1087.51 cm^{-1}	92: 1413.67 cm^{-1}	112: 3195.77 cm^{-1}
73: 1104.37 cm^{-1}	93: 1470.67 cm^{-1}	113: 3197.87 cm^{-1}
74: 1107.81 cm^{-1}	94: 1484.10 cm^{-1}	114: 3216.89 cm^{-1}
75: 1114.38 cm^{-1}	95: 1495.30 cm^{-1}	115: 3217.58 cm^{-1}
76: 1118.64 cm^{-1}	96: 1500.48 cm^{-1}	116: 3219.73 cm^{-1}
77: 1119.81 cm^{-1}	97: 1508.10 cm^{-1}	117: 3228.53 cm^{-1}
78: 1123.03 cm^{-1}	98: 1579.64 cm^{-1}	118: 3230.63 cm^{-1}
79: 1145.71 cm^{-1}	99: 1603.96 cm^{-1}	119: 3235.14 cm^{-1}
80: 1163.63 cm^{-1}	100: 1608.82 cm^{-1}	120: 3237.55 cm^{-1}
81: 1178.48 cm^{-1}	101: 1656.71 cm^{-1}	121: 3241.84 cm^{-1}
82: 1183.65 cm^{-1}	102: 1661.86 cm^{-1}	122: 3244.72 cm^{-1}
83: 1204.15 cm^{-1}	103: 1663.07 cm^{-1}	123: 3502.42 cm^{-1}
84: 1253.88 cm^{-1}	104: 1664.88 cm^{-1}	124: 3570.64 cm^{-1}
85: 1298.02 cm^{-1}	105: 1667.44 cm^{-1}	125: 3575.63 cm^{-1}

* One negative frequency < 50 cm^{-1} due to PH_3 rotation could not be removed by very tight convergence criteria. The structure was used without further optimization as the energy difference to the real local minimum was expected to be negligible.

[Mo(ter)(PH_3)$_2$(NH_2)]$^+$ – PBE0-D3BJ/def2-TZVP (S_0)

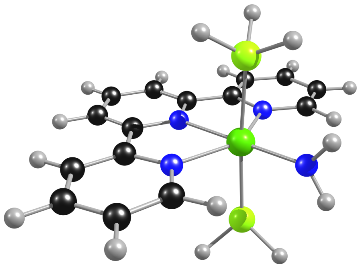

C	0.191982000	-3.034649000	0.196272000
C	0.273141000	-4.341541000	-0.217839000
C	0.314786000	-4.623408000	-1.580270000
C	0.272558000	-3.572034000	-2.467693000
C	0.190357000	-2.266988000	-1.993730000
C	0.139895000	-1.086346000	-2.830653000
C	0.165350000	-1.072011000	-4.216017000
C	0.110106000	0.139210000	-4.883151000
C	0.030553000	1.309021000	-4.148026000
C	0.008059000	1.241513000	-2.764186000
C	-0.072270000	2.370701000	-1.861190000
C	-0.139717000	3.701583000	-2.259592000
C	-0.213480000	4.698638000	-1.313472000
C	-0.218252000	4.336617000	0.030514000
C	-0.149805000	3.007449000	0.369092000
H	0.128862000	0.171160000	-5.964602000
H	3.058142000	0.124632000	1.450150000
H	3.246465000	-0.873503000	-0.413817000
H	3.127261000	1.242742000	-0.352859000
H	-3.048758000	-0.214267000	1.342957000
H	-3.178375000	0.889248000	-0.465703000
H	-3.060103000	-1.227204000	-0.522252000
N	0.150271000	-1.998467000	-0.666696000
N	0.062083000	0.057322000	-2.112013000
N	-0.077231000	2.024117000	-0.551770000
P	2.434497000	0.126137000	0.175929000
P	-2.384945000	-0.143568000	0.091151000
Mo	0.026398000	-0.005984000	0.032549000
N	-0.005023000	-0.063552000	1.992041000

H	0.157484000	-2.772941000	1.245414000
H	0.303314000	-5.130128000	0.522411000
H	0.378788000	-5.643870000	-1.935112000
H	0.302850000	-3.752013000	-3.533636000
H	0.227782000	-1.998961000	-4.769185000
H	-0.013625000	2.267049000	-4.647404000
H	-0.133681000	3.944417000	-3.313422000
H	-0.266485000	5.738361000	-1.609300000
H	-0.274768000	5.080025000	0.814670000
H	-0.150924000	2.684048000	1.401465000
H	0.802066000	-0.035513000	2.606371000
H	-0.831330000	-0.126807000	2.577544000

0:	0.00 cm^{-1}	24:	292.02 cm^{-1}	48:	734.19 cm^{-1}
1:	0.00 cm^{-1}	25:	300.49 cm^{-1}	49:	743.49 cm^{-1}
2:	0.00 cm^{-1}	26:	316.27 cm^{-1}	50:	752.92 cm^{-1}
3:	0.00 cm^{-1}	27:	349.53 cm^{-1}	51:	760.94 cm^{-1}
4:	0.00 cm^{-1}	28:	361.94 cm^{-1}	52:	769.52 cm^{-1}
5:	0.00 cm^{-1}	29:	433.16 cm^{-1}	53:	777.04 cm^{-1}
6:	24.03 cm^{-1}	30:	445.09 cm^{-1}	54:	799.65 cm^{-1}
7:	53.48 cm^{-1}	31:	454.86 cm^{-1}	55:	839.46 cm^{-1}
8:	55.23 cm^{-1}	32:	460.00 cm^{-1}	56:	860.09 cm^{-1}
9:	79.88 cm^{-1}	33:	460.51 cm^{-1}	57:	896.20 cm^{-1}
10:	88.74 cm^{-1}	34:	476.87 cm^{-1}	58:	898.83 cm^{-1}
11:	92.29 cm^{-1}	35:	495.25 cm^{-1}	59:	911.11 cm^{-1}
12:	98.21 cm^{-1}	36:	532.95 cm^{-1}	60:	976.70 cm^{-1}
13:	99.94 cm^{-1}	37:	533.51 cm^{-1}	61:	983.51 cm^{-1}
14:	117.36 cm^{-1}	38:	534.95 cm^{-1}	62:	986.59 cm^{-1}
15:	140.69 cm^{-1}	39:	551.31 cm^{-1}	63:	1007.89 cm^{-1}
16:	143.73 cm^{-1}	40:	593.03 cm^{-1}	64:	1010.26 cm^{-1}
17:	154.66 cm^{-1}	41:	636.85 cm^{-1}	65:	1013.96 cm^{-1}
18:	169.76 cm^{-1}	42:	638.58 cm^{-1}	66:	1020.26 cm^{-1}
19:	227.66 cm^{-1}	43:	651.96 cm^{-1}	67:	1034.28 cm^{-1}
20:	230.67 cm^{-1}	44:	660.84 cm^{-1}	68:	1042.28 cm^{-1}
21:	232.26 cm^{-1}	45:	665.00 cm^{-1}	69:	1058.69 cm^{-1}
22:	236.92 cm^{-1}	46:	672.99 cm^{-1}	70:	1066.96 cm^{-1}
23:	291.00 cm^{-1}	47:	733.46 cm^{-1}	71:	1083.35 cm^{-1}

72: 1092.43 cm^{-1}
73: 1107.73 cm^{-1}
74: 1111.08 cm^{-1}
75: 1117.23 cm^{-1}
76: 1121.77 cm^{-1}
77: 1122.50 cm^{-1}
78: 1123.67 cm^{-1}
79: 1149.32 cm^{-1}
80: 1166.50 cm^{-1}
81: 1185.60 cm^{-1}
82: 1188.75 cm^{-1}
83: 1206.14 cm^{-1}
84: 1301.71 cm^{-1}
85: 1303.75 cm^{-1}
86: 1332.30 cm^{-1}
87: 1333.11 cm^{-1}
88: 1343.06 cm^{-1}
89: 1356.58 cm^{-1}
90: 1387.31 cm^{-1}
91: 1423.37 cm^{-1}
92: 1474.46 cm^{-1}
93: 1488.98 cm^{-1}
94: 1497.51 cm^{-1}
95: 1519.70 cm^{-1}
96: 1522.90 cm^{-1}
97: 1591.54 cm^{-1}
98: 1609.76 cm^{-1}
99: 1611.02 cm^{-1}
100: 1619.01 cm^{-1}
101: 1664.41 cm^{-1}
102: 1669.89 cm^{-1}
103: 1672.18 cm^{-1}
104: 2401.11 cm^{-1}
105: 2406.85 cm^{-1}
106: 2412.46 cm^{-1}
107: 2413.26 cm^{-1}
108: 2416.48 cm^{-1}
109: 2419.97 cm^{-1}
110: 3217.25 cm^{-1}
111: 3219.60 cm^{-1}
112: 3220.85 cm^{-1}
113: 3227.38 cm^{-1}
114: 3230.51 cm^{-1}
115: 3234.30 cm^{-1}
116: 3238.14 cm^{-1}
117: 3241.14 cm^{-1}
118: 3242.61 cm^{-1}
119: 3245.81 cm^{-1}
120: 3246.51 cm^{-1}
121: 3492.57 cm^{-1}
122: 3563.62 cm^{-1}

[Mo(ter)$(PH_3)_2(NH_2)]^+$ – PBE0-D3BJ/def2-TZVP (T_1)

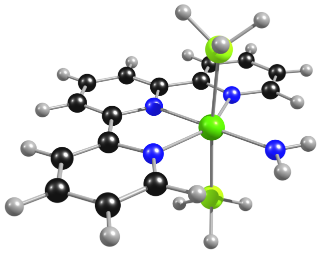

C	0.178987000	-3.128433000	0.208632000
C	0.254479000	-4.428148000	-0.239129000
C	0.295679000	-4.667398000	-1.606747000
C	0.258697000	-3.586377000	-2.463751000
C	0.182342000	-2.296593000	-1.955018000
C	0.138371000	-1.102479000	-2.793832000
C	0.165944000	-1.082971000	-4.178161000
C	0.116212000	0.135680000	-4.838852000
C	0.039998000	1.314066000	-4.111155000
C	0.014775000	1.253424000	-2.727909000
C	-0.062436000	2.396923000	-1.823681000
C	-0.121367000	3.714110000	-2.259328000
C	-0.192219000	4.743715000	-1.343194000
C	-0.202455000	4.425568000	0.008860000
C	-0.142080000	3.102089000	0.383109000
H	0.136857000	0.167020000	-5.920528000
H	3.076296000	1.202247000	0.837093000
H	3.184162000	-0.922490000	0.797413000
H	3.288365000	0.182254000	-1.019033000
H	-3.053692000	-1.252183000	0.658904000
H	-3.163830000	0.871720000	0.727363000
H	-3.198589000	-0.135670000	-1.147482000
N	0.142664000	-2.067590000	-0.612917000
N	0.063838000	0.056141000	-2.094695000
N	-0.073110000	2.090507000	-0.496505000
P	2.487023000	0.119714000	0.145058000
P	-2.441129000	-0.134744000	0.047262000
Mo	0.024993000	-0.003811000	-0.017139000
N	-0.011658000	-0.059889000	2.008405000

H	0.145151000	-2.907195000	1.268149000
H	0.280415000	-5.238610000	0.477399000
H	0.355200000	-5.676180000	-1.994295000
H	0.289000000	-3.740316000	-3.533770000
H	0.225351000	-2.003568000	-4.741782000
H	0.001123000	2.265770000	-4.622486000
H	-0.111431000	3.929926000	-3.318997000
H	-0.238478000	5.773299000	-1.673625000
H	-0.256675000	5.193143000	0.769669000
H	-0.148132000	2.820011000	1.428588000
H	-0.067754000	0.735700000	2.632719000
H	0.023387000	-0.888149000	2.590328000

0:	0.00 cm^{-1}	24:	269.16 cm^{-1}	48:	677.79 cm^{-1}
1:	0.00 cm^{-1}	25:	274.52 cm^{-1}	49:	738.45 cm^{-1}
2:	0.00 cm^{-1}	26:	276.06 cm^{-1}	50:	740.30 cm^{-1}
3:	0.00 cm^{-1}	27:	288.58 cm^{-1}	51:	758.54 cm^{-1}
4:	0.00 cm^{-1}	28:	340.40 cm^{-1}	52:	761.41 cm^{-1}
5:	0.00 cm^{-1}	29:	347.71 cm^{-1}	53:	776.78 cm^{-1}
6:	28.21 cm^{-1}	30:	426.47 cm^{-1}	54:	801.00 cm^{-1}
7:	33.77 cm^{-1}	31:	434.15 cm^{-1}	55:	835.30 cm^{-1}
8:	48.45 cm^{-1}	32:	445.21 cm^{-1}	56:	849.44 cm^{-1}
9:	52.26 cm^{-1}	33:	449.34 cm^{-1}	57:	895.62 cm^{-1}
10:	56.17 cm^{-1}	34:	450.78 cm^{-1}	58:	897.62 cm^{-1}
11:	65.56 cm^{-1}	35:	475.31 cm^{-1}	59:	913.81 cm^{-1}
12:	85.50 cm^{-1}	36:	476.76 cm^{-1}	60:	971.92 cm^{-1}
13:	90.46 cm^{-1}	37:	491.26 cm^{-1}	61:	974.52 cm^{-1}
14:	104.38 cm^{-1}	38:	505.68 cm^{-1}	62:	982.67 cm^{-1}
15:	109.29 cm^{-1}	39:	507.42 cm^{-1}	63:	1005.21 cm^{-1}
16:	111.14 cm^{-1}	40:	533.38 cm^{-1}	64:	1006.54 cm^{-1}
17:	132.41 cm^{-1}	41:	537.51 cm^{-1}	65:	1006.90 cm^{-1}
18:	148.26 cm^{-1}	42:	572.91 cm^{-1}	66:	1007.80 cm^{-1}
19:	193.37 cm^{-1}	43:	609.45 cm^{-1}	67:	1008.65 cm^{-1}
20:	219.77 cm^{-1}	44:	631.81 cm^{-1}	68:	1024.74 cm^{-1}
21:	231.39 cm^{-1}	45:	638.93 cm^{-1}	69:	1050.01 cm^{-1}
22:	249.46 cm^{-1}	46:	642.51 cm^{-1}	70:	1066.37 cm^{-1}
23:	268.38 cm^{-1}	47:	659.49 cm^{-1}	71:	1083.42 cm^{-1}

72: 1090.06 cm^{-1}

73: 1108.98 cm^{-1}

74: 1110.26 cm^{-1}

75: 1116.90 cm^{-1}

76: 1119.62 cm^{-1}

77: 1120.22 cm^{-1}

78: 1123.24 cm^{-1}

79: 1146.58 cm^{-1}

80: 1162.68 cm^{-1}

81: 1182.88 cm^{-1}

82: 1187.06 cm^{-1}

83: 1198.93 cm^{-1}

84: 1299.34 cm^{-1}

85: 1306.14 cm^{-1}

86: 1313.90 cm^{-1}

87: 1317.74 cm^{-1}

88: 1328.51 cm^{-1}

89: 1348.00 cm^{-1}

90: 1364.05 cm^{-1}

91: 1409.47 cm^{-1}

92: 1467.48 cm^{-1}

93: 1487.22 cm^{-1}

94: 1489.36 cm^{-1}

95: 1492.64 cm^{-1}

96: 1506.57 cm^{-1}

97: 1583.35 cm^{-1}

98: 1583.72 cm^{-1}

99: 1611.39 cm^{-1}

100: 1613.62 cm^{-1}

101: 1641.34 cm^{-1}

102: 1659.06 cm^{-1}

103: 1660.83 cm^{-1}

104: 2437.79 cm^{-1}

105: 2438.39 cm^{-1}

106: 2438.71 cm^{-1}

107: 2440.82 cm^{-1}

108: 2445.64 cm^{-1}

109: 2449.00 cm^{-1}

110: 3210.15 cm^{-1}

111: 3210.57 cm^{-1}

112: 3216.14 cm^{-1}

113: 3218.63 cm^{-1}

114: 3219.53 cm^{-1}

115: 3228.87 cm^{-1}

116: 3231.32 cm^{-1}

117: 3235.67 cm^{-1}

118: 3237.60 cm^{-1}

119: 3239.38 cm^{-1}

120: 3244.49 cm^{-1}

121: 3524.65 cm^{-1}

122: 3600.42 cm^{-1}

9.2 Molecular orbitals, spin densities, population analysis and natural bond orbital analysis

Typical single point calculations to obtain energies or wavefunction files for plotting molecular orbitals and spin densities or conducting mayer population analysis were done on the PBE0-D3BJ/def2-TZVPD[30]//PBE0-D3BJ/def2-TZVP level of theory with or without CPCM(THF), to include dispersion as well as diffuse functions in the basis sets paying respect for the ionic character of some of the investigated species. A typical input file is given in the following:

```
! UKS RIJCOSX pbe0 def2-TZVPD D3BJ defgrid3 VeryTightSCF
! Normalprint Printbasis PrintMOs
%geom
maxiter 1000
end
%pal nprocs 6
end
%maxcore 3000
%scf
MaxIter 1000
end
* xyz 0 1
xyz coordinates
*
```

Natural bond orbital (NBO) analysis[13] was conducted by using the NBO implementation within Orca and a typical input file is given in the following:

```
! UKS pbe0 def2-TZVPD D3BJ RIJCOSX defgrid3 TightSCF NBO
! Normalprint Printbasis PrintMOs
%CPCM SMD True
      SMDSOLVENT "THF"
end
%pal nprocs 12
end
%maxcore 3000          #corresponds to 3GB limited scratch memory per core
%scf
MaxIter 1000
end
%nbo
NBOKEYLIST = "$NBO BNDIDX $END"
end
* xyz 0 1
xyz coordinates
*
```

Table S8. Computed bond lengths, Wiberg bond indices (WBI) and Mayer bond orders of Al–C bonds in **1, [1]$^{•-}$**, [**1**]$^{2-}$ and Al_2Me_6. Values obtained on the PBE0-D3BJ/def2-TZVPD//PBE0-D3BJ/def2-TZVP level of theory and by using CPCM(THF).

Compound	d (C–H) / pm	WBI	Mayer
**1**	200.0	0.5654	0.9234
[**1**]$^{•-}$	201.5	0.5500	0.9482
[**1**]$^{2-}$	203.0	0.5340	0.9663
Al_2Me_6	196.4	0.6230	0.9705

Table S9. Computed bond lengths, Wiberg bond indices (WBI) and Mayer bond orders of C–H bonds in neutral, oxidized and reduced CH, acetylene, ethene and benzene. Values obtained on the PBE0-D3BJ/def2-TZVPD//PBE0-D3BJ/def2-TZVP level of theory and by using CPCM(THF).

Compound	d (C–H) / pm	WBI	Mayer
[CH]$^{•}$	111.8	1.0019	1.0887
[CH]$^{-}$	112.5	1.0062	1.2968
[CH]$^{+}$	112.9	0.9984	0.9827
HCCH	106.9	0.9573	1.0625
[HCCH]$^{•-}$	110.0	0.9685	1.1535
[HCCH]$^{•+}$	108.0	0.9460	0.8784
C_2H_4	108.6	0.9325	0.9271
[C_2H_4]$^{•-}$	109.3	0.9852	1.0220
[C_2H_4]$^{•+}$	108.9	0.9081	0.8746
C_6H_6	108.4	0.9172	0.7799
[C_6H_6]$^{•-}$	109.0[a]	0.9244[a]	0.7973[a]
	108.5[b]	0.9343[b]	0.7541[b]
[C_6H_6]$^{•+}$	108.3[a]	0.9000[a]	0.7279[a]
	108.4[b]	0.9104[b]	0.7428[b]

[a] C–H bonds within the C2 axis. [b] C–H bonds not laying within the C2 axis.

Table S10. Computed bond lengths, Wiberg bond indices (WBI) and Mayer bond orders of C–H bonds in neutral, oxidized and reduced methane, formaldehyde, pyrrole, diethyl ether, fluorobenzene and toluene. Values obtained on the PBE0-D3BJ/def2-TZVPD//PBE0-D3BJ/def2-TZVP level of theory and by using CPCM(THF).

Compound	Bond	d / pm	WBI	Mayer
CH_4	C–H	109.1	0.9506	0.9528
$[CH_4]^{\bullet-}$	C–H	111.7	0.9760	0.7397
$[CH_4]^{\bullet+}$	C–H	111.6	0.7847	0.8037
H_2CO	C–H	110.7	0.9134	0.9159
$[H_2CO]^{\bullet-}$	C–H	111.5	0.9463	1.0432
$[H_2CO]^{\bullet+}$	C–H	112.4	0.7685	0.7680
C_4H_5N	N–H	100.7	0.7819	0.9859
$[C_4H_5N]^{\bullet-}$	N–H	101.0	0.8209	0.9127
$[C_4H_5N]^{\bullet+}$	N–H	101.2	0.7457	0.9590
Et_2O	α-C–H	110.1	0.9239	0.9403
$[Et_2O]^{\bullet-}$	α-C–H	111.6	0.9204	0.9143
$[Et_2O]^{\bullet+}$	α-C–H	111.4	0.7923	0.8006
PhF	C–F	134.6	0.9005	0.8116
$[PhF]^{\bullet-}$	C–F	137.0	0.8565	0.7847
$[PhF]^{\bullet+}$	C–F	129.5	1.2322	1.0413
$PhCH_3$	C–CH_3	149.7	1.0401	0.7249
$[PhCH_3]^{\bullet-}$	C–CH_3	149.8	1.0419	0.6396
$[PhCH_3]^{\bullet+}$	C–CH_3	146.1	1.1311	0.9271

Table S11. Computed bond lengths, Wiberg bond indices (WBI) and Mayer bond orders of C–H bonds in neutral, oxidized and reduced water, ammonia, mono silane, sulfur hexafluoride and tricarbonyl chromium benzene complex. Values obtained on the PBE0-D3BJ/def2-TZVPD//PBE0-D3BJ/def2-TZVP level of theory and by using CPCM(THF).

Compound	Bond	d / pm	WBI	Mayer
H_2O	O–H	96.2	0.7717	0.9038
$[H_2O]^{\bullet-}$	O–H	102.5	0.9295	-1.2653
$[H_2O]^{\bullet+}$	O–H	101.3	0.6857	0.7380
NH_3	N–H	101.4	0.8642	0.9756
$[NH_3]^{\bullet-}$	N–H	104.5	0.9293	0.9607
$[NH_3]^{\bullet+}$	N–H	102.2	0.8058	0.8752
SiH_4	Si–H	148.5	0.9609	0.9367
$[SiH_4]^{\bullet-}$	Si–H	151.5[a]	0.9213[a]	0.9849[a]
		161.8[b]	0.7976[b]	0.9536[b]
$[SiH_4]^{\bullet+}$	Si–H	-[c]	-[c]	-[c]
SF_6	S–F	156.5	0.7167	0.9804
$[SF_6]^{\bullet-}$	S–F	160.2[d]	0.6845	0.9920
$Cr(C_6H_6)(CO)_3$	C–H	108.2	0.8957	0.7949
$[Cr(C_6H_6)(CO)_3]^{\bullet-}$	C–H	108.3[e]	0.9104[e]	0.8041[e]
$[Cr(C_6H_6)(CO)_3]^{\bullet+}$	C–H	108.2[e]	0.8869[e]	0.7500[e]

[a] Equatorial Si–H bond. [b] Axial Si–H bond. [c] No minimum structure was found for $[SiH_4]^{\bullet+}$. [d] Reduced SF_6 is not of O_h but of C_{4v} symmetry. Values are given for the shorter S–F bond laying within the C4 axis. [e] All six C–H bonds are slightly different, average value is given.

9.3 Bond dissociation energies (BDEs) and bond orders

Bond dissociation energies (BDEs) of **1**, [**1**]$^{•−}$ and [**1**]$^{2−}$ were computed, based on single point energies (SPEs), which were done as described in section 9.2, according to the following equation:

$$-BDE = SPE(\mathbf{1}) - (SPE([\mathbf{9}]^{•}) + SPE([CH_3]^{•}))$$

The Al–C BDEs within **1**, [**1**]$^{•−}$ and [**1**]$^{2−}$ were determined to 46.5 kcal/mol, 47.6 kcal/mol and 82.6 kcal/mol on the PBE0-D3BJ/def2-TZVPD/CPCM(THF)//PBE0-D3BJ/def2-TZVP/CPCM(THF) level of theory. Using other DFT functionals (BP86,[25,26] TPSS,[27] B3LYP[28]), basis sets, with or without solvent correction or conducting the highly accurate domain-based local pair natural orbital coupled-cluster (DLPNO-CCSD(T))[31] calculations gave values for the BDEs which differed slightly in means of absolute values but always showed the same trend. The BDE is only slightly changing during the first reduction from **1** to [**1**]$^{•−}$, but strongly increased while going to [**1**]$^{2−}$ upon populating the LUMO of **1**. All obtained values are given in the following tables in comparison to the BDE obtained by energy decomposition analysis (EDA), which was conducted using the ADF software package[32] and using the TZ2P[33] basis set, as Ahlrich's basis sets are not available in ADF.

Table S12. Computed BDEs of **1** given in kcal/mol obtained with varying theoretical methods, basis sets and with or without solvent correction.

method	basis	CPCM	-BDE
BP86	def2-TZVP	no	-48.4
TPSS	def2-TZVP	no	-48.7
PBE0	def2-TZVP	no	-50.1
B3LYP	def2-TZVP	no	-49.4
PBE0	def2-TZVPD	no	-49.9
PBE0	def2-TZVPP	no	-50.2
PBE0	def2-QZVP	no	-49.6
PBE0	def2-QZVPD	no	-49.6
**PBE0**	**def2-TZVPD**	**THF**	**-46.5**
PBE0	def2-TZVPP	THF	-46.8
PBE0	def2-QZVPD	THF	-46.2
DLPNO	def2-TZVP	THF	-53.9
PBE0 [a]	TZ2P	no	-49.8

[a] Computation done with ADF instead of Orca.

Table S13. Computed BDEs of [**1**]$^{\bullet-}$ given in kcal/mol obtained with varying theoretical methods, basis sets and with or without solvent correction.

method	basis	CPCM	-BDE
BP86	def2-TZVP	no	-50.3
TPSS	def2-TZVP	no	-50.7
PBE0	def2-TZVP	no	-51.8
B3LYP	def2-TZVP	no	-50.3
PBE0	def2-TZVPD	no	-51.5
PBE0	def2-TZVPP	no	-51.8
PBE0	def2-QZVP	no	-51.2
PBE0	def2-QZVPD	no	-51.1
**PBE0**	**def2-TZVPD**	**THF**	**-47.6**
PBE0	def2-TZVPP	THF	-47.9
PBE0	def2-QZVPD	THF	-47.3
DLPNO	def2-TZVP	THF	-47.0
PBE0 [a]	TZ2P	no	-51.2

[a] Computation done with ADF instead of Orca.

Table S14. Computed BDEs of [**1**]$^{2-}$ given in kcal/mol obtained with varying theoretical methods, basis sets and with or without solvent correction.

method	basis	CPCM	-BDE
BP86	def2-TZVP	no	-73.4
TPSS	def2-TZVP	no	-80.7
PBE0	def2-TZVP	no	-82.7
B3LYP	def2-TZVP	no	-83.5
PBE0	def2-TZVPD	no	-80.0
PBE0	def2-TZVPP	no	-82.8
PBE0	def2-QZVP	no	-81.2
PBE0	def2-QZVPD	no	[a]
**PBE0**	**def2-TZVPD**	**THF**	**-82.6**
PBE0	def2-TZVPP	THF	-83.3
PBE0	def2-QZVPD	THF	-82.5
DLPNO	def2-TZVP	THF	-86.4
PBE0 [b]	TZ2P	no	-81.7

[a] SCF convergence issues when no CPCM was used. [b] Computation done with ADF instead of Orca.

Table S15. Computed BDEs of Al_2Me_6 given in kcal/mol obtained with varying theoretical methods, basis sets and with or without solvent correction.

method	basis	CPCM	-BDE
BP86	def2-TZVP	no	-86.2
TPSS	def2-TZVP	no	-84.4
PBE0	def2-TZVP	no	-86.8
B3LYP	def2-TZVP	no	-87.0
PBE0	def2-TZVPD	no	-86.7
PBE0	def2-TZVPP	no	-86.8
PBE0	def2-QZVP	no	-86.8
PBE0	def2-QZVPD	no	-86.8
**PBE0**	**def2-TZVPD**	**THF**	**-88.1**
PBE0	def2-TZVPP	THF	-88.3
PBE0	def2-QZVPD	THF	-88.3
DLPNO	def2-TZVP	THF	-90.0
PBE0 [a]	TZ2P	no	-87.1

[a] Computation done with ADF instead of Orca.

Table S16. Computed BDEs in kcal/mol of Al–C bonds in **1, [1]**$^{\bullet-}$, [**1**]$^{2-}$ and Al_2Me_6 in comparison with the Al–C atom distance in pm, the Wiberg bond index (WBI) and the Mayer bond order. Values obtained on the PBE0-D3BJ/def2-TZVPD//PBE0-D3BJ/def2-TZVP level of theory and by using CPCM(THF).

Compound	-BDE / kcal*mol^{-1}	ΔE_{Int} / kcal*mol^{-1}	ΔE_{Prep} / kcal*mol^{-1}	ΔE_{Prep} (**9**) / kcal*mol^{-1}	ΔE_{Prep} (CH_3) / kcal*mol^{-1}
**1**	-46.5	-91.7	45.2	34.9	10.3
[**1**]$^{\bullet-}$	-47.6	-96.4	48.7	38.3	10.4
[**1**]$^{2-}$	-82.6	-95.3	12.7	2.2	10.5
Al_2Me_6	-88.1	-98.0	9.8	0.2[a]	9.6

[a] Preparation energy of $[Al_2Me_5]^{\bullet}$

Table S17. Computed BDEs, ΔE_{Int} and ΔE_{Prep} in kcal/mol of C–H bonds in neutral, oxidized and reduced CH, acetylene, ethene and benzene in comparison with the C–H atom distance in pm, the Wiberg bond index (WBI) and the Mayer bond order. Values obtained on the PBE0-D3BJ/def2-TZVPD//PBE0-D3BJ/def2-TZVP level of theory and by using CPCM(THF).

Compound	-BDE / kcal*mol^{-1}	ΔE_{Int} / kcal*mol^{-1}	ΔE_{Prep} / kcal*mol^{-1}
$[CH]^{\bullet}$	-86.8	-86.8	-
$[CH]^{-}$	-85.3	-85.3	-
$[CH]^{+}$	-98.5	-98.5	-
HCCH	-140.0	-134.0	< 0.1
$[HCCH]^{\bullet-}$	-37.2	-129.8	92.6
$[HCCH]^{\bullet+}$	-143.7	-143.7	< 0.1
C_2H_4	-114.6	-117.8	3.2
$[C_2H_4]^{\bullet-}$	-58.4	-115.3	56.9
$[C_2H_4]^{\bullet+}$	-77.4	-124.4	47.0
C_6H_6	-116.1	-118.0	1.9
$[C_6H_6]^{\bullet-}$	-59.0	-113.7[a]<br>-115.9[b]	54.7[a]<br>56.8[b]
$[C_6H_6]^{\bullet+}$	-95.6	-118.1[a]<br>-121.0[b]	25.4<br>22.5

[a] C–H bonds within the C2 axis. [b] C–H bonds not laying within the C2 axis.

Table S18. Computed BDEs, ΔE_{Int} and ΔE_{Prep} in kcal/mol of C–H bonds in neutral, oxidized and reduced methane, formaldehyde, pyrrole, diethyl ether, fluorobenzene and toluene in comparison with the C–H atom distance in pm, the Wiberg bond index (WBI) and the Mayer bond order. Values obtained on the PBE0-D3BJ/def2-TZVPD//PBE0-D3BJ/def2-TZVP level of theory and by using CPCM(THF).

Compound	Bond	-BDE / kcal*mol^{-1}	ΔE_{Int} / kcal*mol^{-1}	ΔE_{Prep} / kcal*mol^{-1}
CH_4	C–H	-109.3	-117.6	8.3
$[CH_4]^{\bullet-}$	C–H	-49.1	-116.8	67.7
$[CH_4]^{\bullet+}$	C–H	-49.8	-134.2	84.4
H_2CO	C–H	-94.4	-95.5	1.1
$[H_2CO]^{\bullet-}$	C–H	-71.6	-94.1	22.5
$[H_2CO]^{\bullet+}$	C–H	-35.3	-172.7	137.4
C_4H_5N	N–H	-102.6	-113.9	11.3
$[C_4H_5N]^{\bullet-}$	N–H	-6.0	-112.5	106.4
$[C_4H_5N]^{\bullet+}$	N–H	-125.9	-131.3	5.5
Et_2O	α-C–H	-97.6	-103.9	6.3
$[Et_2O]^{\bullet-}$	α-C–H	-68.9	-103.4	34.5
$[Et_2O]^{\bullet+}$	α-C–H	-25.2	-116.4	91.3
PhF	C–F	-127.4	-128.1	0.7
$[PhF]^{\bullet-}$	C–F	-75.2	-128.6	53.3
$[PhF]^{\bullet+}$	C–F	-109.7	-131.2	21.5
$PhCH_3$	C–CH_3	-107.4	-120.7	13.3[a]
$[PhCH_3]^{\bullet-}$	C–CH_3	-50.5	-117.1	66.6[b]
$[PhCH_3]^{\bullet+}$	C–CH_3	-95.5	-129.5	33.9[c]

[a] ΔE_{Prep} ($[Ph]^{\bullet}$) = 3.1 and ΔE_{Prep} ($[CH_3]^{\bullet}$) = 10.2. [b] ΔE_{Prep} ($[Ph]^{-}$) = 56.0 and ΔE_{Prep} ($[CH_3]^{\bullet}$) = 10.6. [c] ΔE_{Prep} ($[Ph]^{+}$) = 24.0 and ΔE_{Prep} ($[CH_3]^{\bullet}$) = 9.9.

Table S19. Computed BDEs, ΔE_{Int} and ΔE_{Prep} in kcal/mol of C–H bonds in neutral, oxidized and reduced water, ammonia, mono silane, ferrocene, a chromium arene complex and a prototype of Chirik's molybdenum ammonia complex in comparison with the C–H atom distance in pm, the Wiberg bond index (WBI) and the Mayer bond order. Values obtained on the PBE0-D3BJ/def2-TZVPD//PBE0-D3BJ/def2-TZVP level of theory and by using CPCM(THF).

Compound	Bond	-BDE / $kcal*mol^{-1}$	ΔE_{Int} / $kcal*mol^{-1}$	ΔE_{Prep} / $kcal*mol^{-1}$
H_2O	O–H	-121.8	-121.9	0.1
$[H_2O]^{\bullet -}$	O–H	-40.1	-121.3	81.2
$[H_2O]^{\bullet +}$	O–H	-133.6	-134.2	0.6
NH_3	N–H	-112.7	-113.0	0.2
$[NH_3]^{\bullet -}$	N–H	-40.9	-113.0	72.2
$[NH_3]^{\bullet +}$	N–H	-135.8	-142.9	7.1
SiH_4	Si–H	-92.0	-92.1	0.2
$[SiH_4]^{\bullet -}$	Si–H	-30.2	-83.4[a]<br>-106.5[b]	53.2[a]<br>76.4[b]
$[SiH_4]^{\bullet +}$	Si–H	-[c]	-[c]	-[c]
SF_6	S–F	-101.9	-103.8	1.9
$[SF_6]^{\bullet -}$	S–F	-40.3	-110.8[d]	70.6[d]
$Cr(C_6H_6)(CO)_3$	C–H	-116.7	-118.8	2.1
$[Cr(C_6H_6)(CO)_3]^{\bullet -}$	C–H	-64.1	-116.0	52.0
$[Cr(C_6H_6)(CO)_3]^{\bullet +}$	C–H	-118.8	-120.5	1.7

[a] Equatorial Si–H bond. [b] Axial Si–H bond. [c] No minimum structure was found for $[SiH_4]^{\bullet +}$. [d] Reduced SF_6 is not of O_h but of C_{4v} symmetry. Values are given for the shorter S–F bond laying within the C4 axis.

9.4 Energy decomposition analysis (EDA)

EDA was performed on the PBE0-D3BJ/TZ2P level of theory by using Orca and ADF. The interaction between bond-forming fragments has been decomposed within the EDA[34–37] into the following terms:

$$\Delta E_{Int} = \Delta E_{Elstat} + \Delta E_{Pauli} + \Delta E_{Orb} + \Delta E_{Disp}$$

The term ΔE_{Elstat} corresponds to the classical electrostatic interaction between the unperturbed charge distributions of the deformed reactants and is usually attractive. The Pauli repulsion ΔE_{Pauli} comprises the destabilizing interactions between occupied orbitals and is responsible for any steric repulsion. The orbital interaction ΔE_{Orb} accounts for charge transfer (interaction between occupied orbitals on one moiety with unoccupied orbitals on the other, including HOMO–LUMO interactions) and polarization (empty-occupied orbital mixing on one fragment due to the presence of another fragment). Finally, the ΔE_{Disp} term takes into account the interactions which are due to dispersion forces. As recommended by the ADF software developer no solvent correction was used during decomposition of ΔE_{Int}. Used geometries were obtained by geometry optimization on the PBE0-D3BJ/def2-TZVP level of theory using Orca as described in section 9.1. The geometries of the fragments were obtained by taking the optimized geometry of the full molecule and “deleting” the atoms of the other fragment and vice versa. The wave function files (.rkf) of single point calculations of the fragments were used as input for the EDA calculation, while the spin polarization values of the fragment had opposite signs. We note that the values for ΔE_{Prep} and ΔE_{Int}, as well as all its parts; strongly depend on the electronic state of the fragments. In cases of homolysis, the fragments must show an unpaired electron, or better said the spin density, at the bond-forming atoms to get reasonable values by the EDA. If the homolysis of a closed-shell molecule was investigated, the fragments were treated as doublet-species and if not stated otherwise all herein presented fragments showed correct spin density distributions in the electronic doublet ground state. As oxidation or reduction of molecules can in principle change the spin density distribution within the fragments severely, EDA values of different redox states of a molecule should only be compared if the spin density (in %) at the bond-forming atoms within both fragments (SD_{Atom}) are reasonable. If the homolysis of a radical was investigated, one of the fragments can in principle have a singlet or a triplet ground state. In order to express an unpaired electron (or spin density) at the bond-forming atom, the fragments were

considered in their triplet state. Following Hund’s rule, it is expected that an open-shell singlet configuration lays higher in energy, being less reasonable to use in EDA. The relaxed fragments were considered as singlet or triplet and for the calculation of BDE and ΔE_{Prep} the more stable configuration was used. We want to emphasize that the separation of the BDE into ΔE_{Prep} and ΔE_{Int} is strongly dependent on how much spin density is at located at the bond-forming atom within the fragment for the considered electronic state and there is no physically meaningful justification for this separation. Within this work we use this separation scheme to qualitatively explain trends in redox-induced BDE alterations, which are not predicted by MO theory. All given values should not be discussed in a quantitative manner.

A typical input file for an EDA using ADF is given in the following:

System

atoms

xyz coordinates fragment 1 adf.f=f1

xyz coordinates fragment 2 adf.f=f2

end

Charge 0

end

Task SinglePoint

Engine ADF

Unrestricted

SpinPolarization 0

UnrestrictedFragments

fragments

f1 *path to rkf file*/adf.rkf

f2 *path to rkf file*/adf.rkf

end

eprint

SFO Ovl

end

Print ETSLOWDIN-unrestricted

Relativity

Level None

```
End
beckegrid
quality good
end
ZlmFit
quality good
end
scf
Iterations 100
converge 0.00001 0.0010
end
xc
hybrid pbe0
Dispersion Grimme3 BJDAMP
end
ETSNOCV
Enabled Yes
end
title title of calculation
EndEngine
```

A typical input file for the single point calculations of the fragments using ADF is given in the following:

```
System
atoms
xyz coordinates
end
Charge 0
end
Task SinglePoint
Engine ADF
basis
core none
type TZ2P
end
Unrestricted
SpinPolarization Value for SpinPolarization
Relativity
   Level None
end
beckegrid
quality good
end
ZlmFit
  quality good
end
scf
 Iterations 100
 converge 0.00001 0.0010
end
xc
hybrid pbe0
dispersion Grimme3 BJDAMP
end
title title of calculation
EndEngine
```

Table S20. EDA values of **1, [1]**$^{•–}$, [**1**]$^{2–}$ and Al_2Me_6 in kcal/mol, obtained on the PBE0-D3BJ/TZ2P level of theory. BDE = bond dissociation energy, ΔE_{Int} = interaction energy, SD_{Al} = spin density at aluminum atom within the fragment, ΔE_{Orb} = orbital interaction, ΔE_{Elstat} = electrostatic interaction, ΔE_{Disp} = dispersion interaction, ΔE_{Pauli} = Pauli repulsion, ΔE_{Prep} = preparation energy.

Compound	-BDE	ΔE_{Int}	SD_{Al}	ΔE_{Orb}	ΔE_{Elstat}	ΔE_{Disp}	ΔE_{Pauli}	ΔE_{Prep}
**1**	-49.8	-96.3	0.82	-120.7	-112.0	-3.6	140.0	46.5
[**1**]$^{•–}$	-51.2	-95.7	1.21	-127.1	-123.6	-3.6	158.6	44.5
[**1**]$^{2–}$	-81.7	-94.3	2.03	-133.2	-122.5	-3.6	164.9	12.6
Al_2Me_6	-87.1	-97.1	0.81	-119.0	-118.3	-2.5	142.7	10.1

Table S21. EDA values of neutral, oxidized and reduced CH, acetylene, ethene and benzene in kcal/mol, obtained on the PBE0-D3BJ/TZ2P level of theory. BDE = bond dissociation energy, ΔE_{Int} = interaction energy, SD_C = spin density at bond-forming carbon atom within the fragment, ΔE_{Orb} = orbital interaction, ΔE_{Elstat} = electrostatic interaction, ΔE_{Disp} = dispersion interaction, ΔE_{Pauli} = Pauli repulsion, ΔE_{Prep} = preparation energy.

Compound	-BDE	ΔE_{Int}	SD_C	ΔE_{Orb}	ΔE_{Elstat}	ΔE_{Disp}	ΔE_{Pauli}	ΔE_{Prep}
$[CH]^{\bullet}$	-89.3	-89.3	2.00	-166.6	-51.4	-0.1	128.7	< 0.1
$[CH]^{-}$	-80.8	-80.8	3.00	-129.9	-71.1	-0.1	120.2	< 0.1
$[CH]^{+}$	-128.1	-128.1	1.00	-238.6	-25.6	-0.1	136.2	< 0.1
HCCH	-137.7	-137.7	1.13	-128.1	-46.3	-0.2	36.9	< 0.1
$[HCCH]^{\bullet-}$	-38.5	-127.8	1.16	-140.1	-68.7	-0.2	81.1	89.3
$[HCCH]^{\bullet+}$	-143.2	-143.2	1.53	-144.0	-36.3	-0.2	37.3	< 0.1
C_2H_4	-114.3	-117.3	1.01	-130.6	-61.8	-0.3	75.3	3.0
$[C_2H_4]^{\bullet-}$	-60.4	-114.2	1.46	-119.0	-65.6	-0.3	70.6	53.8
$[C_2H_4]^{\bullet+}$	-79.2	-124.8	1.41	-145.3	-52.1	-0.3	72.8	45.7
C_6H_6	-116.0	-117.7	0.96	-129.2	-64.4	-0.4	76.3	1.7
$[C_6H_6]^{\bullet-}$	-65.3	-116.2[a]	1.53	-117.9	-66.1	-0.4	68.2	51.0
	-65.3	-113.7[b]	1.09	-123.5	-67.5	-0.4	77.7	48.4
$[C_6H_6]^{\bullet+}$	-97.3	-118.3[a]	1.23	-136.8	-59.6	-0.4	78.5	21.0
	-97.3	-121.6[b]	1.03	-138.1	-59.9	-0.4	76.7	24.3

[a] C–H bonds within the C2 axis. [b] C–H bonds not laying within the C2 axis.

Table S22. EDA values of neutral, oxidized and reduced methane, formaldehyde, pyrrole, diethyl ether, fluorobenzene and toluene in kcal/mol, obtained on the PBE0-D3BJ/TZ2P level of theory. BDE = bond dissociation energy, ΔE_{Int} = interaction energy, SD_C = spin density at bond-forming carbon atom within the fragment, ΔE_{Orb} = orbital interaction, ΔE_{Elstat} = electrostatic interaction, ΔE_{Disp} = dispersion interaction, ΔE_{Pauli} = Pauli repulsion, ΔE_{Prep} = preparation energy.

Compound	-BDE	ΔE_{Int}	SD_C	ΔE_{Orb}	ΔE_{Elstat}	ΔE_{Disp}	ΔE_{Pauli}	ΔE_{Prep}
CH_4	-109.4	-116.6	0.98	-129.2	-59.8	-0.2	72.5	7.2
$[CH_4]^{\bullet-}$	-68.8	-116.0	5.27	-124.0	-59.7	-0.2	67.8	47.1
$[CH_4]^{\bullet+}$	-49.1	-136.0	1.55	-161.8	-44.3	-0.2	70.3	86.9
H_2CO	-92.5	-93.4	0.58	-147.6	-72.2	-0.2	126.6	0.9
$[H_2CO]^{\bullet-}$	-74.8	-94.3	1.25	-135.8	-76.5	-0.2	118.2	19.5
$[H_2CO]^{\bullet+}$	-36.1	-167.5	0.95	-168.5	-50.1	-0.2	51.3	131.4
C_4H_5N	-101.8	-115.0	-0.18[a]	-336.1	-121.1	-0.4	342.6	13.1
$[C_4H_5N]^{\bullet-}$	-17.9	-116.3	-0.21[a]	-250.5	-93.9	-0.4	228.4	98.4
$[C_4H_5N]^{\bullet+}$	-129.7	-134.1	0.31[a]	-352.0	-112.1	-0.4	330.4	4.4
Et_2O	-97.5	-103.5	0.87	-133.6	-73.8	-0.4	104.3	6.0
$[Et_2O]^{\bullet-}$	-67.8	-103.7	1.35	-131.0	-73.9	-0.4	101.5	35.9
$[Et_2O]^{\bullet+}$	-25.2	-115.0	0.90	-139.7	-68.2	-0.4	93.2	89.8
PhF	-171.5	-172.3	0.97	-419.9	-174.8	-0.5	422.9	0.8
$[PhF]^{\bullet-}$	-128.6	-175.0	0.90	-403.6	-172.9	-0.5	402.1	46.4
$[PhF]^{\bullet+}$	-155.4	-175.6	1.24	-465.0	-187.1	-0.5	477.1	20.2
$PhCH_3$	-106.7	-118.7	0.96	-171.8	-143.8	-1.8	198.8	12.0
$[PhCH_3]^{\bullet-}$	-57.8	-116.8	1.11	-177.9	-156.5	-1.8	219.3	59.0
$[PhCH_3]^{\bullet+}$	-99.4	-131.0	1.24	-189.5	-143.9	-1.8	204.2	31.7

[a] SD_N = spin density at bond-forming nitrogen atom

Table S23. EDA values of neutral, oxidized and reduced water, ammonia, mono silane, sulfur hexafluoride and a chromium arene complex in kcal/mol, obtained on the PBE0-D3BJ/TZ2P level of theory. BDE = bond dissociation energy, ΔE_{Int} = interaction energy, SD_X = spin density at bond-forming oxygen, nitrogen, silicon or carbon atom within the fragment, ΔE_{Orb} = orbital interaction, ΔE_{Elstat} = electrostatic interaction, ΔE_{Disp} = dispersion interaction, ΔE_{Pauli} = Pauli repulsion, ΔE_{Prep} = preparation energy.

Compound	-BDE	ΔE_{Int}	SP_X	ΔE_{Orb}	ΔE_{Elstat}	ΔE_{Disp}	ΔE_{Pauli}	ΔE_{Prep}
H_2O	-149.5	-149.6	1.03	-398.4	-95.0	-0.1	343.8	0.1
$[H_2O]^{\bullet-}$	-57.5	-125.6	3.41	-279.8	-103.7	-0.1	258.0	68.1
$[H_2O]^{\bullet+}$	-132.5	-132.9	2.05	-266.3	-52.4	-0.1	185.9	0.3
NH_3	-112.2	-112.5	1.05	-221.3	-85.7	-0.1	194.6	0.3
$[NH_3]^{\bullet-}$	-69.0	-113.3	4.15	-191.7	-79.7	-0.1	158.2	44.3
$[NH_3]^{\bullet+}$	-137.2	-144.5	2.01	-200.2	-45.5	-0.1	101.3	7.3
SiH_4	-92.2	-92.4	0.94	-83.5	-48.2	-0.2	39.5	0.2
$[SiH_4]^{\bullet-}$	-31.8	-104.9	2.31	-101.5	-36.5	-0.2	33.4	73.0
	-31.8	-83.3	1.62	-80.7	-47.6	-0.2	45.2	51.5
SF_6	-145.4	-148.3	0.65	-384.5	-164.7	-0.6	401.5	2.9
$[SF_6]^{\bullet-}$	-87.2	-164.1	1.13	-363.2	-147.7	-0.6	347.4	76.9
$Cr(C_6H_6)(CO)_3$	-115.7	-117.8	0.85	-138.6	-66.4	-0.4	87.5	2.1
$[Cr(C_6H_6)(CO)_3]^{\bullet-}$	-70.1	-113.2	0.92	-138.9	-70.8	-0.4	97.0	43.0
$[Cr(C_6H_6)(CO)_3]^{\bullet+}$	-118.5	-119.8	0.97	-139.9	-63.1	-0.4	83.6	1.3

9.5 EPR parameters of $[\mathbf{1}]^{\bullet-}$

The EPR parameters of $[\mathbf{1}]^{\bullet-}$ were computed using the dispersion corrected PBE0-D3BJ functional and the EPR typical IGLO-III basis set and CPCM(THF). The total isotropic *g*-value was determined to 2.0029. Hyperfine coupling constants were calculated for Al. N and H atoms but not for C atoms. due to the minor experimental relevance. due to the low natural abundance of the ^{13}C nucleus.

Table S24. Computed hyperfine couplings constants of $[\mathbf{1}]^{\bullet-}$.

nucleus	A / MHz	nucleus	A / MHz
Al	-0.4393	H1	1.2333
			1.2241
		H2	-0.0289
			-0.0355
N1	3.2971	H3	0.4270
	3.2954		0.4334
	Ø: 3.2963	CH_3:	Ø: 0.5422
N2	1.6218	H4	0.9120
	1.6154		0.9126
	Ø: 1.6186		Ø: 0.9123
N3	-5.1746	H5	-4.8456
			-4.8446
			Ø: 4.8496
		H6	-1.2269
			-1.2316
			Ø: 1.2293
		H7	-1.5314
			-1.5273
			Ø: 1.5294
		H8	-0.8253
			-0.8281
			Ø: 0.8267
		H9	-3.3917
			-3.3882
			Ø: 3.3900

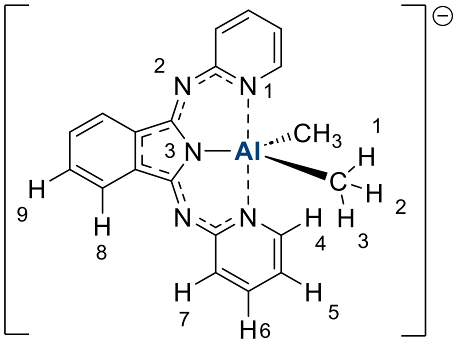

9.6 EDA on molybdenum ammonia complex

Despite the EDA view on chemical bonding is so far seldomly connected to the coordination-induced bond-weakening, we suggest that often the bond-weakening effect can satisfactorily be described by considering pronounced preparation. To show this exemplarily we conducted an EDA on Mo(ter)$(PH_3)_2(NH_3)$ as a prototypical complex for Chirik's compound (**II** in the article). Indeed, the homolysis of the N–H bond is connected to a preparation energy of 36.1 kcal/mol in comparison to only 0.3 kcal/mol in free NH_3.

We note that within the considered unrelaxed Mo(ter)$(PH_3)_2(NH_2)$ fragment the spin population at the nitrogen atom of the NH_2 moeity only amounts to 0.024311 in the electronic ground state. It is expected that the consideration of another electronic state of this fragment gives values for ΔE_{Int} which are even closer to the one obtained for free ammonia (vide supra).

Table S25. EDA values of Mo(ter)$(PH_3)_2(NH_3)$, obtained on the PBE0-D3BJ/TZ2P level of theory. BDE = bond dissociation energy, ΔE_{Int} = interaction energy, ΔE_{Orb} = orbital interaction, ΔE_{Elstat} = electrostatic interaction, ΔE_{Disp} = dispersion interaction, ΔE_{Pauli} = Pauli repulsion, ΔE_{Prep} = preparation energy.

Compound	-BDE	ΔE_{Int}	ΔE_{Orb}	ΔE_{Elstat}	ΔE_{Disp}	ΔE_{Pauli}	ΔE_{Prep}
Mo(ter)$(PH_3)_2(NH_3)$	-56.9	-93.1	-261.8	-106.5	-0.8	276.1	36.1

10. References

[1] W. O. Siegl, *J. Org. Chem.* **1977**, *42*, 1872–1878.

[2] K. Bakthavachalam, N. D. Reddy, *Organometallics* **2013**, *32*, 3174–3184.

[3] G. R. Fulmer, A. J. M. Miller, N. H. Sherden, H. E. Gottlieb, A. Nudelman, B. M. Stoltz, J. E. Bercaw, K. I. Goldberg, *Organometallics* **2010**, *29*, 2176–2179.

[4] L. Van Gerven, J. Talpe, A. Van Itterbeek, *Physica* **1967**, *33*, 207–211.

[5] J. Koziskova, F. Hahn, J. Richter, J. Kožíšek, *Acta Chim. Slovaca* **2016**, *9*, 136–140.

[6] O. V. Dolomanov, L. J. Bourhis, R. J. Gildea, J. A. K. Howard, H. Puschmann, *J. Appl. Crystallogr.* **2009**, *42*, 339–341.

[7] G. M. Sheldrick, *Acta Crystallogr. Sect. A Found. Adv.* **2015**, *71*, 3–8.

[8] G. M. Sheldrick, *Acta Crystallogr. Sect. C Struct. Chem.* **2015**, *71*, 3–8.

[9] I. Noviandri, K. N. Brown, D. S. Fleming, P. T. Gulyas, P. A. Lay, A. F. Masters, L. Phillips, *J. Phys. Chem. B* **1999**, *103*, 6713–6722.

[10] R. E. H. Kuveke, L. Barwise, Y. van Ingen, K. Vashisth, N. Roberts, S. S. Chitnis, J. L. Dutton, C. D. Martin, R. L. Melen, *ACS Cent. Sci.* **2022**, *8*, 855–863.

[11] F. Neese, *Wiley Interdiscip. WIREs Comput. Mol. Sci.* **2012**, *2*, 73–78.

[12] F. Neese, F. Wennmohs, U. Becker, C. Riplinger, *J. Chem. Phys.* **2020**, *152*, 224108.

[13] E. D. Glendening, C. R. Landis, F. Weinhold, *J. Comput. Chem.* **2013**, *34*, 1429–1437.

[14] T. A. Keith, “AIMAll (Version 19.10.12),” **2019**.

[15] Y. Tao, W. Zou, S. Nanayakkara, E. Kraka, *J. Chem. Theory Comput.* **2022**, *18*, 1821–1837.

[16] E. B. Wilson, *The Theory of Infrared and Raman Vibrational Spectra*, McGraw-Hill, New York, **1955**.

[17] J. J. Turner, J. A. Timney, *J. Mol. Spectrosc.* **2022**, *387*, 111662.

[18] J. Baker, P. Pulay, *J. Am. Chem. Soc.* **2006**, *128*, 11324–11325.

[19] J. O. Wenzel, I. Fernández, F. Breher, *Eur. J. Inorg. Chem.* **2023**, *26*, e202300315.

[20] J. O. Wenzel, F. Breher, *Helv. Chim. Acta* **2025**, e00138.

[21] C. Adamo, V. Barone, *J. Chem. Phys.* **1999**, *110*, 6158–6170.

[22] E. Caldeweyher, C. Bannwarth, S. Grimme, *J. Chem. Phys.* **2017**, *147*, 034112.

[23] F. Weigend, R. Ahlrichs, *Phys. Chem. Chem. Phys.* **2005**, *7*, 3297.

[24] M. Bursch, J. Mewes, A. Hansen, S. Grimme, *Angew. Chem. Int. Ed.* **2022**, *61*, e202205735.

[25] J. P. Perdew, *Phys. Rev. B* **1986**, *33*, 8822–8824.

[26] A. D. Becke, *Phys. Rev. A* **1988**, *38*, 3098–3100.

[27] J. Tao, J. P. Perdew, V. N. Staroverov, G. E. Scuseria, *Phys. Rev. Lett.* **2003**, *91*, 146401.

[28] A. D. Becke, *J. Chem. Phys.* **1993**, *98*, 5648–5652.

[29] M. Cossi, N. Rega, G. Scalmani, V. Barone, *J. Comput. Chem.* **2003**, *24*, 669–681.

[30] D. Rappoport, F. Furche, *J. Chem. Phys.* **2010**, *133*, 134105.

[31] Y. Guo, C. Riplinger, U. Becker, D. G. Liakos, Y. Minenkov, L. Cavallo, F. Neese, *J. Chem. Phys.* **2018**, *148*, 011101.

[32] G. te Velde, F. M. Bickelhaupt, E. J. Baerends, C. Fonseca Guerra, S. J. A. van Gisbergen, J. G. Snijders, T. Ziegler, *J. Comput. Chem.* **2001**, *22*, 931–967.

[33] E. Van Lenthe, E. J. Baerends, *J. Comput. Chem.* **2003**, *24*, 1142–1156.

[34] K. Morokuma, *J. Chem. Phys.* **1971**, *55*, 1236–1244.

[35] L. Zhao, M. von Hopffgarten, D. M. Andrada, G. Frenking, *Wiley Interdiscip. Rev. Comput. Mol. Sci.* **2018**, *8*, 1–37.

[36] M. von Hopffgarten, G. Frenking, *Wiley Interdiscip. WIREs Comput. Mol. Sci.* **2012**, *2*, 43–62.

[37] I. Fernández, in *Appl. Theor. Org. Chem.* (Ed.: D.J. Tantillo), World Scientific Publishing, New Jersey, **2018**, pp. 191–226.
